# Structure, Covalency, and Paramagnetism of Homoleptic Actinide and Lanthanide Amidinate Complexes

**DOI:** 10.1021/acs.inorgchem.4c01901

**Published:** 2024-09-02

**Authors:** Boseok Hong, Adrian Näder, Till Sawallisch, Tobias Bode, Sebastian Fichter, Robert Gericke, Peter Kaden, Michael Patzschke, Thorsten Stumpf, Moritz Schmidt, Juliane März

**Affiliations:** † Institute of Resource Ecology, Helmholtz-Zentrum Dresden-Rossendorf (HZDR), Dresden 01328, Germany; ‡ Faculty of Chemistry and Food Chemistry, Technische Universität Dresden, Dresden 01069, Germany; § Institute of Ion Beam Physics and Materials Research, Helmholtz-Zentrum Dresden-Rossendorf (HZDR), Dresden 01328, Germany

## Abstract

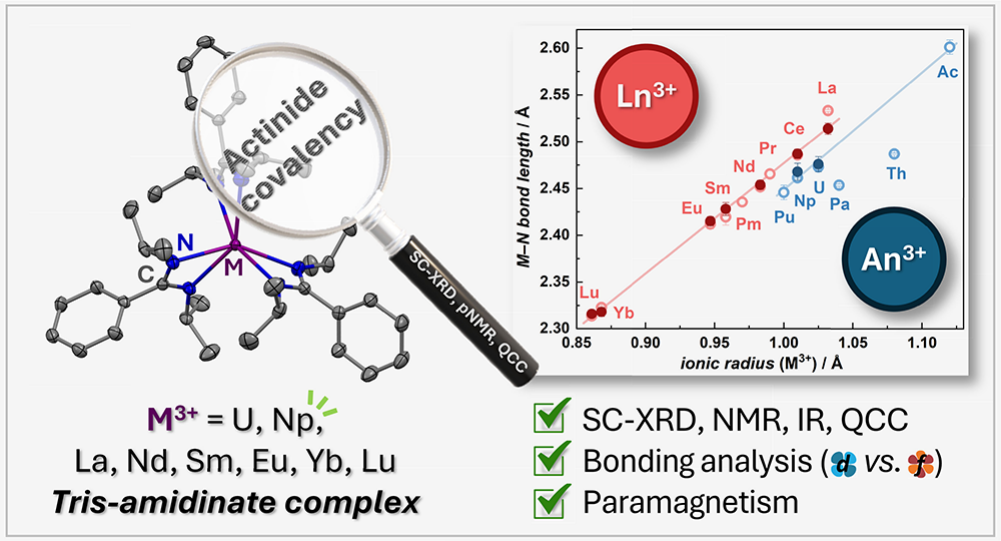

Isostructural trivalent lanthanide and actinide amidinates bearing the *N,N’*-bis(isopropyl)benzamidinate (*i*Pr_2_BA) ligand [Ln^III^/An^III^(*i*Pr_2_BA)_3_] (Ln = La, Nd, Sm, Eu, Yb, Lu; An = U, Np) have been synthesized and characterized in both solid and solution states. All compounds were examined in the solid state utilizing single crystal X-ray diffraction (SC-XRD), revealing a notable deviation in the actinide series with shortened bond lengths compared to the trend in the lanthanide series, suggesting a nonionic contribution to the actinide–ligand bonding. Quantum-chemical bonding analysis further elucidated the nature of these interactions, highlighting increased covalency within the actinide series, as evidenced by higher delocalization indices and greater 5*f* orbital occupation, except for Th(III) and Pa(III), which demonstrated substantial 6*d* orbital occupancies. An in-depth paramagnetic NMR study in solution also sheds light on the covalent character of actinide–ligand bonding, with the separation of pseudocontact (PCS) and contact shift (FCS) contributions employing the Bleaney and Reilley method. This analysis unveiled significant contact contributions in the actinide complexes, indicating enhanced covalency in actinide–ligand bonding. To corroborate these observations, an accurate PCS calculation method based on the Kuprov equation, incorporating both the distribution of electronic spin density and magnetic susceptibility obtained from CASSCF calculations, was applied and compared with experimental values.

## Abstract 2

Exploring the covalency of actinide amidinate complexes: A series of trivalent actinide (U, Np) amidinate and lanthanide (La, Nd, Sm, Eu, Yb, Lu) amidinate complexes have been synthesized and characterized. Notably, single-crystal X-ray diffraction (SC-XRD) results reveal shorter bond lengths in U and Np complexes compared to the lanthanides. Furthermore, intense paramagnetic NMR (pNMR) studies and quantum-chemical bonding analyses indicate enhanced covalency in actinide complexes compared to their lanthanide counterparts.

## 1 Introduction

**Scheme 1:**
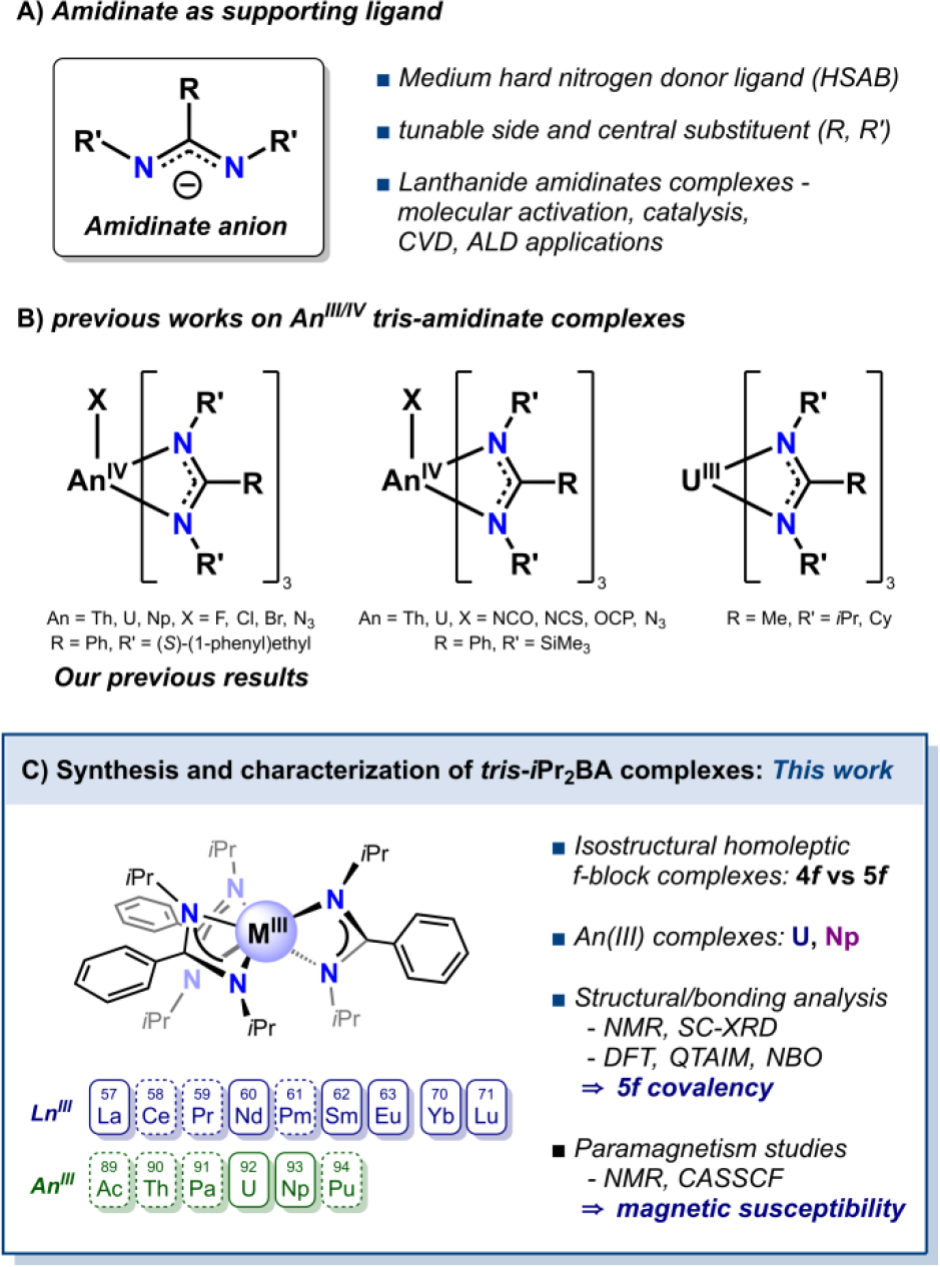
Tris-amidinate *f*-Element Complexes^1−19^

In the dynamic field of metal coordination chemistry, amidinates have emerged as ligands of choice, prized for their ease of preparation and the versatility they afford in modulating both the electronic and steric properties of metal compounds.^1−6^ Their propensity to stabilize various oxidation states of metal ions further enhances their utility and appeal. Among the diverse range of applications, trivalent lanthanide amidinate compounds have gained attention for their roles in molecule activation, catalysis, and in thin film deposition techniques such as chemical vapor deposition (CVD) and atomic layer deposition (ALD), leveraging their volatile properties (Scheme 1A).^2−4,7−11^

Despite these advancements, research related to actinide amidinate complexes remains relatively scarce in comparison to that of their lanthanide counterparts. This is even more pronounced in the case of transuranic elements, where such complexes are exceedingly rare,^12^ underscoring a critical area of potential research and discovery. Our study builds upon previous works on actinide amidinates,^13−16^ such as the Villiers and Arnold groups' development of homoleptic trivalent uranium amidinate complexes via reduction methods, including *N*,*N’*-bis(cyclohexyl)methylamidinate (BCMA) and *N*,*N’*-(isopropyl)methylamidinate (BIMA) complexes,^17,18^ our own group’s report on the synthesis and characterization of a series of tetravalent neptunium amidinate complexes, and the synthesis of enantiomerically pure tetravalent actinide complexes with the chiral (*S*,*S*)-*N*,*N’*-bis(1-phenyl-ethyl)benzamidinate ((*S*)-PEBA) ligand (Scheme 1B).^12,19^

In this paper, we extend this exploration to trivalent *f*-block metal tris-amidinate complexes with the *N*,*N*′-bis(isopropyl)benzamidinate (*i*Pr_2_BA) ligand, presenting a comprehensive study that encompasses synthesis, characterization, and quantum-chemical calculations for bonding analysis (Scheme 1C). Our focus lies on the analysis of the actinide–ligand bonds and their covalency, particularly in comparison to their 4*f* lanthanide homologs. In this context, exploring the paramagnetic properties of actinides holds the potential to be a practical experimental method to probe their electronic behavior and to gather insights on actinide–ligand interactions.^20−29^ Unfortunately, this approach is currently hampered by a lack of reference data for the actinides and a sound fundamental understanding of the electronic and magnetic properties of the actinides. Especially, spin–orbit (SO) coupling and a pronounced tendency to form more covalent bonds limit the usefulness of established methods from lanthanide pNMR, like the Bleaney method. Consequently, new quantum-chemical approaches are required to describe actinide paramagnetism and interpret experimental results from, e.g., pNMR measurements.

Highlighting our findings is the introduction of the first homoleptic Np(III) amidinate complex as well as the in-depth investigation into the magnetic susceptibilities of these amidinate complexes. Bonding trends were analyzed for all *f*-block amidinate complexes, including those that were experimentally unattainable through computational results, thereby offering a comprehensive insight into the behavior of these intriguing compounds and contributing to the ongoing discourse in *f*-block coordination chemistry and paramagnetism.

## 2 Results and Discussion

### 2.1 Synthesis of Ln(III) and An(III) Amidinate Complexes

To facilitate comparison between the 4*f* and 5*f* systems within the same molecular framework, the synthesis of isostructural tris-amidinate complexes is necessary, targeting the trivalent state, a commonly stable oxidation state for lanthanide ions.

**Scheme 2:**
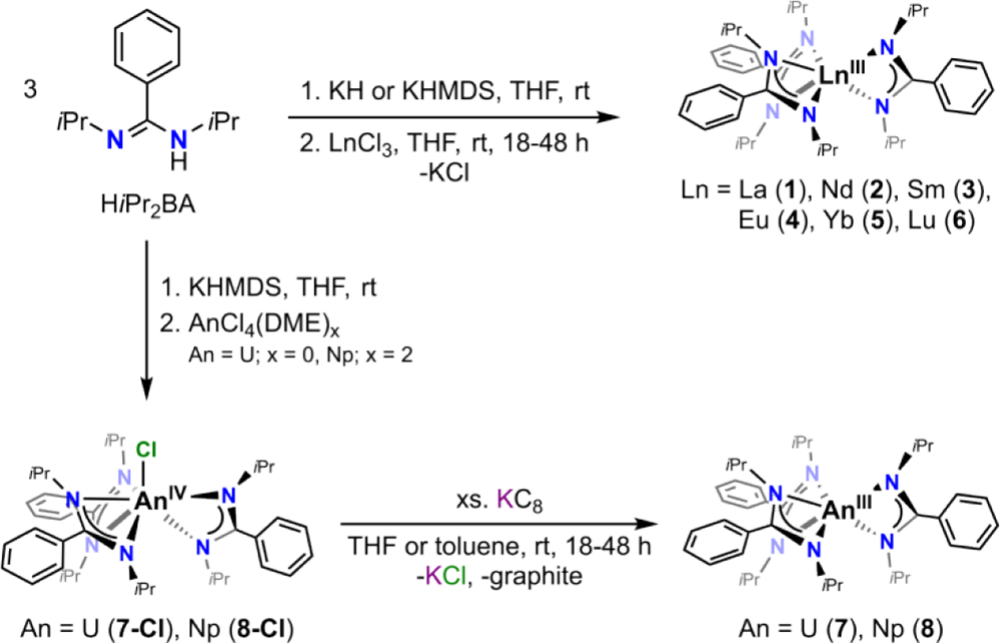
Synthesis of the Homoleptic Trivalent Lanthanide/Actinide Tris-amidinate Complexes (**1**–**8**)

The homoleptic lanthanide tris-amidinate complexes **[Ln**^**III**^**(***i***Pr**_**2**_**BA)**_**3**_**]** (Ln = La (**1**), Nd (**2**), Sm (**3**), Eu (**4**), Yb (**5**), Lu (**6**)) were synthesized via a two-step procedure, involving deprotonation of three equivalents of amidine preligand followed by a salt metathesis with corresponding anhydrous lanthanide trichloride (LnCl_3_) (Scheme 2). Since the lanthanides compounds **1**–**6** were not separable from the protonated ligand and base adduct (e.g., bis(trimethylsilyl)amide; HMDS) in the purification step, ligand deprotonation was carried out using potassium hydride (KH) for all complexes except for the samarium (**3**) reaction, which utilized potassium bis(trimethylsilyl)amide (KHMDS) to avoid the redox side-reactions. These difficulties with the purification prevented the isolation of high-purity products and, consequently, the determination of meaningful reaction yields. When lithium base (e.g., LiHMDS) or deprotonated ligand such as Li-*i*Pr_2_BA was used in the reaction, they exhibited relatively lower conversion compared to the K-*i*Pr_2_BA reaction. This may be attributed to the higher stability and solubility of the Li-*i*Pr_2_BA ligand, resulting in a lower driving force compared to its potassium counterpart.

Furthermore, by adapting procedures from the literature,^12,19^ the synthesis of the actinides(IV) chloro tris-amidinate complexes **[An**^**IV**^**Cl(***i***Pr**_**2**_**BA)**_**3**_**]** (An = U (**7-Cl**), Np (**8-Cl**)) was achieved through the reaction of three equivalents of (*in situ*) deprotonated ligand with tetravalent actinide chloride starting materials. The trivalent actinide complexes **[An**^**III**^**(***i***Pr**_**2**_**BA)**_**3**_**]** (An = U (**7**), Np (**8**)) were subsequently obtained through chemical reduction using an excess amount of KC_8_ (Scheme 2). Both uranium **7** and neptunium **8** complexes can be obtained in THF, yielding dark blue and dark purple homoleptic complexes, respectively. However, due to the instability of the trivalent uranium compound in THF, the reaction condition for the uranium complex was further optimized to be carried out in toluene to mitigate autoxidation and decomposition problems, thus enhancing workup convenience. All trivalent complexes have good solubility in THF, *n*-pentane, benzene, and toluene. All sets of amidinate complexes demonstrated high instability in the presence of moisture and oxygen, with trivalent actinide complexes **7** and **8** being particularly sensitive, undergoing immediate decomposition upon exposure to air and moisture. The consistent structural characteristics of the indicated **[M**^**III**^**(***i***Pr**_**2**_**BA)**_**3**_**]** (**1**–**8**) complexes are additionally proven by their nearly identical IR spectra in the fingerprint region (see Figures S1–S11 in the Supporting Information).

The synthesized isostructural tris-amidinate complexes **1**–**8** were examined in the solid state and in solution utilizing single-crystal XRD and NMR spectroscopy. In particular, a comprehensive paramagnetic NMR study was undertaken, which included research into the complexes’ magnetic susceptibility, allowing for the comparison of experimental data with the outcomes from quantum-chemical calculations. Alongside these analyses, theoretical investigations also explored isostructural complexes such as trivalent promethium, actinium, thorium, protactinium, and plutonium complexes, which exceed the experimental scope of this study and/or the radiological safety limits of our laboratory, and an in-depth analysis of chemical bonding was conducted. This analysis aimed to provide insights into the bonding characteristics of the 5*f* elements and to facilitate a comparative assessment of their properties in relation to the 4*f* elements.

### 2.2 Structural Determination of Complexes

#### Solid-State

The synthesized tris-amidinate complexes **1**–**8** were crystallized via slow evaporation from a saturated *n*-pentane solution at ambient temperature. The obtained crystals were subsequently analyzed using single-crystal X-ray diffraction (SC-XRD) at 100 K and demonstrated isomorphic crystallization in monoclinic space group *C*2/c. An exception is observed with Np complex **8**. At 200 K, **8** crystallizes in the monoclinic space group *C*2/c. However, the formation of a superstructure along the crystallographic *b*-axis is observed upon cooling the crystal to 100 K. Therefore, the molecular structure of **8** was refined in *P*2_1_/*c* at 100 K. However, to ensure a consistent assessment of temperature impact on structural characteristics, comparisons of all structures are based on measurements taken at 100 K (Table 1).

**Table 1:** Selected Average Bond Lengths (Å) and Angles (deg) of Tris-amidinate Compounds [M^III^(*i*Pr_2_BA)_3_] (1–8) Obtained from Single-Crystal X-ray Diffraction Analysis at 100 K in Comparison with Shannon Ionic Radii (SIR)^30^ for hexa-coordinated Ions (Å) and Comprehensive Continuous Shape Measures (CShM)^31^

M =		La (**1**)	Nd (**2**)	Sm (**3**)	Eu (**4**)	Yb (**5**)	Lu (**6**)	U (**7**)	Np (**8**)
space group		*C*2/*c*	*C*2/*c*	*C*2/*c*	*C*2/*c*	*C*2/*c*	*C*2/*c*	*C*2/*c*	*P*2_1_/*c*
SIR (CN = 6, in Å)^30^		1.032	0.983	0.958	0.947	0.868	0.861	1.025	1.01
d(M–N_avg_) (Å)		2.514(6)	2.454(5)	2.428(7)	2.415(5)	2.318(5)	2.316(4)	2.476(8)	2.468(9)
bite angle (deg) (∠N_a_–M–N_b_)^a^		53.3(2)	54.8(1)	55.6(1)	55.9(1)	58.4(3)	58.3(1)	54.0(1)	54.4(1)
torsion angle, τ (deg) (∠N_a_–G1–G2–N_b_)^a^^,^^b^		24.7(8)	26.2(8)	28(1)	28(1)	30.3(7)	30.2(8)	24.8(6)	25(2)
CShM^31^	OC-6	10.8	9.9	9.3	9.2	7.9	7.9	10.5	10.3
	TPR-6	11.8	11.6	12.4	12.1	11.8	11.8	11.4	11.6

a N_a_: Nitrogen atoms at the upper face of the coordination sphere (N1, N2*, N3*; for Np – N1, N3, N5); N_b_: nitrogen atoms at the lower face of the coordination sphere (N1*, N2, N3; for Np – N2, N4, N6).

b G1 is the centroid of N_a_ atoms and G2 is the centroid of N_b_ atoms. For a detailed graphical description, see Table S8 in the Supporting Information.

**Figure 1:**
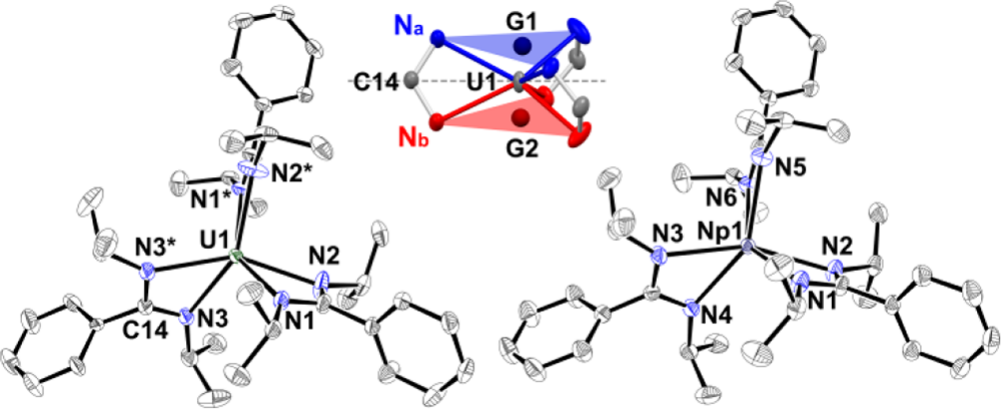
Molecular structure of [U(*i*Pr_2_BA)_3_] (**7**, left) and [Np(*i*Pr_2_BA)_3_] (**8**, right) determined by single-crystal X-ray diffraction analysis. Hydrogen atoms have been omitted for the sake of clarity. Ellipsoids are drawn at the 30 (**7**) and 50% (**8**) probability level, respectively. The asymmetric unit of **7** comprises half of the complex molecule, and C14 and U1 are located on a crystallographically 2-fold rotation axis (symmetry operation *: −*x* + 1, *y*, −z + 3/2). The asymmetric unit of **8** comprises two individual (but conformationally very similar) complex molecules; only one of them is depicted as a representative example. For a detailed structural description and symmetry transformation, see the SI.

The cell parameters for those complexes were comparable to the previously reported cerium complex.^32^ The ligand-to-metal stoichiometry was consistently maintained at a 3:1 ratio, and notably, no additional solvent molecules were coordinated at the metal center, even though the coordination sphere was not fully saturated. Consequently, a 6-fold nitrogen coordination sphere was formed, wherein the bond lengths between the two nitrogen atoms of the bidentate amidinato ligand and the metal were observed to be similar to each other. The molecular structures of the trivalent actinide complexes **7** and **8** are shown in Figure 1 as representatives for the synthesized trivalent amidinate complexes.

The geometry of the coordination sphere was found to be intermediate between trigonal prismatic (TPR-6) and octahedral (OC-6). Within the solid-state structure of the compounds, characterized in *C*2/*c*, the exhibited symmetry involves a *C*_2_ rotational axis connecting the *NCN* carbon of the amidinate ligand at C14 to the metal center (Figure 1). This arrangement results in a disordered phenyl ring at C14. In contrast, the structure within the *P*2_1_/*c* space group of Np **8** demonstrated a well-resolved superstructure. While neither case displayed perfect *C*_3_ rotational symmetry, the deviation of coordination atoms from an ideal *D*_3_ symmetry was marginal (see Table S9 and Figure S30 in the Supporting Information).

To quantitatively evaluate the tilting angle of the amidinate ligand’s binding plane relative to the central metal atom, we determined the centroids (G1: N1–N2*–N3*, G2: N1*–N2–N3) of the nitrogen atoms at the upper (N_a_: N1, N2*, and N3*) and lower faces (N_b_: N1*, N2, and N3) of a triangle prism to create a reference axis (Figure 1). Subsequent measurement of the dihedral angle τ (∠N_a_–G1–G2–N_b_) between the two nitrogen atoms of the amidinate *NCN* backbone (N_a_, N_b_) and the defined centroids revealed the averaged twist angle of the ligands ranged between 24.7(8) and 30.3(7)° (Table 1, see also Tables S7 and S8 in the Supporting Information for detail). An ideal trigonal prismatic coordination would lead to an angle of 0°, and an ideal octahedral coordination would give an angle of 60°. Along the series, a correlation between ionic radius and dihedral angle τ was observed (see Figure S28 in the Supporting Information). Therefore, our tilting angle of the amidinate ligands suggests a coordination polyhedron around the metal ions midway between trigonal prismatic (TPR-6) and octahedral (OC-6).

**Figure 2:**
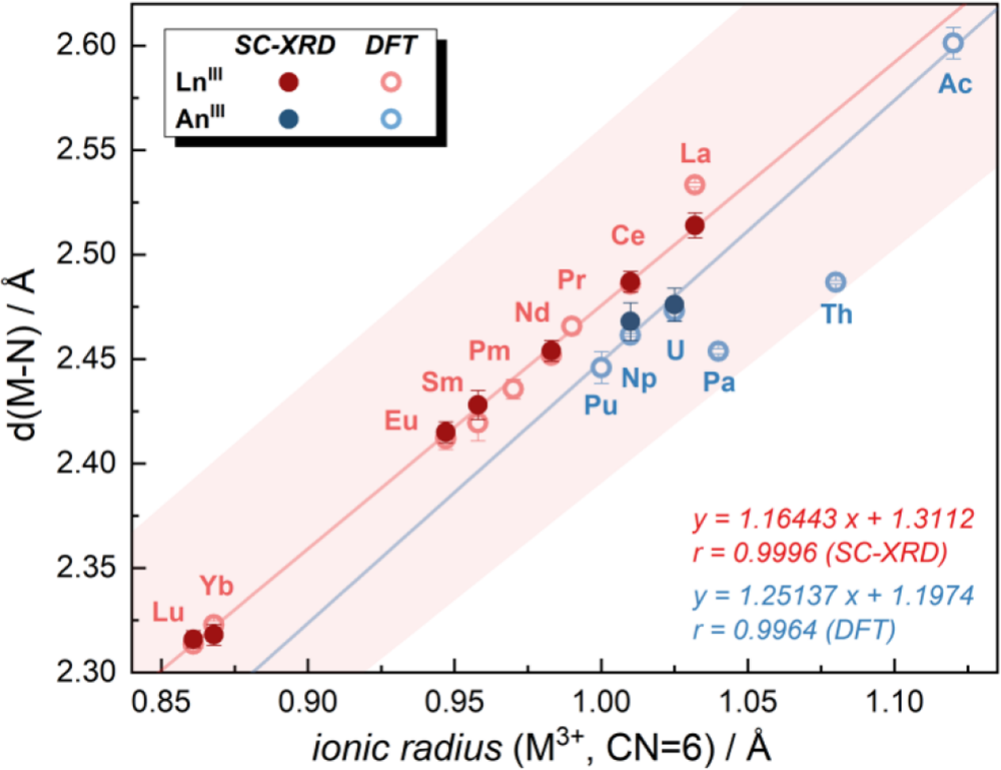
Relation of average bond length (M–N) and ionic radii^30^ of trivalent metal ions with hexa-coordination number (CN) for **[M**^**III**^**(***i***Pr**_**2**_**BA)**_**3**_**]** complexes. The experimental bond length for Ce^**3**+^ was obtained from the literature.^32^ The ionic radius for Th^**3**+^ was taken as 1.08 Å, based on the literature.^33^ The red line represents the linear regression of experimental Ln–N distances with a 3-sigma error range (light red area), while the blue line represents the linear regression of the computationally determined An–N distances without Th and Pa.

When intramolecular bond distances within the lanthanide series are compared, the largest ionic radius of La^3+^ correlates with the greatest average M–N distance of 2.514(6) Å, while the smallest radii of Lu^3+^ is associated with the shortest distances at 2.316(4) Å. In the case of the actinides, U^3+^ exhibits a slightly larger average An–N distance than Np^3+^ (2.476(8) vs. 2.468(9) Å; Table 1). However, the error bars overlap, indicating that this difference is not statistically significant. To compare the lanthanide and actinide series, a plot of M–N distances against Shannon ionic radii (SIR)^30^ (Figure 2) reveals that the M–N distances of trivalent U **7** and Np **8** lie 0.03 and 0.02 Å below the linear regression of the lanthanides, respectively, indicating an increased bond strength within the actinide–nitrogen bonding. Although this observation cannot be directly linked to actinide covalency, it suggests that a further investigation of the bond properties by means of quantum-chemical calculations may be of interest (see below).

To engage in current discussions on covalency in actinide and lanthanide amidinate complexes, it can be beneficial to consider the structural framework proposed by Raymond and Eigenbrot^34^ in 1980, along with recent examples of actinide and lanthanide complexes.^35,36^ It is noted that the effective ligand ionic radius, determined by the difference between the metal–ligand bond length and the metal’s ionic radius, should remain constant for ionic compounds. This constancy is reflected in the linearity and slope of a linear regression plot, where a slope and correlation coefficient (*r*) close to 1 indicate ionic bonding character.

In this regard, the effective ionic radius of the *i*Pr_2_BA ligand is almost invariant, averaging 1.46(1) Å. Based on the experimental results, the Ln^3+^ series regression displayed excellent linearity with an *R*-value of 0.9996 and a slope of 1.16(1) slightly deviating from 1.0 (Figure 2). Considering all experimental data including An^3+^ (U and Np) resulted in only a slight decrease in linearity, with a slope of 1.13(4) and an *R*-value of 0.9953. These findings indicate that the metal–amidinate interaction is primarily ionic. Furthermore, all experimental and DFT-based An/Ln–M distances fall within the 3-sigma range of the lanthanide’s regression (Figure 2), making it more ambiguous to determine the covalent character in metal–amidinate bonding by this method.

When considering only the An^3+^ results, the slope of the linear regression deviates significantly from the criteria for ionic bonding (see Figure S29 in the Supporting Information). When only the two experimental data points are fitted, a slope of 0.53 is obtained. The interpretation of this slope is, of course, limited by the small number of data points. When the larger subset of DFT-derived bond distances is used instead, a yet smaller slope of 0.40(16) is obtained, which is clearly affected by the two points, Th and Pa, which clearly deviate from the linear trend. The intercepts of these regressions, which indicate the effective ionic radius of the ligand, also fall outside the 3-sigma range of the lanthanide regression (Ln^3+^: 1.31(1) Å vs An^3+^: 1.93 Å (SC-XRD), 2.05(17) Å (DFT)).

The best linear regression for the actinides is shown in (Figure 2) and includes all calculated bond distances except the two outliers, Th and Pa. Here, an *R*-factor of 0.9964 is obtained with a slope of 1.25(8) and an intercept of 1.20(8). The slope would indicate a slightly larger degree of covalency in the An–N bonds, supporting the intuitive interpretation of the shorter bond lengths. A detailed discussion of covalency will, nonetheless, have to be based on quantum-chemical calculations.

**Figure 3:**
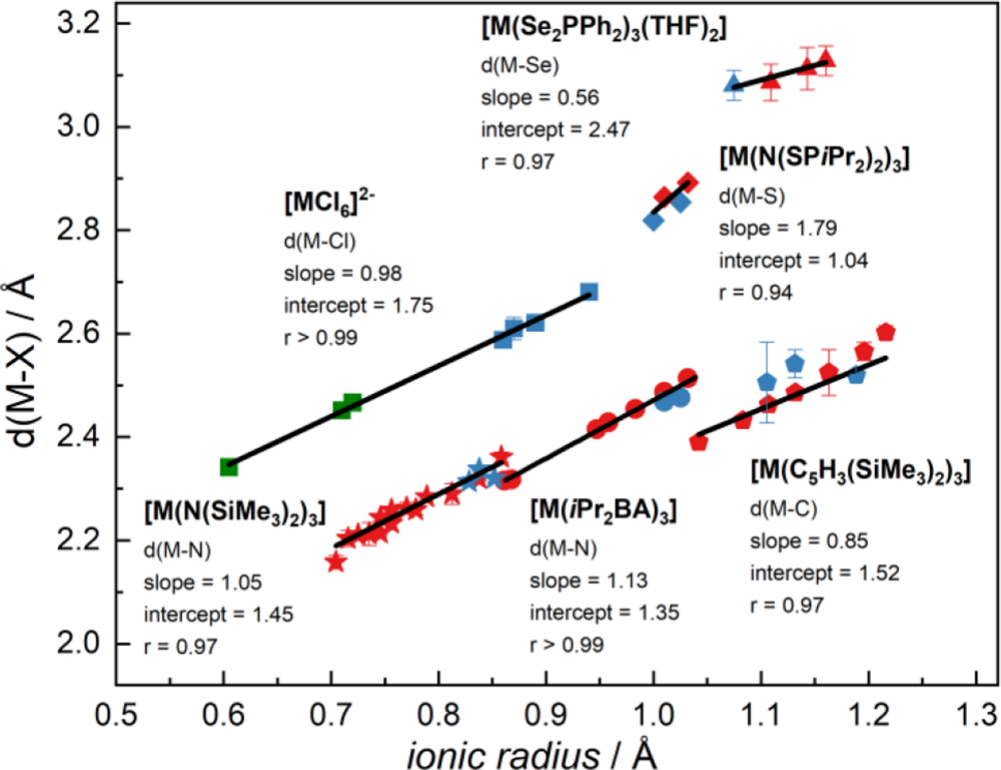
A plot of metal–ligand distances against ionic radius for various isostructural An/Ln complexes with different donor ligands: [M(*i*Pr_2_BA)_3_] (circle, CN 6), [MCl_6_]^2–^ (square, CN 6),^36^ [M(N(SP*i*Pr_2_)_2_)_3_] (diamond, CN 6),^37^ [M(Se_2_PPh_2_)_3_(THF)_2_] (triangle, CN 8),^38^ [M(N(SiMe_3_)_2_)_3_] (star, CN 3),^39−50^ and [M(C_5_H_3_(SiMe_3_)_2_)_3_] (pentagon, CN 9).^35,51−57^ Color codes: *d*-block metal, green; lanthanide, red; and actinide, blue. For ionic radius values not found in the reference,^30^ effective metal ionic radii were calculated according to Raymond and Eigenbrot's method^34^ based on coordination number, ensuring a consistent framework for systematic comparison.

Beyond the comparison of [Ln^III^(*i*Pr_2_BA)_3_] and [An^III^(*i*Pr_2_BA)_3_], it is interesting to compare the trends obtained here to similar series from the literature. To this end, various representative isostructural An and Ln complex series were compared, including metal chloride,^36^ metallocene,^35,51−57^ N-,^39−50^ S-,^37^ and Se-donor^38^ ligand complexes (Figure 3). The plot reveals that softer donor ligand complexes, like those with S and Se, deviate from a slope of 1 more significantly, indicating less ionic character. A consistent trend to larger or smaller slopes is not readily recognizable, i.e., [M(Se_2_PPh_2_)_3_(THF)_2_] exhibits a slope of 0.56, but for [M(N(SP*i*Pr_2_)_2_)_3_] a slope of 1.79 is obtained.

In contrast, the other complexes show slopes closer to 1 in the regression, indicating more ionic bonding. However, even in these cases, some actinide complexes, such as *i*Pr_2_BA and metallocene, show relatively larger deviations from the regression line compared to lanthanides. In such subtle cases, determining covalency through regression analysis alone will be challenging. Thus, while confirming the ionic bonding character of *f*-element amidinates, quantum-chemical calculations are necessary, as they provide in-depth bonding analysis, which may be more effective in identifying potentially weak covalent interactions.

#### 2.2.2 In Solution

Multinuclear NMR spectroscopy has verified that the structures of the complexes in their solid state largely remain intact when dissolved in a solution. By utilizing two-dimensional ^1^H–^13^C NMR spectroscopy, all signals were successfully assigned to their respective positions within the complex, as detailed in the Supporting Information. A single set of signals was detected for all three ligands across the experimental temperature range of 213–363 K. This observation hints toward a *D*_3_ symmetry in the complex molecule in solution, suggesting the three ligands maintain a consistent distance from the metal center over the NMR time scale.

All of the synthesized complexes **2**–**5**, **7**, and **8** exhibit paramagnetic behavior, whereas lanthanum **1** and lutetium complex **6** display a diamagnetic chemical shift due to their absence of unpaired valence *f*-electrons. Consequently, the lanthanum complex **1** was selected as a diamagnetic reference for further analysis of paramagnetic shifts. We used the same reference for the actinides due to the experimental challenges associated with an actinium complex.

In the variable temperature ^1^H NMR experiments conducted on paramagnetic complexes, only the signals corresponding to the methyl groups of the isopropyl units ((C***H***_3_)_2_CHN) displayed splitting at lower temperatures. This observation can be attributed to the characteristic position near the magic angle (∼54.7°), where slowed rotation leads to one side occupying a positive and the other a negative pseudocontact shift (PCS) area. A clear demonstration of this effect is seen in the Yb **5** complex (43.5 ppm vs. −9.7 ppm, Table 2), where two distinct signals were observed at ambient temperature (303 K). We attribute this observation of hindered rotation to the small ionic radius (0.868 Å) of Yb. A broadening in the ^1^H signal of the methyl signals in the diamagnetic Lu **6** complex and splitting into two carbon resonances in the ^13^C spectrum (27.6, 26.2 ppm) underlines this assumption. Conversely, for U **7** the large ionic radius of U^3+^ (1.025 Å) promotes free rotation of the isopropyl group, even at the low-temperature experimental limit of 213 K, and no splitting is observed (see Table S11 and Figure S34 in the Supporting Information). This observation also implies that for other protons located in the phenyl group, chemical exchanges and rotations occur at a rate too rapid to be detected within the time frame of NMR measurements.

**Table 2:**
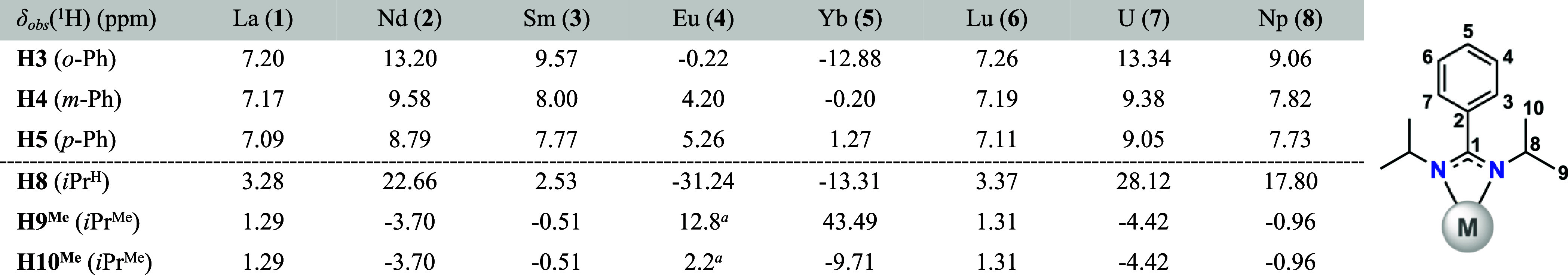
Labeling Chart for NMR Assignments and Observed ^1^H NMR Chemical Shifts for [M^III^(*i*Pr_2_BA)_3_] in Toluene-*d*_8_ (303 K)

a Values separated by deconvolution analysis due to very broad resonance at 303 K.

The methine protons of the isopropyl group ((CH_3_)_2_C***H***N), also positioned near the magic angle, were anticipated to exhibit minimal pseudocontact shifts. Contrary to this expectation, their resonance signals display a greatly pronounced paramagnetic shift, exemplified by a downfield shift of around 20 ppm for Nd **2** (Table 2). This indicates that the chemical shift of the methine proton is primarily influenced by the contact shift (FCS) contribution transmitted through the bond rather than the PCS propagated spatially. In the presence of heavier actinide ions, such as U **7** showing a shift of 25 ppm (Table 2), the effects are more pronounced, reflecting the additional contribution from relativistic spin–orbit (SO) coupling due to their greater mass. This is clearly a consequence of the proximity of the metal centers within the three chemical bonds. Similar shifts had been previously observed by the Arnold group for uranyl(VI) amidinate complexes^58^ and similar findings in guanidinate or amidinate methine protons.^17,59^

For the phenyl-group protons, when considering an amidinate complex molecule in solution exhibiting *D*_3_ symmetry, with the rotation axis aligned along the *z*-axis, the phenyl protons lie relatively horizontally close to the *xy*-plane. This positioning makes it an effective probe for exploring the PCS magnitude governed by the anisotropy of the magnetic susceptibility tensor values (Δχ_ax_, Δχ_rh_). In the ^1^H NMR resonance data presented in Table 2, the *o*-Ph (**H3**), *m*-Ph (**H4**), and *p*-Ph (**H5**) protons of the phenyl moiety for Nd **2**, Sm **3**, U **7**, and Np **8** exhibit chemical shifts that decrease as the distance from the metal ion increases, implying negative axial magnetic susceptibility tensor values (Δχ_ax_ < 0). Conversely, for Eu **4** and Yb **5**, the shifts increase, indicating positive tensor values (Δχ_ax_ > 0).^60,61^ More detailed discussions on this observation will be further explored in the paramagnetism study section.

#### 2.2.3 Structure Optimization and Quantum-Chemical Bonding Analysis

To correlate our experimental observations to the electronic and magnetic characteristics of the metal–ligand interactions, quantum-chemical calculations at the DFT (density functional theory) level of theory and wave function-based approaches were performed, starting with structure optimizations.

The basis for all structure optimizations was experimentally determined crystal structures, with no enforced symmetry in the calculations. The optimizations were carried out using TURBOMOLE,^62^ employing the Hybrid-XC-functional PBE0^63^ and def-SVP/TZVPP^64^ basis sets (for more details, see the SI). The agreement of the optimized structures with synthesized and spectroscopically characterized counterparts was evaluated by comparing the theoretical and experimental An/Ln–N bond lengths. Additionally, aligning the theoretical and experimental structures of the entire complex and specifically the inner coordinating atoms minimized the RMSD (root mean square deviation) of the atom pairs, underscoring the accuracy of our models (see Table S9 and Figure S31 in the Supporting Information). The deviation measured as relative error against the experimental bond length remained below 0.5% throughout the entire subset of compounds, which include the U **7** and Np **8** complexes for the actinides and Ce,^32^ Nd **2**, Sm **3**, Eu **4**, and Yb **5** as lanthanide representatives.

The accuracy of the calculations was further corroborated for Th(III) by comparing our computed values with the crystal structure bonding lengths of a Th(III) metallocene amidinate complex reported by Evans et al.^65^ Despite the varied steric effects presented by two pentamethylcyclopentadienyl (C_5_Me_5_) ligands and differences in amidinate functional groups, the reasonable proximity between the experimentally determined and calculated bond lengths (2.479(3) Å vs 2.487(1) Å) suggests that our computational approach is sufficiently accurate.

The complexes demonstrated comparably similar geometries, maintaining almost *D*_3_ symmetry. However, a noticeable distinction emerged between the lanthanides, which exhibited nearly identical structures (apart from the ligand-to-metal center distances), and the actinide complexes, where distortions and rotations, particularly of the outer phenyl rings and isopropyl groups, were evident (Figure S30). This difference is the first indication of differing bonding situations for the 4f and 5f elements, potentially caused by varying degrees of covalent contributions.

Utilizing the optimized structures of trivalent amidinate complexes, we explored bonding situations and observed trends with a particular focus on the concept of covalency. QTAIM (quantum theory of atoms in molecules) and NPA (natural population analysis) calculations were performed via AIMAll^66^ and NBO7,^67^ yielding delocalization index (DI) values of the metal–ligand bond as well as natural charges (*q*_NAO_) and occupancies of the NAO (natural atomic orbitals) of the central metal atoms, as presented in Table 3. For the NPA, DFT-based NAO was used. The trends and usability of these were validated by NAO based on CASSCF (complete active space self-consistent field) calculations (see Figure S45 in the Supporting Information).

**Table 3:** Summary of DFT, QTAIM Delocalization Indices (δ), NAO-Based Occupancies, and Natural Metal Charges for *f*-block Trivalent Amidinate Complexes (L = *i*Pr_2_BA); Quantities without Explicit Unit Are Given in a.u

		M^3+^	DFT	QTAIM	NAO		
		electron configuration	d(M–N) (Å)	δ_avg_(M–N)	Δ*d*	Δ*f*	*q*_NAO_(M)
[La(L)_3_]	(**1**)	[Xe]	2.533(1)	0.33	0.56 (5*d*)	0.00 (4*f*)	+2.24
[Ce(L)_3_]		[Xe] 4*f*^1^	2.486(0)	0.35	0.67 (5*d*)	0.13 (4*f*)	+2.01
[Pr(L)_3_]		[Xe] 4*f*^2^	2.466(4)	0.35	0.68 (5*d*)	0.15 (4*f*)	+1.99
[Nd(L)_3_]	(**2**)	[Xe] 4*f*^3^	2.452(3)	0.35	0.69 (5*d*)	0.11 (4*f*)	+2.02
[Pm(L)_3_]		[Xe] 4*f*^4^	2.436(5)	0.35	0.70 (5*d*)	0.10 (4*f*)	+2.03
[Sm(L)_3_]	(**3**)	[Xe] 4*f*^5^	2.420(9)	0.36	0.69 (5*d*)	0.11 (4*f*)	+2.01
[Eu(L)_3_]	(**4**)	[Xe] 4*f*^6^	2.412(5)	0.34	0.69 (5*d*)	0.09 (4*f*)	+2.03
[Yb(L)_3_]	(**5**)	[Xe] 4*f*^13^	2.323(2)	0.32	0.68 (5*d*)	0.02 (4*f*)	+2.02
[Lu(L)_3_]	(**6**)	[Xe] 4*f*^14^	2.314(1)	0.32	0.54 (5*d*)	0.00 (4*f*)	+2.12
[Ac(L)_3_]		[Rn]	2.601(8)	0.33	0.33 (6*d*)	0.27 (5*f*)	+2.13
[Th(L)_3_]		[Rn] 5*f*^1^	2.487(1)	0.43	1.29 (6*d*)	–0.53 (5*f*)	+1.62
[Pa(L)_3_]		[Rn] 5*f*^2^	2.454(2)	0.46	1.15 (6*d*)	–0.36 (5*f*)	+1.55
[U(L)_3_]	(**7**)	[Rn] 5*f*^3^	2.473(5)	0.42	0.66 (6*d*)	0.27 (5*f*)	+1.71
[Np(L)_3_]	(**8**)	[Rn] 5*f*^4^	2.462(1)	0.41	0.54 (6*d*)	0.29 (5*f*)	+1.82
[Pu(L)_3_]		[Rn] 5*f*^5^	2.446(8)	0.41	0.53 (6*d*)	0.25 (5*f*)	+1.90

Table 3 presents the metal–ligand bond lengths, the DIs, and natural charges (*q*_NAO_) along the first six lanthanides and actinides. Observing the bond lengths of the lanthanide complexes reveals a pattern of gradual decrease, aligning with the notion that Ln–ligand bonds are predominantly ionic, influenced mainly by the diminishing ionic radii of the lanthanides. This perspective gains support from the DI values, which maintain a small and nearly constant value of around 0.35. A similar consistency is seen in the NAO charges, which are nearly constant around +2.0 e. Among the lanthanides, La is the only exception with a slightly higher charge and lower DI, which can reasonably be attributed to the absence of 4*f* valence electrons.

The actinides show rather different and much more heterogeneous behavior (Table 3). The An–N bond lengths generally also decrease with decreasing ionic radii, but the drop is strongly enhanced for Th and Pa, after which the bond lengths actually increase going to U. The predicted shorter bond lengths coincide with higher DI values, 0.46 for Pa–N compared to 0.42 for U–N, and a minimum in the metal charges (+1.55 e for Pa). Jointly, these trends would suggest a strong increase in covalency for Pa(III) and Th(III).

Overall, this comparison of An and Ln compounds demonstrates how the characteristic 5*f* valence electrons of the actinides are more destabilized by relativistic effects than the 4*f* electrons of the lanthanides and hence more chemically active, resulting in a higher tendency to form covalent M–L bonds.^68^

**Figure 4:**
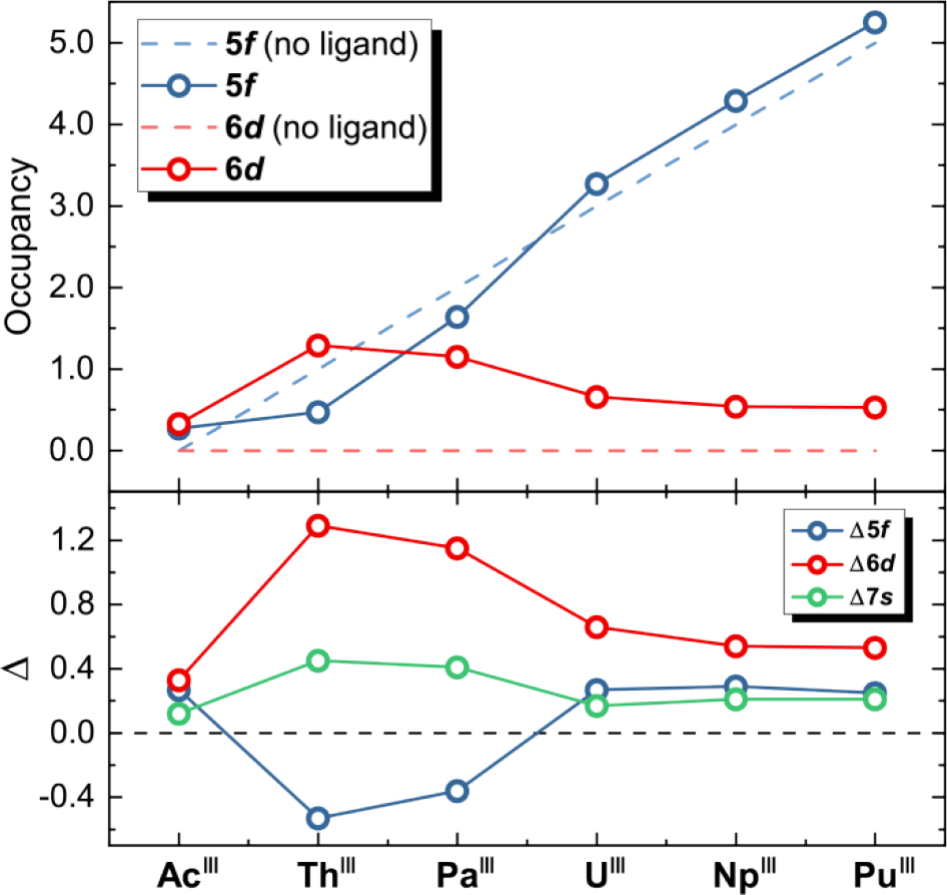
Occupancy of 5*f* and 6*d* orbitals before and after ligand coordination (top), along with the variations in occupancy (Δ) for the 5*f*, 6*d*, and 7*s* orbitals in comparison to the cation’s configuration before ligand attachment (bottom).

Using the distribution of electrons from the NPA, it is possible to correlate the covalency to the predominance of the metal’s valence orbitals. Since this section focuses on the exploration of the origins of covalency for the actinide species, the valence orbitals of interest are the 5*f*, 6*d*, and 7*s* orbitals. Figure 4 shows the change in the occupancy of these orbitals relative to the initial configuration before ligand coordination.

The initial electron configurations of the triply ionized actinides (Ac to Pu) are [Rn], [Rn] 5*f*^1^, [Rn] 5*f*^2^, ..., [Rn] 5*f*^5^ according to our calculations. It might seem surprising at first to have a 5*f*^1^ configuration for Th^3+^, while Th^0^ has 6*d*^2^7*s*^2^ occupation. The atomic configuration for the early actinides is defined by an energetically extremely close ordering of 5*f* and 6*d* orbitals and strongly destabilized 5*f* orbitals—both caused by relativistic effects that are stronger than those for their counterpart lanthanides—which explains the preference of 6*d* over 5*f*. With increasing nuclear or ion charge, however, the 5*f* orbitals become more stabilized, as exemplified by the differently charged Th cations: Th^+^: [Rn] 6*d*^2^7*s*^1^, Th^2+^: [Rn] 5*f*^1^6*d*^1^, and Th^3+^: [Rn] 5*f*^1^. For Th^3+^, this finally yields a 5*f*^1^ electron configuration.^69^ Nevertheless, this stabilization alone is still not sufficient to chemically stabilize Th^3+^ with its lone 5*f* electron, rendering the trivalent form extremely unstable and thus hypothetical for the compounds studied here. The respective ground state configurations for Pa from the neutral atom to the triply ionized form are Pa: [Rn] 5*f*^2^6*d*^1^7*s*^2^, Pa^+^: [Rn] 5*f*^2^7*s*^2^, Pa^2+^: [Rn] 5*f*^2^6*d*^1^, Pa^3+^: [Rn] 5*f*^2^.^68^

However, upon ligand coordination, Th and Pa exhibit notably high 6*d* orbital occupancy (Th: 1.29, Pa: 1.15), alongside a slight increase for the 7*s* electrons and a reduction in the 5*f* orbital occupancy. Via the electron transfer from the ligand through the coordinate covalent bond, the natural charges of Th and Pa are reduced to +1.62 and +1.55 from the original +3 state. Considering these natural oxidation states within the bonding environment of the complex, the electronic configurations of 5*f*^0.47^ 6*d*^1.29^ 7*s*^0.45^ (Th) and 7*s*^0.41^ 5*f*^1.64^ 6*d*^1.15^ (Pa) line up quite well with the configuration order of known oxidation states, and one can see why 6*d* configurations become more favored again. This apparent preference of the 6*d* orbitals for the formation of the An–N bond is furthermore associated with the diffuse nature of the 6*d* orbitals compared to 5*f*, rendering them more effective acceptor orbitals if the choice is allowed by symmetry.^68^ Starting from U the population of all partaking orbitals slowly converges toward the respective values of Pu with relatively low occupations (for Pu 5*f*: 0.25, 6*d*: 0.53, 7*s*: 0.21) matching the decreasing DI and increasing the charge for these An.

### 2.3 Paramagnetism

#### Separation of PCS and FCS *via* VT-pNMR Experiments

Paramagnetic NMR (pNMR) using lanthanides is an established method for the determination of molecular structures in solution.^70−74^ At the same time VT-NMR with actinides, especially TRU elements like Np, remains rare.^75,76^ The rapid relaxation of unpaired electrons causes paramagnetic shifts, with these electrons adapting swiftly to magnetic field changes, leading to enhanced nuclear shielding beyond that provided by electrons in closed shells.^77,78^ The key step for structure determination is the extraction of pseudocontact shifts (PCS) and their separation from bond-perpetrated contact shifts (FCS). The PCS are particularly informative due to their pronounced reliance on the polar coordinates of the observed nucleus, defined within a framework governed by the magnetic anisotropy tensor (Δχ_ax_, Δχ_rh_). To distinguish between the pseudocontact (PCS) and contact contributions (FCS) in Ln(III) complexes, several strategies have been developed,^74,77,79−81^ drawing upon Bleaney's theoretical framework.^82,83^

Building on the aforementioned discussion, two experimental methods were employed to separate these contributions. First, we utilized the separation technique based on the temperature dependence of FCS and PCS as outlined by Bleaney, which exploits the distinct temperature dependencies (respectively T^–1^ and T^–2^) of these shifts. Second, we applied the procedure suggested by Reilley,^78^ which hinges on the conservation of crystal field parameters across complexes of the same ligand. However, due to the absence of necessary literature values, this approach was applied exclusively to lanthanide complexes. The results are summarized in Table 4, and the detailed calculations and data from both methods are available in the Supporting Information.

**Table 4:** Separated PCS Values (δ_PCS_(^1^H)) by Using Bleaney’s *T*^–2^ Temperature Dependency Method (BL) and Reilley's method (RE) at 303 K

	Nd (**2**)		Sm (**3**)		Eu (**4**)		Yb (**5**)		U (**7**)	Np (**8**)
δ_PCS_(^1^H) (ppm)	BL	RE	BL	RE	BL	RE	BL	RE	BL	BL
**H3** (*o*-Ph)	2.14(10)	3.59(8)	–1.25(1)	0.599(13)	–3.06(22)	–3.42(7)	–4.30(19)	–18.8(4)	–1.48(2)	4.28(5)
**H4** (*m*-Ph)	0.64(4)	1.33(3)	–0.57(1)	0.222(5)	–1.25(9)	–1.27(3)	–1.83(9)	–6.97(15)	–0.58(1)	1.47(4)
**H5** (*p*-Ph)	0.54(4)	1.04(2)	–0.51(1)	0.174(4)	–1.17(7)	–0.99(2)	–1.54(7)	–5.45(12)	–0.58(1)	1.03(5)
**H8** (*i*Pr^H^)	3.89(19)	2.38(5)	2.77(8)	0.397(9)	6.78(16)	–2.27(5)	–3.07(21)	–12.5(3)	–2.97(12)	1.00(12)
Δχ_ax_^fit^ (10^–32^ m^3^)	–1.24	–2.15	+0.75	–0.36	+1.88	+2.05	+3.37	+11.3	+0.98	–2.74

To apply these methods, the consistency of geometry along the lanthanide series was first tested using the geometrical factor (*G*_i_) comparison (see Table S14 and Figure S41). While the phenyl group protons showed negligible differences, significant deviations were observed for the isopropyl methyl group due to rotation, leading to their exclusion from each analysis.

The most interesting difference in the results from both methods is observed in the isopropyl methine proton (H8). In the case of separation using the Bleaney method, a very large δ_PCS_ magnitude is exhibited (δ_PCS_(H8)/δ_PCS_(H5) > 5, with the exception of Yb), whereas the Reilley method results show a ratio more akin to the geometrical ratio (*G*_H8_/*G*_H5_ = 2.28), indicating that it may be more reliable. This significant discrepancy can be attributed to the substantial FCS and SO-induced shift in the methine proton region, as mentioned above. Relying solely on temperature dependency for separation can lead to inaccuracies due to this factor.^20,21^

The Bleaney method yields peculiar results for the Sm complex **3**. Here, the Reilley approach suggests a very small paramagnetic shift, in agreement with the minimal magnetic susceptibility tensor, reported in the literature.^70,72^ However, the Bleaney analysis yields a larger PCS with an opposite sign. This discrepancy is likely due to the smaller susceptibility tensor and considerable FCS contributions affecting the accuracy.

The accuracy of results for actinide complexes is notably compromised, which can be largely attributed to the SO contributions arising from the heavier actinide metals in addition to significant FCS contributions. Bleaney's method falls short in providing an accurate quantitative approximation, primarily because it restricts the temperature dependence of magnetic susceptibility to only the *T*^–2^ term,^21,22,60,84,85^ overlooking other influential factors. Furthermore, one of the foundational assumptions of this theory—that the ligand field splitting is significantly smaller than *kT* (210.6 cm^–1^ at 303 K)—is often not met in practice.^82,84,86^ Indeed, quantum-chemical calculations that consider orbital degeneracy, correlation among the *f* electrons, and SO coupling demonstrate that the ligand field splitting in the examined complexes are greater than *kT* in all cases (see Figure S44).

When PCS results for the overall lanthanide series derived from two separation methods were compared with the values obtained from quantum-chemical calculations, it was found that the results from the Reilley method demonstrated a closer correspondence to these values (Tables 4 and 5). Interestingly, for Yb **5**, the Reilley method predicted the results quite accurately, which is believed to be due to the minimized contact contribution (see Table S17). When applying the same CAS calculation approach to the actinide series, it was observed that appropriate PCS signs were shown for U **7**, and both correct signs and magnitudes were noted for Np **8**, indicating the effectiveness of this method in accurately predicting the behavior of these complexes. Further details on this computational method are addressed in the following section.

**Table 5:** δ_PCS_(^1^H) Values of the *i*Pr_2_BA Ligand within the Respective Ln^III^ and An^III^ Complexes at 303 K, Obtained from Kuprov’s equation *via* Spinach Based on Orca CAS χ-Tensors and Spin Densities

δ_PCS_(^1^H) (ppm)	Nd (**2**)	Sm (**3**)	Eu (**4**)	Yb (**5**)	Th	Pa	U (**7**)	Np (**8**)	Pu
**H3** (*o*-Ph)	4.68	2.56	–4.24	–17.3	–1.04	1.09	6.46	0.88	0.24
**H4** (*m*-Ph)	1.71	0.93	–1.57	–6.34	–0.38	0.53	2.37	0.33	0.09
**H5** (*p*-Ph)	1.33	0.73	–1.26	–4.96	–0.30	0.43	1.81	0.26	0.07
**H8** (*i*Pr^H^)	2.98	1.73	–2.90	–12.3	–0.71	1.01	4.02	0.61	0.16
Δχ_ax_ (10^–32^ m^3^)	–2.88	–1.55	+2.66	+10.3	+0.64	–0.82	–4.02	–0.56	–0.16

#### *Ab Initio* Calculation of Pseudocontact Shifts

To assess and compare the separation of paramagnetic chemical shift contributions obtained through experimental procedures, the magnetic properties of the respective actinide and lanthanide complexes were investigated computationally. In concrete terms, efforts were made to derive magnetic susceptibility tensors, as well as PCS values for all protons and three-dimensional representations of the PCS fields of the paramagnetic metal centers based on sophisticated CASSCF calculations. For the actinides, experimental PCS values for the U and Np complexes could not be utilized due to the lack of an appropriate diamagnetic reference and limitations, especially of the Bleaney method (see above). Since the distributed applied theoretical models are universal and the required susceptibility tensor and spin density could be provided by sufficiently accurate calculations, our results nonetheless allow a good and rare theoretical insight into the magnetic anisotropy of the early actinides.

For the purpose of exploring PCS, a relatively new approach was applied, which takes into account the distribution of spin electron density ρ and links it to the magnetic susceptibility χ in a partial differential equation, expanding the picture of the point dipole model used in Bleaney's approach. This relation is expressed in the Kuprov equation^87^:
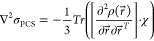
1

Deriving magnetic properties of lanthanide and actinide compounds from electronic structure calculations requires a highly sophisticated approach considering scalar relativistic and SO effects as well as static correlation present due to strongly correlated quasi-degenerate valence orbitals. A common method tackling the latter is the multideterminant CASSCF procedure. Starting orbitals, from which a subset of active orbitals was chosen, were provided either by DFT orbitals of the maximally ionized complex with energetically contracted orbital order or DFT quasi-restricted orbitals (QRO) with subsequent reordering by rotation by 90°. The identification of desired orbitals was done visually as well as on the basis of Löwdin atomic orbital contributions. For the lanthanide complexes, the 4*f* orbitals were chosen as active orbitals (CAS[*n*,7] with *n* being the number of unpaired *f* electrons). For the early actinides, however, in addition to the 5*f* orbitals the 6*d* orbitals must be considered, which are more energetically accessible than the 5d orbitals for the lanthanides, resulting from stronger relativistic effects present in the actinides.^68^ This situation is demonstrated quite well by the natural population of actinides in Table 3 and Figure 4. For this reason, the active space was extended to the 6*d* (and if locatable 7*s*) orbitals, arriving at CAS[*n*,12] and CAS[*n*,13] calculations. Since test calculations have shown a decreasing significance in 6*d* orbitals contribution starting from U and Np (see Figure S46 in the Supporting Information) and procedures with active spaces this large are extremely time-consuming, Pu was treated with the simplified approach using only the 5*f* orbitals.

Preparatory DFT calculations as well as the CASSCF procedure were performed using ORCA 5.0.4.^88^ To account for scalar-relativistic effects, the all-electron second-order DOUGLAS-KROLL-HESS (DKH)^89,90^ approach was applied, alongside recontracted basis sets (DKH-DEF2-TZVPP, An/Ln: SARC-DKH-TZVPP).^91,92^ Additionally, SO coupling was introduced through quasi-degenerate perturbation theory (QDPT).^93^

From the converged CAS calculations, spin densities and magnetic susceptibility tensors could be extracted, which were then processed within the MATLAB^94^ package Spinach,^95^ incorporating eq 1 and finally yielding PCS values and scalar fields. Representative isosurfaces (isovalue = ± 5 ppm) of these PCS fields are shown in Table 6. The schematic also lists axial and rhombic parts (Δχ_ax_ and Δχ_rh_) of the susceptibility tensor, which were derived in order to compare the values to the literature and to have direct values associated with the shape and symmetry of the PCS field. In case these quantities are derived from the traceless susceptibility tensor, Δχ_ax_ = 3 · χ_*zz*_/2 and Δχ_rh_ = (χ*_xx_–* χ_*yy*_)/2, where the absolute eigenvalues are ordered in Mehring order: |χ_*xx*_| < |χ_*yy*_| < |χ_*zz*_|.^61,84^ Under this definition, both axiality and rhombicity exhibit identical signs, with the theoretical upper boundary for the rhombicity to axiality ratio being 1/3, establishing the range (0 < χ_rh_/χ_ax_ < 1/3). Furthermore, the isotropic part of the susceptibility tensor χ_iso_ = 1/3 · Tr(χ) is denoted. The last quantity of the normalized or relative rhombic susceptibility Δχ_rh,rel_ was introduced to get a measure directly connected to the shape of the PCS field independent of the magnitude of the susceptibility (Δχ_rh,rel_ = |(χ*_xx_–* χ_*yy*_)/χ_*xx*_|). The raw susceptibility tensors and spheroid plots of the traceless tensors are given in the Supporting Information (Figure S47). The resulting PCS values for the distinct protons of the ligand are given in Table 5.

**Table 6:**
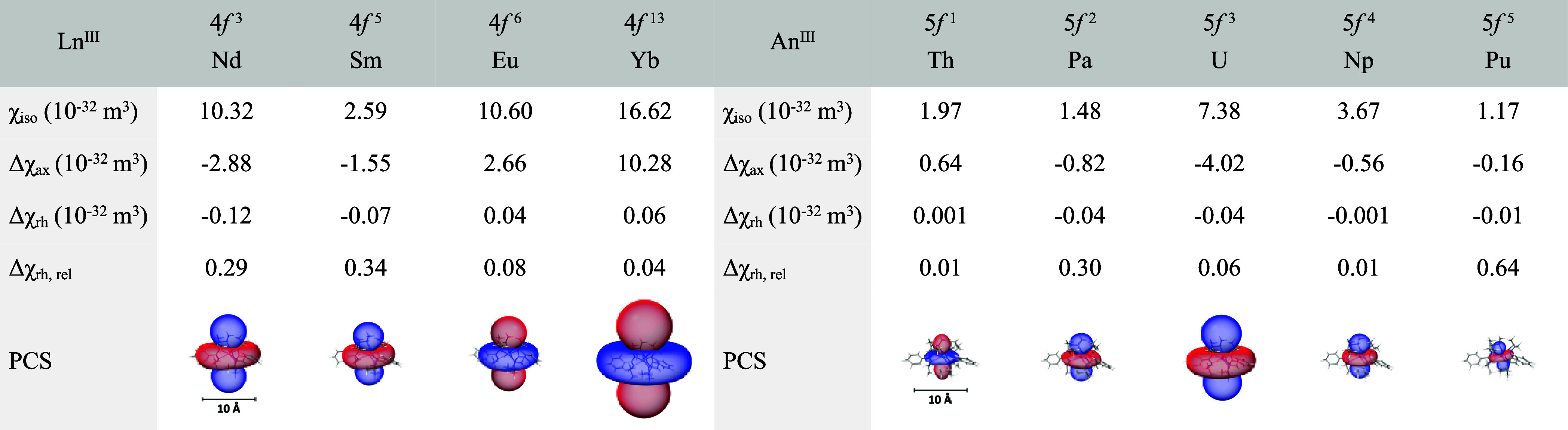
Isosurface Representations (± 5 ppm, Red = Positive, Blue = Negative) of Calculated PCS Fields for the Magnetically Investigated Ln^III^ and An^III^ complexes at 303 K with Associated Axial, Rhombic, and Isotropic Susceptibility Values in 10^–32^ m^3^

As can be seen in Table 6, all of the PCS cones have very small absolute rhombic susceptibility values and mainly exhibit a *d*_*z^2^*_-orbital shape, originating in the *D*_3_ symmetry of the complexes. However, using Δχ_rh,rel_, it can be shown that within the context of their overall magnitude some PCS fields, like those of Nd, Sm, Pa, and Pu, nevertheless tend to exhibit a distortion toward a *d*_*xz*_-orbital resemblance. This interplay of loss of symmetry and magnitude of the magnetic susceptibility can furthermore affect the orientation of the principal axis of the susceptibility tensor and thus the easy axis of magnetization, as can be seen in the strongly tilted case of the PCS cone of the Pu complex. In the case of Pu, which has the smallest ionic radius of the observed An series, the ligands close in more strongly on the central metal ion for bond formation, leading to steric hindrances between the ligand structures and deformations, finally lowering the symmetry of the coordination complex (reflected in the relatively high d(M–N) error (Table 3) and Δχ_rh,rel_ (Table 6) values). The resulting associated susceptibility spheroid, whose orientation directly correlates with the orientation of the PSC cone, has a pronounced tilt against the *C*_3_ axis compared with the other complexes. Nevertheless, the especially small χ_iso_ value smears the allocation of axes within the spheroidal representation, giving this apparent tilt a certain character of ambiguity.

A characteristic and expected behavior are the opposite signs of the PCS cones for Nd and Sm compared to Eu and Yb.^60,73,96^ Rather surprising is the case of Th. Whereas the analogous lanthanide Ce usually shows the same sign as the following elements, Th behaves inversely to its four heavier congeners.^60,73,96^

**Figure 5:**
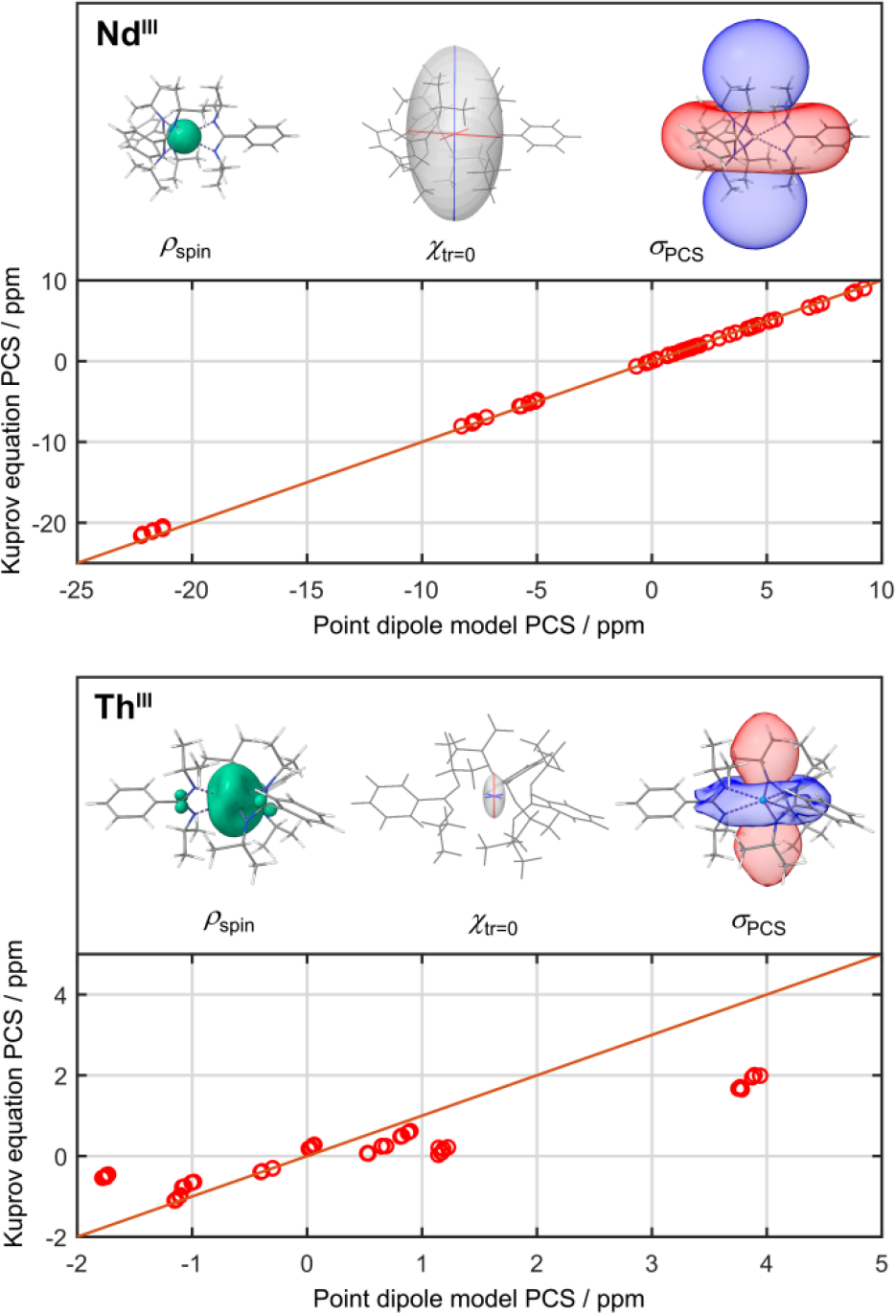
PCS values calculated with Kuprov's equation plotted *vs*. point dipole model PCS values for Nd(III) and Th(III) complexes, demonstrating the agreement of both methods dependent on the underlying spin density distribution. Both diagrams contain pictures of the complex structure with an isosurface plot of the spin density at 0.001 au, an ellipsoid representation of the traceless magnetic susceptibility tensor (red axes = positive eigenvalues, blue axes = negative eigenvalues), and an isosurface plot of the resulting PCS field at ±5 ppm (red = positive, blue = negative).

A striking difference is furthermore found between the early actinides Th and Pa and the lanthanides as well as the later actinides of the series. Compared to the other PCS fields, those from Th and Pa seem less homogeneous, with slight distortions and dips on the torus at the location of the binding ligand. Looking at the underlying spin density (Figure 5), it becomes clear that these disturbances stem from the bigger, more complex, and less spherical distribution of the spin density in these cases.

As was presented earlier, Th and Pa show distinctively high covalency for the An–ligand bond associated with a strong participation of 6*d* orbitals, leading to delocalization of the spin density across the ligands. These sources of perturbation of the central PCS field vanish as the covalency and spin density delocalization decrease toward Pu, as illustrated by the enhanced agreement observed in Figure S49 in the Supporting Information. This circumstance finally shows the importance of and necessity for an accurate construction of the PCS field in cases where the spin density deviates strongly from a small spherical distribution and the point dipole model would fail, especially for nuclei in close proximity to the paramagnetic center. The large discrepancy between the point dipole model PCS values and values obtained from the Kuprov equation for Th is demonstrated in Figure 5. The plot on the top shows that, for a typical lanthanide like Nd, the point dipole model is a sufficient approximation.

## 3 Conclusions

We have presented the synthesis and characterization of a series of isostructural *f*-element complexes, comprising six lanthanides and two actinides, with the *N*,*N*′-bis(isopropyl)benzamidinate (*i*Pr_2_BA) ligand. The Np(III) complex **8** is the first isolated transuranic complex in this family. All complexes crystallize in the same point group *C*2/*c*, with the exception of **8**, for which this structure is obtained only at higher temperatures (200 K). Consequently, the series was ideally suited for an in-depth investigation of trends along the 4*f* and 5*f* series, focusing on the properties of the chemical bonds to the metals and the compounds’ magnetic behavior.

The structural analysis in the solid state immediately reveals the major difference between the actinides and lanthanides. While the lanthanides exhibit the expected predominantly ionic behavior, the bond distances for both actinides were shortened (0.02–0.03 Å) relative to the values expected based on their ionic radii and the trend found for the lanthanides. This may indicate a nonionic contribution to the An–N bond, which was further supported by an analysis of the linear regression according to Raymond and Eigenbrot, which showed a larger deviation from a slope of 1.0 for the actinides. This was confirmed by quantum-chemical calculations, which reveal higher DIs and lower metal charges for the actinides relative to those of the lanthanides. Within the actinide series Th(III) and Pa(III) are predicted to exhibit the highest covalency, mainly driven by 6*d* orbital occupation.

Further experimental proof is found in the paramagnetic NMR studies, which clearly showed the shortcomings of established methods for PCS extraction when applied to the actinides. On the one hand, this is a direct effect of the stronger SO coupling in the actinides, which is neglected in for instance the Bleaney theory. On the other hand, shifts observed for the isopropyl methine protons in close proximity to the metal centers, i.e. only three bonds, show unusual shifts, which cannot be explained by SO effects alone. The explanation must then involve a large FCS contribution, which would be another indication of increased covalency in the An–N bond. Once again, our experimental findings are well supported by quantum-chemical calculations, which, moreover, illustrate a way forward for the accurate calculation of paramagnetic NMR shifts for the actinides with huge potential for future investigations of actinide structure and bonding.

In summary, we have reported a series of novel structures including a rare transuranic amidinate complex and characterized them on a molecular and electronic level with potential implications for, for example, thin layer deposition techniques and a fundamental understanding of actinide behavior in natural and technological systems.

## Experimental Section

**Caution!***Natural uranium (primary isotope*^*238*^*U) and neptunium consist of radioactive nuclides including long-lived* α*-emitters (*^*235*^*U; T*_*1/2*_*= 7.04 × 10*^*8*^*years,*^*238*^*U; T*_*1/2*_*= 4.47 × 10*^*9*^*years,*^*237*^*Np = 2.14 × 10*^*6*^*years). For safe handling, special precautions and equipment are necessary. Therefore, all of the experiments were conducted in a controlled laboratory equipped with appropriate detection equipment and safety protocols at the Institute of Resource Ecology, Helmholtz-Zentrum Dresden-Rossendorf.*

### General Considerations

All manipulations and reactions were carried out under careful exclusion of moisture and oxygen in nitrogen filled glove boxes or using Schlenk techniques. All solvents were predried over CaCl_2_ and distilled from Na/K alloy or potassium hydride and stored over activated 3 Å molecular sieves prior to use. The actinide starting materials UCl_4,_^97^ NpCl_4_(DME)_2_^98^ (DME = dimethoxyethane) as well as the ligand H*i*Pr_2_BA^99^ were prepared according to literature procedures. KH in mineral oil was suspended in *n*-pentane, filtered, washed several times with *n*-pentane and dried in the glovebox atmosphere. The anhydrous lanthanide starting materials and all other chemicals were used as received without further purification.

## Data Availability

The data underlying this study are openly available in RODARE (Rossendorf Data Repository) at DOI: 10.14278/rodare.3060

## Supporting Information Available

Additional experimental procedures, molecular structures, crystallographic data, NMR and IR spectroscopic data, paramagnetism studies, and details of quantum-chemical calculations (PDF)
