# Supplementary material for: Structure, Covalency, and Paramagnetism of Homoleptic Actinide and Lanthanide Amidinate Complexes
Source: Inorg Chem. 2024 Sep 2;63(38):17488–501. doi: 10.1021/acs.inorgchem.4c01901 (PMC11423402; doi:10.1021/acs.inorgchem.4c01901)
Supplement: Supplementary file 1 — ic4c01901_si_001.pdf [file ic4c01901_si_001.pdf]

Structure, covalency, and paramagnetism of homoleptic actinide and lanthanide amidinate complexes

Boseok Hong[1], Adrian Näder[1], Till Sawallisch[1], Tobias Bode[2], Sebastian Fichter[3], Robert Gericke[1], Peter Kaden[1], Michael Patzschke[1], Thorsten Stumpf[1], Moritz Schmidt*[,1], and Juliane März[1]

[1]Institute of Resource Ecology, Helmholtz-Zentrum Dresden-Rossendorf, Bautzner Landstraße 400, 01328 Dresden, Germany.

[2]Faculty of Chemistry and Food Chemistry, Technische Universität Dresden, Bergstraße 66c, 01069 Dresden, Germany.

[3]Institute of Ion Beam Physics and Materials Research, Helmholtz-Zentrum Dresden-Rossendorf, Bautzner Landstraße 400, 01328 Dresden, Germany.

*E-mail: moritz.schmidt@hzdr.de

Supporting Information

Table of Contents

1. General information S2
2. IR spectra S10
3. Molecular structures and crystallographic data S16
4. Quantum chemical calculations S31
5. Paramagnetic NMR analysis S50
6. Magnetism studies S65
7. NMR spectra S72
8. Literature S106

1. General information

1.1. Instrumentation and Methods

Single-Crystal X-ray Diffraction (SC-XRD)

Single crystals of compounds (**1-8**, **7-Cl**, **8-Cl**, H*i*Pr_2BA·NCMe, K-*i*Pr_2BA, [Lu(*i*Pr_2BA)$_2$Cl]$_2$) were placed in mineral oil, cut, mounted on a 100 μm MiTeGen Dual Thickness MicroMounts and located in the temperature-controlled N_2 gas flow at 100 or 200 K from Oxford instruments. The data was collected on a Bruker D8 VENTURE diffractometer equipped with a Photon II 7 array detector and micro focus Mo Kα radiation (λ = 0.71073 Å) with mirror optics monochromator. The computer programs SMART[1] and SAINT[2] were used for data collection in ϕ- and ω-scan modes and data processing, respectively. Absorption correction was performed using SADABS[3]. All structures were solved using ShelXT and all atoms were refined with full-matrix least-squares methods on F^2 using the SHELX-TL package[4,5]. ORTEP-3[6] was used for visualisation.

Nuclear Magnetic Resonance (NMR) Spectroscopy

NMR spectra were recorded on a Varian Inova 400 spectrometer with a ^{1}H frequency of 399.89 MHz and a ^{13}C frequency of 100.56 MHz. All spectra were recorded on a Varian AutoX ID probe head with z gradient. NMR experiments are performed using J Yong Teflon valve-sealed NMR tubes. Deuterated solvents were purchased at Deutero GmbH and dried over potassium metal and stored under 3 Å molecular sieve prior to use. Chemical shifts for ^{1}H and ^{13}C NMR are referenced and reported to internal reference tetramethylsilane (TMS, 0 ppm) or to the solvent used. Coupling constants (*J*) are reported in Hertz (Hz), and splitting patterns are indicated as s (singlet), d (doublet), t (triplet), q (quartet), sept (septet), m (multiplet) and brs (broad singlet). ^{1}H and ^{13}C NMR spectra were recorded directly and signals of ^{1}H and ^{13}C were correlated for all complexes using two-dimensional ^{1}H-^{1}H COSY, ^{1}H-^{13}C HSQC, and ^{1}H-^{13}C HMBC spectra.

Infrared (IR) Spectroscopy

IR spectra were measured on an Agilent Cary 630 FT-IR spectrometer equipped with a single reflection attenuated total reflection (ATR) accessory made of diamond. The measurements were performed in an inert N_2 atmosphere glovebox. The spectra were recorded between 4000 and 650 cm^{-1} with a resolution of 1 cm^{-1}.

Elemental Analysis

Elemental analysis for H, C, and N was performed on a vario MICRO cube (Elementar) with a helium gas flow. Np complexes were not measured due to radiosafety reasons.

Mass Spectroscopy

The mass spectrometric measurements were recorded either in positive or negative mode using an Expression L Compact Mass Spectrometers (Advion, Ithaca, USA) combined with atmospheric pressure chemical ionization (APCI). Nebulizer gas and dry gas were both N_2.

1.2. Procedures

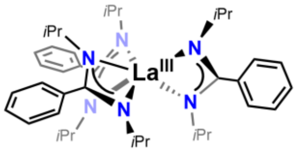

[La(iPr$_2$BA)$_3$] (**1**)

(Method A) 62.0 mg (0.100 mmol, 1.0 eq.) [La(N(SiMe$_3$)$_2$)$_3$] was dissolved in 1 mL THF. A solution of 65.4 mg (0.320 mmol, 3.2 eq.) HiPr$_2$BA in 1mL THF was added and the resulting solutions were stirred for 18 h at room temperature. The insoluble substances were separated by centrifugation. The supernatant was evaporated *in vacuo* to yield a pale-yellow residue. The residue was extracted with 1 mL of *n*-pentane and the volatiles were slowly evaporated to yield an off-white solid **1** [La(iPr$_2$BA)$_3$].

(Method B) a solution containing 61.3 mg (0.300 mmol, 3.0 eq.) HiPr$_2$BA and 13.2 mg (0.330 mmol, 3.3 eq.) KH in 2 mL THF was stirred for 30 min. A powder of 24.5 mg of anhydrous LaCl$_3$ (0.100 mmol, 1.0 eq.) was added slowly to this suspension and the combined pale-yellow suspension was stirred for 18 h at room temperature. The resulting suspension was centrifuged and the supernatant decanted. The solvents were removed *in vacuo* and the resulting pale-yellow residue was extracted with 2 mL of *n*-pentane. The volatiles were removed under reduced pressure to yield an off-white solid **1** [La(iPr$_2$BA)$_3$].

^{1}H NMR (400 MHz, toluene-d$_8$, 303 K, TMS) δ(ppm) = 7.22 – 7.16 (m, 12H, *o*- and *m*-Ph^H), 7.09 (d, ^3J = 5.4 Hz, 3H, *p*-Ph^H), 3.28 (sept, ^3J = 6.3 Hz, 6H, (CH$_3$)$_2$C***H***N), 1.29 (d, ^3J = 6.3 Hz, 36H, (C***H***$_3$)$_2$CHN).

^{13}C{^{1}H} NMR (100.58 MHz, toluene-d$_8$, 303 K, TMS) δ(ppm) = 175.9 (s, N***C***N), 136.6 (s, *i*-Ph^C), 128.5 (s, *m*-Ph^C), 127.7 (s, *p*-Ph^C), 127.0 (s, *o*-Ph^C), 48.6 (s, (CH$_3$)$_2$***C***HN), 27.2 (s, (***C***H$_3$)$_2$CHN).

IR (ATR): ν = 701 (s), 732 (m), 780 (m), 819 (w), 842 (w), 875 (w), 912 (m), 947 (w), 978 (w), 1005 (s), 1028 (w), 1072 (w), 1133 (m), 1165 (m), 1204 (m), 1272 (m), 1332 (s), 1357 (m), 1374 (m), 1439 (s), 1451 (s), 1577 (w), 1600 (w), 1635 (m), 2862 (w), 2920 (m), 2955 (m) cm^{-1}.

APCI-MS *m/z* (%): [*M*]$^+$ calcd for C$_{39}$H$_{57}$N$_6$La 748.4; Found: 749.2 (62) [*M*+H]$^+$, 545.4 (57) [*M*-*i*Pr$_2$BA]$^+$, 204.3 (100) [*i*Pr$_2$BA+H]$^+$.

Anal. calcd for C$_{39}$H$_{57}$N$_6$La (748.81 g/mol): C, 62.6; H, 7.7; N, 11.2; Found: C, 63.0; H, 7.1; N, 11.0. Despite repeated measurements on several synthesized samples, no significant improvement was obtained.

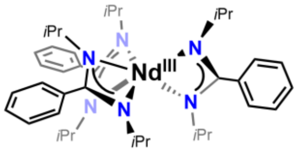

[Nd(iPr$_2$BA)$_3$] (**2**)

A solution containing 61.3 mg (0.300 mmol, 3.0 eq.) HiPr$_2$BA and 13.2 mg (0.330 mmol, 3.3 eq.) KH in 2 mL THF was stirred for 30 min. A powder of 25.1 mg of anhydrous NdCl$_3$ (0.100 mmol, 1.0 eq.) was added slowly to this suspension and the combined pale-yellow suspension was stirred for 24 h at room temperature. The resulting suspension was centrifuged, and the pale blue supernatant decanted. The solvents were removed *in vacuo* and the resulting pale blue residue was extracted with 2 mL of *n*-pentane. The volatiles were removed under reduced

pressure to yield a pale blue crystalline solid. Single crystals were obtained by slow evaporation of a saturated *n*-pentane solution of **2** [Nd(*i*Pr_2BA)$_3$] at ambient temperature.

^{1}H NMR (400 MHz, toluene-d$_8$, 303 K, TMS) δ(ppm) = 22.66 (s, 6H, $(CH_3)_2C\boldsymbol{H}N$), 13.20 (d, ^3J = 6.9 Hz, 6H, *o*-Ph^H), 9.58 (t, ^3J = 7.0 Hz, 6H, *m*-Ph^H), 8.79 (t, ^3J = 7.5 Hz, 3H, *p*-Ph^H), -3.70 (brs, 36H, $(C\boldsymbol{H}_3)_2CHN$).

^{13}C{^{1}H} NMR (100.58 MHz, toluene-d$_8$, 303 K, TMS) δ(ppm) = 136.3 (s, *i*-Ph^C), 133.6 (s, *o*-Ph^C), 130.6 (s, *m*-Ph^C), 131.3 (s, *p*-Ph^C), 60.8 (brs, $(CH_3)_2\boldsymbol{C}HN$), 21.6 (brs, $(\boldsymbol{C}H_3)_2CHN$), Signals for the N***C***N carbon could not be observed.

IR (ATR): ν = 672 (w), 701 (s), 732 (m), 779 (s), 819 (w), 844 (w), 875 (w), 912 (w), 947 (w), 1006 (s), 1028 (w), 1072 (w), 1134 (m), 1165 (m), 1206 (m), 1273 (w), 1333 (s), 1357 (s), 1374 (s), 1437 (s), 1577 (w), 1600 (w), 1636 (w), 2862 (w), 2893 (w), 2919 (w), 2955 (m) cm^{-1}.

APCI-MS *m/z* (%): [*M*]$^+$ calcd for $C_{39}H_{57}N_6Nd$ 751.4; Found: 752.0 (82) [*M*+H]$^+$, 548.4 (65) [*M*-*i*Pr_2BA]$^+$, 204.5 (100) [*i*Pr_2BA+H]$^+$.

Anal. calcd for $C_{39}H_{57}N_6Nd$ (754.14 g/mol): C, 62.1; H, 7.6; N, 11.1; Found: C, 62.5; H, 7.2; N, 11.0. Despite repeated measurements on several synthesized samples, no significant improvement was obtained.

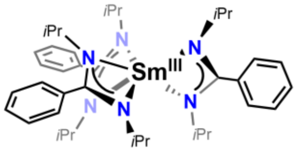

[Sm(*i*Pr_2BA)$_3$] (**3**)

A solution containing 61.3 mg (0.300 mmol, 3.0 eq.) H*i*Pr_2BA and 65.8 mg (0.330 mmol, 3.3 eq.) KHMDS in 1.0 mL THF was stirred for 30 min. A powder of 25.7 mg of anhydrous $SmCl_3$ (0.100 mmol, 1.0 eq.) was added slowly to this solution and the combined pale blue suspension was stirred for 18 h at room temperature. The resulting suspension was centrifuged, and the blue supernatant decanted. The solvents were removed *in vacuo* and the resulting dark blue residue was extracted with 2 mL of *n*-pentane. The volatiles were removed under reduced pressure to yield a blue solid. Single crystals were obtained by slow evaporation of a saturated *n*-pentane solution of **3** [Sm(*i*Pr_2BA)$_3$] at ambient temperature.

^{1}H NMR (400 MHz, toluene-d$_8$, 303 K, TMS) δ(ppm) = 9.57 (d, ^3J = 7.2 Hz, 6H, *o*-Ph^H), 8.00 (t, ^3J = 7.5 Hz, 6H, *m*-Ph^H), 7.77 (t, ^3J = 7.5 Hz, 3H, *p*-Ph^H), 2.53 (sept, ^3J = 6.1 Hz, 6H, $(CH_3)_2C\boldsymbol{H}N$), -0.51 (brs, 36H, $(C\boldsymbol{H}_3)_2CHN$).

^{13}C{^{1}H} NMR (100.58 MHz, toluene-d$_8$, 303 K, TMS) δ(ppm) = 141.8 (s, *i*-Ph^C), 130.0 (s, *m*-Ph^C), 129.2 (s, *o*-Ph^C), 128.7 (s, *p*-Ph^C), 46.6 (s, $(CH_3)_2\boldsymbol{C}HN$), 26.0 (brs, $(\boldsymbol{C}H_3)_2CHN$), Signals for the N***C***N carbon could not be observed.

IR (ATR): ν = 698 (s), 732 (m), 777 (m), 820 (w), 843 (w), 875 (w), 910 (w), 947 (w), 1005 (m), 1073 (m), 1134 (m), 1166 (m), 1209 (m), 1335 (s), 1358 (m), 1374 (m), 1436 (s), 1577 (w), 1637 (w), 1803 (w), 2610 (w), 2860 (m), 2889 (m), 2916 (m), 2954 (m) cm^{-1}.

APCI-MS *m/z* (%): [*M*]$^+$ calcd for $C_{39}H_{57}N_6Sm$ 761.4; Found: 762.5 (77) [*M*+H]$^+$, 558.3 (65) [*M*-*i*Pr_2BA]$^+$, 204.4 (100) [*i*Pr_2BA+H]$^+$.

Anal. calcd for $C_{39}H_{57}N_6Sm$ (760.27 g/mol): C, 61.6; H, 7.6; N, 11.1; Found: C, 60.8; H, 6.9; N, 11.0. Despite

repeated measurements on several synthesized samples, no significant improvement was obtained.

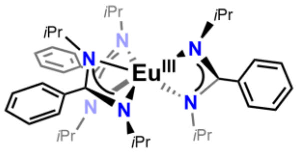

[Eu(*i*Pr$_2$BA)$_3$] (**4**)

A solution containing 61.3 mg (0.300 mmol, 3.0 eq.) H*i*Pr$_2$BA and 13.2 mg (0.330 mmol, 3.3 eq.) KH in 2 mL THF was stirred for 30 min. A powder of 25.8 mg of anhydrous $EuCl_3$ (0.100 mmol, 1.0 eq.) was added slowly to this suspension and the combined off-white suspension was stirred for 36 h at room temperature. The resulting suspension was centrifuged, and the red supernatant decanted. The solvents were removed *in vacuo* and the resulting orange residue was extracted with 2 mL of *n*-pentane. The volatiles were removed under reduced pressure to yield a red crystalline solid. Single crystals were obtained by slow evaporation of a saturated *n*-pentane solution of **4** [Eu(*i*Pr$_2$BA)$_3$] at ambient temperature.

^{1}H NMR (400 MHz, toluene-d$_8$, 303 K, TMS) δ(ppm) = 5.26 (t, ^3J = 7.7 Hz, 3H, *p*-Ph^H), 4.20 (t, ^3J = 7.6 Hz, 6H, *m*-Ph^H), -0.22 (d, ^3J = 7.6 Hz, 6H, *o*-Ph^H), -31.24 (s, 6H, (CH$_3$)$_2$C***H***N), Signals for the (C***H***$_3$)$_2$CHN protons appears as a very broad signal ranging from 28 to -14 ppm at 303 K.

^{1}H NMR (400 MHz, toluene-d$_8$, 273 K, TMS) δ(ppm) = 19.82 (s, 18H, (C***H***$_3$)$_2$CHN), 4.88 (t, ^3J = 7.4 Hz, 3H, *p*-Ph^H), 3.69 (t, ^3J = 7.4 Hz, 6H, *m*-Ph^H), -1.50 (d, ^3J = 7.7 Hz, 6H, *o*-Ph^H), -3.52 (s, 18H, (C***H***$_3$)$_2$CHN)**,** -34.28 (s, 6H, (CH$_3$)$_2$C***H***N).

^{13}C{^{1}H} NMR (100.58 MHz, toluene-d$_8$, 303 K, TMS) δ(ppm) = 132.1 (s, *i*-Ph^C), 127.1 (s, *m*-Ph^C), 122.2 (s, *p*-Ph^C), 116.9 (s, *o*-Ph^C), 31.3 (s, (CH$_3$)$_2$***C***HN), Signals for the (***C***H$_3$)$_2$CHN, N***C***N carbons could not be observed.

IR (ATR): ν = 672 (w), 701 (vs), 733 (m), 779 (s), 820 (w), 844 (w), 875 (w), 912 (m), 949 (w), 1007 (s), 1028 (w), 1072 (m), 1135 (s), 1166 (m), 1209 (m), 1273 (m), 1335 (s), 1358 (s), 1374 (s), 1437 (vs), 1577 (w), 1600 (w), 1636 (m), 2863 (m), 2891 (m), 2920 (m), 2956 (s) cm^{-1}.

APCI-MS *m/z* (%): [*M*]$^+$ calcd for $C_{39}H_{57}N_6Eu$ 762.4; Found: 763.5 (92) [*M*+H]$^+$, 559.3 (90) [*M*-*i*Pr$_2$BA]$^+$, 204.3 (100) [*i*Pr$_2$BA+H]$^+$; 761.9 (95) [*M*-H]$^-$, 203.1 (100) [*i*Pr$_2$BA]$^-$.

Anal. calcd for $C_{39}H_{57}N_6Eu$ (761.87 g/mol): C, 61.5; H, 7.5; N, 11.0; Found: C, 65.2; H, 7.5; N, 11.6. Despite repeated measurements on several synthesized samples, no significant improvement was obtained.

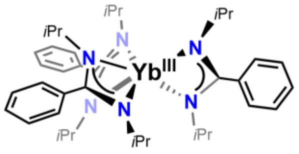

[Yb(*i*Pr$_2$BA)$_3$] (**5**)

A solution containing 61.3 mg (0.300 mmol, 3.0 eq.) H*i*Pr$_2$BA and 13.2 mg (0.330 mmol, 3.3 eq.) KH in 2 mL THF was stirred for 30 min. A powder of 27.9 mg of anhydrous $YbCl_3$ (0.100 mmol, 1.0 eq.) was added slowly to this suspension and the combined pale-yellow suspension was stirred for 24 h at room temperature. The resulting

suspension was centrifuged, and the yellow supernatant decanted. The solvents were removed *in vacuo* and the resulting off-white residue was extracted with 2 mL of *n*-pentane. The volatiles were removed under reduced pressure to yield a pale yellow crystalline solid. Single crystals were obtained by slow evaporation of a saturated *n*-pentane solution of **5** [Yb(*i*Pr_2BA)$_3$] at ambient temperature.

^{1}H NMR (400 MHz, toluene-d$_8$, 303 K, TMS) δ(ppm) = 43.49 (s, 18H, (C***H***$_3$)$_2$CHN), 1.27 (t, ^3J = 7.7 Hz, 3H, *p*-Ph^H), -0.20 (t, ^3J = 7.6 Hz, 6H, *m*-Ph^H), -9.71 (s, 18H, (C***H***$_3$)$_2$CHN), -12.88 (d, ^3J = 8.2 Hz, 6H, *o*-Ph^H), -13.31 (s, 6H, (CH$_3$)$_2$C***H***N).

^{13}C{^{1}H} NMR (100.58 MHz, toluene-d$_8$, 303 K, TMS) δ(ppm) = 118.9 (s, *p*-Ph^C), 118.1 (s, *m*-Ph^C), 106.9 (brs, *o*-Ph^C), 97.5 (s, *i*-Ph^C), 67.2 (brs, (***C***H$_3$)$_2$CHN), 66.3 (s, N***C***N), 40.0 (brs, (CH$_3$)$_2$***C***HN), 19.3 (brs, (***C***H$_3$)$_2$CHN).

IR (ATR): ν = 697 (vs), 719 (m), 734 (m), 777 (s), 823 (w), 844 (w), 877 (w), 910 (m), 950 (w), 1008 (s), 1073 (m), 1123 (s), 1137 (s), 1167 (m), 1216 (s), 1275 (w), 1339 (vs), 1358 (s), 1374 (s), 1435 (vs), 1453 (vs), 1578 (w), 1600 (w), 1635 (w), 2612 (w), 2860 (m), 2894 (m), 2918 (m), 2957 (s) cm^{-1}.

APCI-MS *m/z* (%): [*M*]$^+$ calcd for $C_{39}H_{57}N_6Yb$ 783.4; Found: 784.2 (89) [*M*+H]$^+$, 580.7 (67) [*M*-*i*Pr_2BA]$^+$, 204.5 (100) [*i*Pr_2BA+H]$^+$.

Anal. calcd for $C_{39}H_{57}N_6Yb$ (782.95 g/mol): C, 59.8; H, 7.3; N, 10.7; Found: C, 61.1; H, 7.2; N, 9.9. Despite repeated measurements on several synthesized samples, no significant improvement was obtained.

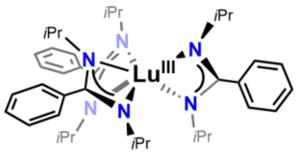

[Lu(*i*Pr_2BA)$_3$] (**6**)

A solution containing 61.3 mg (0.300 mmol, 3.0 eq.) H*i*Pr_2BA and 13.2 mg (0.330 mmol, 3.3 eq.) KH in 2 mL THF was stirred for 30 min. A powder of 28.1 mg of anhydrous $LuCl_3$ (0.100 mmol, 1.0 eq.) was added slowly to this suspension and the combined pale-yellow suspension was stirred for 20 h at room temperature. The resulting suspension was centrifuged, and the supernatant decanted. The solvents were removed *in vacuo* and the resulting pale-yellow residue was extracted with 2 mL of *n*-pentane. The volatiles were removed under reduced pressure to yield a pale yellow crystalline solid. Single crystals were obtained by slow evaporation of a saturated *n*-pentane solution of **6** [Lu(*i*Pr_2BA)$_3$] at ambient temperature.

^{1}H NMR (400 MHz, toluene-d$_8$, 303 K, TMS) δ(ppm) = 7.27 – 7.17 (m, 12H, *o*- and *m*-Ph^H), 7.10 (d, ^3J = 6.4 Hz, 3H, *p*-Ph^H), 3.37 (sept, ^3J = 6.2 Hz, 6H, (CH$_3$)$_2$C***H***N), 1.31 (dd, *J* = 12.5, 6.5 Hz, 36H, (C***H***$_3$)$_2$CHN).

^{13}C{^{1}H} NMR (100.58 MHz, toluene-d$_8$, 303 K, TMS) δ(ppm) = 176.9 (s, N***C***N), 136.6 (s, *i*-Ph^C), 128.6 (s, *m*-Ph^C), 128.0 (s, *p*-Ph^C), 126.7 (s, *o*-Ph^C), 48.5 (s, (CH$_3$)$_2$***C***HN), 26.9 (d, *J* = 134.1 Hz, (***C***H$_3$)$_2$CHN).

IR (ATR): ν = 699 (s), 719 (w), 734 (m), 778 (s), 823 (w), 844 (w), 877 (w), 910 (w), 950 (w), 1010 (s), 1073 (w), 1123 (m), 1138 (m), 1167 (m), 1218 (m), 1340 (vs), 1358 (m), 1374 (m), 1437 (vs), 1454 (vs), 1577 (w), 1630 (w), 2614 (w), 2861 (w), 2895 (m), 2920 (m), 2957 (m) cm^{-1}.

APCI-MS *m/z* (%): [*M*]$^+$ calcd for $C_{39}H_{57}N_6Lu$ 784.4; Found: 785.5 (72) [*M*+H]$^+$, 581.4 (32) [*M*-*i*Pr_2BA]$^+$, 204.4 (100) [*i*Pr_2BA+H]$^+$.

Anal. calcd for $C_{39}H_{57}N_6Lu$ (784.88 g/mol): C, 59.7; H, 7.3; N, 10.7; Found: C, 59.1; H, 6.6; N, 10.6. Despite repeated measurements on several synthesized samples, no significant improvement was obtained.

Due to the steric effects caused by the small ionic radius of lutetium, an insufficient reaction time led to the formation of a bis-amidinate lutetium dimer complex [Lu(*i*Pr$_2$BA)$_2$Cl]$_2$ instead of a tris-amidinate complex. The compound was subsequently isolated, and its crystal structure was characterized with SC-XRD.

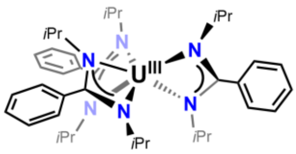

[U(*i*Pr$_2$BA)$_3$] (**7**)

18.6 mg (0.02 mmol, 1.0 eq.) [UCl(*i*Pr$_2$BA)$_3$]·0.5C_7H_8 (**7-Cl**) was dissolved in 2 mL toluene and added to 10.8 mg KC_8 (0.08 mmol, 4.0 eq.) resulting in a color change from green to dark purple. The suspension was vigorously stirred for 48 h at ambient temperature. The resulting suspension was centrifuged, and the supernatant was filtered through 0.45 µm PTFE syringe filter. The solvent was removed *in vacuo* yielding dark blue residue. The residue was extracted into 1 mL *n*-pentane and dried under reduced pressure to yield a dark blue solid. Single crystals were obtained by slow evaporation of a saturated *n*-pentane solution of **7** [U(*i*Pr$_2$BA)$_3$] at ambient temperature.

^{1}H NMR (400 MHz, toluene-d$_8$, 303 K, TMS) δ(ppm) = 28.12 (s, 6H, $(CH_3)_2C\boldsymbol{H}N$), 13.34 (d, ^{3}J = 6.9 Hz, 6H, *o*-Ph^{H}), 9.38 (t, ^{3}J = 7.0 Hz, 6H, *m*-Ph^{H}), 9.05 (t, ^{3}J = 7.5 Hz, 3H, *p*-Ph^{H}), -4.42 (brs, 36H, $(C\boldsymbol{H}_3)_2CHN$).

^{13}C{^{1}H} NMR (100.58 MHz, toluene-d$_8$, 303 K, TMS) δ(ppm) = 128.4 (s, *p*-Ph^{C}), 126.9 (s, *o*-Ph^{C}), 125.0 (s, *m*-Ph^{C}), 15.3 (s, *i*-Ph^{C}), Signals for the N***C***N, $(\boldsymbol{C}H_3)_2CHN$, $(CH_3)_2\boldsymbol{C}HN$ carbons could not be observed.

IR (ATR): ν = 702 (s), 733 (w), 779 (m), 802 (m), 862 (w), 913 (w), 935 (w), 945 (w), 967 (w), 1005 (s), 1073 (m), 1095 (w), 1120 (w), 1134 (w), 1166 (w), 1204 (m), 1260 (m), 1334 (s), 1358 (m), 1374 (m), 1436 (s), 1577 (w), 1601 (w), 1637 (w), 2862 (w), 2922 (w), 2956 (m) cm^{-1}.

APCI-MS *m/z* (%): $[M]^+$ calcd for $C_{39}H_{57}N_6U$ 847.5; Found: 848.2 (88) $[M+H]^+$, 644.5 (63) [*M*-*i*Pr$_2$BA]$^+$, 204.3 (100) [*i*Pr$_2$BA+H]$^+$.

Anal. calcd for $C_{39}H_{57}N_6U$ (847.94 g/mol): C, 55.2; H, 6.8; N, 9.9; Found: C, 58.5; H, 7.7; N, 9.2. Despite repeated measurements on several synthesized samples, no significant improvement was obtained.

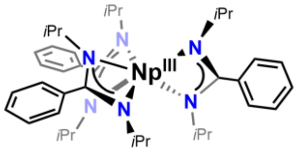

[Np(*i*Pr$_2$BA)$_3$] (**8**)

9.3 mg (0.01 mmol, 1.0 eq.) [NpCl(*i*Pr$_2$BA)$_3$]·0.5C_7H_8 (**8-Cl**) was dissolved in 1.0 mL THF and added to 5.4 mg KC_8 (0.04 mmol, 4.0 eq.) resulting in a color change from crimson to dark purple. The suspension was vigorously stirred for 18 h at ambient temperature. The resulting suspension was centrifuged, and the supernatant was filtered through 0.45 µm PTFE syringe filter. The solvent was removed *in vacuo* and the resulting dark purple residue was

extracted with 1 mL of *n*-pentane. The volatiles were removed under reduced pressure to yield **8** [Np(*i*Pr_2BA)$_3$] as a dark purple solid.

^{1}H NMR (400 MHz, toluene-d$_8$, 303 K, TMS) δ(ppm) = 17.81 (sept, ^3J = 6.3 Hz, 6H, $(CH_3)_2C\boldsymbol{H}N$), 9.06 (d, ^3J = 6.4 Hz, 6H, *o*-Ph^H), 7.81 (t, ^3J = 7.3 Hz, 6H, *m*-Ph^H), 7.72 (t, ^3J = 7.6 Hz, 3H, *p*-Ph^H), -0.96 (brs, 36H, $(C\boldsymbol{H}_3)_2CHN$).

^{13}C{^{1}H} NMR (100.58 MHz, toluene-d$_8$, 303 K, TMS) δ(ppm) = 128.7 (s, *p*-Ph^C), 125.5 (s, *m*-Ph^C), 120.7 (s, *o*-Ph^C), 72.8 (s, *i*-Ph^C), 12.6 (s, $(CH_3)_2\boldsymbol{C}HN$), 8.9 (s, $(\boldsymbol{C}H_3)_2CHN$), Signals for the N***C***N carbon could not be observed.

IR (ATR): ν = 671 (w), 702 (m), 733 (w), 780 (m), 820 (w), 873 (w), 913 (w), 946 (w), 992 (w), 1006 (m), 1027 (w), 1073 (w), 1122 (w), 1134 (w), 1165 (w), 1206 (w), 1334 (s), 1358 (m), 1374 (m), 1458 (s), 1576 (w), 1599 (w), 1622 (w), 2605 (w), 2860 (m), 2893 (w), 2921 (m), 2954 (s) cm^{-1}.

APCI-MS *m/z* (%): [*M*]$^+$ calcd for $C_{39}H_{57}N_6Np$ 846.5; Found: 847.0 (52) [*M*+H]$^+$, 643.6 (65) [*M*-*i*Pr_2BA]$^+$, 204.5 (100) [*i*Pr_2BA+H]$^+$.

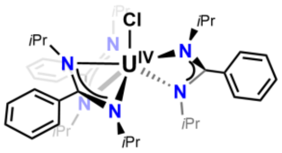

[UCl(*i*Pr_2BA)$_3$]·0.5C_7H_8 (**7-Cl**)

61.3 mg (0.30 mmol, 3.0 eq.) H*i*Pr_2BA and 65.8 mg (0.33 mmol, 3.3 eq.) KHMDS were dissolved in 1 mL THF and stirred for 15 min. Upon adding a 1 mL THF solution of 38.0 mg (0.1 mmol, 1.0 eq.) UCl_4 the color of solution changes to a dark brown. The solution was stirred for 20 h and the solvent was removed *in vacuo*. The dark brown residue was extracted into 5 mL toluene to yield a brownish green solution. The solution was filtered and evaporated under reduced pressure. The resulting residue was washed and triturated with *n*-pentane to yield a green powder. Single crystals were obtained by slow evaporation of a saturated toluene solution of **7-Cl** [UCl(*i*Pr_2BA)$_3$]·0.5C_7H_8 at ambient temperature.

^{1}H NMR (400 MHz, toluene-d$_8$, 368 K, TMS) δ(ppm) = 18.27 (brs, 6H, $(CH_3)_2C\boldsymbol{H}N$), 8.92 (d, ^3J = 7.2 Hz, 6H, *o*-Ph^H), 7.65 (t, ^3J = 7.4 Hz, 6H, *m*-Ph^H), 7.58 (t, ^3J = 7.4 Hz, 3H, *p*-Ph^H), 0.52 (d, ^3J = 6.0 Hz, 36H, $(C\boldsymbol{H}_3)_2CHN$).

^{13}C{^{1}H} NMR (100.58 MHz, toluene-d$_8$, 368 K, referenced to $C_5D_5\boldsymbol{C}D_3$ at 20.4 ppm as the TMS signal could not be observed) δ(ppm) = 129.3 (s, *p*-Ph^C), 126.8 (s, *m*-Ph^C), 117.0 (s, *o*-Ph^C), 114.6 (s, *i*-Ph^C), 33.6 (s, $(CH_3)_2\boldsymbol{C}HN$), 28.3 (s, $(\boldsymbol{C}H_3)_2CHN$). Signal for the N***C***N carbons could not be observed.

IR (ATR): ν = 707 (s), 728 (m), 736 (m), 780 (m), 917 (w), 1009 (m), 1077 (w), 1125 (m), 1137 (m), 1171 (m), 1203 (m), 1335 (m), 1376 (m), 1419 (m), 1445 (m), 1459 (w), 2960 (w) cm^{-1}.

APCI-MS *m/z* (%): [*M*]$^+$ calcd for $C_{39}H_{57}N_6ClU$ 882.5; Found: 883.7 (42) [*M*+H]$^+$, 679.6 (65) [*M*-*i*Pr_2BA]$^+$, 204.4 (100) [*i*Pr_2BA+H]$^+$.

Anal. calcd for $C_{42.5}H_{61}N_6ClU$ (929.45 g/mol): C, 54.9; H, 6.6; N, 9.0; Found: C, 54.3; H, 6.6; N, 9.0.

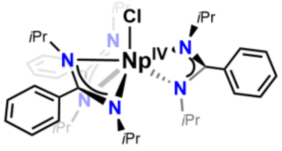

[NpCl(*i*Pr$_2$BA)$_3$]·0.5C$_7$H$_8$ (**8-Cl**)

11.2 mg (0.02 mmol, 1.0 eq.) [NpCl$_4$(DME)$_2$] was dissolved in 1 mL THF to yield a pale orange solution. A solution of 14.5 mg (0.06 mmol, 3.0 eq.) [K-*i*Pr$_2$BA] in 0.5 mL THF was slowly added resulting in a color change to dark red. The solution was stirred for 18 h and the solvent was removed *in vacuo*. The dark red residue was extracted into 1 mL toluene and separated from insoluble substances by centrifugation. The supernatant was evaporated under reduced pressure. The resulting dark red residue was washed and triturated with *n*-pentane to yield a red powder. Single crystals were obtained by diffusion of *n*-pentane into a saturated solution of **8-Cl** [NpCl(*i*Pr$_2$BA)$_3$]·0.5C$_7$H$_8$ at ambient temperature.

^{1}H NMR (400 MHz, toluene-d$_8$, 363 K, TMS) δ(ppm) = 26.52 (brs, 6H, (CH$_3$)$_2$C***H***N), 11.57 (s, 6H, *o*-Ph^H), 8.92 (t, ^3J = 7.2 Hz, 6H, *m*-Ph^H), 8.48 (t, ^3J = 7.5 Hz, 3H, *p*-Ph^H), -0.79 (brs, 36H, (C***H***$_3$)$_2$CHN).

^{13}C{^{1}H} NMR (100.58 MHz, toluene-d$_8$, 363 K, referenced to C$_5$D$_5$***C***D$_3$ at 20.4 ppm as the TMS signal could not be observed) δ(ppm) = 131.3 (s, *p*-Ph^C), 127.4 (s, *m*-Ph^C), 126.1 (s, *o*-Ph^C), 78.1 (s, *i*-Ph^C), 25.3 (s, (***C***H$_3$)$_2$CHN). Signals for the N***C***N, (CH$_3$)$_2$***C***HN carbons could not be observed.

IR (ATR): ν = 706 (s), 728 (m), 737 (m), 780 (m), 1007 (m), 1076 (w), 1125 (m), 1138 (m), 1171 (m), 1204 (m), 1336 (s), 1376 (m), 1422 (s), 1445 (m), 1459 (w), 2960 (w) cm^{-1}.

APCI-MS *m/z* (%): [*M*]$^+$ calcd for C$_{39}$H$_{57}$N$_6$ClNp 881.4; Found: 882.5 (57) [*M*+H]$^+$, 678.5 (71) [*M*-*i*Pr$_2$BA]$^+$, 204.1 (100) [*i*Pr$_2$BA+H]$^+$.

2. IR spectra

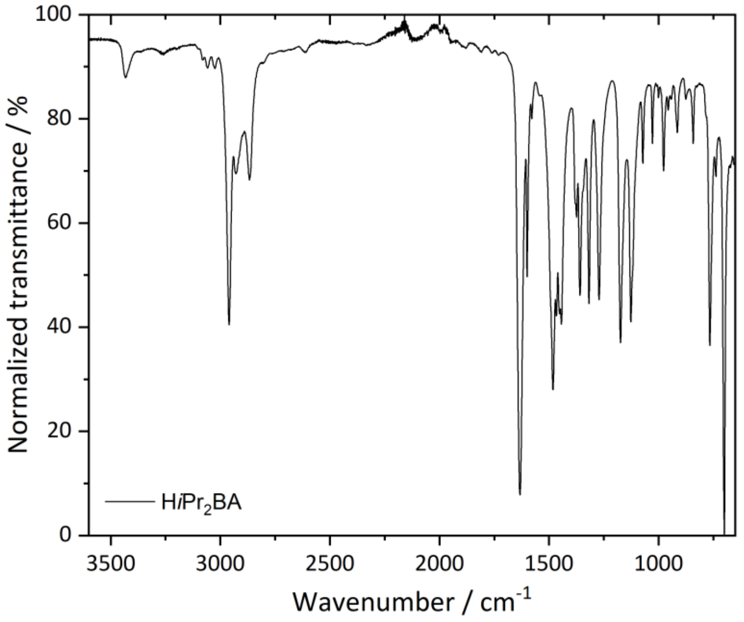

Figure S1. Normalized ATR-IR spectrum of H*i*Pr_2BA ligand

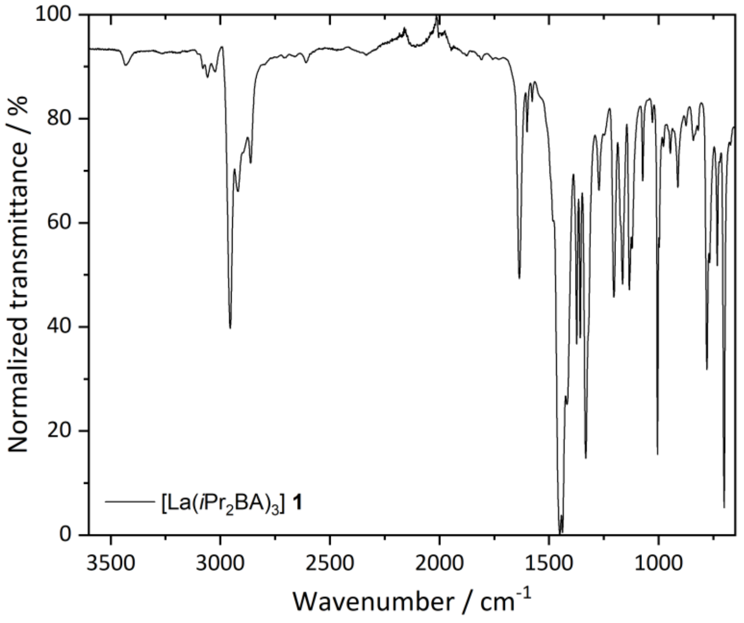

Figure S2. Normalized ATR-IR spectrum of [La(*i*Pr_2BA)$_3$] **1** complex

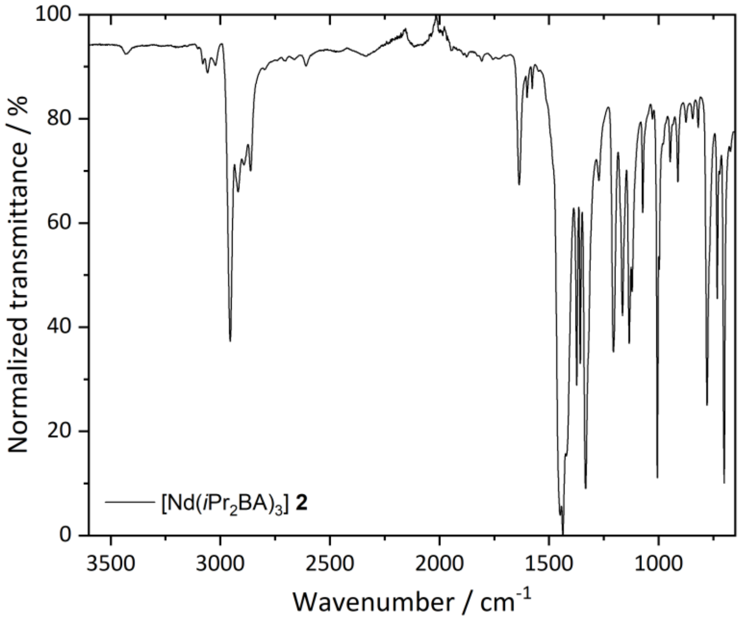

Figure S3. Normalized ATR-IR spectrum of [Nd(*i*Pr$_2$BA)$_3$] **2** complex

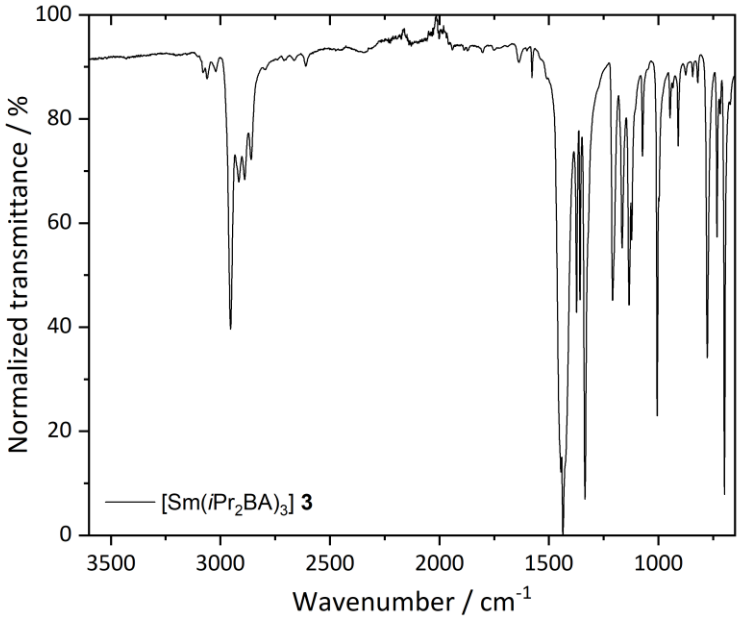

Figure S4. Normalized ATR-IR spectrum of [Sm(*i*Pr$_2$BA)$_3$] **3** complex

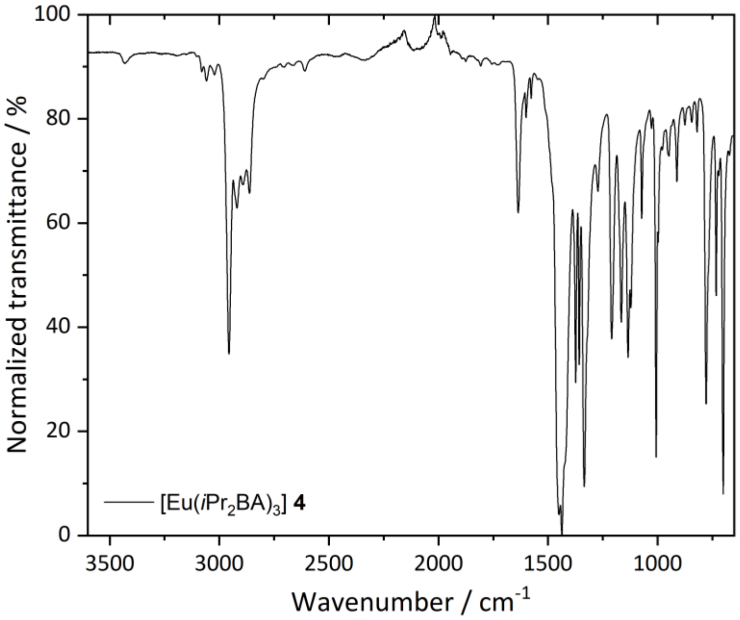

Figure S5. Normalized ATR-IR spectrum of [Eu(iPr$_2$BA)$_3$] **4** complex

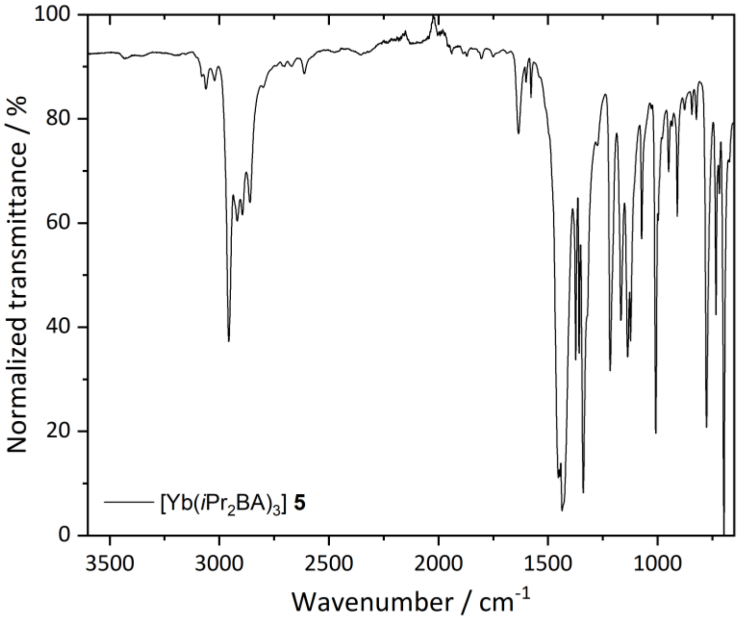

Figure S6. Normalized ATR-IR spectrum of [Yb(iPr$_2$BA)$_3$] **5** complex

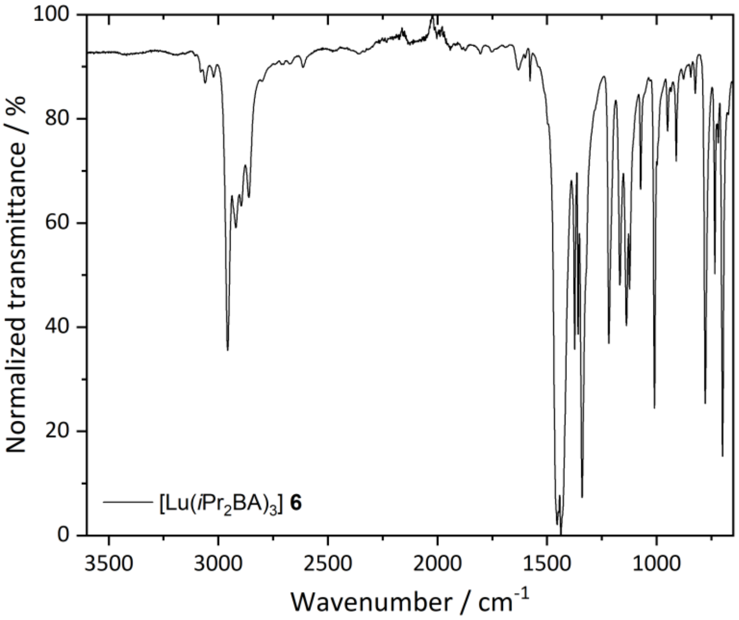

Figure S7. Normalized ATR-IR spectrum of [Lu(*i*Pr$_2$BA)$_3$] **6** complex

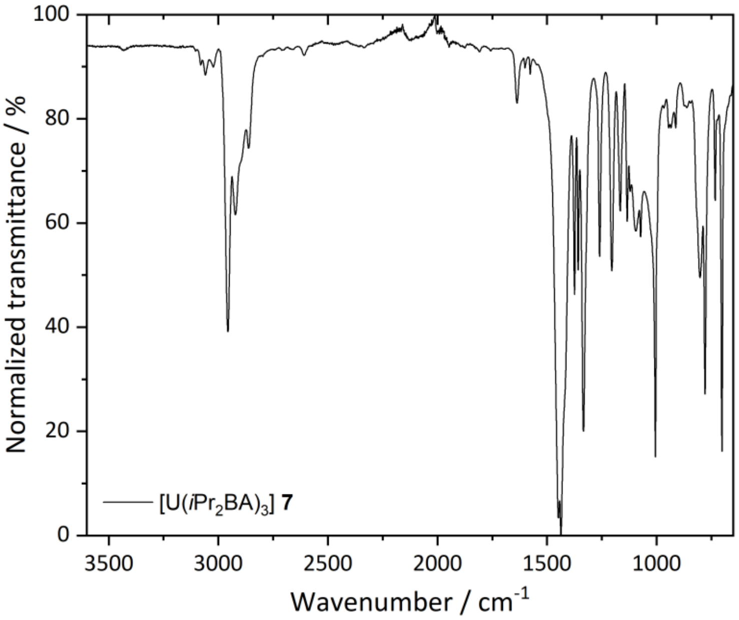

Figure S8. Normalized ATR-IR spectrum of [U(*i*Pr$_2$BA)$_3$] **7** complex

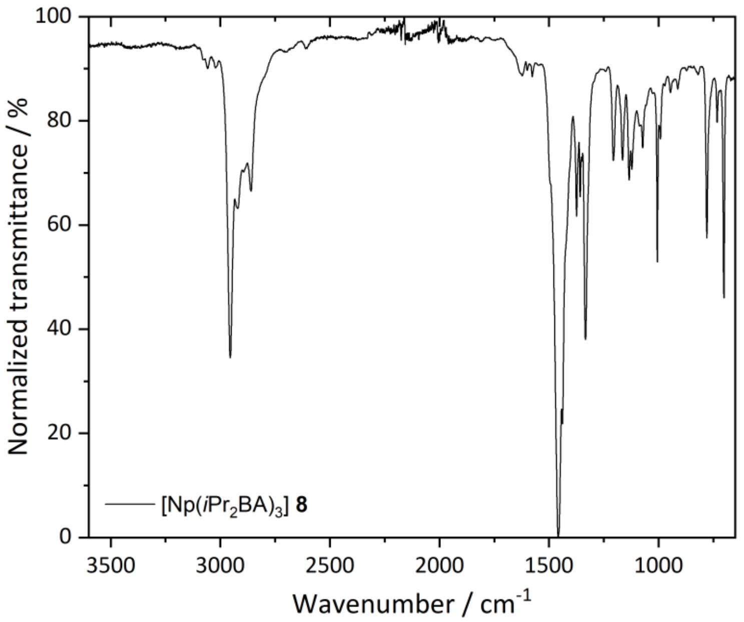

Figure S9. Normalized ATR-IR spectrum of [Np(iPr$_2$BA)$_3$] **8** complex

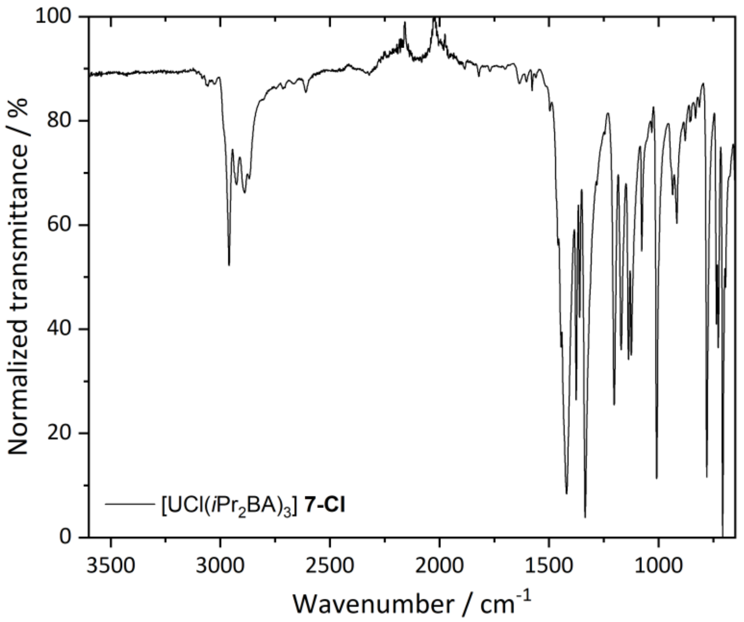

Figure S10. Normalized ATR-IR spectrum of [UCl(iPr$_2$BA)$_3$] **7-Cl** complex

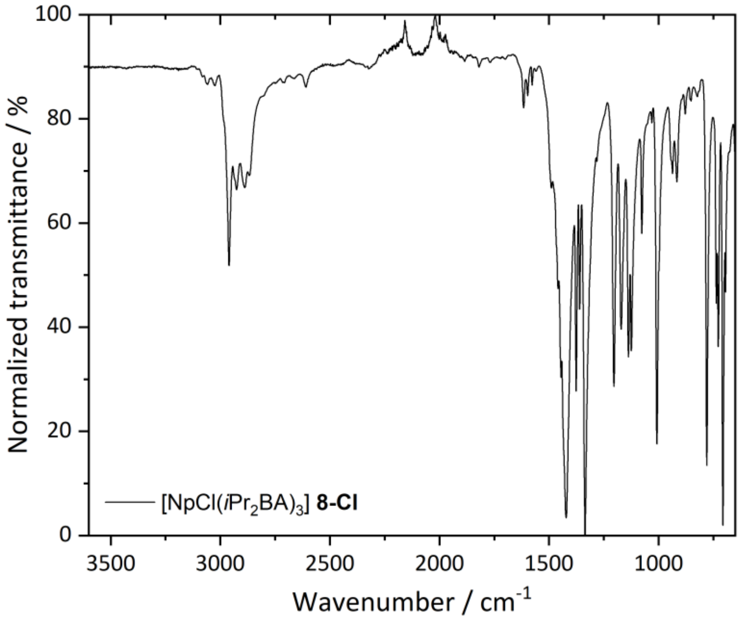

Figure S11. Normalized ATR-IR spectrum of [NpCl(*i*Pr_2BA)$_3$] **8-Cl** complex

3. Molecular structures and crystallographic data

3.1. Crystallographic Data

Table S1. SC-XRD crystal parameters of ligands.

	H*i*Pr_2BA·NCMe	K-*i*Pr_2BA
**Empirical formula**	$C_{15}H_{23}N_3$	$C_{13}H_{19}N_2K$
**M (g/mol)**	245.36	242.40
**Deposit number**	2347979	2347972
**Crystal system**	Tetragonal	Monoclinic
**Space group**	$P\bar{4}2_1c$	*Pnna*
a **(Å)**	16.9634(4)	16.1576(6)
b **(Å)**	16.9634(4)	6.4109(2)
c **(Å)**	9.8413(3)	13.0637(4)
**α (°)**	90	90
**β (°)**	90	90
**γ (°)**	90	90
V **(Å^3)**	2831.90(15)	1353.20(8)
T **(K)**	100(2)	100(2)
Z	8	4
**ρ_{calcd} (Mg/m^3)**	1.151	1.190
μ **(mm^{-1})**	0.069	0.370
**Θ_{max} (°)**	25.999	27.474
**R [$I > 2\sigma(I)$]**[a)]	0.0427	0.0318
**wR_2(int)**[a)]	0.1195	0.0867
**Data/Param**	2783/204	1558/77
**res. Dens (eÅ^{-3})**	0.456, -0.236	0.371, -0.272
**R_{int}**	0.0587	0.0412
**GooF**[b)]	1.146	1.064

a) Definition of the R indices: $R = (\Sigma||F_o|-|F_c||)/\Sigma|F_o|$; $wR_2 = \{\Sigma[w(F_o^2-F_c^2)^2]/\Sigma[w(F_o^2)^2]\}^{1/2}$ with $w = 1/[\sigma^2(F_o^2)+(aP)^2+bP]$; $P = [2F_c^2+\mathrm{Max}(F_o^2)]/3$.
b) $\{\Sigma[w(F_o^2-F_c^2)^2]/(N_o-N_p)\}^{1/2}$.

Table S2. SC-XRD crystal parameters of [Ln^{III}(iPr$_2$BA)$_3$] complexes.

	**1**, [La(iPr$_2$BA)$_3$]	**2**, [Nd(iPr$_2$BA)$_3$]	**3**, [Sm(iPr$_2$BA)$_3$]	**4**, [Eu(iPr$_2$BA)$_3$]	**5**, [Yb(iPr$_2$BA)$_3$]	**6**, [Lu(iPr$_2$BA)$_3$]	[Lu(iPr$_2$BA)$_2$Cl]$_2$
**Empirical formula**	$C_{39}H_{57}N_6La$	$C_{39}H_{57}N_6Nd$	$C_{39}H_{57}N_6Sm$	$C_{39}H_{57}N_6Eu$	$C_{39}H_{57}N_6Yb$	$C_{39}H_{57}N_6Lu$	$C_{52}H_{76}Cl_2N_8Lu_2$
**M (g/mol)**	748.81	754.14	760.25	761.86	782.94	784.87	1234.04
**Deposit number**	2347980	2347977	2347981	2347976	2347971	2347978	2347982
**Crystal system**	Monoclinic	Monoclinic	Monoclinic	Monoclinic	Monoclinic	Monoclinic	Tetragonal
**Space group**	*C*2/*c*	*C*2/*c*	*C*2/*c*	*C*2/*c*	*C*2/*c*	*C*2/*c*	$I4_1/acd$
a **(Å)**	14.1096(11)	14.149(4)	14.1700(6)	14.1789(7)	14.2372(6)	14.2208(11)	18.5824(3)
b **(Å)**	18.6707(15)	18.384(4)	17.9458(8)	17.9081(9)	17.6324(7)	17.6511(13)	18.5824(3)
c **(Å)**	15.5805(13)	15.571(4)	15.6872(7)	15.6809(8)	15.6226(7)	15.6369(12)	32.8526(7)
**α (°)**	90	90	90	90	90	90	90
**β (°)**	98.992(3)	104.841(8)	95.4932(17)	95.5046(17)	94.6528(16)	94.871(3)	90
**γ (°)**	90	90	90	90	90	90	90
**V (Å^3)**	4054.0(6)	4013.7(17)	3970.8(3)	3963.3(3)	3908.9(3)	3910.9(5)	11344.2(4)
**T (K)**	100(2)	100(2)	100(2)	100(2)	100(2)	100(2)	100(2)
Z	4	4	4	4	4	4	8
**ρ_{calcd} (Mg/m^3)**	1.227	1.248	1.272	1.277	1.330	1.333	1.445
**μ (mm^{-1})**	1.086	1.326	1.511	1.615	2.425	2.557	3.594
**Θ_{max} (°)**	25.998	27.099	29.997	29.998	26.000	27.500	27.487
**R [$I > 2\sigma(I)$]**[a]	0.0518	0.0277	0.0189	0.0212	0.0232	0.0179	0.0142
**wR_2(int)**[a]	0.1132	0.0641	0.0439	0.0489	0.0494	0.0393	0.0292
**Data/Param**	3980/230	4422/254	5782/230	5767/231	3847/230	4508/230	3255/150
**res. Dens (eÅ^{-3})**	1.038, -1.750	0.891, -0.979	1.302, -0.537	1.803, -0.567	0.518, -1.157	1.580, -0.731	0.288, -0.334
**R_{int}**	0.0670	0.0356	0.0415	0.0464	0.0829	0.0511	0.0348
**GooF**[b]	1.234	1.098	1.087	1.096	1.085	1.097	1.171

[a] Definition of the R indices: $R = (\Sigma||F_o|-|F_c||)/\Sigma|F_o|$; $wR_2 = \{\Sigma[w(F_o^2-F_c^2)^2]/\Sigma[w(F_o^2)^2]\}^{1/2}$ with $w = 1/[\sigma^2(F_o^2)+(a\mathrm{P})^2+b\mathrm{P}]$; $\mathrm{P} = [2F_c^2+\mathrm{Max}(F_o^2)]/3$.
[b] $\{\Sigma[w(F_o^2-F_c^2)^2]/(N_o-N_p)\}^{1/2}$.

Table S3. SC-XRD crystal parameters of $[An^{III}(iPr_2BA)_3]$ and $[An^{IV}Cl(iPr_2BA)_3]$ complexes.

	**7**, $[U(iPr_2BA)_3]$	**8**, $[Np(iPr_2BA)_3]$		**7-Cl**, $[UCl(iPr_2BA)_3]$	**8-Cl**, $[NpCl(iPr_2BA)_3]$
**Empirical formula**	$C_{39}H_{57}N_6U$	$C_{39}H_{57}N_6Np$		$C_{39}H_{57}ClN_6U$	$C_{39}H_{57}ClN_6Np$
**M (g/mol)**	847.93	846.90		883.38	882.35
**Deposit number**	2347970	2347983	2347974	2347975	2347973
**Crystal system**	Monoclinic	Monoclinic	Monoclinic	Cubic	Cubic
**Space group**	$C2/c$	$P2_1/c$	$C2/c$	$Pa\bar{3}$	$Pa\bar{3}$
a **(Å)**	14.1107(9)	14.1041(4)	14.1946(4)	20.5866(10)	20.6324(3)
b **(Å)**	18.4420(10)	36.7609(10)	18.5587(5)	20.5866(10)	20.6324(3)
c **(Å)**	15.6433(9)	15.5861(4)	15.7336(4)	20.5866(10)	20.6324(3)
**α (°)**	90	90	90	90	90
**β (°)**	97.977(2)	97.7140(9)	97.9259(8)	90	90
**γ (°)**	90	90	90	90	90
V **(Å³)**	4031.5(4)	8007.9(4)	4105.16(19)	8724.8(13)	8783.1(4)
T **(K)**	100(2)	100(2)	200(2)	199(2)	199(2)
Z	4	8	4	8	8
ρ_{calcd} **(Mg/m³)**	1.397	1.405	1.370	1.345	1.335
μ **(mm⁻¹)**	4.058	2.628	2.563	3.813	2.457
Θ_{max} **(°)**	27.998	28.500	25.693	25.478	25.439
$R\ [I > 2\sigma(I)]$[a)]	0.0290	0.0294	0.0250	0.0360	0.0296
wR_2**(int)**[a)]	0.0624	0.0535	0.0544	0.0822	0.0783
**Data/Param**	4873/254	20255/853	3898/206	2714/146	2716/146
**res. Dens (eÅ⁻³)**	1.852, -1.362	1.030, -1.316	1.430, -0.574	0.630, -1.081	1.670, -0.693
R_{int}	0.0601	0.0543	0.0507	0.1181	0.0716
**GooF**[b)]	1.083	1.123	1.092	1.035	1.098

[a)] Definition of the R indices: $R = (\Sigma||F_o|-|F_c||)/\Sigma|F_o|$; $wR_2 = \{\Sigma[w(F_o^2-F_c^2)^2]/\Sigma[w(F_o^2)^2]\}^{1/2}$ with $w = 1/[\sigma^2(F_o^2)+(aP)^2+bP]$; $P = [2F_c^2+\mathrm{Max}(F_o^2)]/3$.

[b)] $\{\Sigma[w(F_o^2-F_c^2)^2]/(N_o-N_p)\}^{1/2}$.

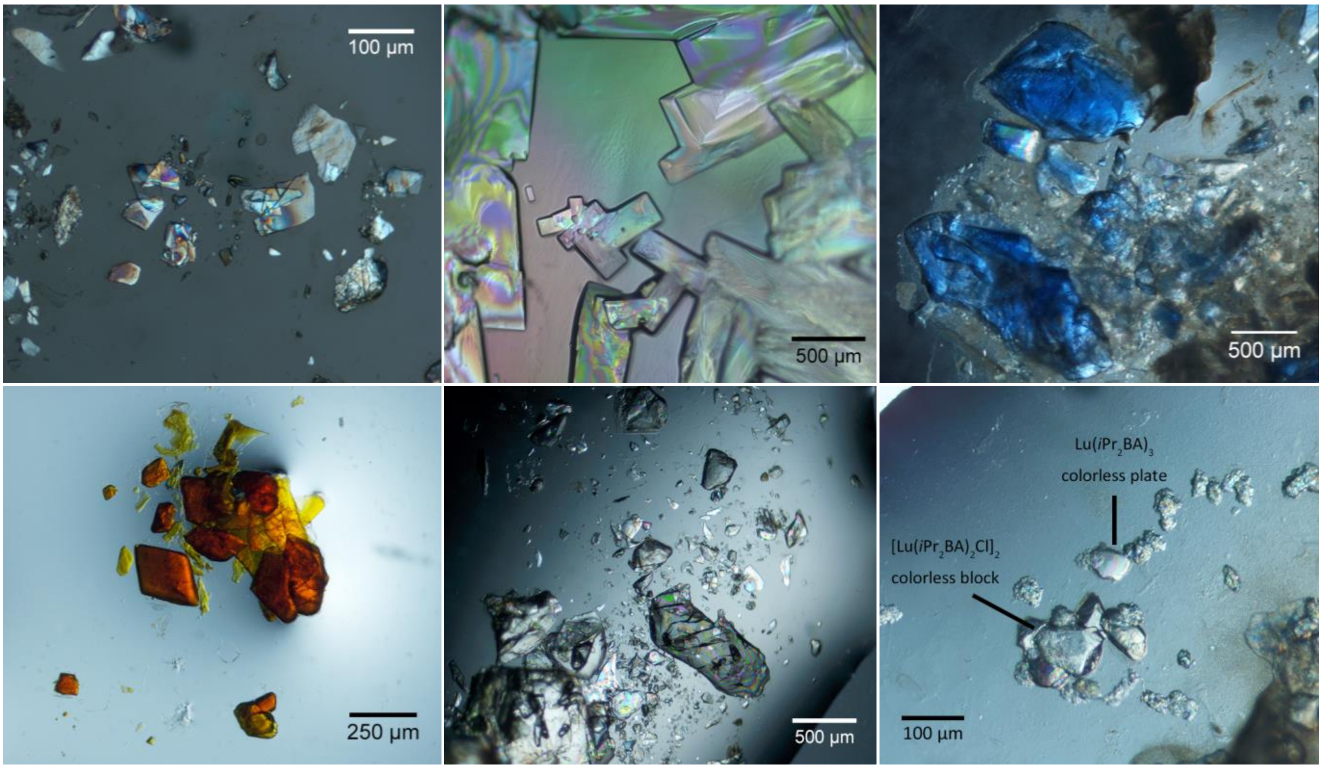

Figure S12. Crystals of trivalent lanthanide complexes [Ln^{III}(*i*Pr$_2$BA)$_3$] (Ln = La (**1,** top left), Nd (**2,** top center), Sm (**3,** top right), Eu (**4,** bottom left), Yb (**5,** bottom center), Lu (**6,** bottom right)).

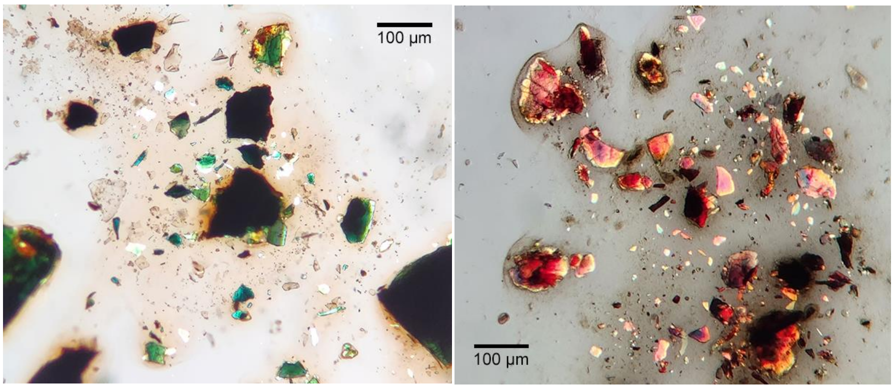

Figure S13. Crystals of trivalent actinide complexes [An^{III}(*i*Pr$_2$BA)$_3$] (An = U (**7**, left), Np (**8**, right)).

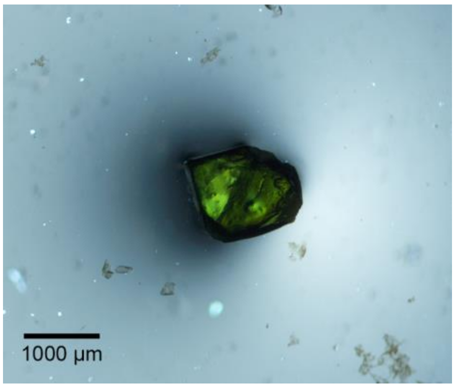

Figure S14. Crystals of tetravalent actinide complex [An^{IV}Cl(*i*Pr$_2$BA)$_3$] (An = U (**7-Cl**))

3.2. Structural Data and Geometrical Analysis

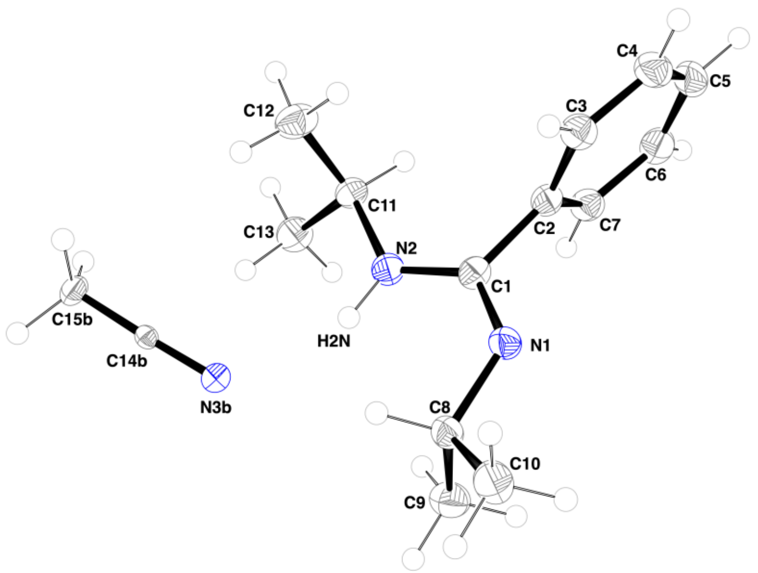

Figure S15. Molecular structure of [H*i*Pr$_2$BA·NCMe]. Ellipsoids are drawn at 50% probability level. Hydrogen atoms are omitted for clarity. The NCMe and the nitrogen bond H atom are disordered in two positions. Only one is depicted as representative example.

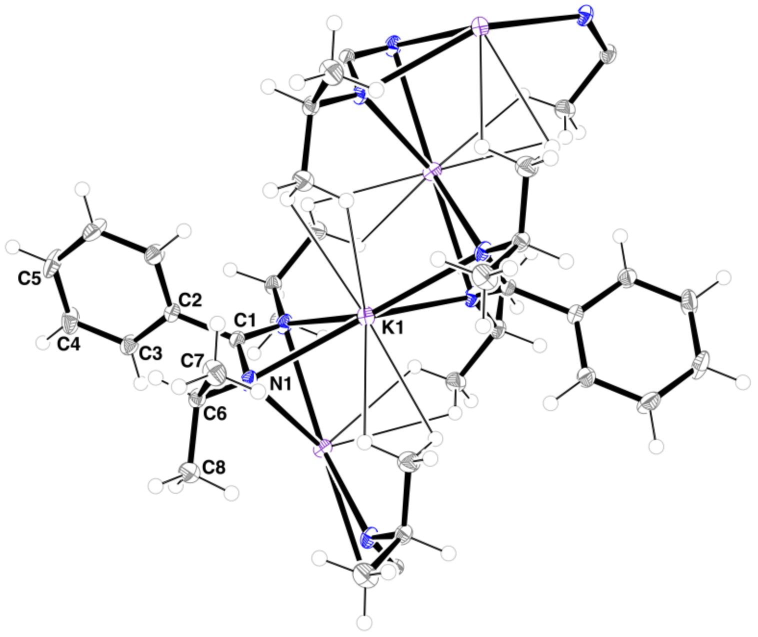

Figure S16. Molecular structure of [K-*i*Pr$_2$BA]. Ellipsoids are drawn at 50% probability level. The asymmetric unit contains half an *i*Pr$_2$BA anion and half a potassium ion.

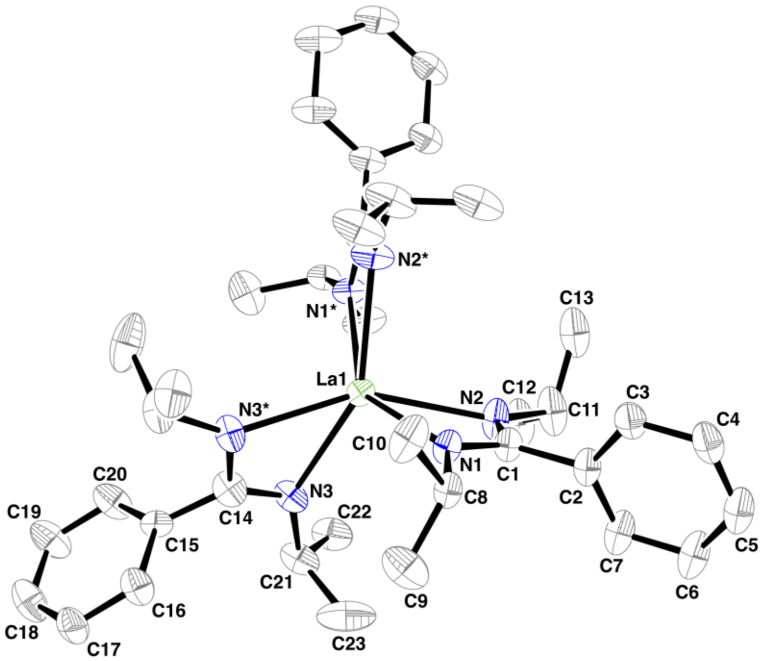

Figure S17. Molecular structure of [La(iPr$_2$BA)$_3$] (**1**). Ellipsoids are drawn at 30% probability level. Hydrogen atoms are omitted for clarity. The isopropyl group at N2 is disorder in two positions and the phenyl group at C14 is disordered along the two-fold rotation axis. Only one part of the disorder is depicted for clarity. La1 is located on a two-fold rotation axis: Symmetry transformation *−x+1, y, −z+3/2.

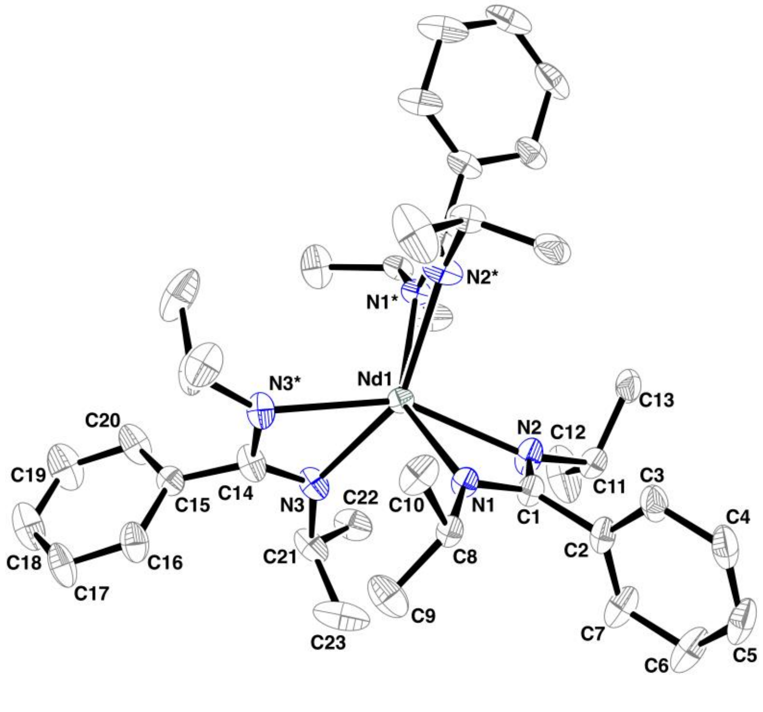

Figure S18. Molecular structure of [Nd(iPr$_2$BA)$_3$] (**2**). Ellipsoids are drawn at 30% probability level. Hydrogen atoms are omitted for clarity. The isopropyl group at N2 is disorder in two positions and the phenyl group at C14 is disordered along the two-fold rotation axis. Only one part of the disorder is depicted for clarity. Nd1 is located on a two-fold rotation axis: Symmetry transformation *−x+1, y, −z+3/2.

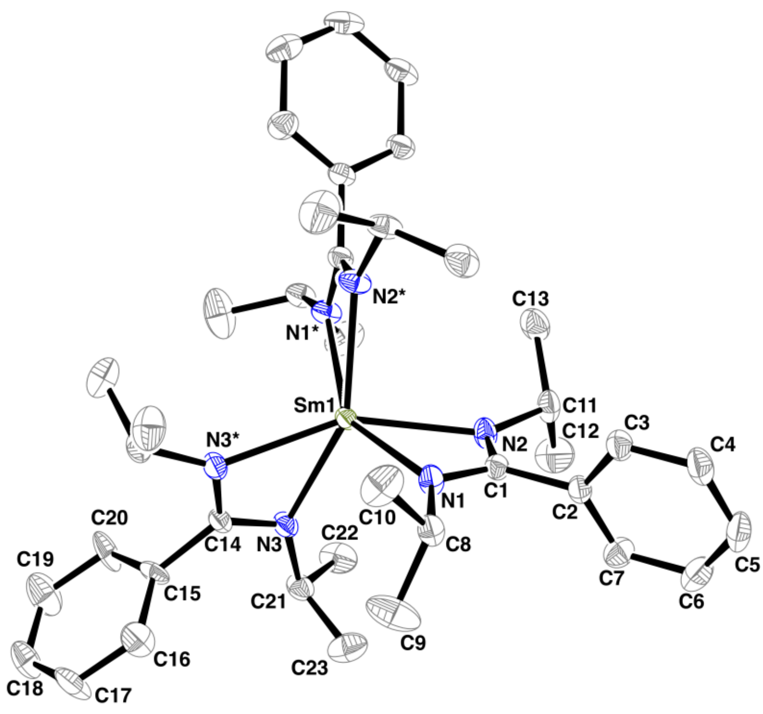

Figure S19. Molecular structure of [Sm(iPr$_2$BA)$_3$] (**3**). Ellipsoids are drawn at 50% probability level. Hydrogen atoms are omitted for clarity. The phenyl group at C14 is disordered along the two-fold rotation axis. Only one part of the disorder is depicted for clarity. Sm1 is located on a two-fold rotation axis: Symmetry transformation *−x+1, y, −z+3/2.

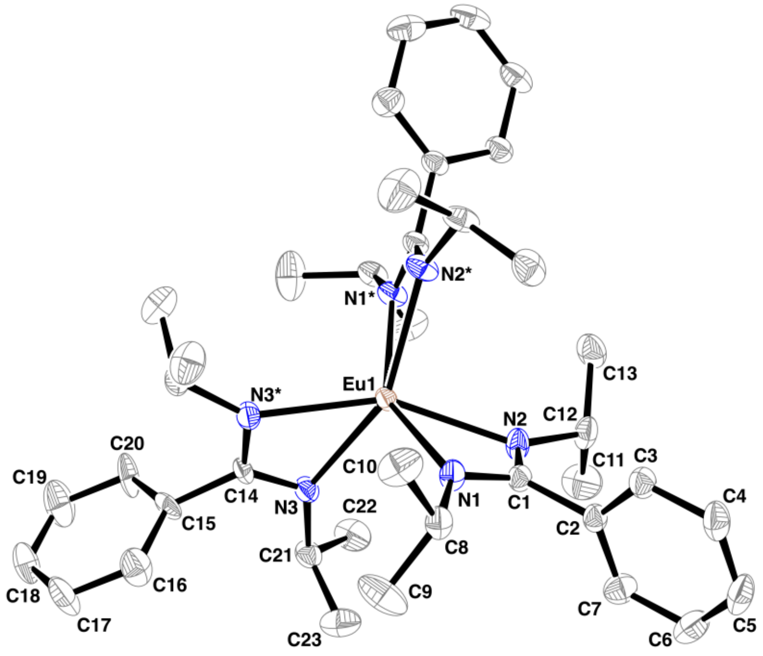

Figure S20. Molecular structure of [Eu(iPr$_2$BA)$_3$] (**4**). Ellipsoids are drawn at 50% probability level. Hydrogen atoms are omitted for clarity. The phenyl group at C14 is disordered along the two-fold rotation axis. Only one part of the disorder is depicted for clarity. Eu1 is located on a two-fold rotation axis: Symmetry transformation *−x+1, y, −z+3/2.

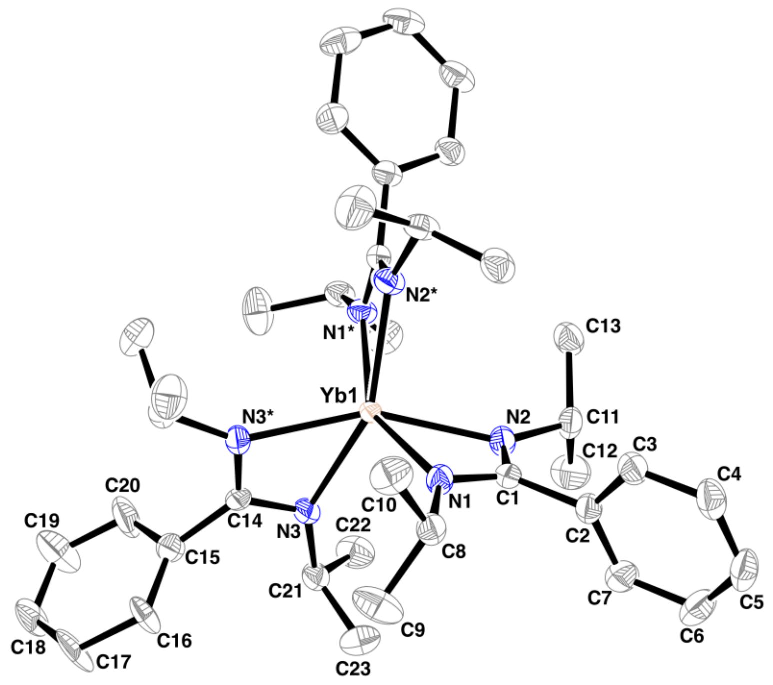

Figure S21. Molecular structure of [Yb(*i*Pr$_2$BA)$_3$] (**5**). Ellipsoids are drawn at 50% probability level. Hydrogen atoms are omitted for clarity. The phenyl group at C14 is disordered along the two-fold rotation axis. Only one part of the disorder is depicted for clarity. Yb1 is located on a two-fold rotation axis: Symmetry transformation *−x+1, y, −z+3/2.

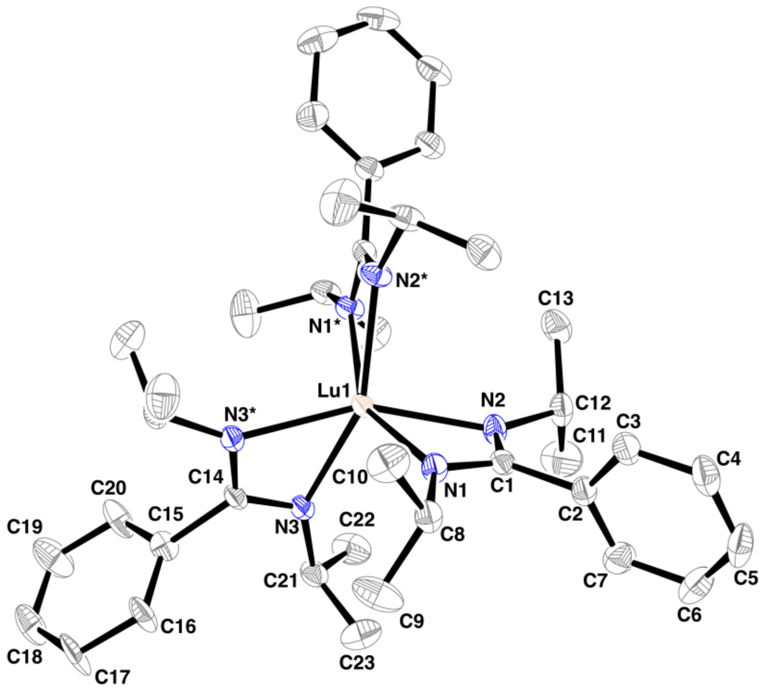

Figure S22. Molecular structure of [Lu(*i*Pr$_2$BA)$_3$] (**6**). Ellipsoids are drawn at 50% probability level. Hydrogen atoms are omitted for clarity. The phenyl group at C14 is disordered along the two-fold rotation axis. Only one part of the disorder is depicted for clarity. Lu1 is located on a two-fold rotation axis: Symmetry transformation *−x+1, y, −z+3/2.

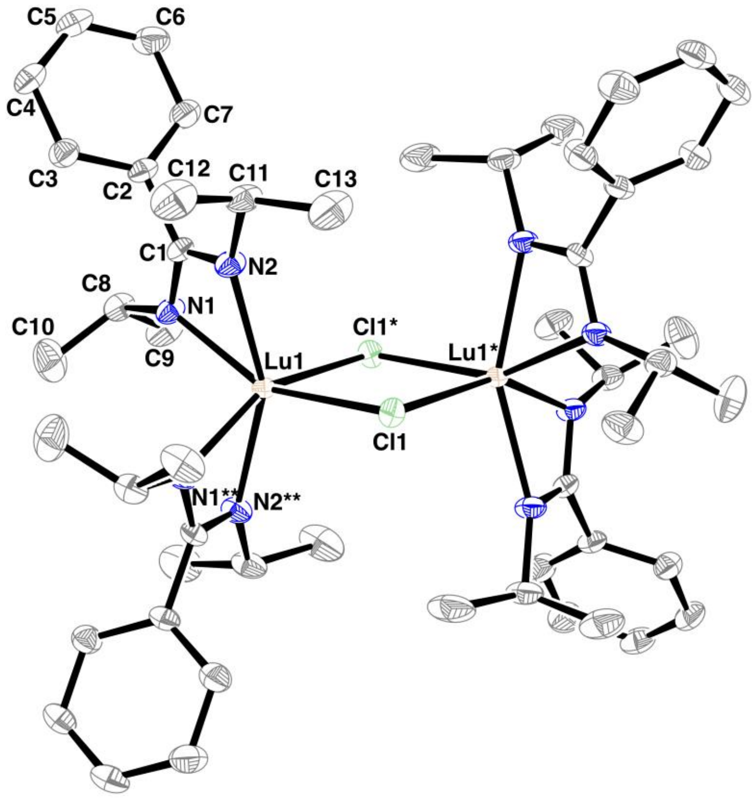

Figure S23. Molecular structure of [Lu(iPr$_2$BA)$_2$Cl]$_2$. Ellipsoids are at the 50% probability level. Hydrogen atoms are omitted for clarity. Lu1 is located on a two-fold rotation axis and Cl1 is located on a two-fold rotoinversion axis.

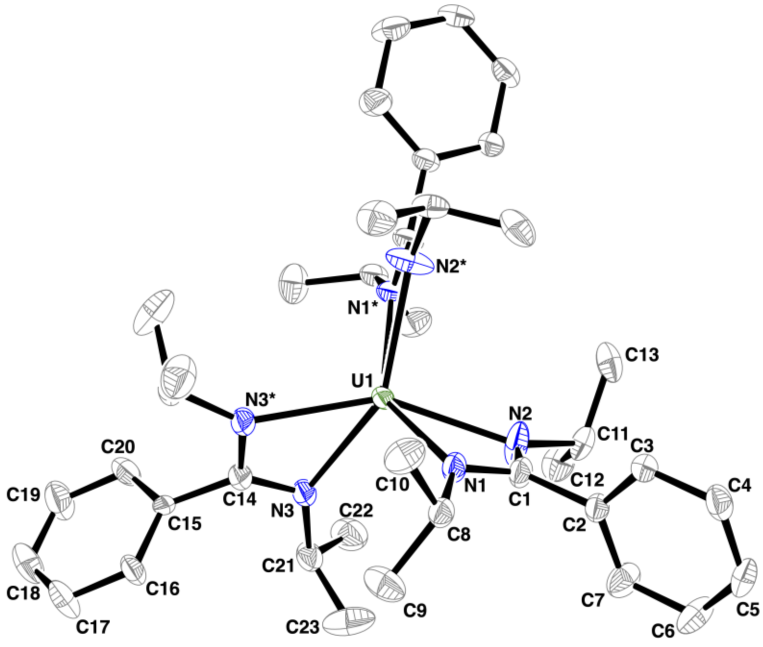

Figure S24. Molecular structure of [U(iPr$_2$BA)$_3$] (**7**). Ellipsoids are drawn at 30% probability level. Hydrogen atoms are omitted for clarity. The isopropyl group at N2 is disorder in two positions and the phenyl group at C14 is disordered along the two-fold rotation axis. Only one part of the disorder is depicted for clarity. U1 is located on a two-fold rotation axis: Symmetry transformation *−x+1, y, −z+3/2.

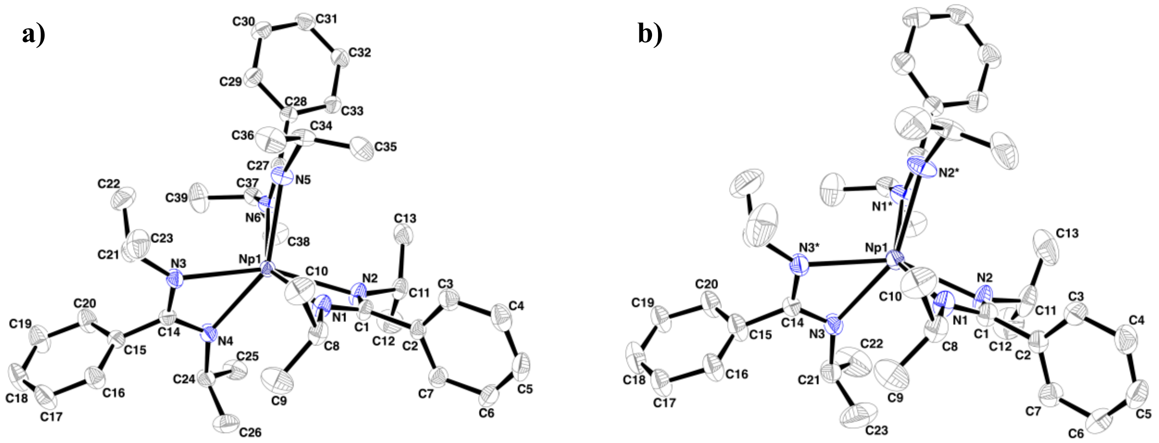

Figure S25. Molecular structure of [Np(*i*Pr$_2$BA)$_3$] (**8**). Hydrogen atoms are omitted for clarity. **a**) Resolved superstructure in P2(1)/c at 100 K. The asymmetric unit contains two complex molecules, but second molecule is omitted for clarity. Ellipsoids are drawn at 50% probability level. **b**) Molecular structure in C2/c at 200 K. Ellipsoids are drawn at 30% probability level. The phenyl group at C14 is disordered along the two-fold rotation axis. Only one part of the disorder is depicted for clarity. Np1 is located on a two-fold rotation axis: Symmetry transformation *−x+1, y, −z+3/2.

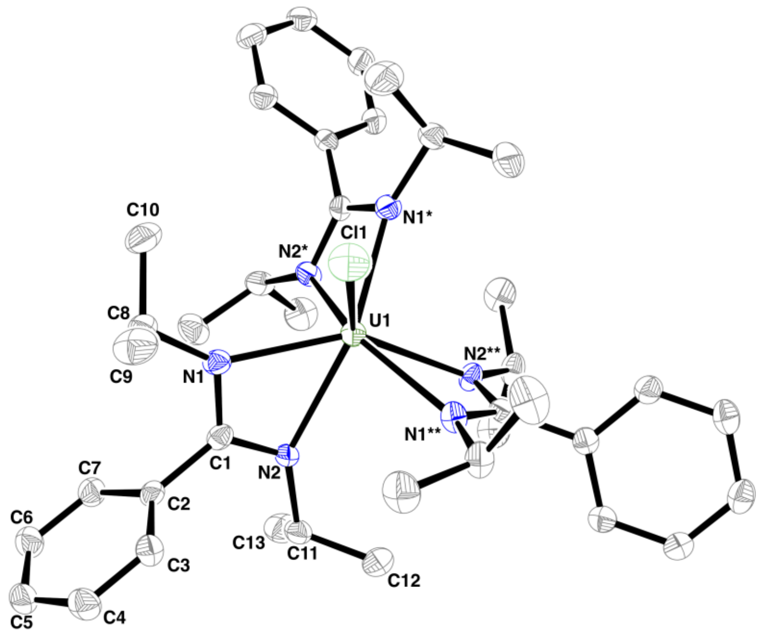

Figure S26. Molecular structure of [UCl(*i*Pr$_2$BA)$_3$]·0.5toluene (**7-Cl**). Ellipsoids are drawn at 30% probability level. Hydrogen atoms are omitted for clarity. The U1-Cl1-axis lays on a 3-fold rotation axis. The data set was treated with SQUEEZE function of the PLATON[7] (259 electrons/unit cell, corresponding to 4 toluene molecules in the unit).

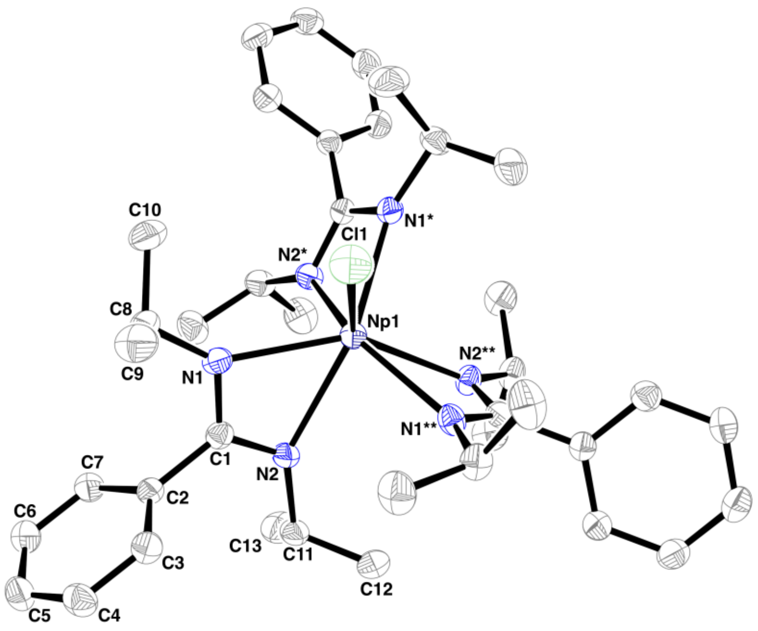

Figure S27. Molecular structure of [NpCl(*i*Pr_2BA)$_3$]·0.5toluene (**8-Cl**). Ellipsoids are drawn at 30% probability level. Hydrogen atoms are omitted for clarity. The Np1-Cl1-axis lays on a 3-fold rotation axis. The data set was treated with SQUEEZE function of the PLATON[7] (266 electrons/unit cell, corresponding to 4 toluene molecules in the unit). The data set was treated with XABS2 program.[8]

Table S4. Bond length (Å) for [M^{III}(*i*Pr_2BA)$_3$] (M^{III} = Ln^{III} or An^{III}) complexes. Measurement uncertainties and standard deviations of average are indicated by brackets on the last digits.

	La (**1**)	Ce[9]	Nd (**2**)	Sm (**3**)	Eu (**4**)	Yb (**5**)	Lu (**6**)	U (**7**)	Np (**8**) *C2/c*
M-N1	2.511(4)	2.4820(17)	2.454(2)	2.4210(12)	2.4096(13)	2.318(2)	2.3141(15)	2.475(3)	2.463(3)
M-N2	2.511(4)	2.4860(14)	2.449(2)	2.4276(12)	2.4137(14)	2.314(2)	2.3130(16)	2.469(3)	2.462(3)
M-N1*	2.511(4)	2.4820(17)	2.454(2)	2.4210(12)	2.4096(13)	2.318(2)	2.3141(15)	2.475(3)	2.463(3)
M-N2*	2.511(4)	2.4860(14)	2.449(2)	2.4276(12)	2.4137(14)	2.314(2)	2.3130(16)	2.469(3)	2.462(3)
M-N3	2.520(4)	2.4924(15)	2.459(2)	2.4353(12)	2.4202(13)	2.323(2)	2.3207(15)	2.485(3)	2.469(3)
M-N3*	2.520(4)	2.4924(15)	2.459(2)	2.4353(12)	2.4202(13)	2.323(2)	2.3207(15)	2.485(3)	2.469(3)
Mean(M-N)	2.514(6)	2.487(5)	2.454(5)	2.428(7)	2.415(5)	2.318(5)	2.316(4)	2.476(8)	2.465(5)

Table S5. Bond length (Å) for two [Np^{III}(*i*Pr_2BA)$_3$] (**8**) complexes from the *P*2(1)/c crystal structure. Measurement uncertainties and standard deviations of average are indicated by brackets on the last digits.

Np1	Np (**8**) *P*2_1/*c*	Np2	Np (**8**) *P*2_1/*c*
Np1-N1	2.464(2)	Np2-N7	2.460(2)
Np1-N2	2.472(2)	Np2-N8	2.464(2)
Np1-N3	2.486(2)	Np2-N9	2.473(2)
Np1-N4	2.471(2)	Np2-N10	2.469(2)
Np1-N5	2.459(2)	Np2-N11	2.456(2)
Np1-N6	2.462(2)	Np2-N12	2.480(2)
Mean(Np1-N)	2.469(10)	Mean(Np2-N)	2.467(9)

Table S6. Bond length (Å) for [An^{IV}Cl(*i*Pr_2BA)$_3$]·0.5toluene complexes. Measurement uncertainties and standard deviations of average are indicated by brackets on the last digits.

	U (**7-Cl**)	Np (**8-Cl**)
An-N1	2.378(4)	2.373(3)
An-N2	2.454(4)	2.445(3)
An-N1*	2.378(4)	2.373(3)
An-N2*	2.454(4)	2.445(3)
An-N1**	2.378(4)	2.373(3)
An-N2**	2.454(4)	2.445(3)
Mean(An-N)	2.416(44)	2.409(42)
An-Cl	2.664(2)	2.662(2)

Table S7. Bite angle and torsion angle (°) for [M^{III}($i$$Pr_2$BA)$_3$] ($M^{III}$ = Ln^{III} or An^{III}) complexes. G1 and G2 represent the centroids formed by the upper and lower nitrogen atoms in the coordination sphere, respectively. The dihedral angle (τ, N_a-G1-G2-N_b) represents the torsion angle of the ligand binding plane against the pseudo-axial symmetry axis (G1-G2). Coordinates for centroids G1 and G2, along with the dihedral angle τ, were determined using the Diamond[10] program. Measurement uncertainties and standard deviations of average are indicated by brackets on the last digits.

	La (**1**)	Nd (**2**)	Sm (**3**)	Eu (**4**)	Yb (**5**)	Lu (**6**)	U (**7**)	Np (**8**) *C2/c*
N1-M-N2	53.39(13)	54.82(7)	55.57(4)	55.87(5)	58.25(8)	58.26(5)	53.98(9)	54.30(9)
N1*-M-N2*	53.39(13)	54.82(7)	55.57(4)	55.86(5)	58.25(8)	58.26(5)	53.98(9)	54.30(9)
N3-M-N3*	53.1(2)	54.85(11)	55.58(6)	55.93(6)	58.72(11)	58.40(8)	54.05(12)	54.43(12)
Mean(N_a-M-N_b)	53.3(2)	54.8(1)	55.6(1)	55.9(1)	58.4(3)	58.3(1)	54.0(1)	54.3(1)
N1-G1-G2-N2	25.21(16)	26.69(8)	29.32(5)	29.05(5)	30.78(9)	30.73(6)	25.21(12)	25.49(11)
N1*-G1-G2-N2*	25.21(16)	26.69(8)	29.32(5)	29.05(5)	30.78(9)	30.73(6)	25.21(12)	25.49(11)
N3-G1-G2-N3*	23.71(14)	25.20(8)	26.71(5)	26.74(5)	29.46(9)	29.14(6)	24.11(1)	24.83(10)
Mean(N_a-G1-G2-N_b)	24.7(8)	26.2(8)	28(1)	28(1)	30.3(7)	30.2(8)	24.8(6)	25.3(4)

Table S8. Bite angle and torsion angle (°) for two [Np^{III}($i$$Pr_2$BA)$_3$] (**8**) complexes from the $P2_1$/c crystal structure. G1 and G2 represent the centroids formed by the upper and lower nitrogen atoms in the coordination sphere, respectively. The dihedral angle (τ, N_a-G1-G2-N_b) represents the torsion angle of the ligand binding plane against the pseudo-axial symmetry axis (G1-G2). Coordinates for centroids G1 and G2, along with the dihedral angle τ, were determined using the Diamond[10] program. Measurement uncertainties and standard deviations of average are indicated by brackets on the last digits.

Np1	Np (**8**) $P2_1/c$	Np2	Np (**8**) $P2_1/c$
N1-Np1-N2	54.38(8)	N7-Np2-N8	54.48(7)
N3-Np1-N4	54.42(7)	N9-Np2-N10	54.34(8)
N5-Np1-N6	54.49(7)	N11-Np2-N12	54.58(7)
Mean(N_a-Np1-N_b)	54.43(10)	Mean(N_a-Np2-N_b)	54.47(14)
N1-G1-G2-N2	26.21(9)	N7-G1-G2-N8	24.31(9)
N3-G1-G2-N4	23.95(8)	N9-G1-G2-N10	27.96(9)
N5-G1-G2-N6	23.68(9)	N11-G1-G2-N12	24.36(9)
Mean(N_a-G1-G2-N_b)	24.6(1.2)	Mean(N_a-G1-G2-N_b)	25.5(1.9)

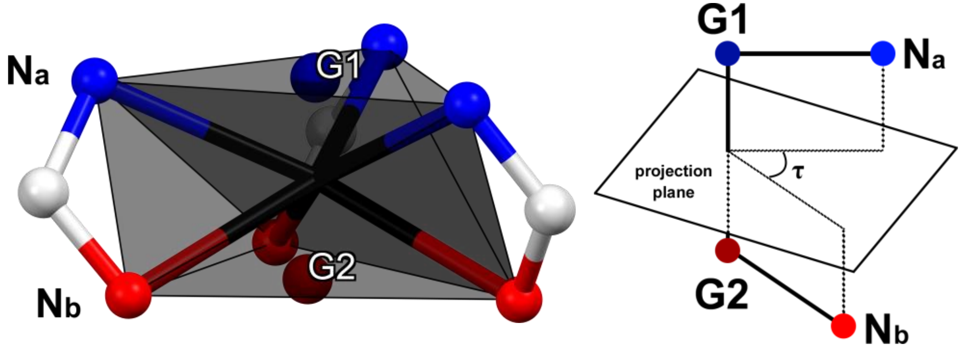

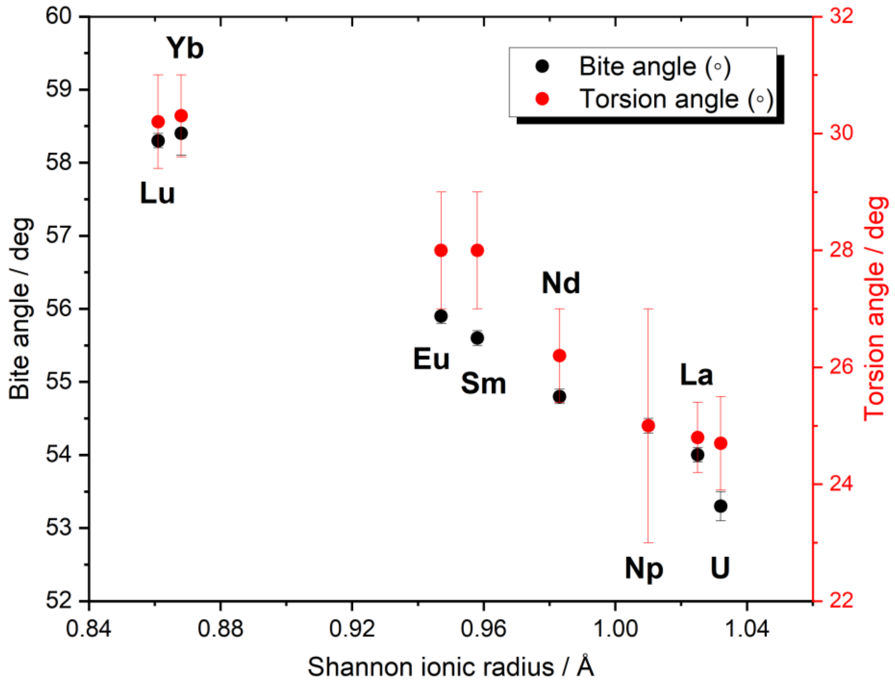

Figure S28. Relation between Shannon ionic radii[11] and the averaged dihedral angle (τ) of ligands in [M^{III}(*i*Pr$_2$BA)$_3$] complexes.

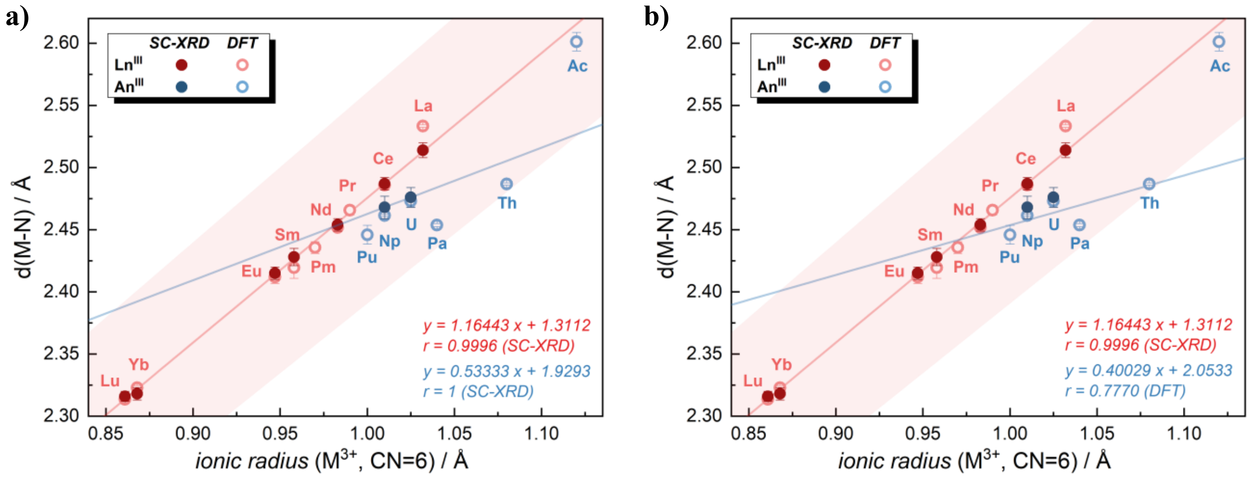

Figure S29. Relation of average bond length (M–N) and ionic radii[11] of trivalent metal ions with coordination number 6 for [M^{III}(*i*Pr$_2$BA)$_3$] complexes. The red line represents the linear regression of experimental Ln–N distances with a 3-sigma error range (light red area), while the blue line represents the linear regression of **(a)** the experimental An–N distances and **(b)** the computationally determined An–N distances.

M^{III}(*i*Pr$_2$BA)$_3$ (CN = 6)	Intercept	Slope	R^2	*r*	Note
Ln^{3+} and An^{3+} (SC-XRD)	1.35(4)	1.13(4)	0.99	0.9953	-
Ln^{3+} (SC-XRD)	1.31(1)	1.16(1)	0.99	0.9996	-
An^{3+} (SC-XRD)	1.93	0.53	-	1	2 data points (U and Np)
Ln^{3+} and An^{3+} (DFT)	1.46(9)	1.00(9)	0.90	0.9542	-
Ln^{3+} (DFT)	1.30(3)	1.18(3)	0.99	0.9977	-
An^{3+} (DFT)	2.05(17)	0.40(16)	0.50	0.7770	-
An^{3+} (DFT)	1.20(8)	1.25(8)	0.99	0.9964	Without Th and Pa

Metal−ligand distances and ionic radii for various isostructural metal complexes with different donor ligands, as related to Figure 3 in main text.

	d(M-Cl) / Å	M^{4+} ionic radius (CN 6)[a] / Å	Ref
$[TiCl_6]^{2-}$	2.34(1)	0.605	12
$[HfCl_6]^{2-}$	2.45(1)	0.71	12
$[ZrCl_6]^{2-}$	2.47(1)	0.72	12
$[PuCl_6]^{2-}$	2.59(1)	0.86	12
$[NpCl_6]^{2-}$	2.61(2)	0.87	12
$[UCl_6]^{2-}$	2.62(1)	0.89	12
$[ThCl_6]^{2-}$	2.68(1)	0.94	12

	d(M-N) / Å	M^{3+} ionic radius (CN 3)[a] / Å	Ref
$Yb(L^1)_3$	2.16(1)	0.70	13
$Tm(L^1)_3$·*n*-hexane	2.20(2)	0.72	14
$Er(L^1)_3$	2.21(1)	0.73	15
$Ho(L^1)_3$	2.21(2)	0.74	16
$Dy(L^1)_3$	2.214(4)	0.75	15
$Dy(L^1)_3$	2.24(1)	0.75	16
$Tb(L^1)_3$	2.23(1)	0.76	17
$Tb(L^1)_3$	2.258(4)	0.76	16
$Eu(L^1)_3$	2.26(1)	0.78	13
$Gd(L^1)_3$	2.26(1)	0.77	16
$Sm(L^1)_3$	2.284(3)	0.79	18
$Nd(L^1)_3$	2.29(2)	0.81	19
$Ce(L^1)_3$	2.319(3)	0.84	20
$La(L^1)_3$	2.36(1)	0.86	21
$Pu(L^1)_3$·*n*-pentane	2.31(1)	0.83	22
$Np(L^1)_3$·diethylether	2.338(3)	0.84	23
$U(L^1)_3$·cyclohexane	2.32(1)	0.85	24

	d(M-Se) / Å	M^{3+} ionic radius (CN 8)[a] / Å	Ref
$Nd(L^2)_3(THF)_2$	3.09(4)	1.11	25
$Ce(L^2)_3(THF)_2$	3.11(4)	1.14	25
$La(L^2)_3(THF)_2$	3.13(3)	1.16	25
$Pu(L^2)_3(THF)_2$	3.08(3)	1.07	25

	d(M-S) / Å	M^{3+} ionic radius (CN 6)[a] / Å	Ref
$Ce(L^3)_3$	2.864(2)	1.10	26
$La(L^3)_3$	2.892(1)	1.032	26
$Pu(L^3)_3$	2.819(3)	1.00	26
$U(L^3)_3$	2.854(7)	1.025	26

	d(M-C) / Å	M^{3+} ionic radius (CN 9)[a] / Å	Ref
$Yb(L^4)_3$	2.390(5)	1.04	27
$Dy(L^4)_3$	2.432(3)	1.08	28
$Gd(L^4)_3$	2.463(3)	1.11	28
$Sm(L^4)_3$	2.486(1)	1.13	29
$Nd(L^4)_3$	2.52(4)	1.16	28
$Ce(L^4)_3$	2.57(2)	1.20	30
$La(L^4)_3$	2.60(1)	1.22	28
$Pu(L^4)_3$	2.51(8)	1.11	31
$U(L^4)_3$	2.54(3)	1.13	32
$Th(L^4)_3$	2.519(2)	1.19	33

[a] For ionic radius values not found in the reference[11], effective metal ionic radii were calculated according to RAYMOND and EIGENBROT's method[13] based on coordination number, ensuring a consistent framework for systematic comparison.

L^1 = $N(SiMe_3)_2$; bis(trimethylsilyl)amido
L^2 = Se_2PPh_2; diphenylphosphinodiselenoato
L^3 = $N(SP\textit{i}Pr_2)_2$; *S,S'*-bis(diisopropylthiophosphoryl)amido
L^4 = $C_5H_3(SiMe_3)_2$; η^5-1,3-bis(trimethylsilyl)cyclopentadienyl

4. Quantum chemical calculations

4.1. Structure optimization

The optimization calculations were carried out using TurboMole 7.3.1.[34] Frequency analyses for the confirmation of found minima were done using the def-SVP basis. After confirmation, the structure was refined using the bigger def-TZVPP basis set. In order to simulate the solvent, the COSMO (*Conductor-like Screening Model*)[35] (ε(THF) = 7.58) was applied. Scalar-relativistic effects originating in the heavy Ln and especially An atoms were at least partially accounted for by using ECPs (*Effective Core Potential*) replacing – depending on the element – the inner 28, 46 or 60 core electrons. (ECPs were attributed automatically by TurboMole)

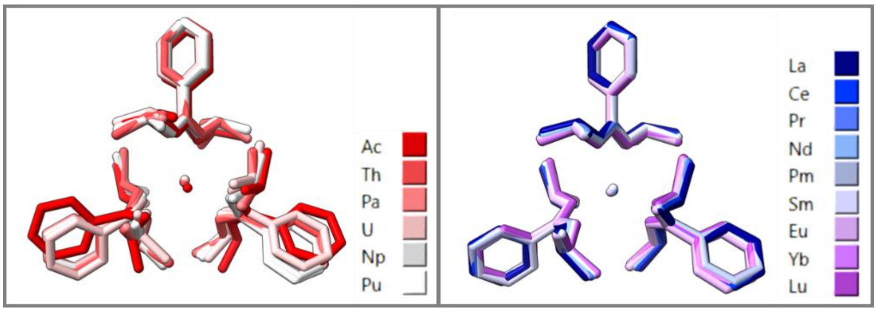

Figure S30. Overlay of the optimized [An^{III}/Ln^{III}($i$$Pr_2$BA)$_3$] complexes

XYZ coordinates of optimized structures (def-TZVPP basis)

Below the coordinates (given in Å) of all theoretically investigated Ln and An complexes – obtained from structure optimization with the def-TZVPP basis – are listed in the xyz format. These were subsequently used for all mentioned *all electron*-DFT and CAS investigations.

Lanthanide complexes:

[La(*i*Pr$_2$BA)$_3$]

```
103
 Energy=  -1879.3289777960
La  -0.0019001   0.0195339  -0.3173804
 N  -2.0490777   1.0749951   0.7360069
 N  -1.5672897   1.7046745  -1.3795935
 C  -2.3652777   1.7994389  -0.3268924
 C  -3.5720382   2.6817681  -0.3386095
 C  -3.5692251   3.8856317   0.3583371
 H  -2.6850887   4.1790051   0.9132929
 C  -4.6895677   4.7044294   0.3458457
 H  -4.6776639   5.6427660   0.8880854
 C  -5.8242893   4.3211197  -0.3573724
 H  -6.7005388   4.9586217  -0.3642807
 C  -5.8324725   3.1184287  -1.0519242
 H  -6.7156595   2.8144385  -1.6016503
 C  -4.7088045   2.3041374  -1.0459230
 H  -4.7103254   1.3681979  -1.5934932
 C  -2.9676558   0.8583621   1.8313570
 H  -3.7670578   1.6090791   1.8196390
 C  -3.6134871  -0.5183105   1.7022050
 H  -2.8406337  -1.2934406   1.6898283
 H  -4.2920534  -0.7200643   2.5351343
 H  -4.1768213  -0.5917082   0.7693878
 C  -2.2229569   0.9843800   3.1514810
 H  -1.7671437   1.9719138   3.2459878
 H  -2.8934973   0.8265363   3.9992725
 H  -1.4266410   0.2365198   3.2084522
 C  -1.6212993   2.6349319  -2.4850381
 H  -2.5532517   3.2124098  -2.4592411
 C  -1.5770862   1.8708190  -3.7995454
 H  -2.4146914   1.1742438  -3.8711155
 H  -1.6151024   2.5515680  -4.6530478
 H  -0.6500997   1.2945356  -3.8695228
 C  -0.4565129   3.6160917  -2.3886337
 H   0.4923680   3.0702090  -2.3898487
 H  -0.4538297   4.3155163  -3.2286251
 H  -0.5126519   4.1878940  -1.4598404
 N   0.1100831  -2.2820296   0.7360549
 N   1.9438475   1.2559175   0.7299500
 N  -0.6918063  -2.1774095  -1.3735778
 N   2.2429917   0.5163122  -1.3838647
 C  -0.3717907  -2.9163573  -0.3221537
 C   2.7342113   1.1456731  -0.3273762
 C  -0.5456919  -4.4011729  -0.3313866
 C   4.1217246   1.7023960  -0.3256734
 C  -1.5960513  -4.9899077   0.3651809
 C   5.1350265   1.0541605   0.3730741
 H  -2.2843468  -4.3641680   0.9223821
 H   4.9093197   0.1454728   0.9200079
 C  -1.7627792  -6.3675112   0.3474725
 C   6.4249594   1.5657965   0.3711634
 H  -2.5871434  -6.8177913   0.8881242
 H   7.2097172   1.0515869   0.9137353
 C  -0.8742866  -7.1673593  -0.3595006
 C   6.7088303   2.7356339  -0.3216174
 H  -1.0027066  -8.2432913  -0.3713052
 H   7.7152369   3.1373740  -0.3203550
 C   0.1800326  -6.5841403  -1.0503530
 C   5.6989085   3.3889948  -1.0159876
 H   0.8775455  -7.2040689  -1.6015263
 H   5.9149524   4.3030409  -1.5567867
 C   0.3407901  -5.2058207  -1.0397952
 C   4.4112292   2.8714822  -1.0216896
 H   1.1579936  -4.7476652  -1.5857305
 H   3.6231293   3.3755399  -1.5699367
 C   0.7573612  -2.9731732   1.8287618
 C   2.2328809   2.1513331   1.8275587
 H   0.5044029  -4.0402336   1.8149321
 H   3.2871367   2.4532450   1.8129555
 C   2.2724457  -2.8472607   1.6961604
 C   1.3807961   3.4113356   1.7041466
 H   2.5595450  -1.7908966   1.6847597
 H   0.3191635   3.1444186   1.6957063
 H   2.7874675  -3.3362633   2.5271098
 H   1.5583579   4.0960970   2.5375131
 H   2.6144055  -3.2975316   0.7617923
 H   1.6021901   3.9341340   0.7712030
 C   0.2802778  -2.3934685   3.1515224
 C   1.9638540   1.4442149   3.1470189
 H  -0.8029163  -2.4900132   3.2482231
 H   2.5790863   0.5469335   3.2378877
 H   0.7527912  -2.8988524   3.9968759
 H   2.1741174   2.0991452   3.9956340
 H   0.5324967  -1.3308357   3.2106827
 H   0.9139463   1.1429689   3.2063761
 C  -1.4799380  -2.6867804  -2.4734099
 C   3.0718237   0.0785708  -2.4847960
 H  -1.5173688  -3.7824317  -2.4472005
 H   4.0443292   0.5846845  -2.4614207
 C  -0.8476680  -2.2689209  -3.7922065
 C   2.3936815   0.4226577  -3.8021356
 H   0.1723485  -2.6501347  -3.8707449
 H   2.2230365   1.4981203  -3.8806349
 H  -1.4254715  -2.6398410  -4.6418689
 H   2.9990961   0.1019893  -4.6530069
 H  -0.8081466  -1.1781582  -3.8622020
 H   1.4247002  -0.0800858  -3.8697588
 C  -2.9097431  -2.1643693  -2.3674138
 C   3.3203563  -1.4233289  -2.3785436
 H  -2.9080603  -1.0697441  -2.3682705
 H   2.3664808  -1.9602619  -2.3758109
 H  -3.5233120  -2.5097012  -3.2035274
 H   3.9200621  -1.7882722  -3.2164074
 H  -3.3717202  -2.4976850  -1.4356545
 H   3.8411049  -1.6609654  -1.4484949
```

[Ce(iPr$_2$BA)$_3$]

103
Energy= -2322.7712945780

Ce	0.0018513	0.0056059	-0.3068285
N	-2.0229264	1.0095212	0.7286378
N	-1.4735372	1.7166667	-1.3425210
C	-2.3073954	1.7736890	-0.3151944
C	-3.5130974	2.6570417	-0.3343860
C	-3.5373928	3.8274049	0.4171517
H	-2.6765458	4.0919476	1.0210463
C	-4.6539388	4.6510962	0.3937442
H	-4.6626823	5.5641448	0.9775908
C	-5.7586078	4.3047586	-0.3735796
H	-6.6320672	4.9459273	-0.3890559
C	-5.7405379	3.1343078	-1.1210229
H	-6.6006146	2.8589172	-1.7202014
C	-4.6197295	2.3162457	-1.1052519
H	-4.6005018	1.4057780	-1.6939440
C	-2.9631620	0.7582539	1.7975264
H	-3.7925167	1.4748487	1.7592625
C	-3.5450466	-0.6448282	1.6525634
H	-2.7381812	-1.3844389	1.6568640
H	-4.2327134	-0.8771194	2.4698156
H	-4.0827983	-0.7413832	0.7070864
C	-2.2629718	0.9167776	3.1388285
H	-1.8536171	1.9232624	3.2453183
H	-2.9501325	0.7294265	3.9671438
H	-1.4362341	0.2049564	3.2173718
C	-1.4979621	2.6677227	-2.4307626
H	-2.3941106	3.2971604	-2.3735803
C	-1.5245244	1.9281047	-3.7602825
H	-2.4063915	1.2882578	-3.8305479
H	-1.5325198	2.6265918	-4.6002415
H	-0.6383784	1.2943197	-3.8552274
C	-0.2762597	3.5770105	-2.3407017
H	0.6378637	2.9756477	-2.3661892
H	-0.2470615	4.2874159	-3.1707654
H	-0.2826840	4.1362652	-1.4029463
N	0.1428204	-2.2510294	0.7273770
N	1.8873193	1.2536474	0.7252389
N	-0.7455539	-2.1267616	-1.3432811
N	2.2193705	0.4279956	-1.3484128
C	-0.3823384	-2.8777609	-0.3147161
C	2.6918164	1.1118771	-0.3174113
C	-0.5557723	-4.3624407	-0.3299741
C	4.0661342	1.6997782	-0.3321812
C	-1.5637967	-4.9589054	0.4205897
C	5.0868716	1.1208428	0.4148070
H	-2.2206126	-4.3389973	1.0206287
H	4.8781866	0.2402689	1.0121515
C	-1.7286655	-6.3366430	0.4014671
C	6.3633726	1.6649996	0.3953087
H	-2.5199561	-6.7930596	0.9846877
H	7.1545580	1.2047046	0.9756169
C	-0.8798131	-7.1288797	-0.3606543
C	6.6256879	2.7983172	-0.3634990
H	-1.0058541	-8.2050885	-0.3725750
H	7.6214664	3.2255452	-0.3757467
C	0.1310734	-6.5377585	-1.1074325
C	5.6080479	3.3825056	-1.1065924
H	0.7968949	-7.1517479	-1.7026550
H	5.8072694	4.2678112	-1.6991447
C	0.2889630	-5.1590929	-1.0960323
C	4.3342846	2.8320559	-1.0946680
H	1.0728760	-4.6948770	-1.6841022
H	3.5399062	3.2820331	-1.6797642
C	0.8242134	-2.9428993	1.7982814
C	2.1492638	2.1854431	1.7988063
H	0.6110406	-4.0179949	1.7610850
H	3.1890773	2.5323569	1.7646643
C	2.3317485	-2.7559145	1.6551330
C	1.2407351	3.4030156	1.6566515
H	2.5765000	-1.6890572	1.6596658
H	0.1926327	3.0874965	1.6596004
H	2.8722366	-3.2390949	2.4731291
H	1.3921679	4.1106259	2.4758649
H	2.6824157	-3.1759108	0.7101368
H	1.4330175	3.9168741	0.7125643
C	0.3385489	-2.4109983	3.1383425
C	1.9243832	1.4968827	3.1366137
H	-0.7387766	-2.5530960	3.2439758
H	2.5809345	0.6309890	3.2417537
H	0.8403595	-2.9145211	3.9678184
H	2.1111830	2.1800737	3.9683273
H	0.5479896	-1.3403494	3.2156885
H	0.8901634	1.1487475	3.2103824
C	-1.5601606	-2.6211172	-2.4301335
C	3.0533279	-0.0320899	-2.4357623
H	-1.6617612	-3.7114263	-2.3715623
H	4.0470808	0.4283123	-2.3816683
C	-0.9055545	-2.2787348	-3.7603611
C	2.4246329	0.3569325	-3.7656003
H	0.0882890	-2.7253135	-3.8296419
H	2.3113772	1.4403502	-3.8387312
H	-1.5074838	-2.6347628	-4.5996652
H	3.0325833	0.0122081	-4.6052709
H	-0.7967231	-1.1948172	-3.8574863
H	1.4325524	-0.0938305	-3.8582661
C	-2.9558374	-2.0117353	-2.3401773
C	3.2284366	-1.5446503	-2.3407396
H	-2.8872417	-0.9197482	-2.3648679
H	2.2499440	-2.0343355	-2.3611732
H	-3.5868378	-2.3381619	-3.1707345
H	3.8258838	-1.9290981	-3.1713372
H	-3.4386073	-2.2954526	-1.4028402
H	3.7188341	-1.8158424	-1.4036407

[Pr(*i*Pr_2BA)$_3$]

```
103
 Energy=  -2364.6692304470
Pr  -0.0173037  -0.0136121  -0.3379729
 N  -2.0143967   0.9862637   0.6994306
 N  -1.4571555   1.7171916  -1.3585213
 C  -2.2897277   1.7716525  -0.3319123
 C  -3.4803561   2.6748419  -0.3320848
 C  -3.4771620   3.8350553   0.4353671
 H  -2.6059216   4.0766551   1.0339895
 C  -4.5797736   4.6775916   0.4346414
 H  -4.5673954   5.5823516   1.0311874
 C  -5.6976740   4.3603055  -0.3260710
 H  -6.5605651   5.0158014  -0.3234071
 C  -5.7065570   3.2004745  -1.0900847
 H  -6.5771050   2.9479189  -1.6841775
 C  -4.5996939   2.3634194  -1.0968457
 H  -4.6021429   1.4605957  -1.6974641
 C  -2.9520549   0.7372456   1.7710660
 H  -3.8056261   1.4223008   1.7025463
 C  -3.4835894  -0.6890382   1.6662860
 H  -2.6525107  -1.4005126   1.7023361
 H  -4.1704162  -0.9192513   2.4846978
 H  -4.0083825  -0.8344406   0.7198695
 C  -2.2722069   0.9630998   3.1134833
 H  -1.9050506   1.9879736   3.1946832
 H  -2.9595235   0.7716387   3.9408002
 H  -1.4176589   0.2889569   3.2200525
 C  -1.4765858   2.6721726  -2.4428666
 H  -2.3526857   3.3270562  -2.3643272
 C  -1.5528721   1.9389463  -3.7742475
 H  -2.4572118   1.3296976  -3.8300720
 H  -1.5529157   2.6404725  -4.6117497
 H  -0.6909204   1.2755781  -3.8873667
 C  -0.2271350   3.5444762  -2.3742741
 H   0.6674685   2.9156788  -2.4143472
 H  -0.1913773   4.2539107  -3.2048841
 H  -0.2012041   4.1032930  -1.4366284
 N   0.2224670  -2.2504726   0.6804799
 N   1.8202677   1.2609931   0.6964767
 N  -0.6883200  -2.1419832  -1.3781048
 N   2.1779694   0.4262543  -1.3676209
 C  -0.3271318  -2.8852122  -0.3420741
 C   2.6382878   1.1142785  -0.3344218
 C  -0.5319699  -4.3654788  -0.3279726
 C   4.0143235   1.6975350  -0.3325021
 C  -1.5363598  -4.9273844   0.4533662
 C   5.0286193   1.0995995   0.4081656
 H  -2.1709794  -4.2832264   1.0517716
 H   4.8129460   0.2088020   0.9876631
 C  -1.7252530  -6.3020757   0.4678532
 C   6.3078124   1.6377147   0.4046487
 H  -2.5128298  -6.7317030   1.0759117
 H   7.0939619   1.1631123   0.9802741
 C  -0.9042100  -7.1255067  -0.2916477
 C   6.5792999   2.7830149  -0.3326725
 H  -1.0481075  -8.1994301  -0.2764319
 H   7.5773294   3.2051463  -0.3330019
 C   0.1022029  -6.5687074  -1.0701884
 C   5.5679873   3.3857624  -1.0695560
 H   0.7462119  -7.2072373  -1.6635827
 H   5.7745042   4.2804717  -1.6452642
 C   0.2841482  -5.1931343  -1.0921641
 C   4.2911413   2.8421790  -1.0728224
 H   1.0652275  -4.7555972  -1.7039231
 H   3.5014500   3.3073973  -1.6523079
 C   0.8814492  -2.9305924   1.7718749
 C   2.0804005   2.1781154   1.7827994
 H   0.7321361  -4.0144123   1.6973208
 H   3.1016773   2.5723427   1.7176982
 C   2.3805020  -2.6551305   1.7107288
 C   1.1138098   3.3553779   1.6993949
 H   2.5617076  -1.5767142   1.7478779
 H   0.0822397   2.9913275   1.7255495
 H   2.9054610  -3.1252140   2.5461832
 H   1.2592988   4.0496457   2.5309900
 H   2.8016447  -3.0331401   0.7769450
 H   1.2527720   3.8990944   0.7627594
 C   0.2988354  -2.4638530   3.0981102
 C   1.9384902   1.4546368   3.1138812
 H  -0.7706974  -2.6775353   3.1484245
 H   2.6429482   0.6232598   3.1806452
 H   0.7922437  -2.9540798   3.9406056
 H   2.1180498   2.1321211   3.9519395
 H   0.4334253  -1.3841761   3.2076555
 H   0.9276545   1.0501763   3.2154901
 C  -1.5211842  -2.6373477  -2.4507578
 C   3.0205981  -0.0236104  -2.4523579
 H  -1.6363294  -3.7253592  -2.3776880
 H   4.0166013   0.4293332  -2.3796646
 C  -0.8752483  -2.3197574  -3.7913548
 C   2.4113986   0.3888747  -3.7842831
 H   0.1114664  -2.7809677  -3.8651021
 H   2.3123151   1.4743438  -3.8460312
 H  -1.4906283  -2.6769742  -4.6203375
 H   3.0246072   0.0466403  -4.6211981
 H  -0.7516639  -1.2387503  -3.9022389
 H   1.4149644  -0.0482269  -3.8930343
 C  -2.9082717  -2.0092402  -2.3555241
 C   3.1839482  -1.5384556  -2.3763419
 H  -2.8274402  -0.9183391  -2.3945348
 H   2.2019071  -2.0198062  -2.4087821
 H  -3.5507584  -2.3371600  -3.1765648
 H   3.7847641  -1.9160649  -3.2076829
 H  -3.3865217  -2.2761818  -1.4109286
 H   3.6658208  -1.8250737  -1.4393739
```

[Nd(*i*Pr$_2$BA)$_3$]

```
103
 Energy=  -2409.0783950800
Nd  -0.0108815   0.0031120  -0.3481554
 N  -2.0025204   0.9731774   0.7067252
 N  -1.4910541   1.6563182  -1.3815116
 C  -2.2985796   1.7360885  -0.3343656
 C  -3.4846715   2.6450623  -0.3297026
 C  -3.4683816   3.8145707   0.4232787
 H  -2.5908315   4.0585409   1.0115966
 C  -4.5658394   4.6638375   0.4204457
 H  -4.5429921   5.5763230   1.0047841
 C  -5.6917397   4.3438224  -0.3272229
 H  -6.5502600   5.0050344  -0.3266668
 C  -5.7140322   3.1742563  -1.0760055
 H  -6.5908032   2.9195411  -1.6599247
 C  -4.6122011   2.3307190  -1.0812457
 H  -4.6239920   1.4209959  -1.6712294
 C  -2.9264929   0.7403736   1.7940410
 H  -3.7688929   1.4402404   1.7387803
 C  -3.4834876  -0.6766100   1.6958755
 H  -2.6626048  -1.4000545   1.7144829
 H  -4.1599548  -0.8970859   2.5256258
 H  -4.0270489  -0.8106354   0.7582711
 C  -2.2221435   0.9537422   3.1251366
 H  -1.8326335   1.9708437   3.1996189
 H  -2.9015791   0.7779390   3.9623329
 H  -1.3814678   0.2613311   3.2201500
 C  -1.5139937   2.6131642  -2.4654086
 H  -2.4060966   3.2471015  -2.3993289
 C  -1.5527662   1.8816037  -3.7985100
 H  -2.4379354   1.2462244  -3.8668506
 H  -1.5633468   2.5856914  -4.6337072
 H  -0.6701936   1.2450096  -3.9036496
 C  -0.2867933   3.5153403  -2.3764274
 H   0.6235222   2.9087091  -2.4068852
 H  -0.2567523   4.2289306  -3.2037526
 H  -0.2871048   4.0712770  -1.4365328
 N   0.1650920  -2.2062200   0.7047768
 N   1.8272745   1.2436751   0.7082327
 N  -0.6871469  -2.1127836  -1.3823713
 N   2.1630335   0.4577380  -1.3806141
 C  -0.3455345  -2.8481098  -0.3348909
 C   2.6362443   1.1146556  -0.3322216
 C  -0.5300586  -4.3311193  -0.3278035
 C   4.0187371   1.6823751  -0.3248742
 C  -1.5547184  -4.9064774   0.4165675
 C   5.0260710   1.0646440   0.4090751
 H  -2.2140895  -4.2713803   0.9976571
 H   4.8000406   0.1699718   0.9785615
 C  -1.7336665  -6.2825906   0.4140094
 C   6.3109726   1.5888753   0.4127047
 H  -2.5383704  -6.7227426   0.9913572
 H   7.0916014   1.0988444   0.9828816
 C  -0.8821336  -7.0939179  -0.3246944
 C   6.5951123   2.7402353  -0.3102894
 H  -1.0196509  -8.1687874  -0.3241969
 H   7.5974498   3.1519854  -0.3044176
 C   0.1462107  -6.5237583  -1.0639411
 C   5.5910005   3.3622963  -1.0409533
 H   0.8144341  -7.1528488  -1.6403974
 H   5.8075354   4.2616411  -1.6056413
 C   0.3182174  -5.1467688  -1.0694401
 C   4.3085070   2.8323162  -1.0518954
 H   1.1150997  -4.6985304  -1.6523846
 H   3.5243927   3.3123103  -1.6268299
 C   0.8348602  -2.8859182   1.7910121
 C   2.0945936   2.1582679   1.7956693
 H   0.6535569  -3.9660689   1.7379697
 H   3.1244822   2.5305968   1.7398107
 C   2.3393794  -2.6546918   1.6876899
 C   1.1544190   3.3558048   1.6977389
 H   2.5512000  -1.5812713   1.7020498
 H   0.1151247   3.0138678   1.7165450
 H   2.8726894  -3.1259360   2.5172754
 H   1.3070205   4.0510505   2.5272633
 H   2.7257743  -3.0599537   0.7501653
 H   1.3135723   3.8921034   0.7599040
 C   0.3000137  -2.3828124   3.1230121
 C   1.9235894   1.4436101   3.1272769
 H  -0.7745173  -2.5592096   3.2014215
 H   2.6040029   0.5931505   3.2016935
 H   0.7971039  -2.8796196   3.9593973
 H   2.1168943   2.1192514   3.9637046
 H   0.4746379  -1.3077226   3.2154971
 H   0.9011357   1.0691474   3.2237778
 C  -1.5033878  -2.6195489  -2.4628908
 C   3.0046159   0.0006269  -2.4639563
 H  -1.5923673  -3.7104272  -2.3978486
 H   3.9967364   0.4630712  -2.4007139
 C  -0.8615103  -2.2775771  -3.7987336
 C   2.3862350   0.3919133  -3.7974193
 H   0.1366202  -2.7135505  -3.8726052
 H   2.2722775   1.4752588  -3.8693472
 H  -1.4655533  -2.6456417  -4.6312538
 H   3.0021073   0.0498066  -4.6323542
 H  -0.7660320  -1.1934113  -3.9033415
 H   1.3960958  -0.0603166  -3.8997376
 C  -2.9054023  -2.0255282  -2.3664645
 C   3.1823247  -1.5118465  -2.3708630
 H  -2.8490338  -0.9330542  -2.3973401
 H   2.2046617  -2.0026071  -2.3954478
 H  -3.5387826  -2.3640369  -3.1903943
 H   3.7843014  -1.8936539  -3.1994482
 H  -3.3781779  -2.3097492  -1.4240209
 H   3.6696277  -1.7834391  -1.4321461
```

[Pm(*i*Pr$_2$BA)$_3$]

103				C	-0.7913190	-7.0628040	-0.3235243
Energy= -2454.1199142230				C	6.6487938	2.6101857	-0.3083771
Pm	-0.0318272	0.0533144	-0.3422202	H	-0.9102706	-8.1492829	-0.3175661
N	-2.0334757	0.9654279	0.6961188	H	7.6689785	3.0024071	-0.3052745
N	-1.5017089	1.6868071	-1.3767017	C	0.1769048	-6.4695021	-1.1354040
C	-2.3272096	1.7437880	-0.3383238	C	5.6753624	3.1992595	-1.1171162
C	-3.5284659	2.6397949	-0.3354080	H	0.8189166	-7.0902634	-1.7656033
C	-3.5609905	3.7754378	0.4812450	H	5.9310598	4.0547732	-1.7474989
H	-2.7025493	4.0048530	1.1185527	C	0.3263145	-5.0830254	-1.1458970
C	-4.6782510	4.6099815	0.4800012	C	4.3749701	2.6955197	-1.1253648
H	-4.6936798	5.4987966	1.1159655	H	1.0810111	-4.6146198	-1.7837390
C	-5.7754674	4.3091409	-0.3288000	H	3.6117585	3.1508229	-1.7625598
H	-6.6529110	4.9608286	-0.3259858	C	0.8978424	-2.8038724	1.7755475
C	-5.7496033	3.1744199	-1.1413515	C	2.0775362	2.1616775	1.7891519
H	-6.6072811	2.9343981	-1.7749291	H	0.7572551	-3.8993031	1.7143238
C	-4.6279965	2.3458248	-1.1492514	H	3.1181728	2.5334518	1.7390800
H	-4.6022035	1.4592073	-1.7887949	C	2.3961429	-2.5195317	1.6720164
C	-2.9530975	0.6877699	1.7767550	C	1.1459317	3.3656469	1.6448894
H	-3.8256360	1.3657660	1.7246224	H	2.5709280	-1.4306555	1.6822282
C	-3.4684451	-0.7464254	1.6570690	H	0.0941274	3.0324844	1.6636128
H	-2.6203953	-1.4520651	1.6665091	H	2.9527821	-2.9749878	2.5066764
H	-4.1468290	-1.0032291	2.4862674	H	1.2953596	4.0937486	2.4581736
H	-4.0099195	-0.8839785	0.7086578	H	2.7989481	-2.9156697	0.7274752
C	-2.2636626	0.9155575	3.1172941	H	1.3201934	3.8754813	0.6849290
H	-1.9044837	1.9525565	3.2004436	C	0.3402190	-2.3374333	3.1155738
H	-2.9456776	0.7128473	3.9576903	C	1.8900245	1.4817015	3.1402482
H	-1.3937282	0.2465315	3.2156704	H	-0.7358420	-2.5564927	3.1887995
C	-1.4948478	2.6564740	-2.4494742	H	2.5731723	0.6256236	3.2461142
H	-2.3884781	3.3053524	-2.3876800	H	0.8549388	-2.8291012	3.9557930
C	-1.5267927	1.9438120	-3.7967358	H	2.0767650	2.1825020	3.9688118
H	-2.4240380	1.3124969	-3.8837894	H	0.4731365	-1.2493247	3.2242337
H	-1.5233187	2.6636239	-4.6299876	H	0.8586586	1.1064587	3.2376428
H	-0.6435171	1.2937972	-3.9045738	C	-1.5224232	-2.5472868	-2.4563113
C	-0.2600199	3.5495367	-2.3263949	C	2.9673039	-0.0992308	-2.4451711
H	0.6551249	2.9335632	-2.3535085	H	-1.6206741	-3.6472104	-2.3927107
H	-0.2093810	4.2851536	-3.1448977	H	3.9916612	0.3126321	-2.3778735
H	-0.2707634	4.0933867	-1.3694257	C	-0.9010813	-2.2125144	-3.8072927
N	0.2014771	-2.1445854	0.6930758	C	2.3783697	0.3331263	-3.7833594
N	1.8267109	1.2217740	0.7191246	H	0.0993152	-2.6613282	-3.9003544
N	-0.6836463	-2.0533602	-1.3867812	H	2.3216189	1.4303605	-3.8487600
N	2.1505391	0.4019278	-1.3623333	H	-1.5267415	-2.5785878	-4.6362020
C	-0.3185649	-2.7900511	-0.3438398	H	2.9840370	-0.0363498	-4.6255492
C	2.6377588	1.0643720	-0.3201677	H	-0.7945694	-1.1212819	-3.9174225
C	-0.4856617	-4.2798927	-0.3376030	H	1.3578418	-0.0665863	-3.8965946
C	4.0356576	1.6056524	-0.3161902	C	-2.9228198	-1.9467054	-2.3302062
C	-1.4523292	-4.8794633	0.4771734	C	3.0691055	-1.6214477	-2.3515611
C	5.0136529	1.0216416	0.4963847	H	-2.8634399	-0.8453354	-2.3588977
H	-2.0863119	-4.2524555	1.1101560	H	2.0596765	-2.0651205	-2.3676530
H	4.7485973	0.1700662	1.1290724	H	-3.5828027	-2.2831908	-3.1457285
C	-1.6074172	-6.2653670	0.4806338	H	3.6525367	-2.0397497	-3.1873389
C	6.3165614	1.5187737	0.4960296	H	-3.3803579	-2.2332069	-1.3709308
H	-2.3681275	-6.7257733	1.1163014	H	3.5509720	-1.9177658	-1.4075072
H	7.0763297	1.0530977	1.1289399				

[Sm(*i*Pr$_2$BA)$_3$]

```
103
 Energy=  -2505.7001124890
Sm  -0.0008818  -0.0676247  -0.3437470
 N  -1.9890358   0.8845685   0.6590390
 N  -1.3868554   1.6348224  -1.3803349
 C  -2.2253883   1.6990284  -0.3576714
 C  -3.3820681   2.6453078  -0.3440370
 C  -3.3102290   3.8265602   0.3868722
 H  -2.4066237   4.0593307   0.9391810
 C  -4.3876979   4.7007503   0.4109533
 H  -4.3228281   5.6206449   0.9803366
 C  -5.5477796   4.3953391  -0.2890272
 H  -6.3912784   5.0751848  -0.2658016
 C  -5.6237884   3.2158261  -1.0183200
 H  -6.5273583   2.9723785  -1.5650043
 C  -4.5428803   2.3458828  -1.0491820
 H  -4.5993288   1.4259124  -1.6204143
 C  -2.9256679   0.6799980   1.7403736
 H  -3.7496957   1.4006300   1.6764335
 C  -3.5168159  -0.7228750   1.6465073
 H  -2.7137839  -1.4659584   1.6743148
 H  -4.2021227  -0.9221935   2.4742907
 H  -4.0581508  -0.8496884   0.7070010
 C  -2.2270724   0.8800744   3.0772436
 H  -1.8155978   1.8881343   3.1540115
 H  -2.9168691   0.7208284   3.9093533
 H  -1.4024707   0.1692000   3.1794371
 C  -1.3877168   2.6031761  -2.4534406
 H  -2.2715840   3.2484979  -2.3844960
 C  -1.4328107   1.8855946  -3.7942811
 H  -2.3266052   1.2631381  -3.8700036
 H  -1.4324666   2.5980028  -4.6225560
 H  -0.5588881   1.2381666  -3.9027160
 C  -0.1492561   3.4868470  -2.3507487
 H   0.7505720   2.8650128  -2.3660569
 H  -0.0977141   4.1969410  -3.1799828
 H  -0.1538053   4.0454230  -1.4128107
 N   0.2387568  -2.2441049   0.6587137
 N   1.7406851   1.2663523   0.6989371
 N  -0.7136185  -2.1247495  -1.3800955
 N   2.1569646   0.3874872  -1.3371516
 C  -0.3389093  -2.8734691  -0.3538604
 C   2.5786108   1.1173844  -0.3154991
 C  -0.5602656  -4.3506387  -0.3347619
 C   3.9355196   1.7436006  -0.3138360
 C  -1.5656713  -4.8969583   0.4561806
 C   4.9742653   1.1674192   0.4096666
 H  -2.1868523  -4.2431539   1.0581627
 H   4.7915531   0.2636343   0.9802603
 C  -1.7729034  -6.2688927   0.4747900
 C   6.2368295   1.7435342   0.3999582
 H  -2.5611263  -6.6866776   1.0901574
 H   7.0425276   1.2857670   0.9619856
 C  -0.9696320  -7.1045813  -0.2902597
 C   6.4668446   2.9046626  -0.3265155
 H  -1.1284281  -8.1763409  -0.2724001
 H   7.4519309   3.3560960  -0.3319140
 C   0.0378588  -6.5630218  -1.0780383
 C   5.4308736   3.4850928  -1.0468562
 H   0.6679097  -7.2112761  -1.6757883
 H   5.6051289   4.3914736  -1.6149133
 C   0.2386870  -5.1901496  -1.1039557
 C   4.1707371   2.9038725  -1.0441092
 H   1.0205789  -4.7645762  -1.7230004
 H   3.3622550   3.3516712  -1.6112492
 C   0.9187716  -2.9310139   1.7332778
 C   1.9778415   2.1985674   1.7775794
 H   0.7470861  -4.0118179   1.6664091
 H   2.9905667   2.6132781   1.7087833
 C   2.4197408  -2.6827409   1.6264738
 C   0.9877150   3.3543360   1.6842004
 H   2.6200297  -1.6072643   1.6500227
 H  -0.0348882   2.9665312   1.6982001
 H   2.9600324  -3.1554323   2.4504798
 H   1.1092221   4.0505532   2.5179068
 H   2.8074030  -3.0750420   0.6843863
 H   1.1251105   3.9016875   0.7497059
 C   0.3844027  -2.4486122   3.0737457
 C   1.8531185   1.4827249   3.1144571
 H  -0.6875771  -2.6389641   3.1552969
 H   2.5704409   0.6624032   3.1820429
 H   0.8915409  -2.9479643   3.9025454
 H   2.0280637   2.1687538   3.9465936
 H   0.5461401  -1.3721488   3.1757448
 H   0.8491715   1.0646811   3.2239890
 C  -1.5699954  -2.6091367  -2.4392324
 C   3.0228953  -0.0426728  -2.4114682
 H  -1.6807643  -3.6979375  -2.3737165
 H   4.0025724   0.4437229  -2.3349181
 C  -0.9524952  -2.2763960  -3.7891670
 C   2.4113890   0.3358484  -3.7524267
 H   0.0343460  -2.7330835  -3.8866308
 H   2.2733437   1.4161973  -3.8259900
 H  -1.5833446  -2.6291939  -4.6082909
 H   3.0444161   0.0082683  -4.5804146
 H  -0.8363010  -1.1941177  -3.8916649
 H   1.4328117  -0.1389391  -3.8647416
 C  -2.9548831  -1.9841564  -2.3059407
 C   3.2346125  -1.5504963  -2.3224323
 H  -2.8738415  -0.8930849  -2.3286611
 H   2.2682100  -2.0626320  -2.3587111
 H  -3.6131803  -2.3009572  -3.1187178
 H   3.8525496  -1.9146406  -3.1471401
 H  -3.4132457  -2.2661643  -1.3560203
 H   3.7184056  -1.8152627  -1.3803035
```

[Eu(*i*Pr$_2$BA)$_3$]

103				C	2.7008716	10.3542575	10.4669508
Energy=	-2558.0832556200			H	2.5914969	11.4412101	10.4320971
Eu	5.9634571	12.6917095	11.7333925	H	2.4292509	10.0189678	11.4697857
N	7.3723907	13.3521472	9.8817789	H	1.9989857	9.9191483	9.7517357
N	5.2552902	14.1255978	9.9292342	C	4.5070304	10.4131108	8.7266962
N	5.0389194	10.5638491	11.0895208	H	4.4891955	11.5055230	8.6698536
C	6.4295834	14.0903410	9.3168104	H	3.8113222	10.0132286	7.9847307
C	6.6766098	14.8444567	8.0507719	H	5.5157457	10.0808502	8.4735548
C	7.4497306	16.0007245	8.0617328	N	6.8786409	10.5645153	12.3953956
H	7.8717048	16.3523113	8.9966756	C	7.7844963	9.9659012	13.3502051
C	7.6798046	16.6994875	6.8850733	H	7.7094376	8.8726875	13.3171088
H	8.2789421	17.6024251	6.9026050	C	9.2174854	10.3515761	13.0153163
C	7.1466961	16.2407623	5.6873866	H	9.3311568	11.4382468	13.0504682
H	7.3304544	16.7835119	4.7676581	H	9.4856135	10.0157898	12.0117267
C	6.3774182	15.0844549	5.6717385	H	9.9192783	9.9133073	13.7286949
H	5.9604034	14.7217978	4.7394553	C	7.4144318	10.4226198	14.7576102
C	6.1387507	14.3919487	6.8503641	H	7.4420632	11.5150744	14.8117477
H	5.5332659	13.4923801	6.8418945	H	8.1063892	10.0184283	15.5007677
C	8.6029088	13.0034594	9.2077009	H	6.4027221	10.1001273	15.0113421
H	8.7045808	13.5728945	8.2763040	N	4.5543880	13.3739494	13.5760734
C	8.5871748	11.5199138	8.8538292	N	6.6711870	14.1470051	13.5171084
H	8.4494664	10.9243425	9.7612044	C	5.4951954	14.1240513	14.1281150
H	9.5213508	11.2151207	8.3751631	C	5.2461572	14.9051258	15.3770932
H	7.7597672	11.2962102	8.1775838	C	4.4488537	16.0445554	15.3454082
C	9.7934068	13.3344476	10.0947973	H	4.0068691	16.3611299	14.4072089
H	9.8117011	14.3981707	10.3397299	C	4.2217972	16.7720150	16.5051009
H	10.7338322	13.0737672	9.6036695	H	3.6043476	17.6620368	16.4710333
H	9.7317515	12.7724890	11.0305307	C	4.7817338	16.3585463	17.7070193
C	4.2268258	15.0858112	9.5952380	H	4.6009145	16.9239833	18.6135643
H	4.4804462	15.6090097	8.6657224	C	5.5747707	15.2188709	17.7437107
C	2.9001193	14.3716506	9.3868557	H	6.0133269	14.8919053	18.6792951
H	2.9776416	13.6348349	8.5851464	C	5.8104458	14.4979840	16.5816699
H	2.1077335	15.0800587	9.1343473	H	6.4356060	13.6123068	16.6059054
H	2.6081808	13.8479685	10.3008889	C	3.3232862	13.0336683	14.2532946
C	4.1144220	16.1258201	10.7052690	H	3.2217411	13.6114594	15.1795248
H	3.9082083	15.6296202	11.6583436	C	3.3379986	11.5532073	14.6199323
H	3.3107593	16.8383582	10.5027352	H	3.4793236	10.9501659	13.7180759
H	5.0506608	16.6776000	10.8099624	H	2.4019364	11.2524102	15.0974043
C	5.9579871	9.8688192	11.7441222	H	4.1627351	11.3354154	15.3013450
C	5.9545201	8.3750541	11.7479853	C	2.1338009	13.3574660	13.3620752
C	6.8897473	7.6693607	10.9981998	H	2.1145092	14.4195105	13.1098968
H	7.6247851	8.2112978	10.4135812	H	1.1926605	13.0987420	13.8528335
C	6.8820502	6.2816899	10.9975652	H	2.1984110	12.7894661	12.4302707
H	7.6111464	5.7388746	10.4073997	C	7.6947015	15.1212605	13.8249192
C	5.9437466	5.5903431	11.7529726	H	7.4424852	15.6626522	14.7442268
H	5.9390752	4.5067363	11.7543218	C	9.0269318	14.4199815	14.0411549
C	5.0120395	6.2915703	12.5074600	H	8.9584893	13.7004473	14.8592809
H	4.2793360	5.7565644	13.1002811	H	9.8161132	15.1388037	14.2735213
C	5.0152088	7.6791903	12.5019446	H	9.3171166	13.8778936	13.1373092
H	4.2853333	8.2289955	13.0856859	C	7.7936383	16.1381987	12.6923209
C	4.1332029	9.9635024	10.1354090	H	8.0052073	15.6243078	11.7498498
H	4.2052158	8.8702799	10.1732150	H	8.5882869	16.8651154	12.8786819
				H	6.8503069	16.6755900	12.5769954

[Yb(iPr$_2$BA)$_3$]

103
Energy = -3007.0013039630

Yb	-0.0046230	-0.0021091	0.0014841
N	-2.0921706	-0.1619449	1.0075533
N	-1.9273847	0.8203910	-1.0084562
C	-2.7003351	0.4462475	0.0001368
C	-4.1720944	0.6965546	0.0021841
C	-4.7084266	1.7127309	0.7859856
H	-4.0502967	2.3214294	1.3959151
C	-6.0762197	1.9466179	0.7870471
H	-6.4863211	2.7433637	1.3965763
C	-6.9176072	1.1595146	0.0114895
H	-7.9861452	1.3395839	0.0152073
C	-6.3859272	0.1409754	-0.7688057
H	-7.0387370	-0.4771392	-1.3740188
C	-5.0170428	-0.0864776	-0.7772508
H	-4.5992228	-0.8785819	-1.3885486
C	-2.8109831	-0.8204629	2.0756919
H	-3.8878486	-0.6449568	1.9715712
C	-2.5686337	-2.3236766	2.0052391
H	-1.4947661	-2.5260233	2.0464069
H	-3.0550857	-2.8428831	2.8347755
H	-2.9509531	-2.7303332	1.0673682
C	-2.3722933	-0.2656286	3.4225721
H	-2.5582875	0.8084807	3.4804024
H	-2.9051010	-0.7556740	4.2408932
H	-1.3019544	-0.4325882	3.5649858
C	-2.3900804	1.6828636	-2.0730741
H	-3.4652686	1.8686445	-1.9691458
C	-2.1558471	1.0206384	-3.4225399
H	-2.6852725	0.0680967	-3.4856746
H	-2.4957541	1.6626472	-4.2385829
H	-1.0900853	0.8258159	-3.5638390
C	-1.6697623	3.0238890	-1.9950237
H	-0.5886007	2.8640406	-2.0332377
H	-1.9565908	3.6773751	-2.8226338
H	-1.9012262	3.5287992	-1.0556847
N	1.1793549	-1.7279569	1.0118564
N	0.9105538	1.8757657	1.0144180
N	0.2403192	-2.0798762	-1.0035103
N	1.6751074	1.2499970	-1.0082105
C	0.9577146	-2.5578353	0.0028841
C	1.7353643	2.1042732	0.0027413
C	1.4885333	-3.9531226	-0.0010288
C	2.6811110	3.2593320	0.0021821
C	0.8802633	-4.9367856	0.7716317
C	3.8403723	3.2147613	0.7694034
H	0.0177849	-4.6833670	1.3779414
H	4.0500990	2.3372485	1.3707739
C	1.3711206	-6.2347624	0.7634030
C	4.7248911	4.2839866	0.7617817
H	0.8884893	-6.9973385	1.3634561
H	5.6292822	4.2395599	1.3572781
C	2.4797402	-6.5549828	-0.0095338
C	4.4504029	5.4090685	-0.0049007
H	2.8656087	-7.5675591	-0.0126989
H	5.1390932	6.2456681	-0.0078036
C	3.0925807	-5.5739694	-0.7783311
C	3.2909581	5.4590530	-0.7679316
H	3.9588201	-5.8186299	-1.3818663
H	3.0717882	6.3353747	-1.3666995
C	2.5954137	-4.2784195	-0.7779615
C	2.4117670	4.3854413	-0.7684211
H	3.0688558	-3.5121139	-1.3815633
H	1.5091630	4.4195571	-1.3683070
C	2.1167969	-2.0197771	2.0738205
C	0.6958930	2.8297677	2.0800635
H	2.5047828	-3.0393000	1.9676939
H	1.3924691	3.6702023	1.9814356
C	3.2944965	-1.0555844	1.9939119
C	-0.7230119	3.3803574	1.9968153
H	2.9301521	-0.0254393	2.0344523
H	-1.4404261	2.5557476	2.0305337
H	3.9934194	-1.2132805	2.8191682
H	-0.9334509	4.0618045	2.8248974
H	3.8317341	-1.1844274	1.0527482
H	-0.8719329	3.9162911	1.0579256
C	1.4253184	-1.9185656	3.4251363
C	0.9417768	2.1711086	3.4290406
H	0.5911738	-2.6199222	3.4888983
H	1.9624921	1.7900184	3.4959834
H	2.1223233	-2.1319633	4.2389741
H	0.7800453	2.8785297	4.2457768
H	1.0315736	-0.9097105	3.5694414
H	0.2567756	1.3310340	3.5655543
C	-0.2640147	-2.9135196	-2.0722461
C	2.6501096	1.2303178	-2.0763988
H	0.1147855	-3.9364229	-1.9654557
H	3.3442273	2.0720308	-1.9721668
C	0.2016776	-2.3780175	-3.4179155
C	1.9546154	1.3587996	-3.4232144
H	1.2917370	-2.3570568	-3.4722558
H	1.3875128	2.2897235	-3.4814764
H	-0.1756263	-2.9944227	-4.2372804
H	2.6785514	1.3406660	-4.2412478
H	-0.1645063	-1.3586889	-3.5619812
H	1.2588577	0.5285795	-3.5658078
C	-1.7859221	-2.9641442	-2.0069441
C	3.4583123	-0.0599862	-2.0057745
H	-2.1903709	-1.9490673	-2.0493444
H	2.7834729	-0.9194997	-2.0459860
H	-2.1992093	-3.5410251	-2.8380367
H	4.1658646	-0.1308457	-2.8356460
H	-2.1148435	-3.4173976	-1.0702653
H	4.0141055	-0.1137057	-1.0681489

[Lu(*i*Pr$_2$BA)$_3$]

103			
Energy =	-3083.9505749930		
Lu	-0.0002788	0.0005692	0.0009459
N	-2.0762624	-0.1668859	1.0087777
N	-1.9130348	0.8340752	-0.9983917
C	-2.6859019	0.4466722	0.0053993
C	-4.1591649	0.6880035	0.0044778
C	-4.7028626	1.7066924	0.7798636
H	-4.0499266	2.3223255	1.3883946
C	-6.0713634	1.9364804	0.7719192
H	-6.4871082	2.7357753	1.3742428
C	-6.9060595	1.1430412	-0.0043504
H	-7.9750074	1.3206327	-0.0083790
C	-6.3671657	0.1208913	-0.7749081
H	-7.0146237	-0.5022704	-1.3806824
C	-4.9976950	-0.1030273	-0.7738466
H	-4.5742666	-0.8967895	-1.3790704
C	-2.7951392	-0.8308427	2.0738235
H	-3.8710530	-0.6466496	1.9751154
C	-2.5646996	-2.3351417	1.9900600
H	-1.4924091	-2.5463108	2.0255700
H	-3.0520523	-2.8571769	2.8172855
H	-2.9540513	-2.7312040	1.0506848
C	-2.3485070	-0.2908298	3.4241550
H	-2.5239721	0.7845430	3.4909230
H	-2.8848671	-0.7823371	4.2392540
H	-1.2798144	-0.4697426	3.5634185
C	-2.3824026	1.6920911	-2.0638855
H	-3.4598161	1.8656139	-1.9623470
C	-2.1383528	1.0336317	-3.4135172
H	-2.6550453	0.0742314	-3.4778552
H	-2.4863404	1.6717214	-4.2292288
H	-1.0699675	0.8536752	-3.5542218
C	-1.6782143	3.0414965	-1.9847059
H	-0.5951748	2.8952176	-2.0229461
H	-1.9730613	3.6913053	-2.8123962
H	-1.9161414	3.5435379	-1.0455185
N	1.1854888	-1.7147563	1.0030756
N	0.8944667	1.8832509	1.0031729
N	0.2313040	-2.0715308	-1.0018651
N	1.6759751	1.2389840	-1.0048038
C	0.9548899	-2.5487467	-0.0001679
C	1.7303614	2.1017173	-0.0010913
C	1.4804285	-3.9460335	-0.0011052
C	2.6792241	3.2543021	-0.0003954
C	0.8673466	-4.9249723	0.7737623
C	3.8423655	3.2028464	0.7605067
H	0.0056196	-4.6662372	1.3788699
H	4.0554640	2.3200075	1.3528047
C	1.3536417	-6.2246429	0.7710226
C	4.7252625	4.2734346	0.7606620
H	0.8679187	-6.9830751	1.3738144
H	5.6317386	4.2243066	1.3526057
C	2.4615954	-6.5517732	0.0000668
C	4.4463394	5.4059437	0.0066446
H	2.8436939	-7.5657779	0.0008255
H	5.1337448	6.2435979	0.0098900
C	3.0783621	-5.5758916	-0.7720976
C	3.2842481	5.4617972	-0.7518810
H	3.9439188	-5.8260251	-1.3743632
H	3.0617175	6.3437513	-1.3410625
C	2.5866342	-4.2782376	-0.7760678
C	2.4056964	4.3876277	-0.7588413
H	3.0642376	-3.5157846	-1.3812610
H	1.4997559	4.4278045	-1.3532807
C	2.1217464	-2.0074418	2.0659169
C	0.6854610	2.8363366	2.0706955
H	2.5032689	-3.0296384	1.9622287
H	1.3869750	3.6727815	1.9728724
C	3.3061922	-1.0517202	1.9845537
C	-0.7299999	3.3958811	1.9903470
H	2.9500535	-0.0187218	2.0247030
H	-1.4529786	2.5762416	2.0248007
H	4.0036584	-1.2139784	2.8101411
H	-0.9339950	4.0777771	2.8196830
H	3.8427636	-1.1854201	1.0437889
H	-0.8771451	3.9339850	1.0524991
C	1.4321277	-1.8989932	3.4176860
C	0.9286043	2.1750333	3.4190747
H	0.5917849	-2.5927385	3.4828698
H	1.9465471	1.7863194	3.4843692
H	2.1280073	-2.1180988	4.2309607
H	0.7735797	2.8834791	4.2362393
H	1.0482812	-0.8863604	3.5617358
H	0.2371183	1.3404407	3.5563996
C	-0.2776855	-2.9054942	-2.0682641
C	2.6506182	1.2180559	-2.0732942
H	0.1055987	-3.9270631	-1.9643405
H	3.3398883	2.0641545	-1.9719882
C	0.1772132	-2.3687069	-3.4172057
C	1.9550206	1.3385900	-3.4209091
H	1.2667693	-2.3401688	-3.4778782
H	1.3800567	2.2645792	-3.4809326
H	-0.2005095	-2.9892484	-4.2332449
H	2.6799637	1.3255822	-4.2381507
H	-0.1974562	-1.3524856	-3.5610245
H	1.2670291	0.5019383	-3.5630184
C	-1.7989156	-2.9626052	-1.9943395
C	3.4668999	-0.0668574	-1.9992041
H	-2.2081946	-1.9494610	-2.0348806
H	2.7980381	-0.9311111	-2.0364270
H	-2.2143441	-3.5412357	-2.8231540
H	4.1738288	-0.1352670	-2.8297910
H	-2.1205787	-3.4174685	-1.0560596
H	4.0244133	-0.1139972	-1.0623260

Actinide complexes:

[Ac(*i*Pr$_2$BA)$_3$]

```
103
 Energy=  -2223.3556752250
Ac  -0.0702835   0.3022692  -0.0136692
 C  -6.8096559   0.2747931   0.6831836
 N  -2.4909973   0.4602527  -0.9437392
 H  -3.1635843   0.5638059  -4.2584622
 N  -2.3136748  -0.1686126   1.2281604
 H  -6.6639992  -2.8307990  -0.6585466
 H  -7.5233174   0.9947485   1.0666975
 H  -2.6676330  -2.8328672   3.3036129
 C  -3.0728039   0.0022516   0.1564447
 H  -1.5835125   0.6103975  -3.4688702
 C  -4.5318582  -0.3305406   0.1778238
 H  -5.1137729   1.5413629   1.0473289
 C  -4.9757171  -1.5589882  -0.3009314
 C  -2.6167426   0.2647546  -3.3613211
 H  -4.2582702  -2.2725651  -0.6905943
 C  -2.7945460  -0.8524412   2.4092170
 H  -1.2484690  -2.3626992   2.3490824
 H  -2.7437520  -2.8330748   1.5319292
 H  -3.8909464  -0.8421988   2.4431066
 C  -7.2481370  -0.9535616   0.2062650
 C  -2.2820672  -0.1348274   3.6493065
 H  -8.3043737  -1.1954491   0.2166506
 H  -2.6132653   0.9054467   3.6574939
 H   0.1905392  -3.2323276  -4.0029577
 C  -3.2480218   0.8580946  -2.1099369
 H  -2.6369368  -0.6215583   4.5606898
 H  -4.2790673   0.4892482  -2.0477884
 H  -2.6059748  -0.8256093  -3.3101117
 C  -3.3014723   2.3796266  -2.2033289
 H  -1.1881836  -0.1421577   3.6687547
 H  -2.2882414   2.7878676  -2.2673301
 C  -5.4566156   0.5840227   0.6711536
 H  -3.8590883   2.7034065  -3.0860916
 C  -2.3405186  -2.3096089   2.4012675
 H  -3.7788794   2.8027713  -1.3170534
 C  -6.3279354  -1.8705215  -0.2848753
 C   3.6633004   5.8488857  -1.1691795
 C   4.0709165  -4.9730411  -0.3822864
 C  -0.0033220   4.6289327   1.5302218
 C   3.5935521  -0.4133340   1.9824856
 C   4.0815777   3.4302507   0.1225017
 C   1.7128455  -4.6420930   1.0422263
 C   3.4152645   0.6030966  -2.3273207
 C  -1.1542193  -3.4929719  -1.6164082
 C   2.4203673   1.7427517  -2.5300231
 C   0.2874859  -3.0475875  -1.8433562
 C   0.7751072   2.8221465   3.0804041
 C   1.8416279  -1.3403622   3.5235478
 C   4.8937732   5.5818542  -0.5835896
 C   3.5899109  -6.0077845   0.4095422
 C   1.0302176   3.5306366   1.7585239
 C   2.5254311  -1.4861436   2.1712795
 C   1.6029389   1.5276857  -3.7950785
 C   0.4681349  -2.4923343  -3.2486784
 C   2.6450734   4.9074324  -1.1110624
 C   3.3711206  -3.7775231  -0.4656473
 C   2.8473406   3.6927716  -0.4638983
 C   2.1893423  -3.6046977   0.2475921
 C   1.7511010   2.6760539  -0.3960632
 C   1.4405827  -2.3121288   0.1617466
 C   5.1011365   4.3696089   0.0615994
 C   2.4084780  -5.8405769   1.1202582
 N   0.9880567   2.5621499   0.6845154
 N   1.5457198  -1.3945428   1.1113448
 N   1.5243939   1.8412106  -1.3974260
 N   0.6521969  -2.0431944  -0.8684206
 H   4.0311150   0.7834386  -1.4437590
 H  -1.2698701  -3.9262510  -0.6207719
 H   4.0746893   0.4930069  -3.1922255
 H  -1.4630670  -4.2347764  -2.3574021
 H   2.8810159  -0.3405191  -2.1786312
 H  -1.8279243  -2.6335390  -1.6880074
 H  -0.2048330   2.3337508   3.0699927
 H   1.3292633  -0.3761441   3.5901753
 H   0.8915739   2.3435023  -3.9376232
 H   1.5053239  -2.1962125  -3.4172805
 H   2.9925988   2.6707075  -2.6516912
 H   0.9296284  -3.9312522  -1.7467695
 H   0.7869559   3.5267894   3.9146473
 H   2.5658869  -1.3940529   4.3397725
 H   1.5345692   2.0595985   3.2652975
 H   1.0969807  -2.1259283   3.6664482
 H   5.6891250   6.3163597  -0.6300218
 H   4.1347371  -6.9423516   0.4727900
 H   2.0180761   4.0055242   1.8167724
 H   3.0252953  -2.4618559   2.1526079
 H   0.1985532   5.1594309   0.5977094
 H   4.1046529  -0.5460405   1.0265335
 H  -1.0036375   4.1919967   1.4604517
 H   3.1339773   0.5799664   1.9816409
 H   1.6870844   5.1138658  -1.5747486
 H   3.7429572  -2.9707477  -1.0875812
 H   4.2424230   2.4863987   0.6312932
 H   0.7937697  -4.5085241   1.6019128
 H   1.0382908   0.5930538  -3.7283034
 H  -0.1630035  -1.6110581  -3.3940101
 H   0.0021814   5.3541525   2.3482589
 H   4.3370856  -0.4464747   2.7828213
 H   6.0592258   4.1549473   0.5205855
 H   2.0282777  -6.6451045   1.7390200
 H   3.4955481   6.7927900  -1.6747383
 H   4.9930430  -5.0976457  -0.9380082
 H   2.2476184   1.4678149  -4.6749562
```

[Th(*i*Pr_2BA)$_3$]

103			
Energy=	-2255.3464549160		
Th	0.0015102	-0.0035868	1.0559424
C	-5.8076852	-3.0912457	1.7570304
N	-2.2108784	-0.7715766	0.2231171
H	-3.3058740	-1.0453264	-2.9793921
N	-1.3354129	-1.9051119	1.9439319
H	-4.5493118	-5.6964423	-0.0038589
H	-6.7142695	-2.7722016	2.2577985
H	0.0666006	-4.1217418	4.0644342
C	-2.3359741	-1.7634127	1.0899684
H	-1.6391715	-0.8780773	-2.3820318
C	-3.5295275	-2.6607478	1.1046767
H	-4.7357318	-1.2906349	2.2305692
C	-3.4815319	-3.9010803	0.4774325
C	-2.6408240	-1.2436476	-2.1351285
H	-2.5698158	-4.2159913	-0.0179044
C	-1.3591964	-2.8473332	3.0422619
H	0.7396438	-2.5965229	3.4621268
H	0.3889716	-3.9070699	2.3312145
H	-2.0346864	-3.6795417	2.8093668
C	-5.7577886	-4.3283001	1.1277835
C	-1.8562572	-2.1747934	4.3163873
H	-6.6255787	-4.9771895	1.1360442
H	-2.8800625	-1.8162939	4.1903922
H	2.5669425	-2.3480126	-2.9761795
C	-3.1354201	-0.5541868	-0.8690244
H	-1.8330742	-2.8655713	5.1629374
H	-4.1198389	-0.9668586	-0.6163623
H	-2.5801899	-2.3241547	-1.9883183
C	-3.2867878	0.9429570	-1.0920580
H	-1.2213652	-1.3140137	4.5483730
H	-2.3150400	1.3827467	-1.3364316
C	-4.6972213	-2.2592499	1.7447634
H	-3.9746789	1.1501435	-1.9147334
C	0.0412639	-3.4083181	3.2378400
H	-3.6582488	1.4334579	-0.1902483
C	-4.5931131	-4.7317189	0.4879233
C	-1.8292963	6.3276323	0.4999926
C	6.3981117	-1.5895446	0.4884322
C	-2.9576113	1.6622778	3.2460000
C	2.9345351	1.7406178	3.2306209
C	0.3781247	5.1986860	1.7478044
C	4.3206223	-2.9239896	1.7561719
C	2.4573437	2.3788630	-1.0991257
C	0.8369536	-3.3261879	-1.0882957
C	1.0828989	2.9911909	-0.8748815
C	2.0550389	-2.4422632	-0.8661805
C	-0.9380946	2.6851540	4.3240041
C	2.7949057	-0.5105437	4.3257565
C	-0.9078754	7.1408318	1.1468535
C	6.6418856	-2.7921536	1.1388298
C	-1.7708702	2.5939715	3.0508380
C	3.1396801	0.2443225	3.0474632
C	0.2379777	2.9038205	-2.1402280
C	2.4030583	-1.6711797	-2.1338974
C	-1.6490560	4.9518581	0.4791751
C	5.1181956	-1.0535336	0.4735367
C	-0.5451795	4.3816168	1.1041836
C	4.0740361	-1.7189032	1.1070394
C	-0.3551183	2.9004253	1.0900611
C	2.6963073	-1.1428711	1.0928789
C	0.1962457	6.5742122	1.7702031
C	5.6012751	-3.4581582	1.7730686
N	-0.9680170	2.1040083	1.9509037
N	2.3134227	-0.2101565	1.9498357
N	0.4364522	2.2973099	0.2185355
N	1.7775561	-1.5320006	0.2242654
H	3.0684243	2.4575722	-0.1978832
H	0.5978179	-3.8907091	-0.1851053
H	2.9782088	2.8729073	-1.9222763
H	1.0048389	-4.0276893	-1.9084861
H	2.3558799	1.3169814	-1.3433368
H	-0.0312278	-2.7078165	-1.3360503
H	-0.5087389	1.7046597	4.5523386
H	1.7309861	-0.3850071	4.5498859
H	-0.7297469	3.3881025	-1.9932326
H	3.3061496	-1.0747186	-1.9880596
H	1.2136077	4.0507121	-0.6226226
H	2.9066270	-3.0851600	-0.6115357
H	-1.5468688	3.0073425	5.1724770
H	3.3777334	-0.1406941	5.1730022
H	-0.1166246	3.3936745	4.1987232
H	2.9925165	-1.5782688	4.2095643
H	-1.0502099	8.2148794	1.1653756
H	7.6416868	-3.2097616	1.1515267
H	-2.1533609	3.5958650	2.8204458
H	4.1979399	0.0665411	2.8203745
H	-3.5634076	1.6116935	2.3393145
H	3.1988637	2.2820380	2.3203850
H	-2.6045211	0.6511266	3.4690380
H	1.8830313	1.9482811	3.4499108
H	-2.3705176	4.3146471	-0.0200001
H	4.9269307	-0.1119628	-0.0289977
H	1.2412274	4.7545675	2.2309274
H	3.5057777	-3.4446734	2.2466632
H	0.0575306	1.8527002	-2.3866170
H	1.5832501	-0.9902391	-2.3834929
H	-3.5876878	1.9969038	4.0729908
H	3.5396980	2.1219178	4.0560535
H	0.9186272	7.2049069	2.2750290
H	5.7866215	-4.3969715	2.2817134
H	-2.6919310	6.7653212	0.0113865
H	7.2069931	-1.0662492	-0.0078870
H	0.7399901	3.3819151	-2.9850857

[Pa(*i*Pr$_2$BA)$_3$]

103				C	0.4097421	3.3026514	1.6282996
Energy=	-2289.0363047390			C	2.7472342	-1.5308499	2.0410805
Pa	0.0906139	0.0106808	-0.0261583	C	1.8779148	1.6728941	-3.5763275
C	-6.3121189	1.2207297	0.8431777	C	0.7858876	-2.9733987	-3.0516382
N	-2.1180703	0.6333228	-0.8914694	C	1.8782957	4.7586084	-1.2850857
H	-3.2364280	0.8079178	-4.0911587	C	3.4040172	-3.9880733	-0.4859906
N	-2.0625357	-0.5380047	1.0161556	C	2.3649851	3.7281636	-0.4878494
H	-7.0103251	-1.6988376	-0.7250828	C	2.2520779	-3.7905501	0.2678055
H	-6.8050090	2.0607706	1.3184211	C	1.5848579	2.4633216	-0.3361356
H	-2.2784913	-2.9367459	3.3783460	C	1.5080405	-2.4983685	0.1780023
C	-2.7944631	0.0669468	0.0948039	C	4.3050904	5.0588053	0.0282430
H	-1.7897452	-0.0100561	-3.4596162	C	2.4820519	-6.0078733	1.1795811
C	-4.2858604	0.1091531	0.1653341	N	0.6893098	2.2987517	0.6239652
H	-4.3376870	1.9932904	1.1889654	N	1.7597171	-1.4800695	0.9844945
C	-5.0418368	-0.9262533	-0.3737448	N	1.7437544	1.4337904	-1.1518672
C	-2.8034489	0.3212617	-3.2136449	N	0.5546453	-2.2962150	-0.7167749
H	-4.5425254	-1.7653708	-0.8450785	H	3.9848493	-0.0134853	-1.4095870
C	-2.6264404	-1.1482177	2.2012194	H	-1.8016326	-3.5579232	-0.8290319
H	-0.8261930	-2.2531822	2.6238167	H	4.0877407	0.0959214	-3.1787886
H	-1.9926032	-3.1392404	1.6375164	H	-1.6930745	-4.0349111	-2.5362611
H	-3.6866373	-1.3772368	2.0379451	H	2.7016155	-0.7004395	-2.4101548
C	-7.0646005	0.1845961	0.3056844	H	-1.7241306	-2.3196764	-2.0856798
C	-2.5220863	-0.2026869	3.3914111	H	0.8400462	1.9743952	3.2668992
H	-8.1463894	0.2134844	0.3611151	H	1.3427923	-0.9266729	3.5567147
H	-3.0940951	0.7099824	3.2118125	H	1.3802825	2.6448321	-3.5677821
H	0.4492311	-3.6800917	-3.8142285	H	1.8755215	-3.0173021	-2.9962318
C	-2.7539497	1.2718552	-2.0238349	H	3.3754444	2.2626400	-2.1696925
H	-2.8973851	-0.6726872	4.3039000	H	0.5029049	-4.2803020	-1.3840979
H	-3.7824709	1.5543611	-1.7677224	H	0.8821605	3.6948710	3.7121529
H	-3.3997841	-0.5627435	-2.9783476	H	2.8001819	-1.7107144	4.2054529
C	-1.9824109	2.5358849	-2.3731771	H	2.2132454	2.9800084	2.7849925
H	-1.4757363	0.0785487	3.5455138	H	1.5379157	-2.6796403	3.4236797
H	-0.9453889	2.2829908	-2.6127021	H	4.3796399	7.0062228	-0.8736126
C	-4.9267127	1.1836500	0.7727493	H	4.1674732	-7.1427333	0.4847230
H	-2.4231636	3.0401609	-3.2360247	H	0.7550613	4.2850730	1.2835713
C	-1.8921669	-2.4515126	2.4792542	H	3.4322322	-2.3713283	1.8751222
H	-1.9709173	3.2279325	-1.5290623	H	-1.6091015	3.6339948	0.9211395
C	-6.4272887	-0.8881338	-0.3039932	H	4.0660410	-0.1180063	1.0666019
C	2.6008169	5.9355202	-1.4221306	H	-1.4676532	2.3987267	2.1754924
C	4.0920615	-5.1906099	-0.4068575	H	2.8928913	0.6150124	2.1645968
C	-1.0945056	3.3735859	1.8479606	H	0.9287445	4.6392834	-1.7947282
C	3.5573821	-0.2424812	2.0243033	H	3.7634474	-3.1959779	-1.1333480
C	3.5815409	3.8828971	0.1680484	H	3.9638107	3.0778992	0.7857165
C	1.7951480	-4.8044504	1.1029129	H	0.8958900	-4.6516685	1.6891044
C	3.4076388	0.1307241	-2.3247416	H	1.1097392	0.9006354	-3.6833084
C	-1.3564682	-3.3078480	-1.7938131	H	0.5032804	-1.9620537	-3.3600012
C	2.6484223	1.4490267	-2.2813380	H	-1.3465030	4.1136905	2.6107397
C	0.1623551	-3.2858095	-1.6973360	H	4.3031659	-0.2360150	2.8223592
C	1.1309232	2.9739862	2.9293717	H	5.2531168	5.1721027	0.5409499
C	2.0707822	-1.7270527	3.3918872	H	2.1188774	-6.7949726	1.8300133
C	3.8153499	6.0874452	-0.7659434	H	2.2145412	6.7355796	-2.0427665
C	3.6313660	-6.2029612	0.4247801	H	4.9896293	-5.3374415	-0.9961572
				H	2.5413635	1.6307760	-4.4436814

[U(iPr$_2$BA)$_3$]

103			
Energy=	-2324.6030029380		
U	6.5111766	5.4321153	3.8300985
N	5.0827018	4.7813467	5.7358865
N	7.9751338	4.6367129	1.9978653
N	7.1987159	4.0138963	5.7417348
N	5.8518881	3.8900605	2.0180419
N	7.4880234	7.6380793	4.3949953
N	5.6333415	7.6274000	3.1200554
C	6.0129916	4.0481486	6.3285967
C	7.0321958	3.9120593	1.4172277
C	5.7361239	3.2954899	7.5901851
C	7.2834414	3.1616007	0.1490479
C	5.0033863	2.1136710	7.5552936
C	8.0110039	1.9762347	0.1691581
H	4.6302983	1.7438359	6.6067078
H	8.3969620	1.6021324	1.1108600
C	4.7519163	1.4117957	8.7257561
H	4.1842006	0.4893012	8.6897329
C	5.2246336	1.8914638	9.9404286
H	5.0259600	1.3449546	10.8548022
C	5.9526846	3.0735702	9.9798276
H	6.3227242	3.4525633	10.9253500
C	6.2108104	3.7709836	8.8081692
H	6.7847306	4.6906087	8.8358271
C	3.8292650	5.1211096	6.3719834
C	9.2206593	4.9684585	1.3409131
H	3.6815888	4.5170738	7.2751220
H	9.3649661	4.3404656	0.4537682
C	3.8456031	6.5897877	6.7837191
C	9.1912602	6.4251507	0.8887081
H	4.0196484	7.2193772	5.9056448
H	9.0146232	7.0773188	1.7495789
H	2.8977079	6.8853218	7.2406820
H	10.1353777	6.7148093	0.4201755
H	4.6508051	6.7800034	7.4963792
H	8.3825902	6.5890030	0.1732898
C	2.6758367	4.8385934	5.4213817
C	10.3830232	4.7196936	2.2894334
H	2.6580299	3.7850980	5.1356078
H	10.4074557	3.6755975	2.6073710
H	1.7168399	5.0925552	5.8788019
H	11.3366832	4.9629639	1.8152748
H	2.7848253	5.4350863	4.5112915
H	10.2791677	5.3433858	3.1813011
C	8.2271190	3.0676077	6.1159637
C	4.8060786	2.9587564	1.6570018
H	7.9899769	2.6060799	7.0819475
H	5.0424589	2.4665982	0.7060839
C	9.5605518	3.7861125	6.2512427
C	3.4896799	3.7021924	1.4891916
H	9.5010470	4.5753657	7.0030979
H	3.5724359	4.4671431	0.7147008
H	10.3544158	3.0916369	6.5355770
H	2.6822051	3.0177572	1.2193204
H	9.8375321	4.2453455	5.2985582
H	3.2153311	4.1956944	2.4257355
C	8.3114269	1.9589980	5.0713669
C	4.6872642	1.8806702	2.7295999
H	8.5141885	2.3915566	4.0867636
H	4.4792681	2.3434031	3.6993134
H	9.1066251	1.2480104	5.3099410
H	3.8820284	1.1781906	2.4998923
H	7.3657615	1.4162477	5.0099994
H	5.6218561	1.3230552	2.8188685
C	6.5587018	8.3304194	3.7558625
C	6.5491934	9.8251239	3.7545128
C	5.6307602	10.5212915	4.5334912
H	4.9195135	9.9718049	5.1401224
C	5.6241523	11.9089840	4.5346849
H	4.9085247	12.4441724	5.1478954
C	6.5301177	12.6101182	3.7493954
H	6.5226702	13.6937140	3.7473475
C	7.4457191	11.9185441	2.9668179
H	8.1540830	12.4611934	2.3517464
C	7.4581114	10.5308959	2.9730364
H	8.1768190	9.9888334	2.3685556
C	8.4006891	8.2440070	5.3399980
C	4.7100066	8.2173808	2.1763541
H	8.3937546	9.3352964	5.2337896
H	4.7278589	9.3109439	2.2543731
C	9.8149631	7.7529244	5.0730915
C	3.2956892	7.7455152	2.4778700
H	9.8620492	6.6664514	5.1850711
H	3.2385204	6.6563267	2.3980288
H	10.1266458	8.0024389	4.0569947
H	3.0008697	8.0268039	3.4907226
H	10.5246360	8.1966183	5.7751733
H	2.5786019	8.1736791	1.7737503
C	7.9679179	7.9059712	6.7633444
C	5.1169093	7.8390785	0.7556216
H	7.9321145	6.8197918	6.8925963
H	5.1357617	6.7496508	0.6523469
H	8.6617956	8.3212690	7.4987174
H	4.4178791	8.2456338	0.0203189
H	6.9695738	8.3001067	6.9650499
H	6.1175270	8.2136511	0.5300965
C	6.7925267	3.6431455	-1.0600696
C	8.2420685	1.2773853	-1.0072722
C	7.0308609	2.9491031	-2.2379030
H	6.2226199	4.5655581	-1.0763476
C	7.7543466	1.7638004	-2.2133019
H	8.8061949	0.3523144	-0.9828060
H	6.6492491	3.3333596	-3.1766777
H	7.9378002	1.2200585	-3.1324954

[Np(*i*Pr$_2$BA)$_3$]

103			
Energy=	-2362.1235048420		
Np	-0.0001212	-0.0046196	0.0168389
C	-5.7261835	-3.1717298	0.7553766
N	-2.0360932	-0.9368288	-1.0065953
H	-2.9585070	-0.6791879	-4.2505305
N	-1.4671974	-1.7013628	1.0294818
H	-4.5886332	-5.5433571	-1.3781520
H	-6.5967552	-2.9212930	1.3503161
H	-0.2382209	-4.3336727	2.7700822
C	-2.3087303	-1.7413094	0.0083981
H	-1.4291841	-0.1881228	-3.5084065
C	-3.4989317	-2.6447471	0.0014521
H	-4.6197625	-1.4369088	1.3734208
C	-3.4966485	-3.8005597	-0.7725024
C	-2.2755937	-0.8750412	-3.4206047
H	-2.6257370	-4.0401009	-1.3724177
C	-1.4760153	-2.6761299	2.0973510
H	0.6484645	-3.0283868	1.9555220
H	-0.3286388	-4.1539914	1.0069948
H	-2.3878105	-3.2838552	2.0551682
C	-5.7182236	-4.3273721	-0.0149826
C	-1.4450692	-1.9624961	3.4407299
H	-6.5818784	-4.9818118	-0.0217505
H	-2.3084245	-1.3030395	3.5492296
H	2.0778331	-2.2142019	-4.2532782
C	-2.9689892	-0.6785725	-2.0804900
H	-1.4453544	-2.6771794	4.2669575
H	-3.8152161	-1.3742545	-2.0295468
H	-1.8950180	-1.8940021	-3.5145429
C	-3.5154524	0.7398753	-1.9558323
H	-0.5407970	-1.3525824	3.5201997
H	-2.6897334	1.4580372	-1.9669431
C	-4.6182854	-2.3361931	0.7674959
H	-4.1942669	0.9778646	-2.7787028
C	-0.2787661	-3.6098844	1.9521480
H	-4.0527730	0.8627976	-1.0134953
C	-4.6002598	-4.6418449	-0.7767142
C	-1.7300033	6.2911568	-0.7875352
C	6.3270099	-1.6187901	-0.7639516
C	-2.9807259	2.0419616	1.9589481
C	3.2585660	1.5579886	1.9570304
C	0.2763777	5.1642293	0.7634640
C	4.3423547	-2.8264635	0.7539416
C	2.3946392	2.6692508	-1.9678405
C	1.1248716	-3.4168074	-1.9657900
C	0.8921806	2.9036213	-2.0857235
C	2.0765865	-2.2308538	-2.0833496
C	-0.9654590	2.2236839	3.4405785
C	2.4128423	-0.2775833	3.4421889
C	-0.9032186	7.1066152	-0.0256830
C	6.6223094	-2.7467658	-0.0093295
C	-1.5720576	2.6094657	2.0996910
C	3.0486021	0.0540914	2.1003351
C	0.3699765	2.4005666	-3.4234667
C	1.9017597	-1.5280819	-3.4215517
C	-1.5479871	4.9154738	-0.7805214
C	5.0408370	-1.0976245	-0.7618681
C	-0.5455590	4.3446217	-0.0031295
C	4.0425033	-1.6973661	-0.0011973
C	-0.3532231	2.8627736	0.0065156
C	2.6603332	-1.1294131	0.0078320
C	0.1019148	6.5408609	0.7480324
C	5.6270712	-3.3511068	0.7478558
N	-0.7359723	2.1143217	1.0291057
N	2.2019120	-0.4254212	1.0308478
N	0.2074686	2.2245539	-1.0084556
N	1.8272974	-1.2990150	-1.0064731
H	2.7739932	3.0747571	-1.0279229
H	1.2857461	-3.9461669	-1.0246897
H	2.9354794	3.1377332	-2.7940020
H	1.2631029	-4.1204604	-2.7906031
H	2.6048604	1.5953357	-1.9787737
H	0.0889453	-3.0642287	-1.9790379
H	-0.8923612	1.1352950	3.5194911
H	1.4325280	0.2007634	3.5207485
H	-0.7035200	2.5785649	-3.5123719
H	2.5919484	-0.6869002	-3.5109341
H	0.7120743	3.9840690	-2.0344027
H	3.1033805	-2.6123771	-2.0304401
H	-1.5796559	2.5828391	4.2695629
H	3.0306012	0.0781094	4.2700335
H	0.0390438	2.6385020	3.5450086
H	2.2731343	-1.3551291	3.5489130
H	-1.0420896	8.1812574	-0.0347928
H	7.6263194	-3.1544211	-0.0116121
H	-1.6409145	3.7030778	2.0586903
H	4.0308948	-0.4314262	2.0594124
H	-3.4300645	2.3589715	1.0158242
H	3.7566512	1.7878682	1.0131339
H	-2.9425567	0.9482026	1.9609954
H	2.2912521	2.0698847	1.9588356
H	-2.1868146	4.2775788	-1.3811674
H	4.8074699	-0.2186455	-1.3524497
H	1.0559238	4.7199164	1.3723364
H	3.5650805	-3.2937406	1.3483396
H	0.5431684	1.3244951	-3.5123539
H	0.8829151	-1.1409275	-3.5108963
H	-3.6241193	2.3689594	2.7797060
H	3.8633888	1.9543711	2.7765697
H	0.7504537	7.1731546	1.3431530
H	5.8522334	-4.2324796	1.3369553
H	-2.5170294	6.7277198	-1.3913410
H	7.1002219	-1.1436462	-1.3563707
H	0.8760810	2.8952431	-4.2557229

[Pu(*i*Pr$_2$BA)$_3$]

103			
Energy=	-2401.6149912180		
Pu	-0.0297142	0.0541974	1.1160203
C	-5.6029470	-3.3727269	1.8486294
N	-2.0374429	-0.9203700	0.1019993
H	-2.9611537	-0.7024128	-3.1448960
N	-1.4462371	-1.6507651	2.1461473
H	-4.3358709	-5.6350786	-0.3303111
H	-6.4847294	-3.1844115	2.4499463
H	-0.0706408	-4.1923967	3.9106725
C	-2.2758652	-1.7344738	1.1184761
H	-1.4512406	-0.1593724	-2.3993365
C	-3.4127688	-2.7030337	1.0994886
H	-4.5979063	-1.5868675	2.4946705
C	-3.3456586	-3.8426946	0.3046139
C	-2.2736911	-0.8751895	-2.3136572
H	-2.4633526	-4.0204308	-0.3001289
C	-1.4095536	-2.6265372	3.2123612
H	0.7319713	-2.8409366	3.0812833
H	-0.1656307	-4.0407329	2.1454341
H	-2.2809448	-3.2893613	3.1545897
C	-5.5297131	-4.5123024	1.0580509
C	-1.4450426	-1.9144851	4.5563499
H	-6.3531183	-5.2165866	1.0424837
H	-2.3474774	-1.3068091	4.6483381
H	1.9965054	-2.1443531	-3.1726371
C	-2.9755120	-0.7020875	-0.9749551
H	-1.4190074	-2.6292183	5.3821789
H	-3.7968263	-1.4270559	-0.9233782
H	-1.8578385	-1.8800715	-2.4077889
C	-3.5707555	0.6971775	-0.8549802
H	-0.5799947	-1.2530886	4.6523593
H	-2.7694771	1.4426577	-0.8708801
C	-4.5458958	-2.4738066	1.8730275
H	-4.2588462	0.9095014	-1.6772419
C	-0.1541096	-3.4827212	3.0836432
H	-4.1093178	0.8056154	0.0884224
C	-4.3980418	-4.7469437	0.2877030
C	-1.6479661	6.3481777	0.2907522
C	6.2357012	-1.7387704	0.3027577
C	-2.9992110	2.0920843	3.0370962
C	3.2395433	1.5210556	3.0667446
C	0.3103711	5.1858139	1.8769742
C	4.2352375	-2.8529624	1.8706719
C	2.4191275	2.6098312	-0.8748585
C	0.9935264	-3.3289290	-0.8962331
C	0.9294145	2.9184678	-0.9778956
C	1.9872889	-2.1768312	-1.0030656
C	-0.9909609	2.2821048	4.5273300
C	2.3486873	-0.2857839	4.5584507
C	-0.8250309	7.1482502	1.0727581
C	6.5047268	-2.8626753	1.0732127
C	-1.5917854	2.6610197	3.1820416
C	2.9963362	0.0224288	3.2172366
C	0.3725220	2.4639741	-2.3190790
C	1.8414941	-1.4587465	-2.3363268
C	-1.4871473	4.9698924	0.2964965
C	4.9662079	-1.1783307	0.3100143
C	-0.5089902	4.3816230	1.0915257
C	3.9600218	-1.7310572	1.0955914
C	-0.3419574	2.8977859	1.1014810
C	2.5947126	-1.1251860	1.1073290
C	0.1561475	6.5648560	1.8639520
C	5.5015244	-3.4203630	1.8554035
N	-0.7542710	2.1551653	2.1180956
N	2.1436716	-0.4421108	2.1470416
N	0.2245600	2.2519143	0.0939566
N	1.7694000	-1.2449775	0.0800448
H	2.8251605	2.9886381	0.0650391
H	1.1433212	-3.8786678	0.0348685
H	2.9757197	3.0578257	-1.7017741
H	1.0979831	-4.0241584	-1.7331078
H	2.5743279	1.5268824	-0.8949148
H	-0.0277470	-2.9364954	-0.8933552
H	-0.9191599	1.1941471	4.6114487
H	1.3787853	0.2142802	4.6294976
H	-0.6924381	2.6927166	-2.3938111
H	2.5603698	-0.6411168	-2.4172924
H	0.7997081	4.0046949	-0.9045972
H	2.9995525	-2.5950929	-0.9502322
H	-1.6081576	2.6460476	5.3519377
H	2.9708989	0.0628200	5.3859113
H	0.0135420	2.6965218	4.6335717
H	2.1846809	-1.3588880	4.6732298
H	-0.9480029	8.2248402	1.0654331
H	7.4947691	-3.3031569	1.0643003
H	-1.6584192	3.7542726	3.1322846
H	3.9682560	-0.4843637	3.1825904
H	-3.4431660	2.4024545	2.0892023
H	3.7456392	1.7345508	2.1232901
H	-2.9586462	0.9983931	3.0459185
H	2.2827960	2.0528072	3.0617739
H	-2.1242441	4.3436253	-0.3179693
H	4.7511615	-0.3051934	-0.2960133
H	1.0719693	4.7277637	2.4982414
H	3.4516656	-3.2820050	2.4853464
H	0.4951487	1.3830207	-2.4276188
H	0.8371599	-1.0362362	-2.4257927
H	-3.6477310	2.4233275	3.8520211
H	3.8491811	1.9103289	3.8862276
H	0.8013953	7.1851774	2.4750024
H	5.7063740	-4.2982374	2.4570320
H	-2.4158635	6.7986452	-0.3272708
H	7.0157219	-1.2993352	-0.3080399
H	0.8932313	2.9496166	-3.1476300

4.2. Comparison to crystal structures

The overlays in **Figure S31** validate - additionally to the very similar An/Ln-N bond lengths - the good agreement between the crystal structures and the optimized geometries. For some of the structures a larger deviation can be detected for the outer phenyl rings, which illustrates the already mentioned differences between a deformed molecule due to crystal packing and a relaxed state with more degrees of freedom in solution. The inner regions of coordinating nitrogen atoms including the central atom on the other hand seem to be almost untouched by these effects as the structures are almost perfectly aligned in this case. Since most of the of the properties of interest focus on the immediate area around the central metal and the coordinative bonds towards it coming from the nitrogen atoms, this marks a solid foundation for the evaluation of electronic and magnetic characteristics.

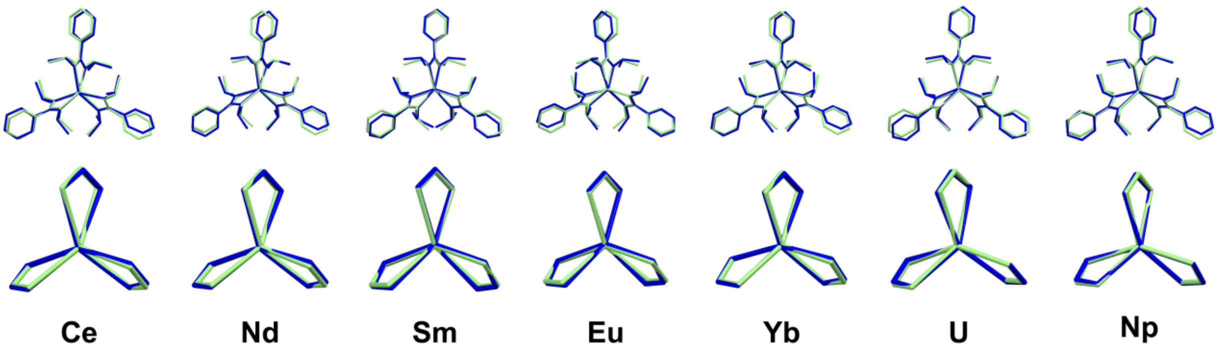

Figure S31. Overlay of calculated (green) and crystallographic (blue) complex structures. The upper row shows the whole structure (without hydrogen atoms) whereas the bottom row depicts only the coordinating nitrogen atoms and the central metal ion.

Table S9. Comparative analysis of bond lengths and RMSD for atoms in An/Ln complexes: calculated vs. experimental data from XRD Measurements. The relative error δd refers to the experimental value.

An^{III}/Ln^{III} complex	d(M-N) bond lengths (Å)			RMSD for atoms between calculated and experimental structures (Å)	
	calculation	experiment	δd (%)	All atoms	Inner coordination atoms and metal
Ce[9]	2.486(0)	2.487(5)	0.04	0.297	0.099
Nd (**2**)	2.452(3)	2.454(5)	0.08	0.320	0.109
Sm (**3**)	2.419(9)	2.428(7)	0.37	0.306	0.092
Eu (**4**)	2.412(5)	2.415(5)	0.12	0.333	0.114
Yb (**5**)	2.323(2)	2.318(5)	0.22	0.293	0.079
U (**7**)	2.473(5)	2.476(8)	0.12	0.312	0.100
Np (**8**)	2.462(1)	2.468(9)	0.24	0.273	0.080

4.3. Exploring the concept of covalency

One approach to get a quantitative measure of this property is Bader's QTAIM-Analysis,[36] allowing for a subdivision of the molecule into atomic fragments based on the electronic density. Using these fragments, it is possible to approximate the delocalized electrons between two atoms along the bond path, rendering the resulting so called Delocalization Index (DI) as a representation of covalency. In order to get accurate values from this kind of analysis, it is necessary to perform a more sophisticated *all electron single point* calculation including the Douglas-Kroll-Hess-Hamiltonian[37,38] (and according basis sets: DKH-DEF2-TZVPP, An/Ln: SARC-DKH-TZVPP) treating the scalar-relativistic effects explicitly. These were carried out using ORCA 5.0.4.[39]

A more indirect way to tackle the question of covalency is offered by the charge at the central metal, which after ligand coordination, allows the deduction of transferred electrons from the nitrogen atoms. Here, a NPA was applied to get the natural charge of the metal, resulting from summation of occupancies of all natural atomic orbitals and subsequent subtraction from the nuclear charge. As an intermediate step, this also gives an insight into the distribution of the electrons between 5*f*, 6*d* and 7*s* orbitals. The foundation for this analysis were the previously performed SVP TURBOMOLE calculations, which were chosen since the numerical errors are expected to be lower when using smaller basis sets. This prevents a pronounced occupation of chemically rather meaningless Rydberg orbitals.

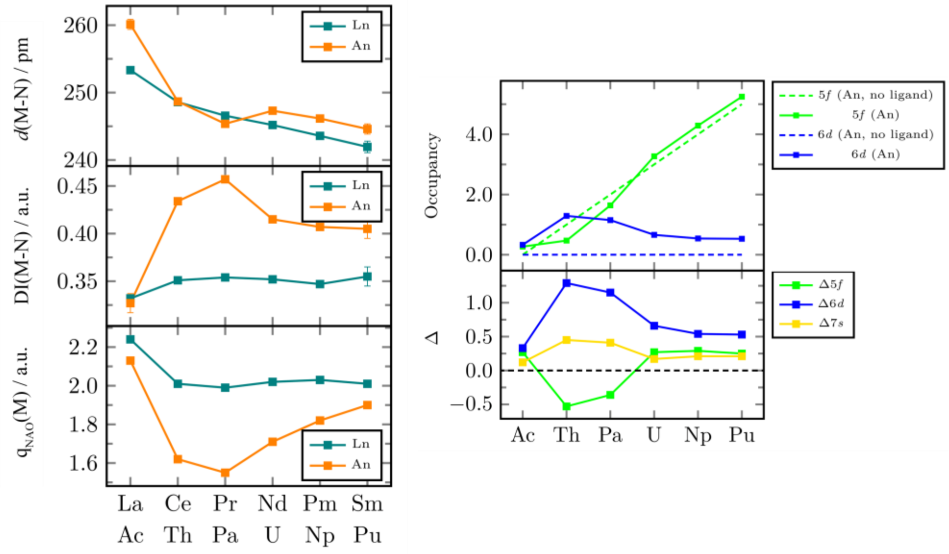

Figure S32. The diagrams on the left side depict the M-N bond length (**1**), the according DI (**2**) and natural charge of the central metal (**3**) for the first six lanthanides and actinides. The diagrams on the right side visualize the occupancy of 5*f* and 6*d* orbitals before and after ligand coordination (**4**) as well as the change in occupancy (Δ) of 5*f*, 6*d* and 7*s* orbitals relative to the configuration of the actinide cation before ligand coordination (**5**).

4.4. Electric Quadrupole Moments

For molecules that have at least C_3 symmetry, examining the dipole moments of the central metals is unrewarding since they do not exist. Therefore, to effectively describe the charge distribution, it is necessary to employ higher order terms from the Taylor expansion, such as the quadrupole moment. With the help of these, useful properties like the charge localization and deviation from spherically symmetric distribution can be deduced. The traceless quadrupole tensors were derived from the *all electron single point* calculations using MultiWFN.[40] They were then plotted via VMD[41] utilizing an associated VMD script in the form of ellipsoids, whose three principal axes correspond to the eigenvectors of the quadrupole tensor (**Figure S33**). These ellipsoids allow for an intuitive deduction of the charge distribution as their extent in a certain direction behaves inversely to the localization of electron density. Examining the quadrupole moments from the side view Th and Pa differ greatly from the other ellipsoids through their strong oblate shape meaning the electron density is located mainly above and below the ellipsoid. This could be explained by a stronger occupation of orbitals with extent in z-direction, like $5f_{z^3}$ or $6d_{z^2}$. Recalling Diagram 4 of **Figure S32** it is reasonable to assume that the latter plays a major role here. The prevalence of both $5f_{z^3}$ and $6d_{z^2}$ is backed when looking at the ellipsoids from above. Their way bigger expansion compared to the ellipsoids from Ac, U, Np and Pu suggests a strong localization of electron density in the xy-plane, which is the case for said orbitals. It is furthermore noticeable that the ellipsoids are very symmetric in the xy-plane realizing a circle like shape resembling an even charge distribution in this dimension. A comparable symmetric distribution is only exhibited by uranium.

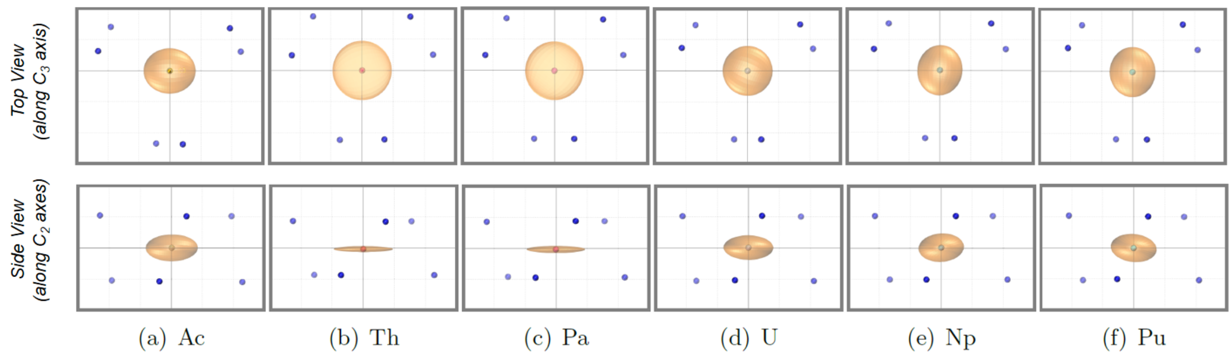

Figure S33. Electric quadrupole moments of the first six actinides represented by ellipsoids, seen from above (*top row*) and the side (*bottom row*). The blue dots mark the position of the nitrogen atoms. 1 gray square = 1 Å.

5. Paramagnetic NMR analysis

5.1. Experimental NMR Chemicals Shifts

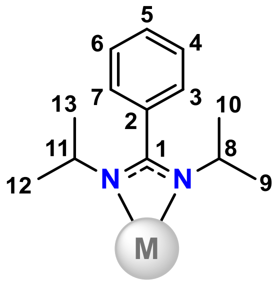

Table S10. Observed chemical ^{1}H and ^{13}C NMR shifts (ppm) for [M^{III}(*i*Pr_2BA)$_3$] in toluene-d_8 (303 K). Dashes (-) indicate signals that were not observed.

	La^{III}	Lu^{III}	Nd^{III}	Sm^{III}	Eu^{III}	Yb^{III}	U^{III}	Np^{III}
H3	7.20	7.26	13.20	9.57	-0.22	-12.88	13.34	9.06
H4	7.17	7.19	9.58	8.00	4.20	-0.20	9.38	7.82
H5	7.09	7.11	8.79	7.77	5.26	1.27	9.05	7.73
H8	3.28	3.37	22.66	2.53	-31.24	-13.31	28.12	17.80
$H9^{Me}$	1.29	1.31	-3.70	-0.51	12.8[a)]	43.49	-4.42	-0.96
$H10^{Me}$	1.29	1.31	-3.70	-0.51	2.2[a)]	-9.71	-4.42	-0.96
C1	175.90	176.89	-	-	-	66.32	-	-
C2	136.64	136.60	136.34	141.81	132.08	97.47	15.31	72.82
C3	127.02	126.70	133.56	129.18	116.89	106.85	126.87	120.74
C4	128.51	128.63	130.64	129.98	127.10	118.13	124.98	125.46
C5	127.72	127.97	131.26	128.68	122.17	118.91	128.40	128.74
C8	48.57	48.46	60.77	46.57	31.32	39.96	-	12.56
$C9^{Me}$	27.18	26.90	21.61	26.00	-	67.19	-	8.95
$C10^{Me}$	27.18	26.90	21.61	26.00	-	19.33	-	8.95

[a)] Values separated by deconvolution analysis due to very broad resonance at 303 K.

Table S11. Variable temperature ^{1}H NMR data in ppm. The light gray shadow on the table indicates a very broad resonance or undeterminable regions, due to the near coalescence temperature of the isopropyl methyl group resonance.

				**[Nd(*i*Pr$_2$BA)$_3$]**			
Temperature (K)	H3	H4	H5	H8	H9,10^{Me}	H9^{Me}	H10^{Me}
213.15	16.17	10.72	9.62	31.75		-18.68	6.28
223.15	15.78	10.57	9.51	30.46		-17.63	5.90
233.15	15.40	10.42	9.40	29.26		-16.48	5.36
243.15	15.05	10.29	9.30	28.15		-15.07	4.20
253.15	14.70	10.15	9.20	27.07		-12.00	2.48
263.15	14.37	10.02	9.11	26.08	-4.97		
273.15	14.06	9.91	9.02	25.15	-4.42		
283.15	13.75	9.79	8.94	24.26	-4.12		
293.15	13.48	9.69	8.86	23.45	-3.92		
**303.15**	**13.20**	**9.58**	**8.79**	**22.66**	**-3.70**		
313.15	12.95	9.49	8.72	21.94	-3.49		
323.15	12.71	9.40	8.65	21.26	-3.27		
333.15	12.49	9.31	8.59	20.60	-3.09		
343.15	12.28	9.24	8.54	19.99	-2.90		
353.15	12.06	9.15	8.47	19.39	-2.74		
				**[Sm(*i*Pr$_2$BA)$_3$]**			
Temperature (K)	H3	H4	H5	H8	H9,10^{Me}	H9^{Me}	H10^{Me}
218.15	9.89	8.05	7.81	3.14		-4.43	2.86
228.15	9.85	8.05	7.80	3.06		-4.31	2.80
238.15	9.82	8.05	7.80	2.97		-4.18	2.69
248.15	9.78	8.04	7.80	2.90		-3.82	2.53
258.15	9.73	8.03	7.78	2.82			
268.15	9.71	8.03	7.80	2.77			
278.15	9.67	8.02	7.78	2.69	-0.54		
288.15	9.63	8.02	7.78	2.62	-0.54		
298.15	9.59	8.01	7.77	2.56	-0.53		
**303.15**	**9.57**	**8.00**	**7.77**	**2.53**	**-0.51**		
308.15	9.55	8.00	7.77	2.50	-0.50		
318.15	9.52	7.99	7.76	2.45	-0.47		
328.15	9.48	7.98	7.75	2.39	-0.44		
338.15	9.45	7.97	7.75	2.34	-0.41		
348.15	9.41	7.96	7.74	2.30	-0.38		
358.15	9.37	7.95	7.73	2.25	-0.34		
				**[Eu(*i*Pr$_2$BA)$_3$]**			
Temperature (K)	H3	H4	H5	H8	H9,10^{Me}	H9^{Me}	H10^{Me}
213.15	-4.71	2.39	3.94	-41.42		27.44	-5.97
223.15	-4.12	2.63	4.11	-40.17		26.02	-5.53
233.15	-3.55	2.86	4.27	-38.92		24.65	-5.11
243.15	-2.99	3.03	4.45	-37.69		23.4	-4.69
253.15	-2.47	3.30	4.61	-36.52		22.17	-4.32
263.15	-1.97	3.50	4.75	-35.38		21.02	-3.97
273.15	-1.50	3.69	4.89	-34.28		19.87	-3.53
283.15	-1.06	3.87	5.02	-33.24		18.48	-2.92
293.15	-0.63	4.04	5.14	-32.24		16.70	-1.62
**303.15**	**-0.22**	**4.20**	**5.26**	**-31.24**		**12.80**	**2.20**
313.15	0.14	4.34	5.36	-30.35			
323.15	0.49	4.48	5.46	-29.47			
333.15	0.86	4.64	5.58	-28.59	6.29		
343.15	1.16	4.75	5.65	-27.78	6.02		
353.15	1.47	4.87	5.74	-27.00	5.75		

(table continues)

Temperature (K)	H3	H4	H5	H8	H9,10^{Me}	H9^{Me}	H10^{Me}
**[Yb(*i*Pr$_2$BA)$_3$]**							
218.15	-21.31	-3.51	-1.40	-19.83		62.25	-15.51
228.15	-20.14	-3.06	-1.05	-18.96		59.59	-14.69
238.15	-19.00	-2.61	-0.68	-18.10		57.01	-13.90
248.15	-17.92	-2.19	-0.34	-17.27		54.56	-13.15
258.15	-16.90	-1.79	-0.02	-16.48		52.24	-12.44
268.15	-15.94	-1.42	0.28	-15.74		50.06	-11.79
278.15	-15.02	-1.06	0.57	-15.04		48.03	-11.17
288.15	-14.17	-0.74	0.82	-14.35		46.10	-10.59
298.15	-13.35	-0.42	1.08	-13.70		44.28	-10.05
**303.15**	**-12.88**	**-0.20**	**1.27**	**-13.31**		**43.49**	**-9.71**
308.15	-12.58	-0.13	1.31	-13.08		42.59	-9.53
318.15	-11.85	0.15	1.53	-12.49		40.97	-9.05
328.15	-11.15	0.40	1.74	-11.95		39.41	-8.61
338.15	-10.51	0.65	1.93	-11.41		37.97	-8.17
348.15	-9.88	0.89	2.13	-10.91		36.58	-7.79
358.15	-9.27	1.12	2.31	-10.39		35.33	-7.37

Temperature (K)	H3	H4	H5	H8	H9,10^{Me}	H9^{Me}	H10^{Me}
**[U(*i*Pr$_2$BA)$_3$]**							
213.15	15.05	9.97	9.53	36.83			
223.15	14.84	9.90	9.48	35.66	-6.02		
233.15	14.63	9.83	9.42	34.55	-5.53		
243.15	14.43	9.76	9.36	33.48	-5.38		
253.15	14.23	9.69	9.31	32.48	-5.23		
263.15	14.04	9.62	9.25	31.51	-5.08		
273.15	13.86	9.57	9.20	30.60	-4.91		
283.15	13.68	9.50	9.15	29.73	-4.72		
293.15	13.51	9.44	9.10	28.92	-4.58		
**303.15**	**13.34**	**9.38**	**9.05**	**28.12**	**-4.42**		
313.15	13.18	9.32	9.00	27.36	-4.26		
323.15	13.03	9.28	8.97	26.66	-4.08		
333.15	12.86	9.21	8.91	25.95	-3.96		
343.15	12.71	9.16	8.87	25.27	-3.82		
353.15	12.57	9.11	8.83	24.64	-3.68		

Temperature (K)	H3	H4	H5	H8	H9,10^{Me}	H9^{Me}	H10^{Me}
**[Np(*i*Pr$_2$BA)$_3$]**							
213.15	11.36	8.58	8.37	23.00		-9.72	3.40
223.15	11.06	8.48	8.29	22.39		-8.46	2.40
233.15	10.78	8.39	8.22	21.77			
243.15	10.52	8.32	8.16	21.18			
253.15	10.21	8.20	8.05	20.54	-2.08		
263.15	9.96	8.12	7.98	19.95	-1.85		
273.15	9.72	8.04	7.92	19.39	-1.59		
283.15	9.42	7.94	7.84	18.83	-1.41		
293.15	9.27	7.89	7.80	18.32	-1.17		
**303.15**	**9.06**	**7.82**	**7.73**	**17.80**	**-0.96**		
313.15	8.88	7.76	7.68	17.33	-0.79		
323.15	8.70	7.70	7.63	16.86	-0.61		
333.15	8.54	7.64	7.59	16.41	-0.45		
343.15	8.40	7.59	7.56	16.00	-0.29		
353.15	8.24	7.54	7.50	15.58	-0.17		

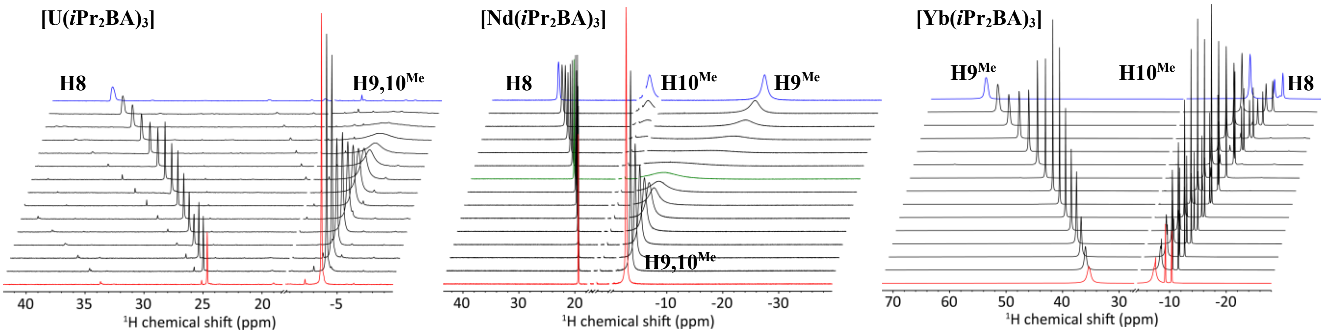

Figure S34. Stacked ^1H NMR spectra from variable temperature experiments on U, Nd, Yb amidinate complexes, featuring the isopropyl group protons resonance region.

VT-NMR spectra of three complexes with distinct ionic radii are shown: U^{3+} (1.025 Å), Nd^{3+} (0.983 Å), and Yb^{3+} (0.868 Å).[11] The U complex, having the largest ionic radius, exhibits no hindered rotation of the isopropyl group within the NMR timeframe, even at the lowest experimental temperature. Conversely, the Yb complex, with the smallest ionic radius, displays hindered rotation at all temperature ranges, leading to distinct PCS shifts for the two methyl groups of the isopropyl group ($H9^{Me}$ and $H10^{Me}$). The Nd complex, lying between these two cases, rotation hindrance is apparent in the lower temperature region. With increasing temperature, however, the PCS shifts of the isopropyl group's methyl groups coalesce into a singular resonance.

5.2. Curie and Bleaney plots

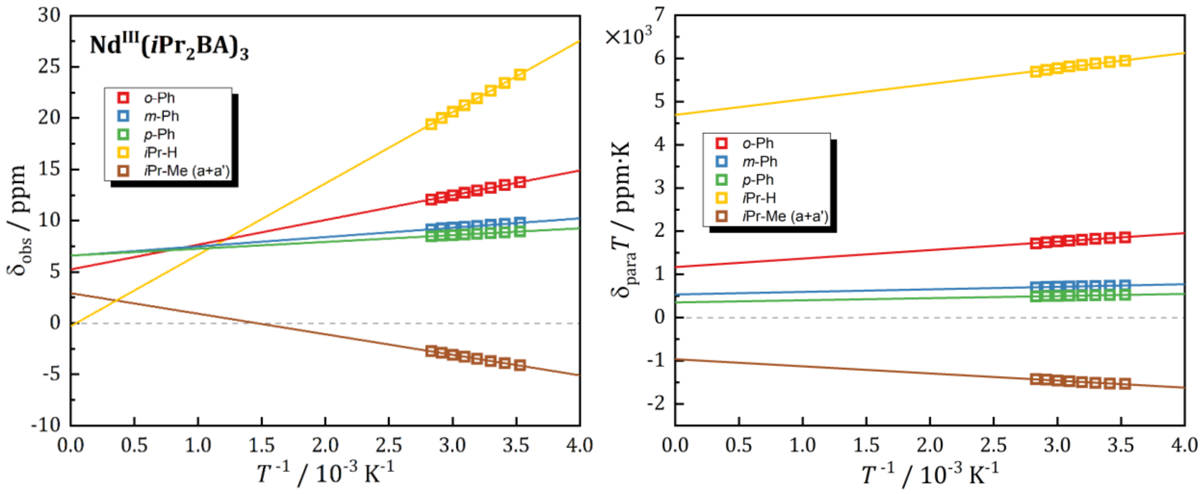

Figure S35. Curie (left) and Bleaney (right) plots illustrating the temperature dependence of paramagnetic ^{1}H NMR shifts in [Nd^{III}(iPr$_2$BA)$_3$], with the straight lines representing the best fit of the data to Curie's law and $\boldsymbol{\delta_{para}T = f(1/T)}$, respectively.

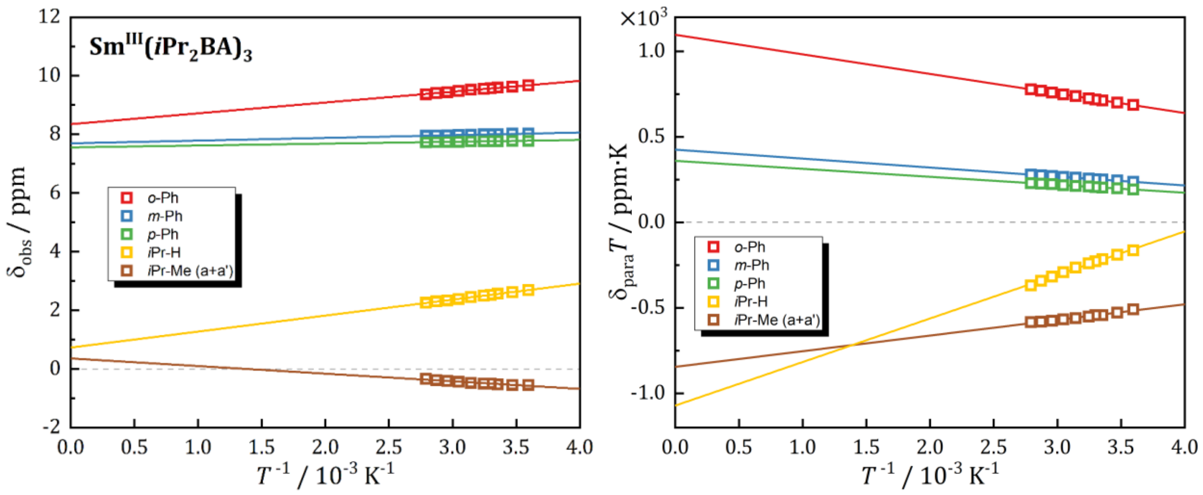

Figure S36. Curie (left) and Bleaney (right) plots illustrating the temperature dependence of paramagnetic ^{1}H NMR shifts in [Sm^{III}(iPr$_2$BA)$_3$], with the straight lines representing the best fit of the data to Curie's law and $\boldsymbol{\delta_{para}T = f(1/T)}$, respectively.

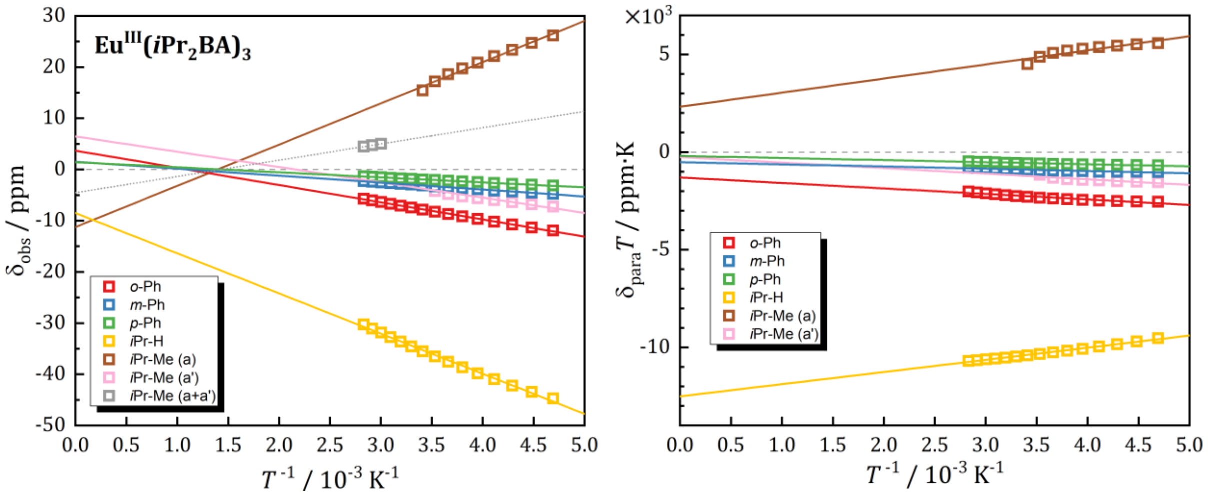

Figure S37. Curie (left) and Bleaney (right) plots illustrating the temperature dependence of paramagnetic ^{1}H NMR shifts in [Eu^{III}(*i*Pr$_2$BA)$_3$], with the straight lines representing the best fit of the data to Curie's law and $\boldsymbol{\delta_{para}T = f(1/T)}$, respectively.

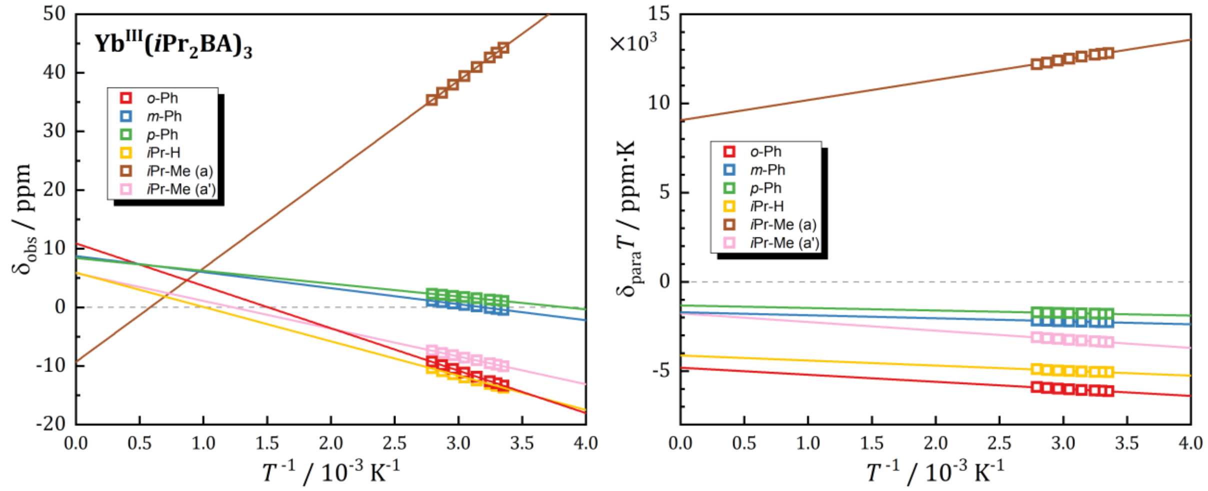

Figure S38. Curie (left) and Bleaney (right) plots illustrating the temperature dependence of paramagnetic ^{1}H NMR shifts in [Yb^{III}(*i*Pr$_2$BA)$_3$], with the straight lines representing the best fit of the data to Curie's law and $\boldsymbol{\delta_{para}T = f(1/T)}$, respectively.

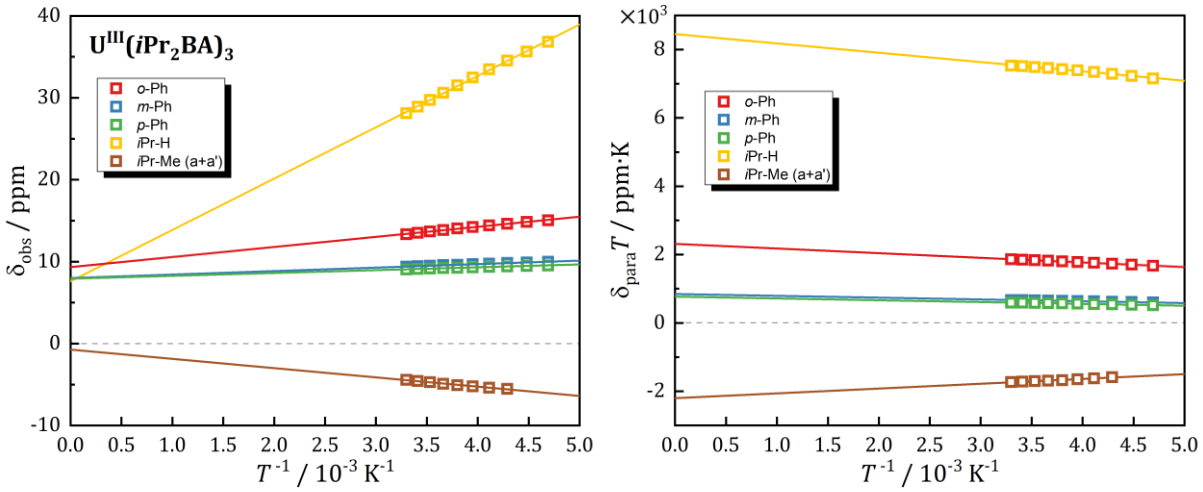

Figure S39. Curie (left) and Bleaney (right) plots illustrating the temperature dependence of paramagnetic ^{1}H NMR shifts in [U^{III}(iPr$_2$BA)$_3$], with the straight lines representing the best fit of the data to Curie's law and $\boldsymbol{\delta_{para}T = f(1/T)}$, respectively.

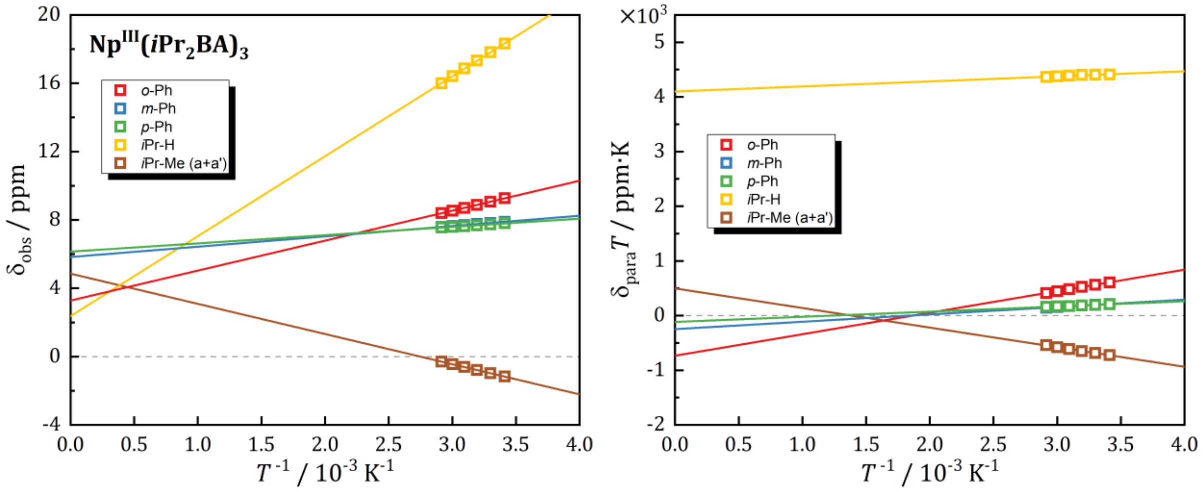

Figure S40. Curie (left) and Bleaney (right) plots illustrating the temperature dependence of paramagnetic ^{1}H NMR shifts in [Np^{III}(iPr$_2$BA)$_3$], with the straight lines representing the best fit of the data to Curie's law and $\boldsymbol{\delta_{para}T = f(1/T)}$, respectively.

Table S12. Summary of the best fit analysis of CURIE plots. Brackets indicate the standard error of the last digits by linear regression analysis.

		H3<br>*o*-Ph^H	H4<br>*m*-Ph^H	H5<br>*p*-Ph^H	H8<br>*i*Pr-H	H9,10^{Me}<br>*i*Pr-Me (a+a')	H9^{Me}<br>*i*Pr-Me (a)	H10^{Me}<br>*i*Pr-Me (a')
	Intercept	5.24(5)	6.58(3)	6.60(2)	-0.29(9)	2.93(9)	-	-
Nd^{III}	Slope	2.41(2)	0.91(1)	0.66(1)	6.96(3)	-2.00(3)	-	-
	R^2	>0.99	>0.99	>0.99	>0.99	>0.99	-	-
	Intercept	8.35(3)	7.70(2)	7.56(2)	0.73(1)	0.36(7)	-	-
Sm^{III}	Slope	0.37(1)	0.09(1)	0.06(1)	0.55(0)	-0.26(2)	-	-
	R^2	>0.99	0.97	0.94	>0.99	0.93	-	-
	Intercept	3.67(13)	1.51(6)	1.41(3)	-8.52(53)	-3.24(16)	-11.25(137)	6.45(131)
Eu^{III}	Slope	-3.36(4)	-1.36(2)	-0.98(1)	-7.84(14)	3.18(5)	8.05(34)	-2.99(33)
	R^2	>0.99	>0.99	>0.99	>0.99	>0.99	0.99	0.91
	Intercept	10.91(9)	8.75(4)	8.42(4)	5.92(13)	-	-9.32(13)	5.84(7)
Yb^{III}	Slope	-7.24(3)	-2.74(1)	-2.19(1)	-5.86(4)	-	15.99(4)	-4.74(2)
	R^2	>0.99	>0.99	>0.99	>0.99	-	>0.99	>0.99
	Intercept	9.33(10)	8.00(4)	7.92(4)	7.60(32)	-0.73(16)	-	-
U^{III}	Slope	1.23(3)	0.42(1)	0.35(1)	6.27(8)	-1.13(4)	-	-
	R^2	>0.99	>0.99	>0.99	>0.99	0.99	-	-
	Intercept	3.28(6)	5.83(1)	6.14(7)	2.37(9)	4.86(2)	-	-
Np^{III}	Slope	1.75(2)	0.60(0)	0.48(2)	4.68(3)	-1.77(1)	-	-
	R^2	>0.99	>0.99	0.99	>0.99	>0.99	-	-

Table S13. Separation of contact (δ_{FCS}) and pseudocontact (δ_{PCS}) contributions with 1/T and $1/T^2$ from variable temperature ^{1}H NMR chemical shifts of *i*Pr_2BA complex series using BLEANEY method.

Nd^{III} (303 K) (ppm)		H3<br>*o*-Ph^H	H4<br>*m*-Ph^H	H5<br>*p*-Ph^H	H8<br>*i*Pr-H	$H9,10^{Me}$<br>*i*Pr-Me (a+a')	$H9^{Me}$<br>*i*Pr-Me (a)	$H10^{Me}$<br>*i*Pr-Me (a')
Exp	δ_{para}	6.00	2.41	1.70	19.38	-4.99	-	-
best fit range (283-353 K)	δ_{FCS}	3.86(10)	1.77(4)	1.16(3)	15.48(18)	-3.18(12)	-	-
	δ_{PCS}	2.14(10)	0.64(4)	0.54(4)	3.89(19)	-1.79(13)	-	-
	R^2	0.98	0.97	0.97	0.98	0.97	-	-
	$\delta_{FCS}+\delta_{PCS}$	5.99	2.41	1.70	19.37	-4.97	-	-
Sm^{III} (303 K) (ppm)		H3<br>*o*-Ph^H	H4<br>*m*-Ph^H	H5<br>*p*-Ph^H	H8<br>*i*Pr-H	$H9,10^{Me}$<br>*i*Pr-Me (a+a')	$H9^{Me}$<br>*i*Pr-Me (a)	$H10^{Me}$<br>*i*Pr-Me (a')
Exp	δ_{para}	2.37	0.83	0.68	-0.75	-1.80	-	-
best fit range (278-358 K)	δ_{FCS}	3.62(1)	1.40(1)	1.19(1)	-3.53(7)	-2.79(5)	-	-
	δ_{PCS}	-1.25(1)	-0.57(1)	-0.51(1)	2.77(8)	0.99(5)	-	-
	R^2	>0.99	>0.99	>0.99	>0.99	0.97	-	-
	$\delta_{FCS}+\delta_{PCS}$	2.37	0.83	0.68	-0.76	-1.79	-	-
Eu^{III} (303 K) (ppm)		H3<br>*o*-Ph^H	H4<br>*m*-Ph^H	H5<br>*p*-Ph^H	H8<br>*i*Pr-H	$H9,10^{Me}$<br>*i*Pr-Me (a+a')	$H9^{Me}$<br>*i*Pr-Me (a)	$H10^{Me}$<br>*i*Pr-Me (a')
Exp	δ_{para}	-7.42	-2.97	-1.83	-34.52	-	11.51	0.91
best fit range (213-353 K)	δ_{FCS}	-4.28(25)	-1.69(10)	-0.64(8)	-41.27(18)	-	7.65(155)	-0.83(66)
	δ_{PCS}	-3.06(22)	-1.25(9)	-1.17(7)	6.78(16)	-	7.86(127)	-3.11(53)
	R^2	0.93	0.93	0.95	>0.99	-	0.82	0.82
	$\delta_{FCS}+\delta_{PCS}$	-7.35	-2.94	-1.81	-34.49	-	15.52	-3.94
Yb^{III} (303 K) (ppm)		H3<br>*o*-Ph^H	H4<br>*m*-Ph^H	H5<br>*p*-Ph^H	H8<br>*i*Pr-H	$H9,10^{Me}$<br>*i*Pr-Me (a+a')	$H9^{Me}$<br>*i*Pr-Me (a)	$H10^{Me}$<br>*i*Pr-Me (a')
Exp	δ_{para}	-20.08	-7.37	-5.82	-16.59	-	42.20	-11.00
best fit range (298-358 K)	δ_{FCS}	-15.87(18)	-5.62(8)	-4.36(7)	-13.61(20)	-	29.88(32)	-5.83(15)
	δ_{PCS}	-4.30(19)	-1.83(9)	-1.54(7)	-3.07(21)	-	12.28(34)	-5.26(17)
	R^2	>0.99	>0.99	0.98	0.97	-	>0.99	>0.99
	$\delta_{FCS}+\delta_{PCS}$	-20.17	-7.45	-5.90	-16.68	-	42.15	-11.09

U^{III} (303 K) (ppm)		H3<br>*o*-Ph^H	H4<br>*m*-Ph^H	H5<br>*p*-Ph^H	H8<br>*i*Pr-H	$H9,10^{Me}$<br>*i*Pr-Me (a+a')	$H9^{Me}$<br>*i*Pr-Me (a)	$H10^{Me}$<br>*i*Pr-Me (a')
Exp	δ_{para}	6.14	2.21	1.96	24.84	-5.71	-	-
best fit range (213-303 K)	δ_{FCS}	7.63(2)	2.79(1)	2.54(1)	27.89(14)	-7.26(9)	-	-
	δ_{PCS}	-1.48(2)	-0.58(1)	-0.58(1)	-2.97(12)	1.53(8)	-	-
	R^2	>0.99	>0.99	>0.99	0.99	0.98	-	-
	$\delta_{FCS}+\delta_{PCS}$	6.16	2.22	1.96	24.92	-5.73	-	-
Np^{III} (303 K) (ppm)		H3<br>*o*-Ph^H	H4<br>*m*-Ph^H	H5<br>*p*-Ph^H	H8<br>*i*Pr-H	$H9,10^{Me}$<br>*i*Pr-Me (a+a')	$H9^{Me}$<br>*i*Pr-Me (a)	$H10^{Me}$<br>*i*Pr-Me (a')
Exp	δ_{para}	1.86	0.65	0.64	14.52	-2.25	-	-
best fit range (293-343 K)	δ_{FCS}	-2.42(5)	-0.82(4)	-0.38(5)	13.52(12)	1.64(10)	-	-
	δ_{PCS}	4.28(5)	1.47(4)	1.03(5)	1.00(12)	-3.90(11)	-	-
	R^2	>0.99	>0.99	>0.99	0.93	>0.99	-	-
	$\delta_{FCS}+\delta_{PCS}$	1.86	0.65	0.64	14.53	-2.26	-	-

5.3. Geometrical parameter analysis and REILLEY method[42–46]

$$\delta_{para}^{i,Ln} = \delta_{PCS}^{i,Ln} + \delta_{FCS}^{i,Ln}$$

The paramagnetic chemical shift consists of a combination of the contact interaction ($\delta_{FCS}^{i,Ln}$), which is the result of the paramagnetic lanthanide ion's (Ln) spin density extending to the nucleus (i) through the chemical bonding, and the pseudocontact interaction ($\delta_{PCS}^{i,Ln}$), originating from the spatial dipole-dipole interaction between the magnetic moments of the lanthanide center (Ln) and the nucleus (i). These interactions, $\delta_{FCS}^{i,Ln}$ and $\delta_{PCS}^{i,Ln}$, can be broken down into the multiplication of various factors as presented in the following equation:

$$\delta_{para}^{i,Ln} = G_i \cdot A_2^0\langle r^2\rangle \cdot C_D^{Ln} + F_i \cdot \langle S_z\rangle_{Ln}$$

Where G_i is the geometric factor of the nucleus (i) containing the structural information which can be defined by following equation in the rotational symmetry. Where r_i is the Ln-nucleus (i) distance and θ_i is the angle between the Ln-nucleus (i) vector and the principal axis of the magnetic susceptibility tensor.

$$G_i = \frac{3\,cos^2\,\theta_i - 1}{r_i^3}$$

$A_2^0\langle r^2\rangle$ is the second-order axial ligand field parameter, C_D^{Ln} serves as a magnetic constant at a specific temperature (303 K in this study), reflecting the axial magnetic anisotropy of the paramagnetic ion (referred to here as BLEANEY's constant). F_i is related to the coupling constant of electron-nucleus hyperfine interactions, and $\langle S_z\rangle_{Ln}$ is the average magnetization of electron spin in alignment with the external magnetic field.

Table S14. Calculated geometrical factors (G_i) obtained from DFT optimized structure.

G_i		Nd^{III}	Sm^{III}	Eu^{III}	Yb^{III}	U^{III}	Np^{III}
H3	*o*-Ph	-0.0062(9)	-0.0064(2)	-0.0063(1)	-0.00656(3)	-0.0063(2)	-0.00616(4)
H4	*m*-Ph	-0.0024(2)	-0.00234(3)	-0.00232(1)	-0.002406(5)	-0.00230(3)	-0.002276(9)
H5	*p*-Ph	-0.00186(1)	-0.00181(1)	-0.001820(3)	-0.001887(1)	-0.0017732(1)	-0.0017796(3)
H8	*i*Pr-H	-0.0036(12)	-0.0043(1)	-0.0043(1)	-0.00467(4)	-0.0039(2)	-0.00418(3)
$H9^{Me}$	*i*Pr-Me(a)	-0.0057(40)	-0.0044(47)	-0.0041(49)	-0.0040(52)	-0.0043(44)	-0.0047(44)
$H10^{Me}$	*i*Pr-Me(a')	0.0159(88)	0.0153(98)	0.0152(97)	0.0155(99)	0.0156(98)	0.0153(97)
C2	*i*-Ph	-0.01178(4)	-0.01232(7)	-0.01239(4)	-0.01324(5)	-0.011790(2)	-0.011875(3)
C3	*o*-Ph	-0.00652(39)	-0.00678(3)	-0.00677(3)	-0.00714(1)	-0.00657(10)	-0.00656(1)
C4	*m*-Ph	-0.00334(14)	-0.00345(1)	-0.00345(1)	-0.003605(3)	-0.00336(3)	-0.00336(1)
C5	*p*-Ph	-0.00270(1)	-0.00278(1)	-0.00279(1)	-0.002904(1)	-0.0027035(1)	-0.002715(1)
C8	*i*Pr-H	-0.00073(330)	-0.00045(23)	-0.00011(13)	0.00012(6)	-0.00023(47)	-0.00078(16)
$C9^{Me}$	*i*Pr-Me(a)	-0.00541(203)	-0.00421(39)	-0.00394(15)	-0.00389(7)	-0.00407(18)	-0.00457(23)
$C10^{Me}$	*i*Pr-Me(a')	0.01414(295)	0.01355(28)	0.01354(11)	0.01407(9)	0.01370(18)	0.01339(6)

Table S15. $<S_z>$ and C_D values from the literature

	$<S_z>_{303K}$[47,48]	C_D[49,50]
Nd^{III}	-4.448	-4.2
Sm^{III}	0.224	-0.7
Eu^{III}	7.569	4.0
Yb^{III}	2.589	22

Table S16. Ratio of paramagnetic shift and the averaged geometrical factor for the ^{1}H at 303 K.[42,45]

	o-Ph/*p*-Ph		Diff. (%)	*m*-Ph/*p*-Ph		Diff. (%)	*i*Pr-H/*p*-Ph		Diff. (%)
	G_{H3}/G_{H5}	$(\delta_{para})_{H3}/(\delta_{para})_{H5}$		G_{H4}/G_{H5}	$(\delta_{para})_{H4}/(\delta_{para})_{H5}$		G_{H8}/G_{H5}	$(\delta_{para})_{H8}/(\delta_{para})_{H5}$	
Nd^{III}	3.47(8)	3.53	1.7	1.28(1)	1.42	10.8	2.28(18)	11.40	400
Sm^{III}		3.49	0.4		1.22	-4.6		-1.10	-148
Eu^{III}		4.05	16.8		1.62	26.8		18.86	727
Yb^{III}		3.45	-0.6		1.27	-1.1		2.85	25
U^{III}		3.13	-9.7		1.13	-11.9		12.67	456
Np^{III}		2.91	-16.2		1.02	-20.7		22.69	895

$$\frac{\delta_{para}^{i,Ln}}{\delta_{para}^{j,Ln}} = \frac{G_i \cdot A_2^0\langle r^2\rangle \cdot C_D^{Ln}}{G_j \cdot A_2^0\langle r^2\rangle \cdot C_D^{Ln}} = \frac{G_i}{G_j}$$

The geometrical factors (G_i) and paramagnetic shift (δ_{para}) ratios for protons at H3 (*o*-Ph), H4 (*m*-Ph), and H8 (*i*Pr-H) were analyzed in comparison to the proton at H5 (*p*-Ph), which is located furthest from the paramagnetic center. It can be assumed that for protons distanced by multiple bonds from the paramagnetic center, the contribution from contact interactions becomes minimal that it can generally be disregarded. Consequently, in the equation governing paramagnetic shifts, the contact term ($F_i \cdot \langle S_z\rangle_{Ln}$) vanishes, leaving only the pseudocontact contribution. Given that the ligand field parameter ($A_2^0\langle r^2\rangle$) and the BLEANEY constant (C_D) are constants, the ratio can be represented solely by the geometrical factors. Significant discrepancies might suggest either the possibility of contact contributions extending over such distances or inaccuracies in the equation's depiction of pseudocontact contributions. Thus, a close correlation implies a negligible contact contribution.

Upon examining the results presented in the above table, discrepancies observed for the H4 (*m*-Ph) position indicate that contact contributions cannot be entirely ignored even at this distance. For H8 (*i*Pr-H) protons, a pronounced error was observed, largely ascribed to substantial FCS contributions. Moreover, it is important to consider the alterations in the geometrical factor brought by the isopropyl group's rotation. Consequently, due to the large deviations in geometrical factors resulting from the rotation of the isopropyl group, protons of the isopropyl methyl group ($H9^{Me}$, $H10^{Me}$) were excluded from the REILLEY analysis.

Notably, For the Yb complex, an increase in the *J* value (total angular momentum quantum number) enhances the pseudocontact contribution. This leads to the observed reduction in sensitivity to contact contribution.

Figure S41. Analysis of geometric deviations[42,51]

$$\frac{\delta_{para}^{i,Ln}}{\langle S_z \rangle_{Ln}} = \left(F_i - \frac{G_i}{G_j} \cdot F_j\right) + \frac{G_i}{G_j} \cdot \frac{\delta_{para}^{j,Ln}}{\langle S_z \rangle_{Ln}}$$

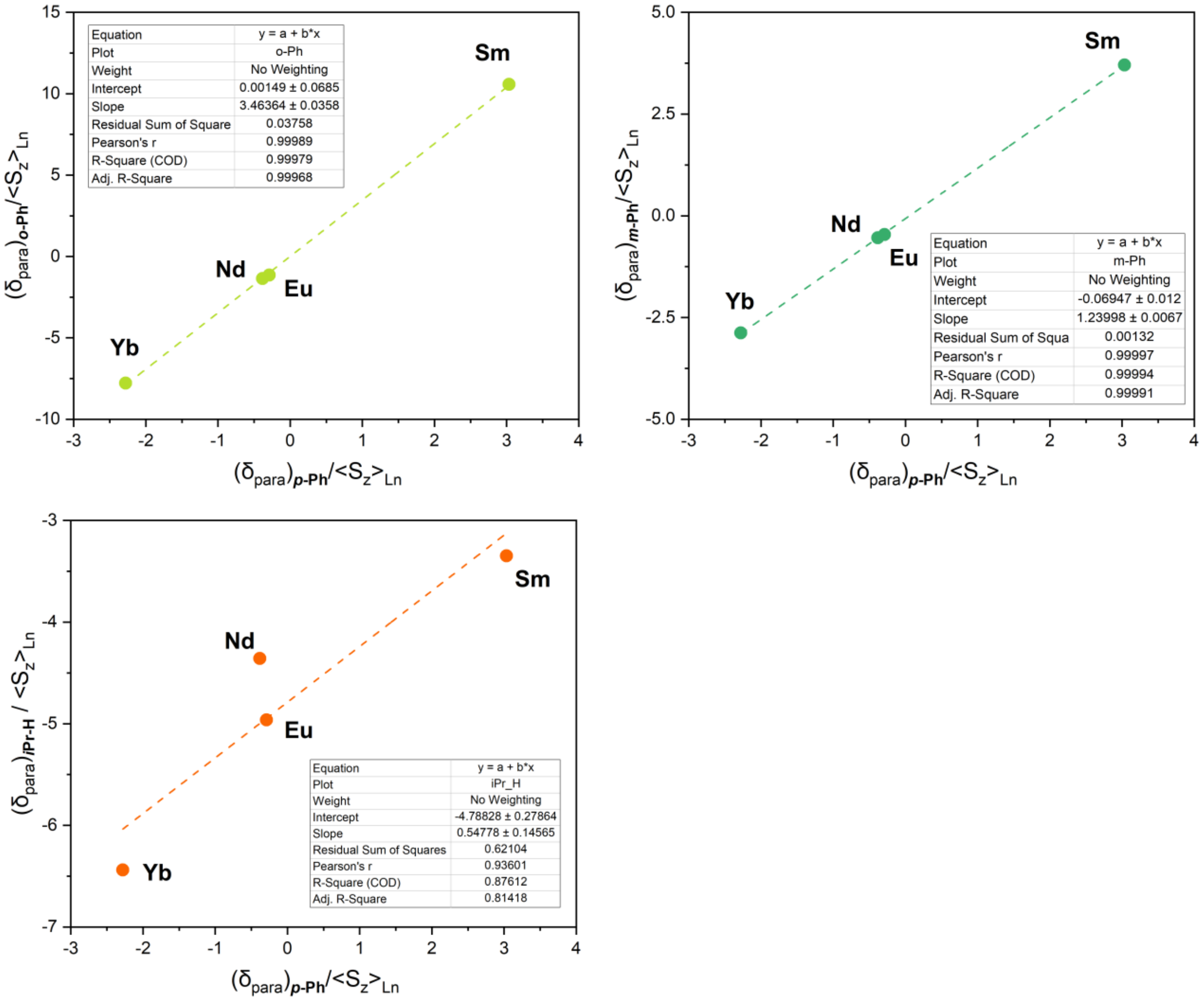

To evaluate the origins of the deviations observed in **Table S16**, an analysis was conducted using the equation above, which assumes that the ligand field parameter remains constant throughout the Ln series. Therefore, deviations from linearity indicate either a structural change affecting the geometrical factor (G_i) or variations in the ligand field. While the phenyl group protons (H3, H4, and H5) demonstrate good linearity, the observed deviations in the H8 (*i*Pr-H) suggest that the deviations in the isopropyl methine group are likely due to changes in the geometrical factor.

Figure S42. Determination of the crystal field parameter[42] $A_2^0\langle r^2\rangle$

$$\frac{\delta_{para}^{i,Ln}}{\langle S_z\rangle_{Ln}} = G_i \cdot A_2^0\langle r^2\rangle \cdot \frac{C_D^{Ln}}{\langle S_z\rangle_{Ln}} + F_i$$

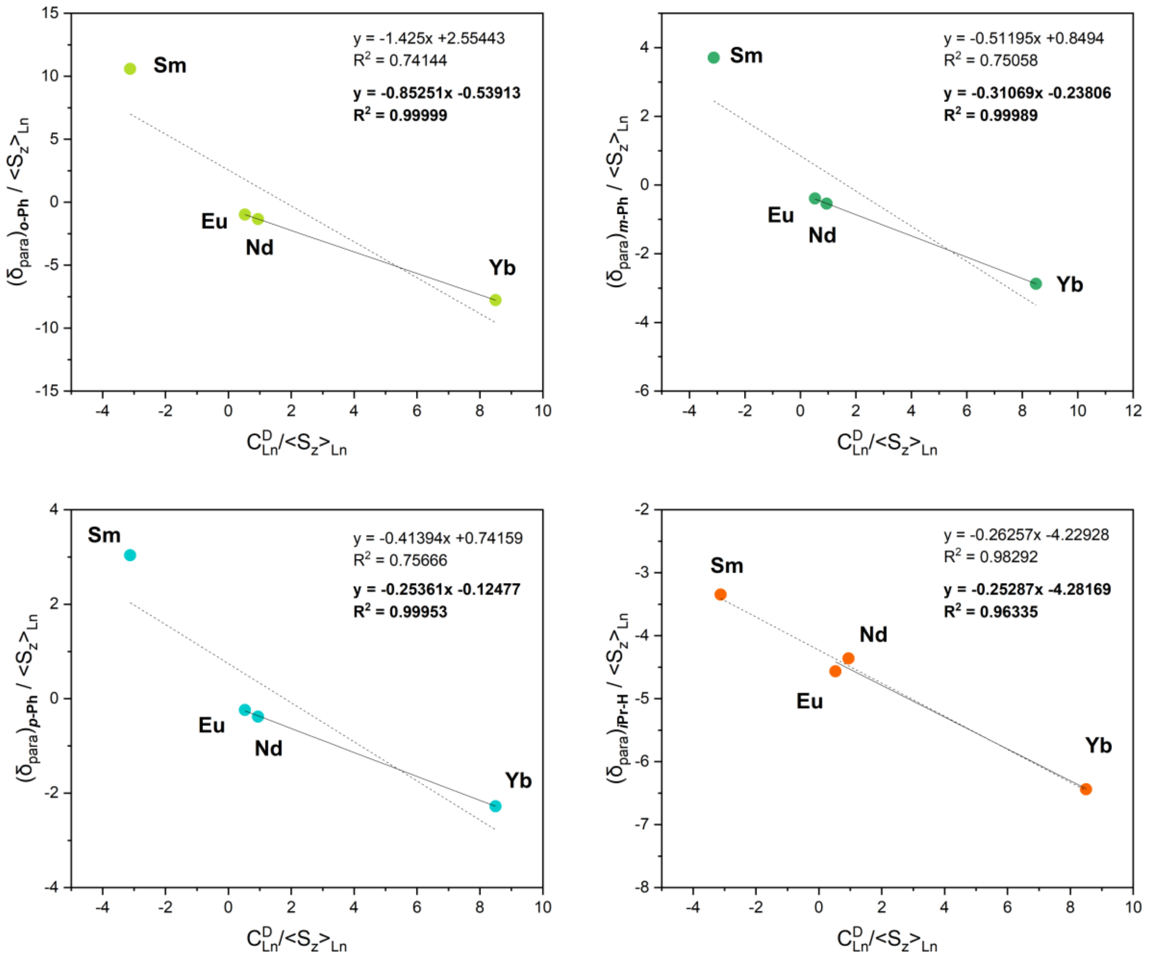

$C_D/\langle S_z\rangle_{303K}$ plot	G_i		Intercept		**Slope**		Statistics	note	Results	
	Value	SE	Value	SE	Value	SE	R^2		$A_2^0\langle r^2\rangle$	Avg
**H3 (*o*-Ph)**	-0.006369	0.000290	-0.53913	0.0076	-0.85251	0.00154	>0.99	w/o Sm	134	
**H4 (*m*-Ph)**	-0.002360	0.000053	-0.23806	0.01118	-0.31069	0.00226	>0.99	w/o Sm	132	134±3
**H5 (*p*-Ph)**	-0.001846	0.000004	-0.12477	0.0193	-0.25361	0.0039	>0.99	w/o Sm	137	
**H8 (*i*Pr-H)**	-0.004227	0.000519	-4.28169	0.17087	-0.25287	0.03455	0.96	w/o Sm	1013	

Figure S43. Determination of F_i parameters[42]

$$\frac{\delta_{para}^{i,Ln}}{C_D^{Ln}} = F_i \cdot \frac{\langle S_z \rangle_{Ln}}{C_D^{Ln}} + G_i \cdot A_2^0 \langle r^2 \rangle$$

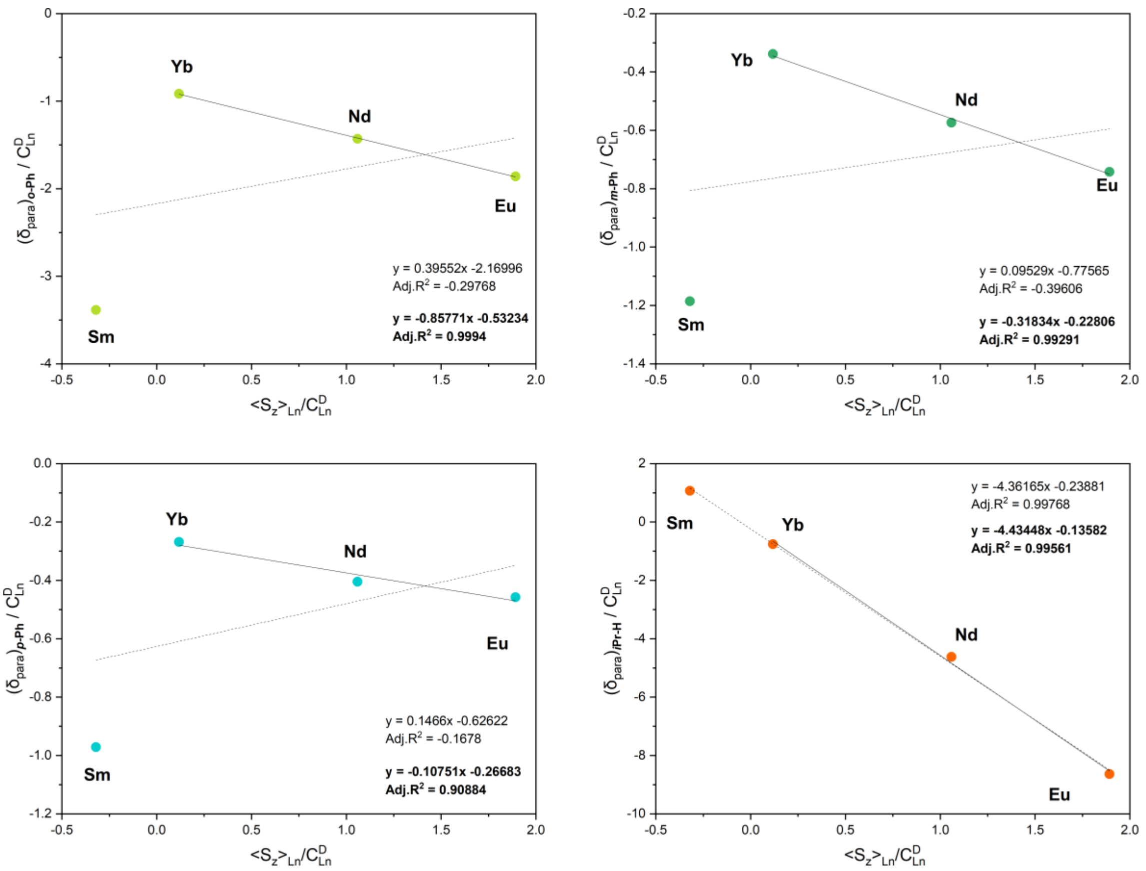

$<S_z>_{303K}/C_D$ plot	G_i		Intercept		**Slope**		Statistics	note	Results
	Value	SE	Value	SE	Value	SE	R^2		F_i
**H3 (*o*-Ph)**	-0.006369	0.000290	-0.85771	0.01159	-0.53234	0.00925	>0.99	w/o Sm	-0.532(9)
**H4 (*m*-Ph)**	-0.002360	0.000053	-0.31834	0.01706	-0.22806	0.0136	>0.99	w/o Sm	-0.228(14)
**H5 (*p*-Ph)**	-0.001846	0.000004	-0.26683	0.02946	-0.10751	0.02349	0.91	w/o Sm	-0.108(23)
**H8 (*i*Pr-H)**	-0.004227	0.000519	-0.13582	0.26078	-4.43448	0.20799	>0.99	w/o Sm	-4.434(208)

Table S17. Calculated contact (δ_{FCS}) and pseudocontact (δ_{PCS}) contributions for the series of *i*Pr_2BA complexes using REILLEY's method.

Nd^{III} (303 K) (ppm)		H3 *o*-Ph^{H}	H4 *m*-Ph^{H}	H5 *p*-Ph^{H}	H8 *i*Pr-H
Exp	δ_{para}	6.00	2.41	1.70	19.38
REILLEY's method	δ_{FCS}	2.37(4)	1.01(6)	0.48(10)	19.7(9)
	δ_{PCS}	3.59(8)	1.33(3)	1.04(2)	2.38(5)
	$\delta_{FCS}+\delta_{PCS}$	5.96	2.35	1.52	22.11
**Sm^{III} (303 K) (ppm)**		**H3 *o*-Ph^{H}**	**H4 *m*-Ph^{H}**	**H5 *p*-Ph^{H}**	**H8 *i*Pr-H**
Exp	δ_{para}	2.37	0.83	0.68	-0.75
REILLEY's method	δ_{FCS}	-0.119(2)	-0.051(3)	-0.024(5)	-0.993(47)
	δ_{PCS}	0.599(13)	0.222(5)	0.174(4)	0.397(9)
	$\delta_{FCS}+\delta_{PCS}$	0.48	0.17	0.15	-0.60
**Eu^{III} (303 K) (ppm)**		**H3 *o*-Ph^{H}**	**H4 *m*-Ph^{H}**	**H5 *p*-Ph^{H}**	**H8 *i*Pr-H**
Exp	δ_{para}	-7.42	-2.97	-1.83	-34.52
REILLEY's method	δ_{FCS}	-4.03(7)	-1.73(10)	-0.81(18)	-33.6(16)
	δ_{PCS}	-3.42(7)	-1.27(3)	-0.99(2)	-2.27(5)
	$\delta_{FCS}+\delta_{PCS}$	-7.45	-2.99	-1.81	-35.8
**Yb^{III} (303 K) (ppm)**		**H3 *o*-Ph^{H}**	**H4 *m*-Ph^{H}**	**H5 *p*-Ph^{H}**	**H8 *i*Pr-H**
Exp	δ_{para}	-20.08	-7.37	-5.82	-16.59
REILLEY's method	δ_{FCS}	-1.38(2)	-0.59(4)	-0.28(6)	-11.5(5)
	δ_{PCS}	-18.8(4)	-6.97(15)	-5.45(12)	-12.5(3)
	$\delta_{FCS}+\delta_{PCS}$	-20.2	-7.56	-5.73	-24.0

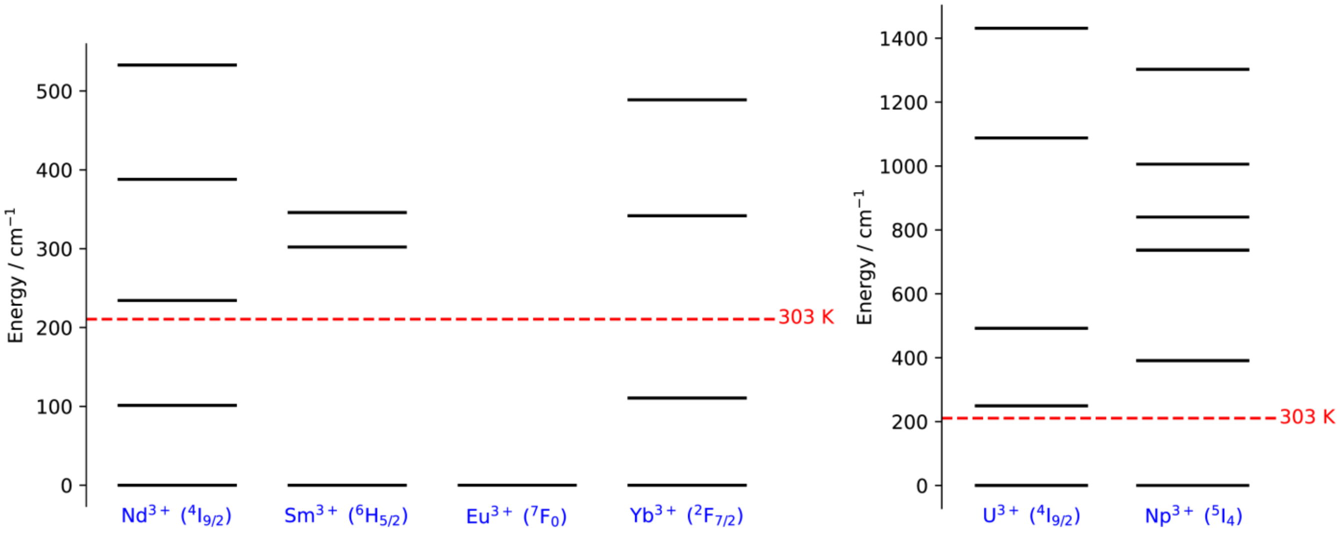

Figure S44. Energy splitting of the ground terms of [Ln^{III}/An^{III}(*i*Pr_2BA)$_3$] **2−5, 7, 8** due to the ligand field, derived from the CASSCF calculation.

6. Magnetism studies

6.1. *Ab initio* calculations details

The following table shows the active spaces for all considered lanthanides and actinides and respective spin multiplicities that were taken into consideration.

Table S18. Composition of active spaces used for the CAS calculations of selected [An^{III}/Ln^{III}(*i*Pr_2BA)$_3$] complexes.

Ln^{III} / An^{III}	Active space	Multiplicities and number of roots
Nd	CAS[3,7]	35 quartets, 112 doublets
Sm	CAS[5,7]	21 sextets, 224 quartets, 490 doublets
Eu	CAS[6,7]	7 septets, 140 quintets, 588 triplets, 490 singlets
Yb	CAS[13,7]	7 doublets
Th	CAS[1,13]	13 doublets
Pa	CAS[2,12]	66 triplets, 78 singlets
U	CAS[3,13]	35 quartets, 112 doublets
Np	CAS[4,12]	35 quintets, 210 triplets, 196 singlets
Pu	CAS[5,7]	21 sextets, 224 quartets, 490 doublets

6.2. Comparison of DFT- and CAS-NAO derived electron configurations

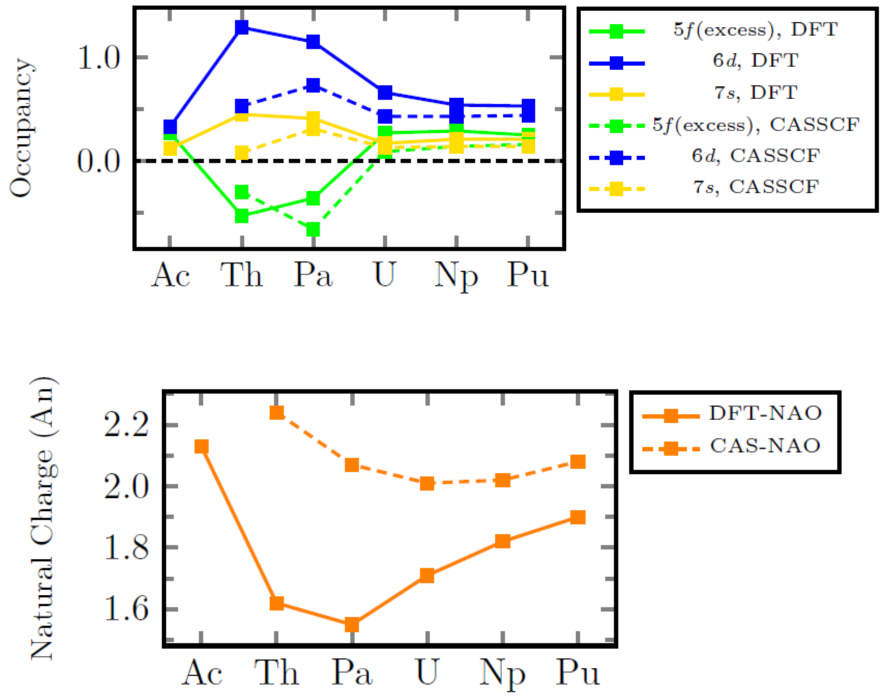

Figure S45. Comparison of DFT- and CAS-NAO derived electron configurations

6.3. Energy diagrams and MO plots of extended active space cases

The following diagrams (**Figure S46**) show the resulting CAS MO of the extended active space calculations for the cases from Th to Np sorted according to energy together with the associated state-averaged occupancy presented in parentheses. The solid lines composed of blue and red color represent the MO energy levels, and the proportion of each color indicates the relative percentage contributions of the *f* and *d* Löwdin atomic orbitals to the MO, respectively. The series demonstrates the strong hybridization of 5*f* and 6*d* orbitals for cases like Th and Pa (also mirrored by the same occupational numbers) and the subsequent increasing energetic separation of 5*f* and 6*d* and simultaneous decreasing contribution of 6*d* orbitals.

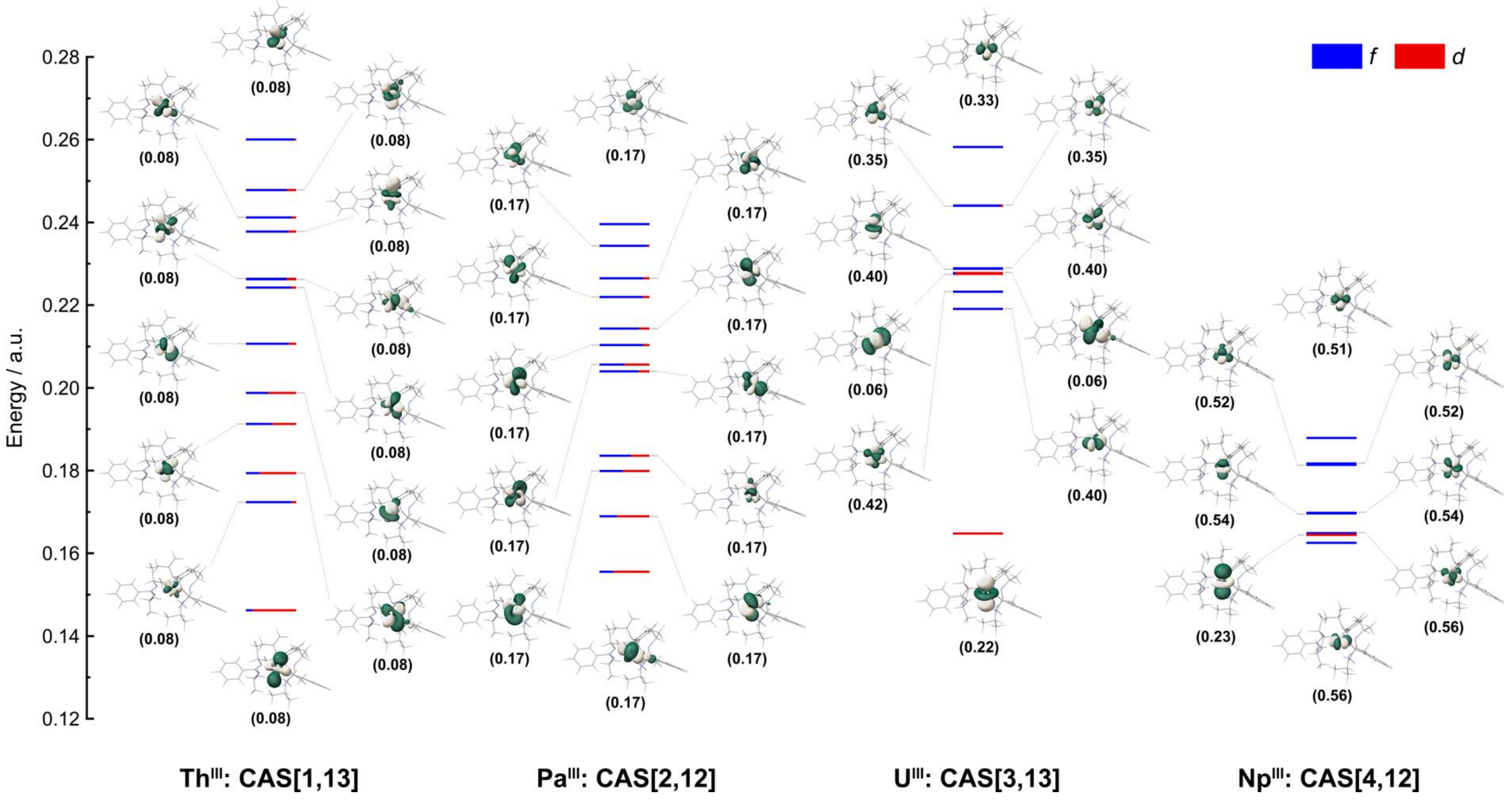

Figure S46. Energy diagrams and MO plots of [An(*i*Pr$_2$BA)$_3$] complexes (An = Th, Pa, U, Np). Iso-surface = 0.05.

Raw Orca CAS χ tensors from CASSCF calculations

The following molar susceptibility tensors are given in $cm^3 \cdot K/mol$ (T = 303 K).

Ln(III)	An(III)
**Nd**<br>chi_Nd=<br>[1.634355 -0.017288 -0.001322<br>-0.017288 1.640758 -0.005538<br>-0.001322 -0.005538 1.219590]	**Th**<br>chi_Th=<br>[0.255533 0.000067 0.000076<br>0.000067 0.255290 0.000010<br>0.000076 0.000010 0.348778]
**Sm**<br>chi_Sm=<br>[0.440199 -0.000855 -0.003628<br>-0.000855 0.461871 0.003931<br>-0.003628 0.003931 0.226615]	**Pa**<br>chi_Pa=<br>[0.249123 -0.002026 -0.004548<br>-0.002026 0.258042 -0.012312<br>-0.004548 -0.012312 0.136250]
**Eu**<br>chi_Eu=<br>[1.793654 -0.004651 0.038377<br>-0.004651 1.414495 -0.004276<br>0.038377 -0.004276 1.411419]	**U**<br>chi_U=<br>[0.707022 0.001321 -0.117169<br>0.001321 1.267411 -0.005319<br>-0.117169 -0.005319 1.239671]
**Yb (CASSCF)**<br>chi_Yb=<br>[1.910114 -0.005719 -0.039056<br>-0.005719 1.921860 -0.029297<br>-0.039056 -0.029297 3.406370]	**Np**<br>chi_Np=<br>[0.559480 0.000184 0.000352<br>0.000184 0.559333 0.000383<br>0.000352 0.000383 0.478081]
**Yb (CAS+NEVPT2)**<br>chi_Yb=<br>[1.976817 -0.002155 -0.058678<br>-0.002155 1.979378 -0.049833<br>-0.058678 -0.049833 3.021517]	**Pu**<br>chi_Pu=<br>[0.179272 -0.000249 0.004598<br>-0.000249 0.176085 0.002076<br>0.004598 0.002076 0.155446]

Ellipsoid plots of traceless χ tensors

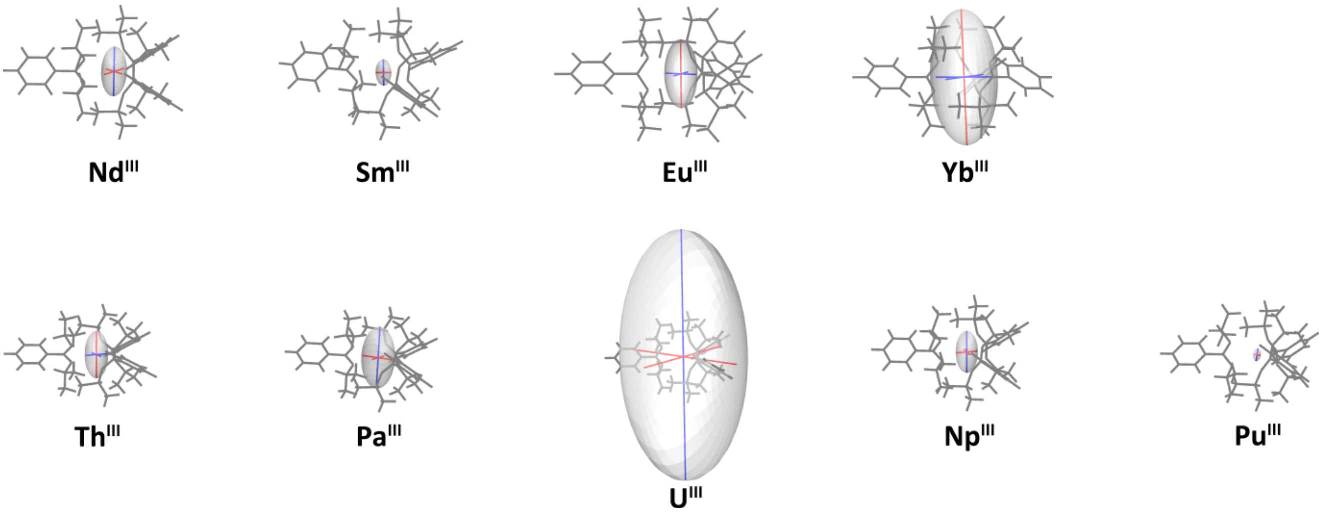

Figure S47. Spheroid representations of the traceless χ tensors of respective Ln^{III} and An^{III} complexes, where the three principal axes correspond to the eigenvalues of the tensor. Negative eigenvalues are plotted in blue, positive values are red. The correlation between the sign of an eigenvalue associated with a certain axis and the sign of the respective lobe of the PCS field can be readily recognized. The spheroids are scaled by an arbitrary factor for better visual inspection. Said factor for the An^{III} plots is **five times** the factor of the Ln^{III} plots. Therefore, these plots are solely meant for relative evaluation within the respective series.

Table S19. Traceless χ tensors derived from CASSCF calculations (in units of 10^{-30} m^3, SI units) and their computed eigenvalues. The table also includes the axiality ($\Delta\chi_{ax}$) and rhombicity ($\Delta\chi_{rh}$) of magnetic susceptibility tensors (in units of 10^{-32} m^3)**.**

		Nd^{III}	Sm^{III}	Eu^{III}	Yb^{III}		U^{III}	Np^{III}
	T	303 K	303 K	303 K	303 K	303 K[a)]	303 K	303 K
traceless susceptibility tensor	xx	0.0094	0.0044	0.0175	-0.0346	-0.0240	-0.0251	0.0019
	xy	-0.0012	-0.0001	-0.0003	-0.0004	-0.0001	0.0001	0.0000
	xz	-0.0001	-0.0002	0.0026	-0.0027	-0.0040	-0.0081	0.0000
	yx	-0.0012	-0.0001	-0.0003	-0.0004	-0.0001	0.0001	0.0000
	yy	0.0098	0.0059	-0.0086	-0.0338	-0.0239	0.0135	0.0019
	yz	-0.0004	0.0003	-0.0003	-0.0020	-0.0034	-0.0004	0.0000
	zx	-0.0001	-0.0002	0.0026	-0.0027	-0.0040	-0.0081	0.0000
	zy	-0.0004	0.0003	-0.0003	-0.0020	-0.0034	-0.0004	0.0000
	zz	-0.0192	-0.0103	-0.0088	0.0684	0.0479	0.0116	-0.0037
Eigenvalue	χ_{xx}	0.0084	0.0044	-0.0085	-0.0337	-0.0238	0.0130	0.0019
	χ_{yy}	0.0108	0.0059	-0.0092	-0.0349	-0.0245	0.0138	0.0019
	χ_{zz}	-0.0192	-0.0103	0.0177	0.0685	0.0483	-0.0268	-0.0037
axiality and rhombicity	$\Delta\chi_{ax}$	-2.8793	-1.5468	2.6619	10.281	7.244	-4.0182	-0.5601
	$\Delta\chi_{rh}$	-0.121	-0.0749	0.0351	0.0614	0.036	-0.0392	-0.0014

[a)] results from CAS+NEVPT2

Pseudocontact Shift Calculations

Figure S48. Comparison of PCS values: calculated using KUPROV's equation versus those obtained from the point dipole model for lanthanide complexes at 303 K.

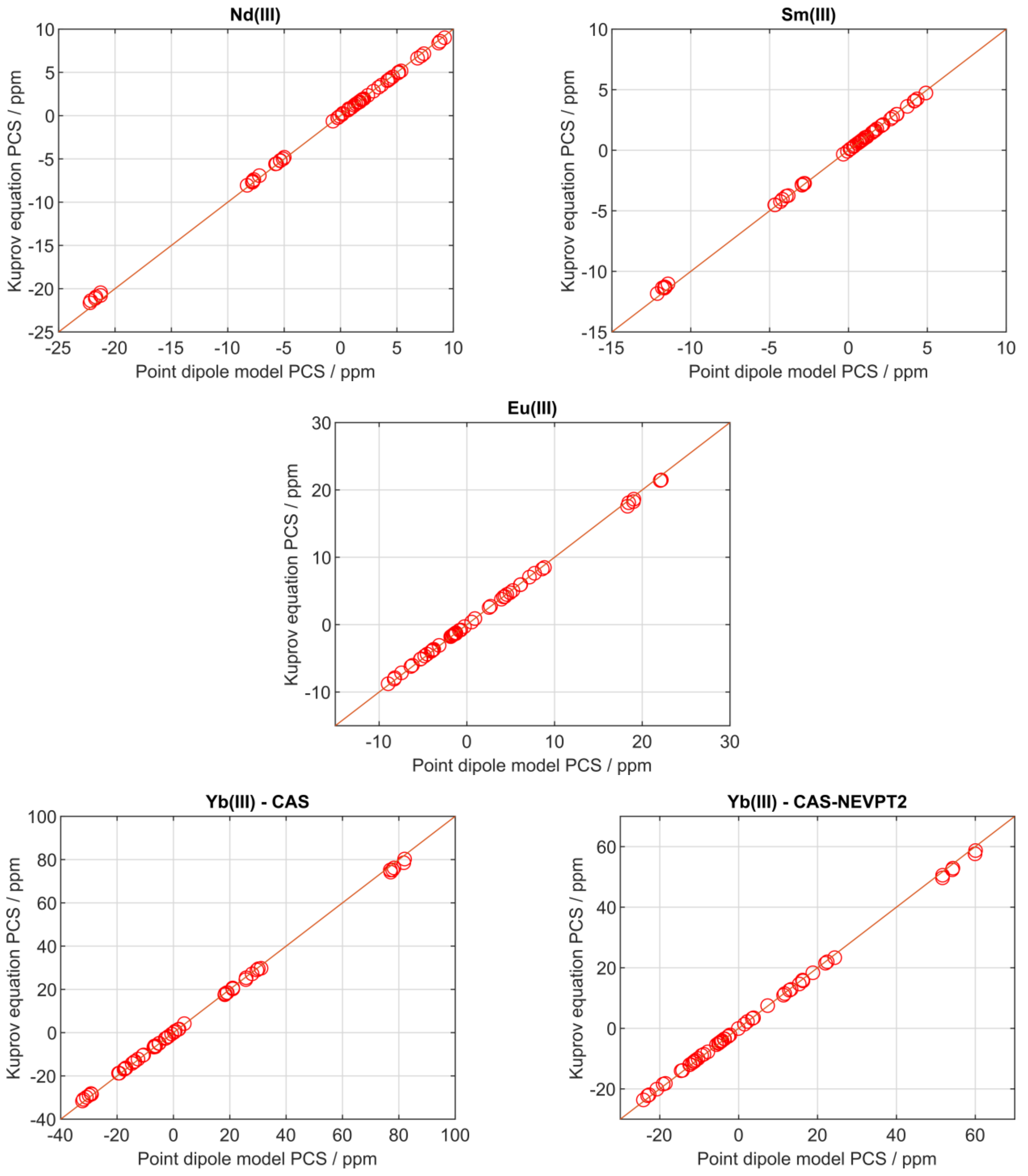

Figure S49. Comparison of PCS values: calculated using KUPROV's equation versus those obtained from the point dipole model for actinide complexes at 303 K.

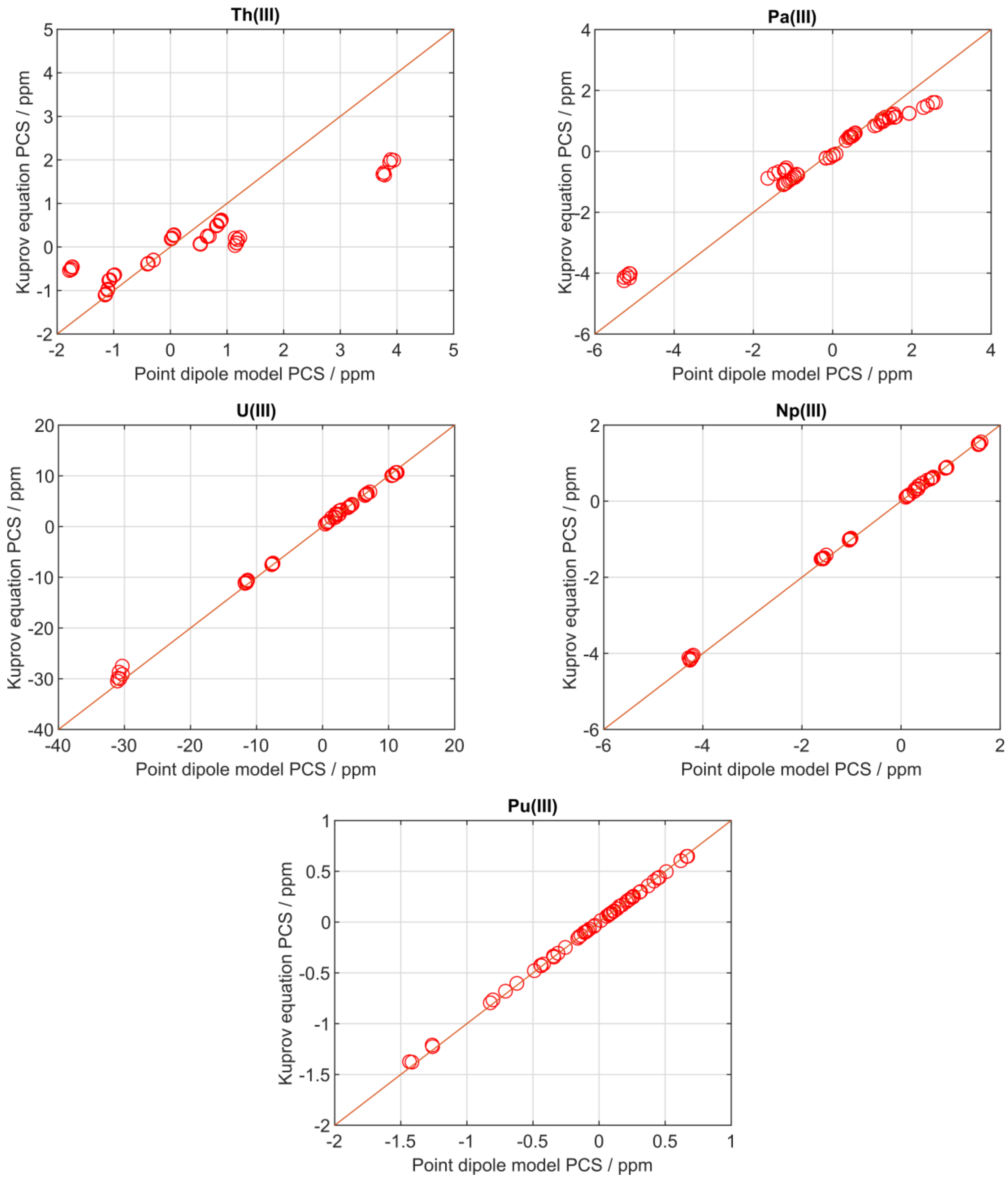

Table S20. Comparison of axiality of magnetic susceptibility tensors derived from experimental separation methods (best fit tensors) and CASSCF calculations at 303 K.

	(10^{-32} m^3)	Nd^{III}	Sm^{III}	Eu^{III}	Yb^{III}		U^{III}	Np^{III}
BLEANEY	$\Delta\chi_{ax}^{fit}$	-1.242	0.748	1.878	3.365		0.978	-2.737
REILLEY	$\Delta\chi_{ax}^{fit}$	-2.155	-0.359	2.052	11.286		-	-
Calculation	$\Delta\chi_{ax}$	-2.879	-1.547	2.662	10.281 (CAS)	7.244 (NEVPT2)	-4.018	-0.560

7. NMR spectra

7.1. Ligand

H*i*Pr_2BA

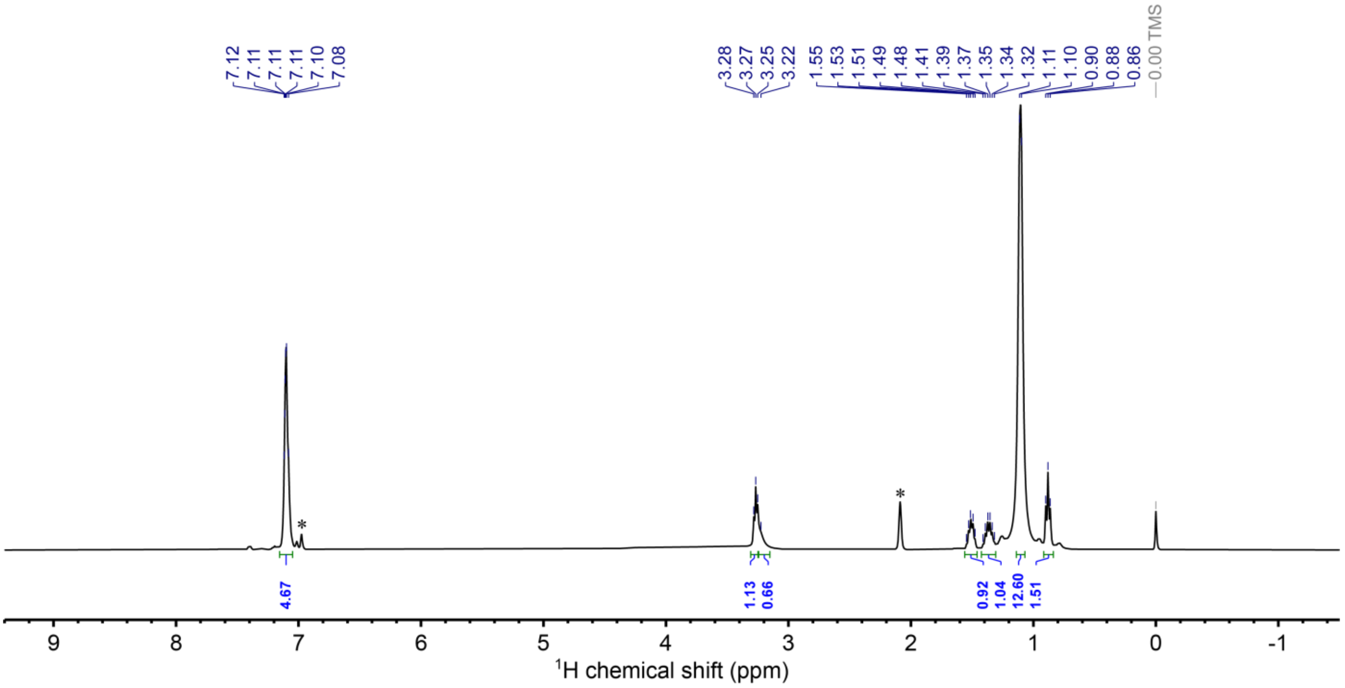

Figure S50. ^{1}H-NMR spectrum of H*i*Pr_2BA in toluene-d_8 (indicated by asterisks) at 303 K.

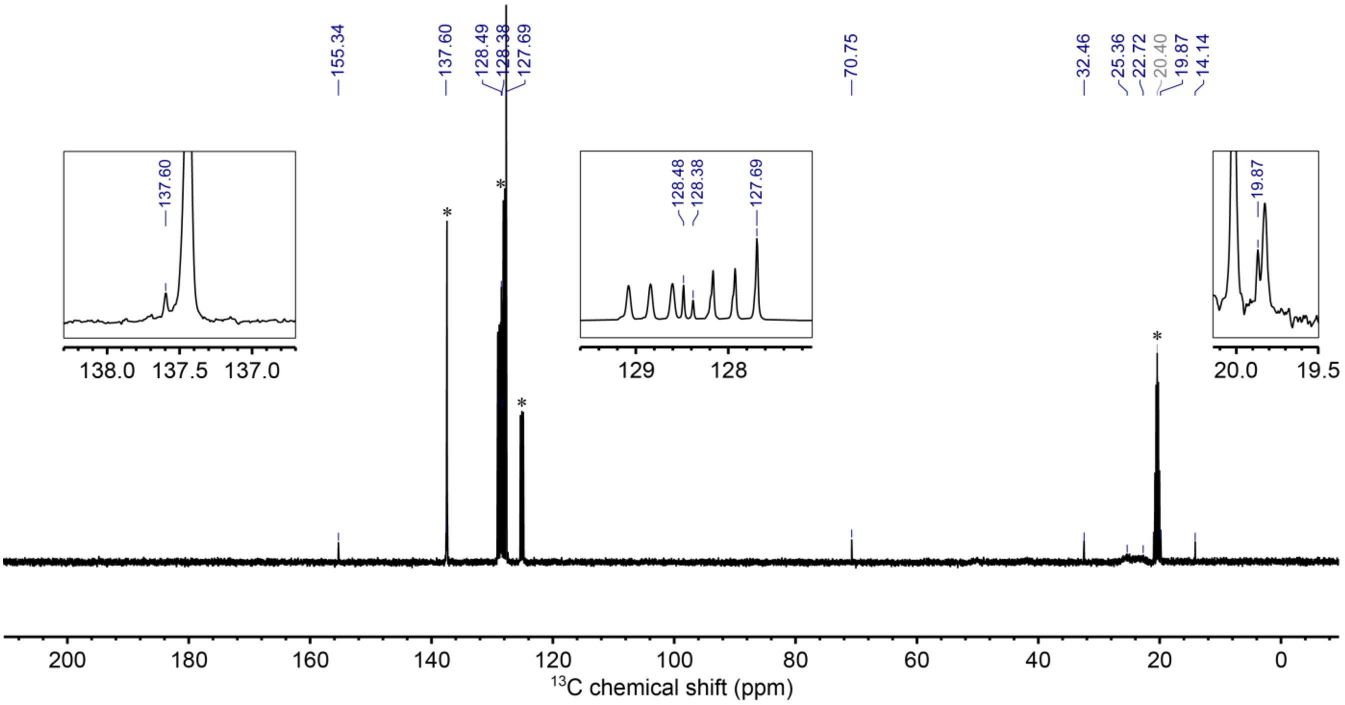

Figure S51. ^{13}C-NMR spectrum of H*i*Pr_2BA in toluene-d_8 (indicated by asterisks) at 303 K. A scaled-up version of the compound peaks is shown as inserts above the main spectrum for clarity.

K-*i*Pr$_2$BA

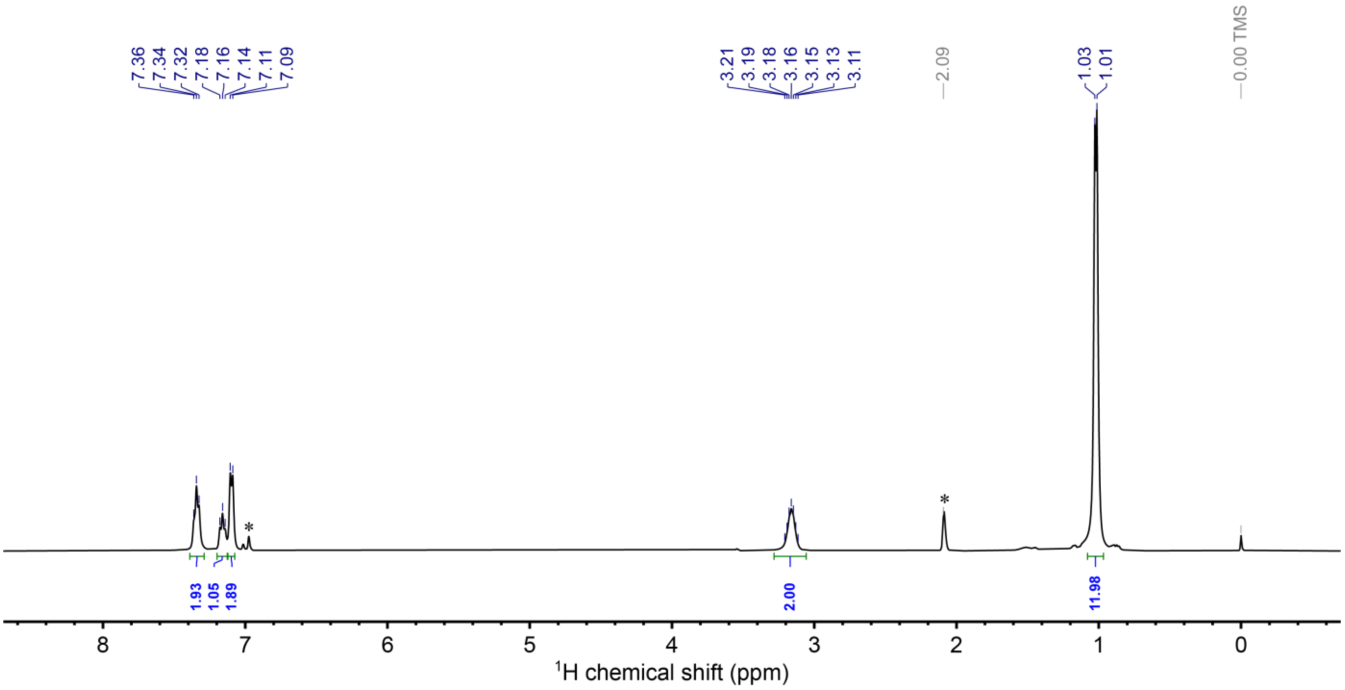

Figure S52. ^{1}H-NMR spectrum of K-*i*Pr$_2$BA in toluene-d_8 (indicated by asterisks) at 303 K.

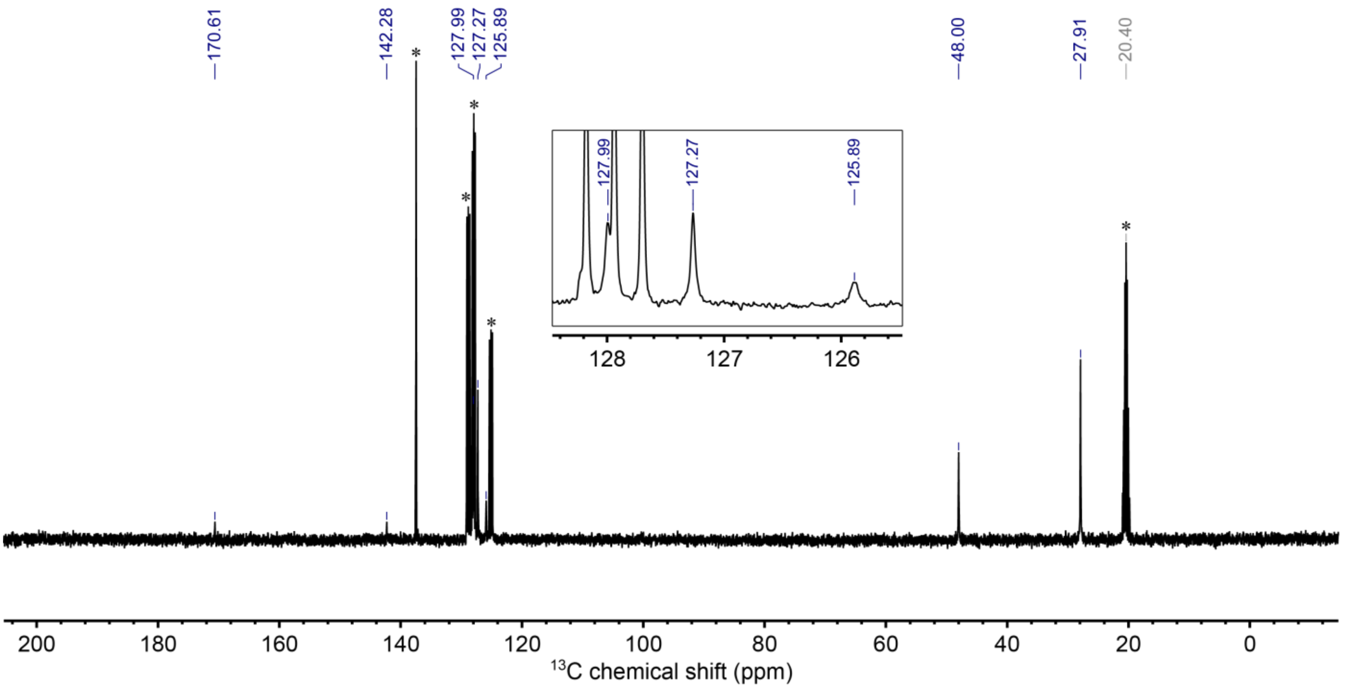

Figure S53. ^{13}C-NMR spectrum of K-*i*Pr$_2$BA in toluene-d_8 (indicated by asterisks) at 303 K. A scaled-up version of the compound peaks is shown as an insert above the main spectrum for clarity.

7.2. Complexes

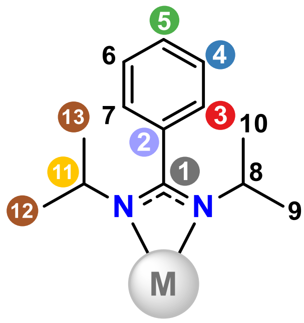

Figure S54. Labeling chart for NMR assignments and analyses**.**

[La(*i*Pr$_2$BA)$_3$] (**1**)

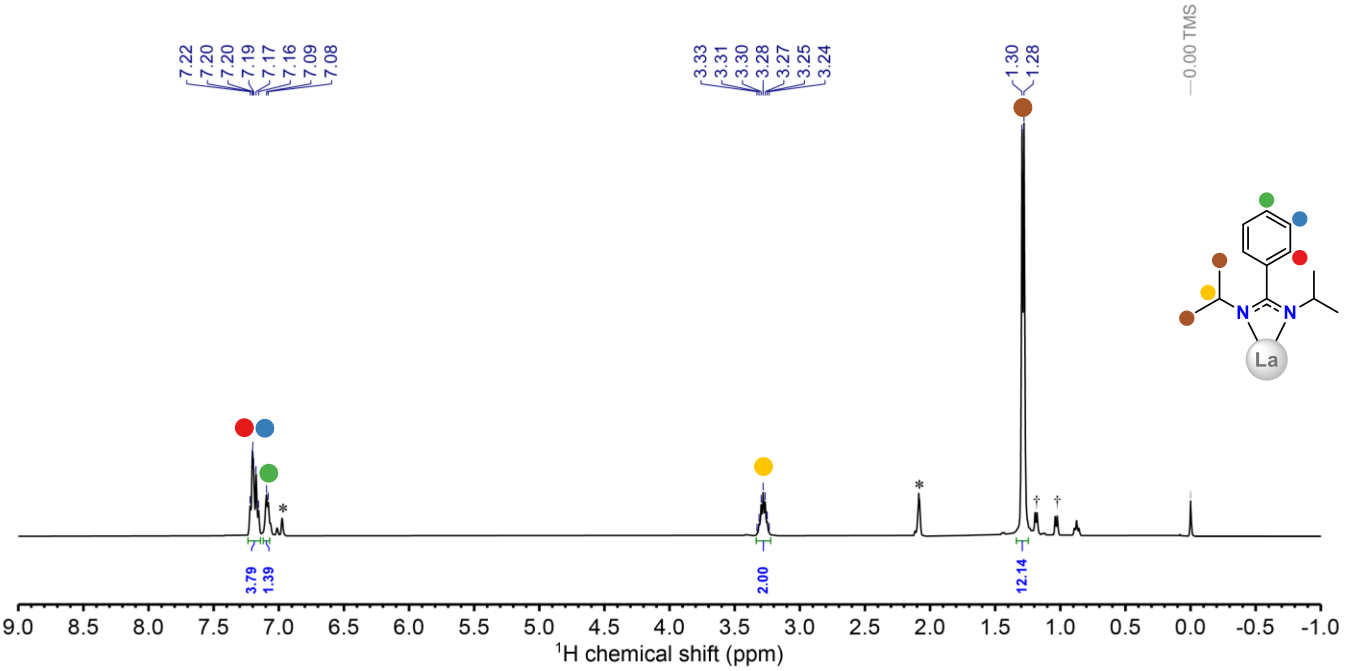

Figure S55. ^{1}H-NMR spectrum of [La(*i*Pr$_2$BA)$_3$] (**1**) in toluene-d_8 (indicated by asterisks) at 303 K. Dagger (†) indicates signals from inseparable free ligand.

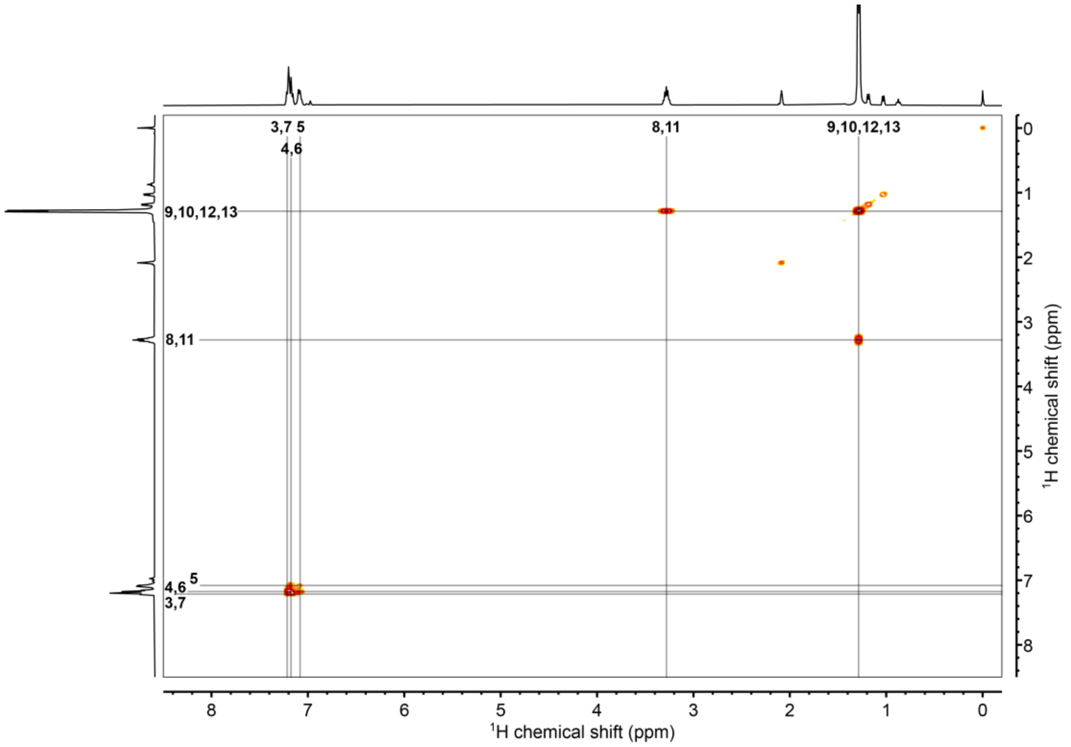

Figure S56. ^{1}H-^{1}H COSY spectrum of [La(*i*Pr$_2$BA)$_3$] (**1**) in toluene-*d*$_8$ at 303 K.

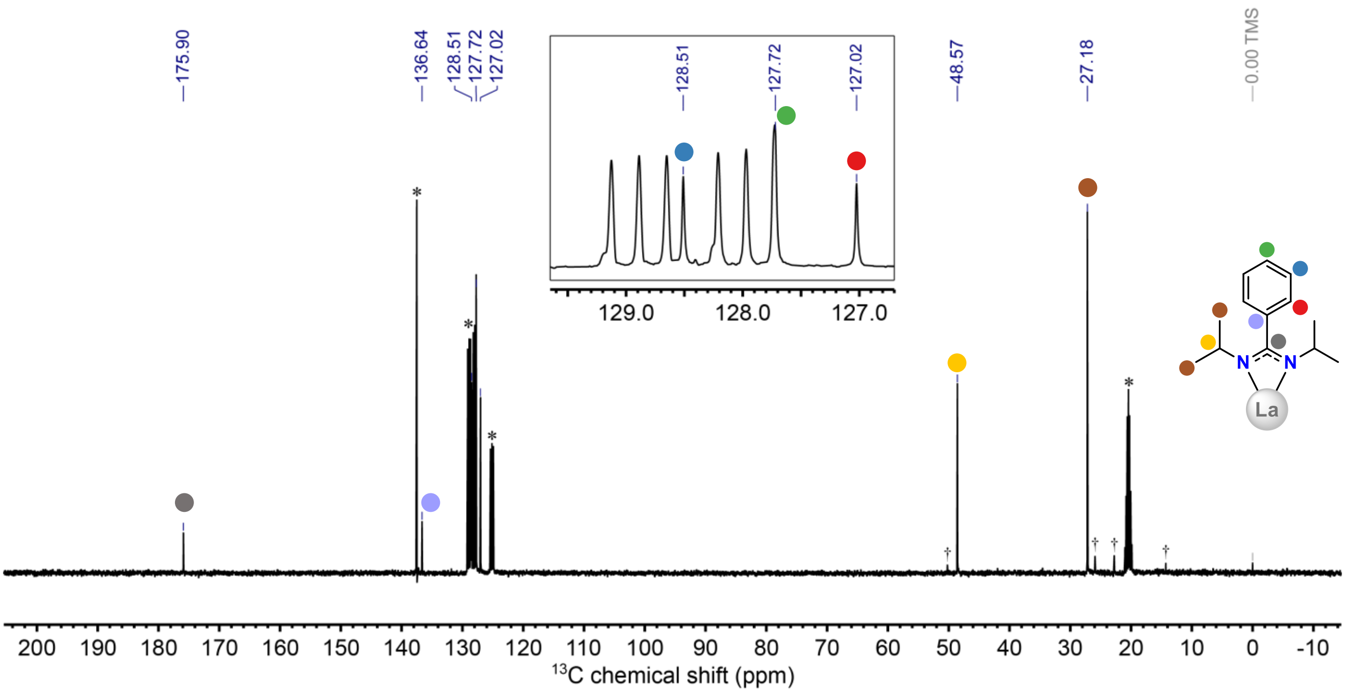

Figure S57. ^{13}C-NMR spectrum of [La(*i*Pr$_2$BA)$_3$] (**1**) in toluene-*d*$_8$ (indicated by asterisks) at 303 K. Dagger (†) indicates signals from inseparable free ligand. A scaled-up version of the compound peaks is shown as an insert above the main spectrum for clarity.

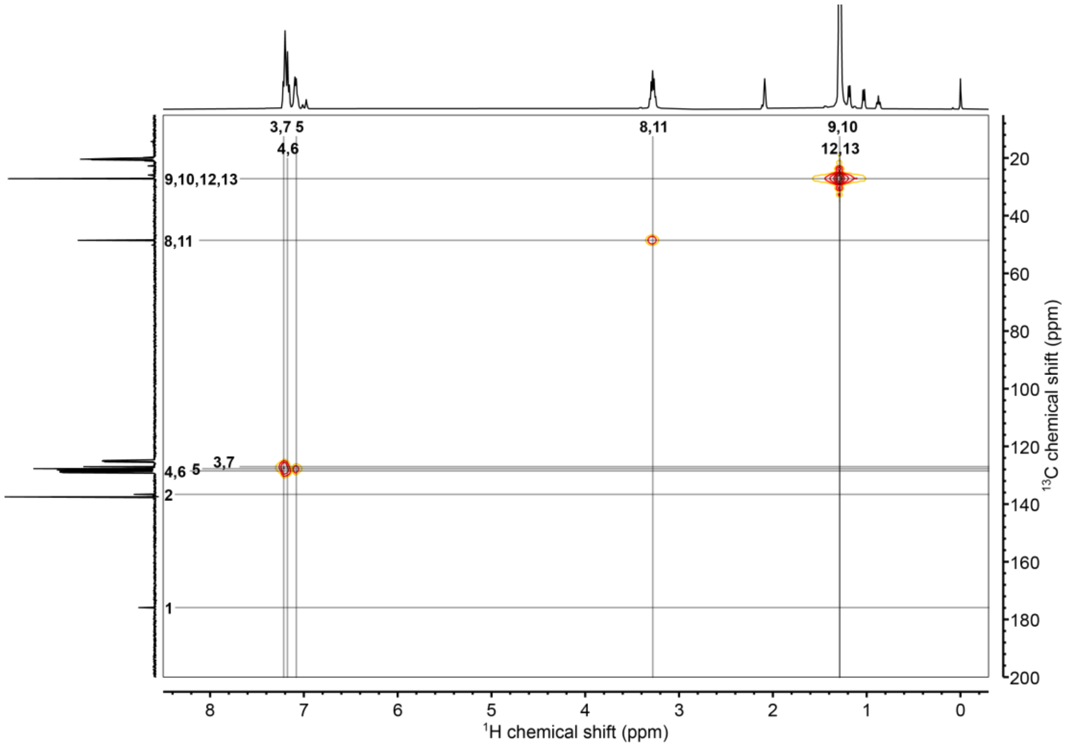

Figure S58. ^{1}H-^{13}C HSQC spectrum of [La(*i*Pr_2BA)$_3$] (**1**) in toluene-d_8 at 303 K.

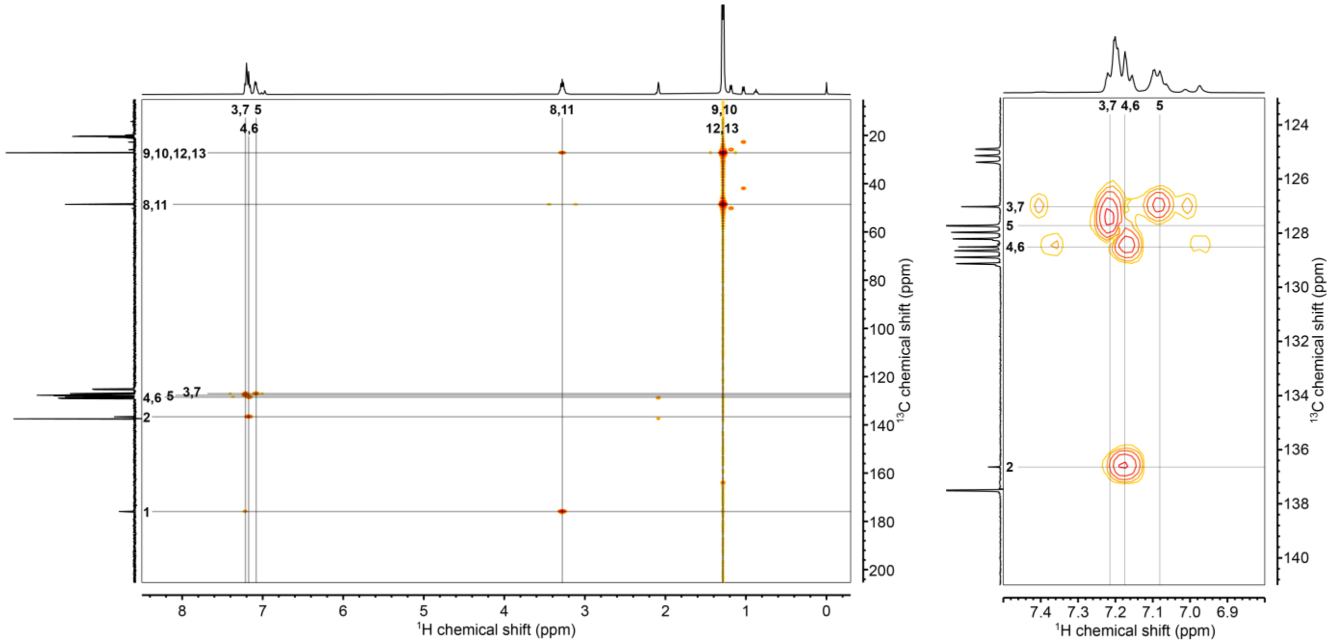

Figure S59. ^{1}H-^{13}C HMBC spectra of [La(*i*Pr_2BA)$_3$] (**1**) in toluene-d_8 at 303 K.

[Nd(iPr$_2$BA)$_3$] (**2**)

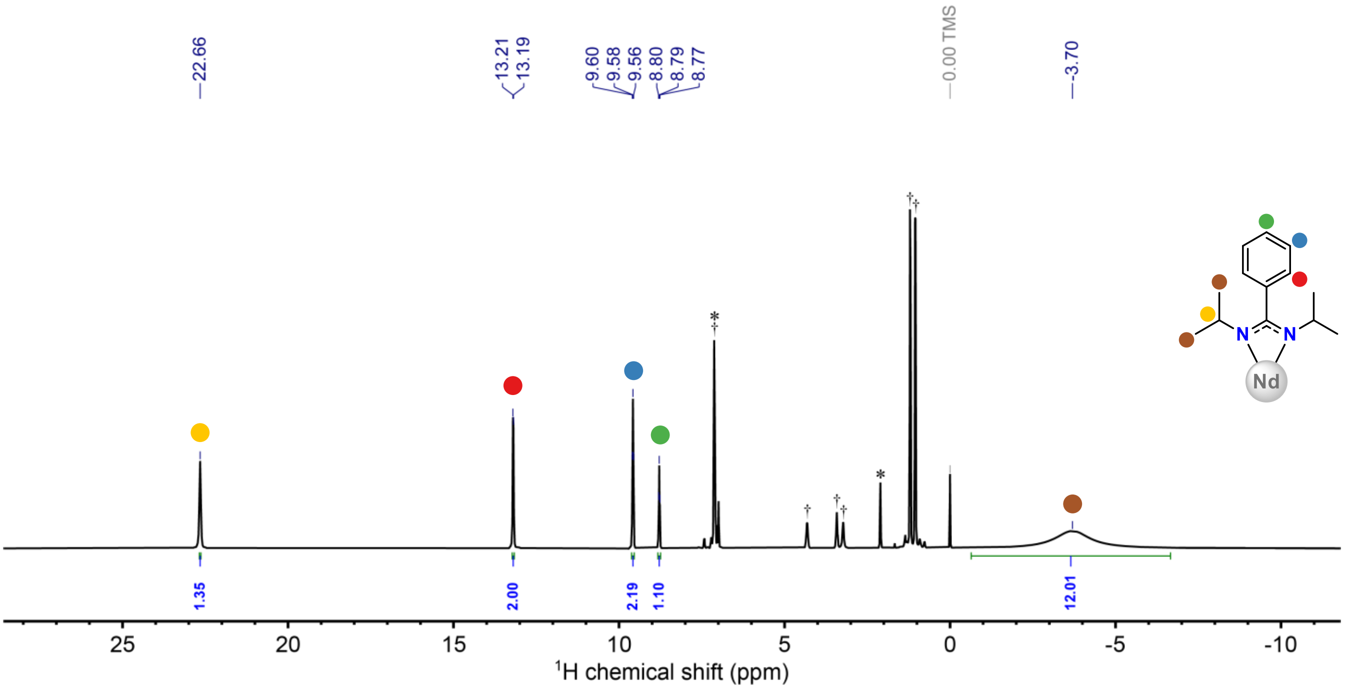

Figure S60. ^{1}H-NMR spectrum of [Nd(iPr$_2$BA)$_3$] (**2**) in toluene-d_8 (indicated by asterisks) at 303 K. Dagger (†) indicates signals from inseparable free ligand.

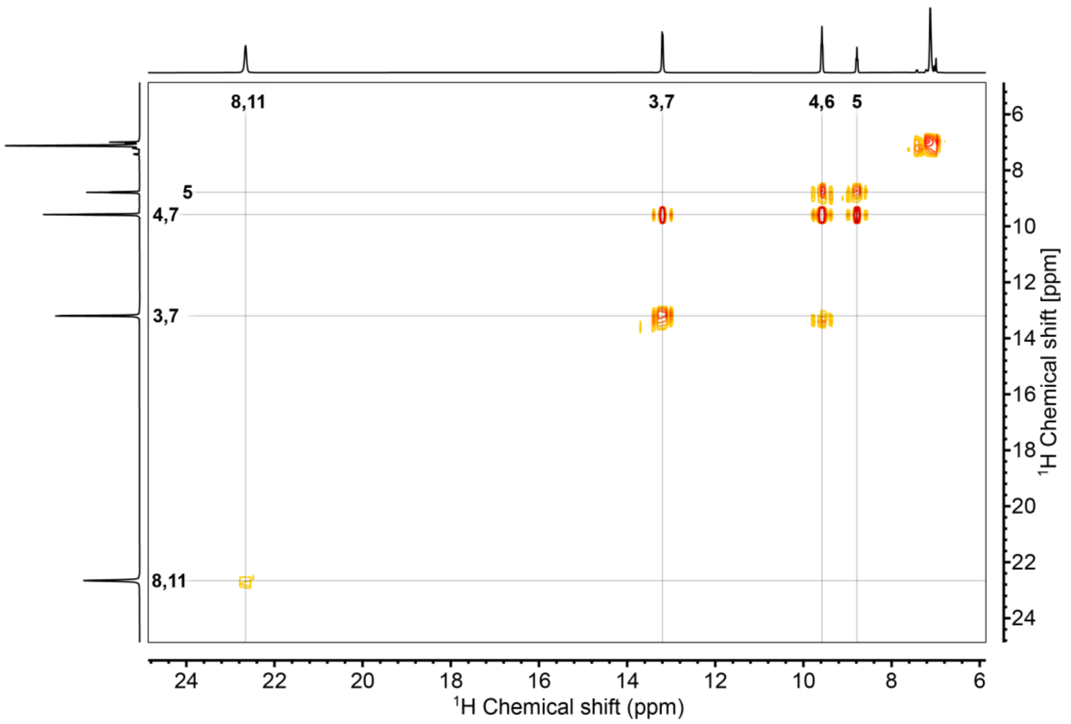

Figure S61. ^{1}H-^{1}H COSY spectrum of [Nd(iPr$_2$BA)$_3$] (**2**) in toluene-d_8 at 303 K.

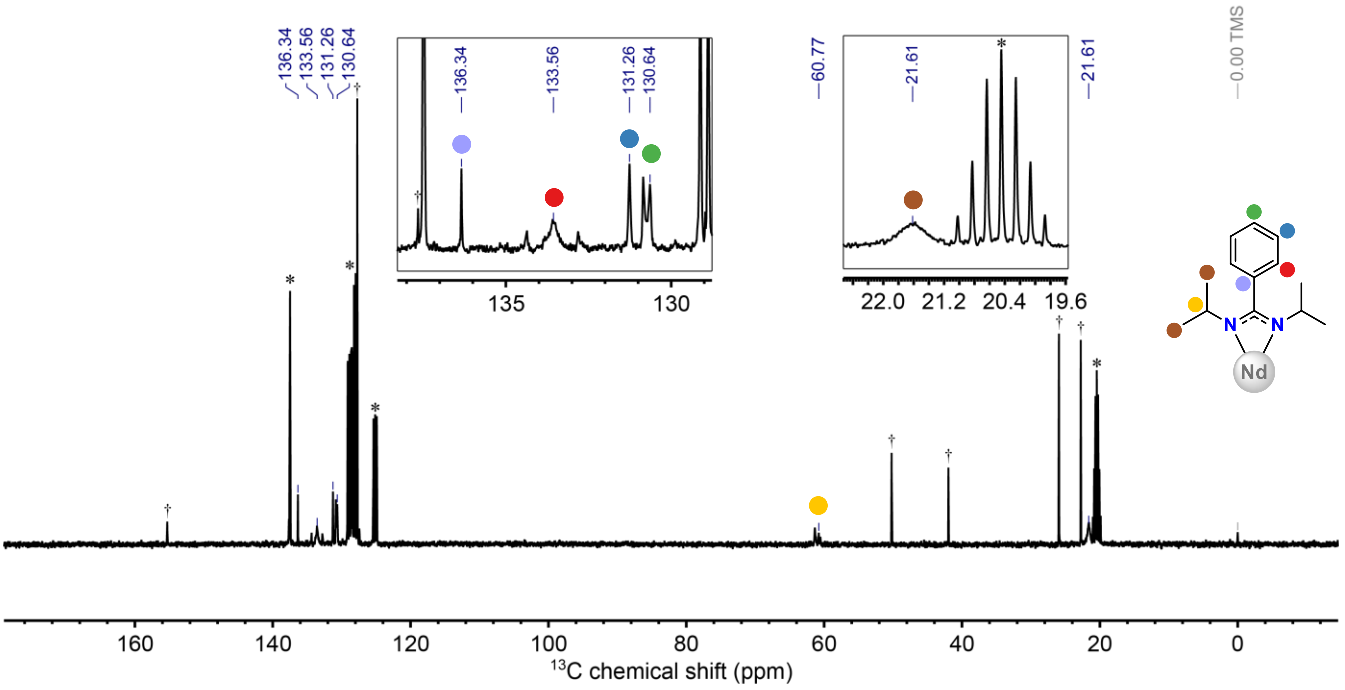

Figure S62. ^{13}C-NMR spectrum of [Nd(*i*Pr_2BA)$_3$] (**2**) in toluene-d_8 (indicated by asterisks) at 303 K. Dagger (†) indicates signals from inseparable free ligand. A scaled-up version of the compound peaks is shown as inserts above the main spectrum for clarity.

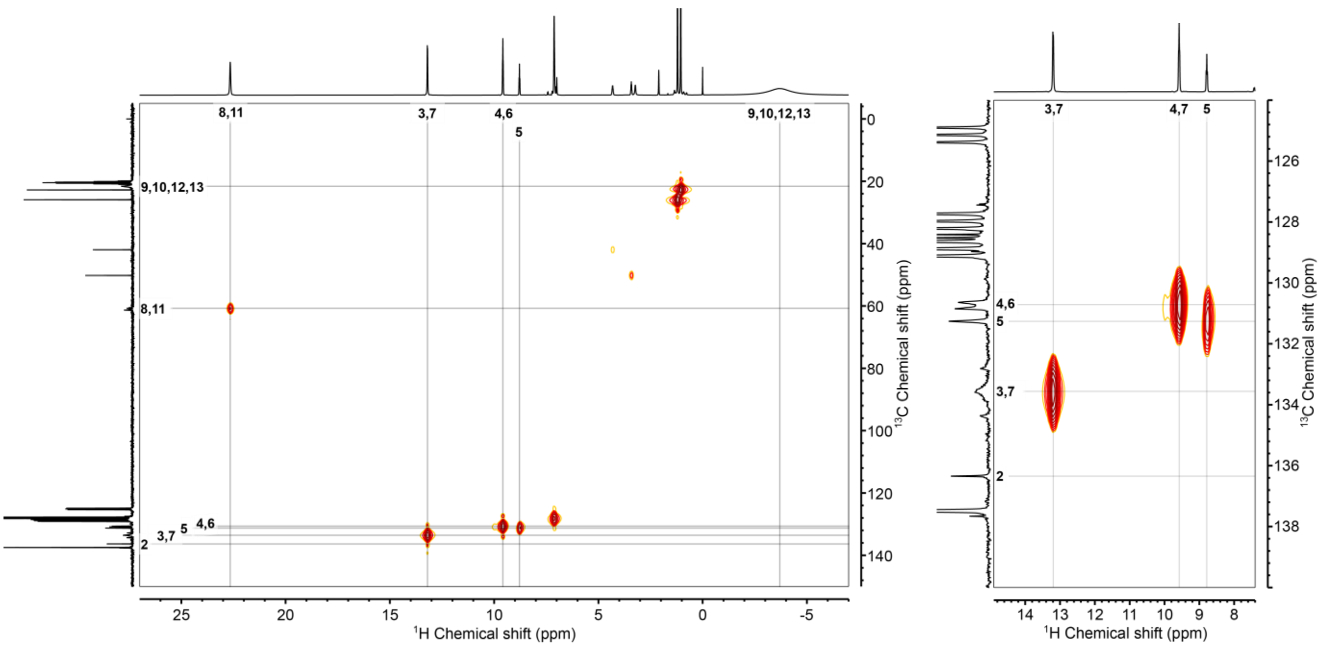

Figure S63. ^{1}H-^{13}C HSQC spectra of [Nd(*i*Pr_2BA)$_3$] (**2**) in toluene-d_8 at 303 K.

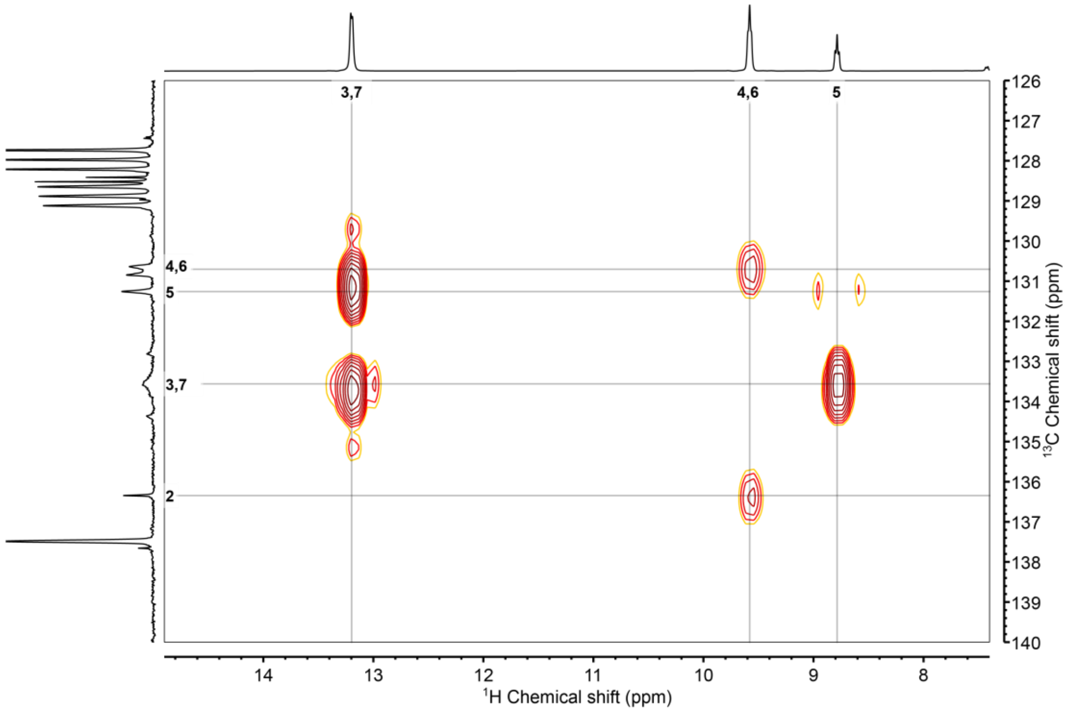

Figure S64. ^{1}H-^{13}C HMBC spectrum of [Nd(iPr$_2$BA)$_3$] (**2**) in toluene-d_8 at 303 K.

[Sm(*i*Pr$_2$BA)$_3$] (**3**)

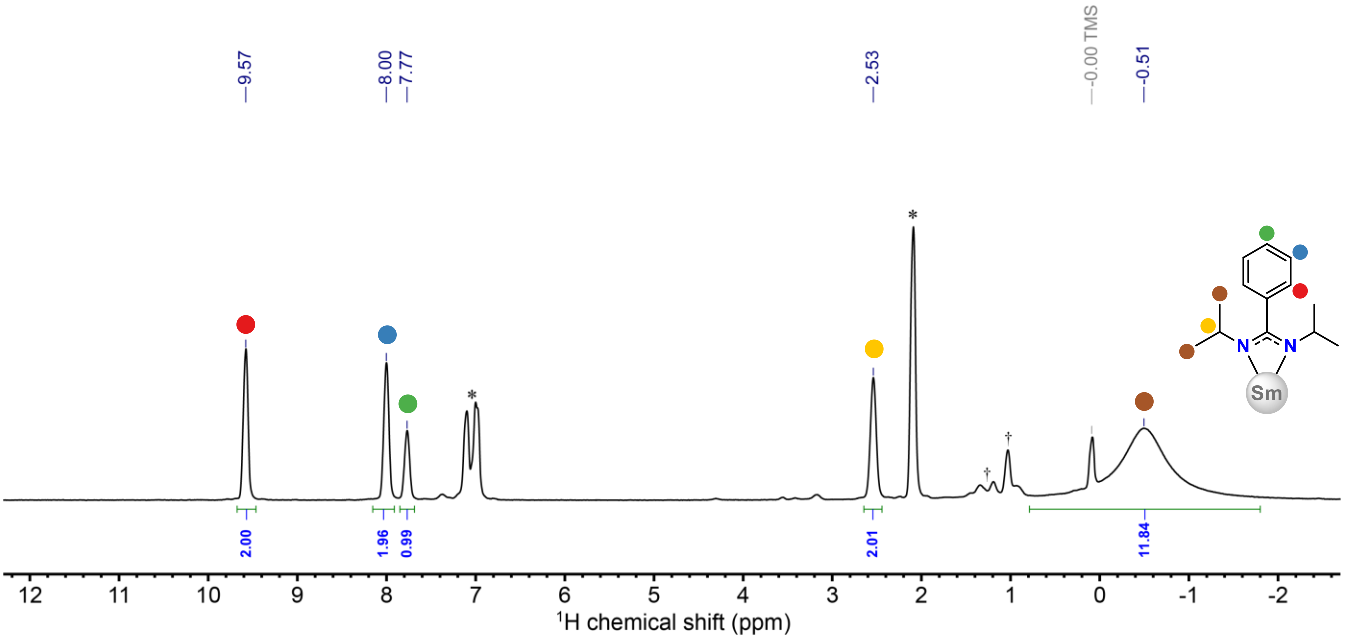

Figure S65. ^{1}H-NMR spectrum of [Sm(*i*Pr$_2$BA)$_3$] (**3**) in toluene-d_8 (indicated by asterisks) at 303 K. Dagger (†) indicates signals from inseparable free ligand.

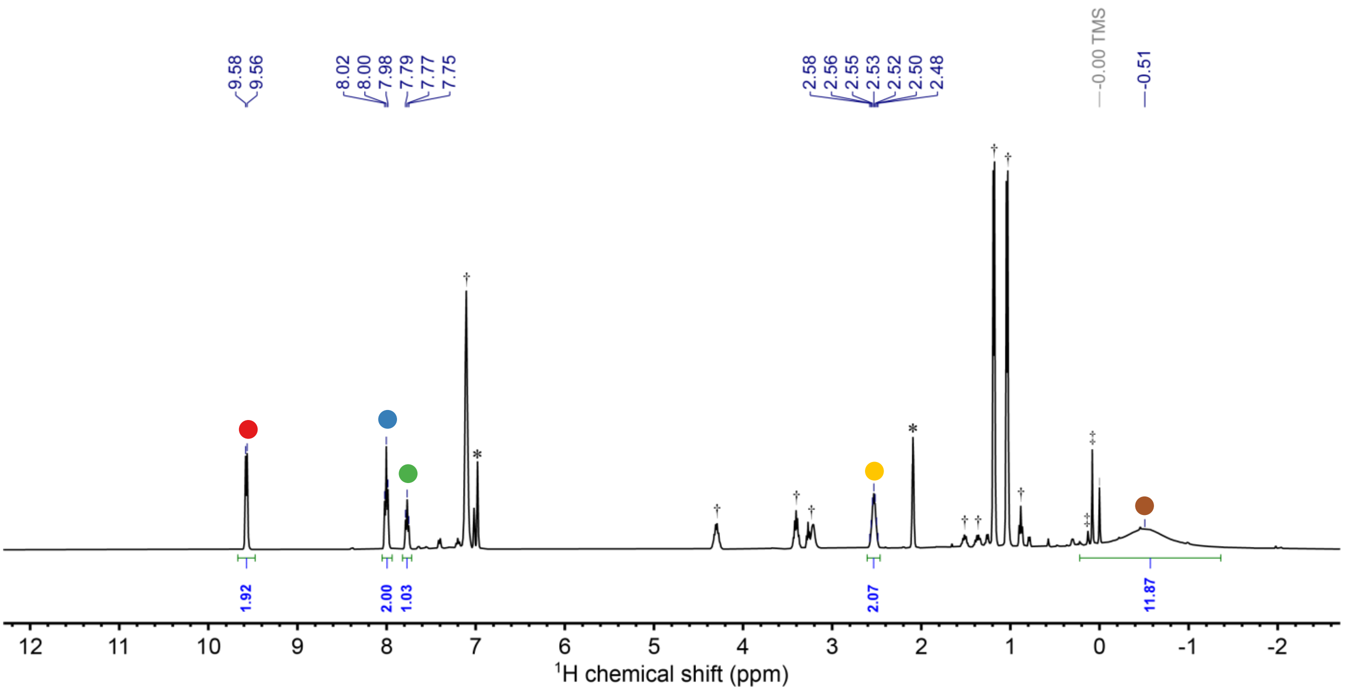

Figure S66. ^{1}H-NMR spectrum of [Sm(*i*Pr$_2$BA)$_3$] (**3**) in toluene-d_8 (indicated by asterisks) at 303 K. Dagger (†) indicates signals from inseparable free ligand. Double dagger (‡) indicates signals from HMDS impurities from base.

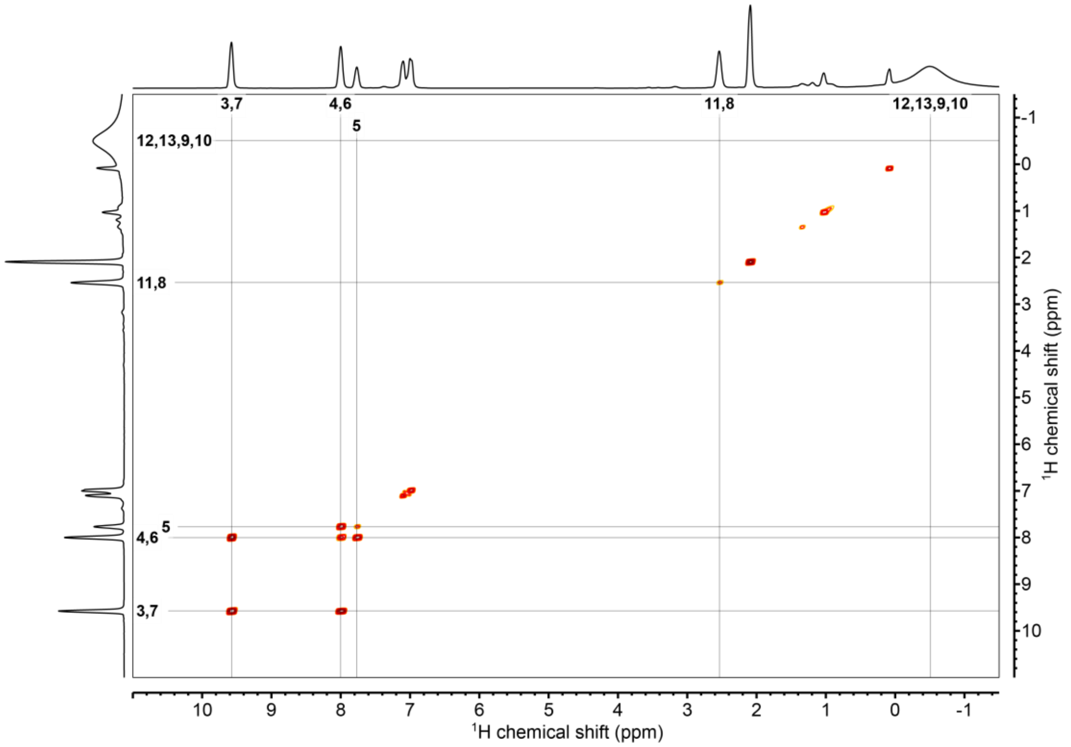

Figure S67. ^{1}H-^{1}H COSY spectrum of [Sm(*i*Pr$_2$BA)$_3$] (**3**) in toluene-*d*$_8$ at 303 K.

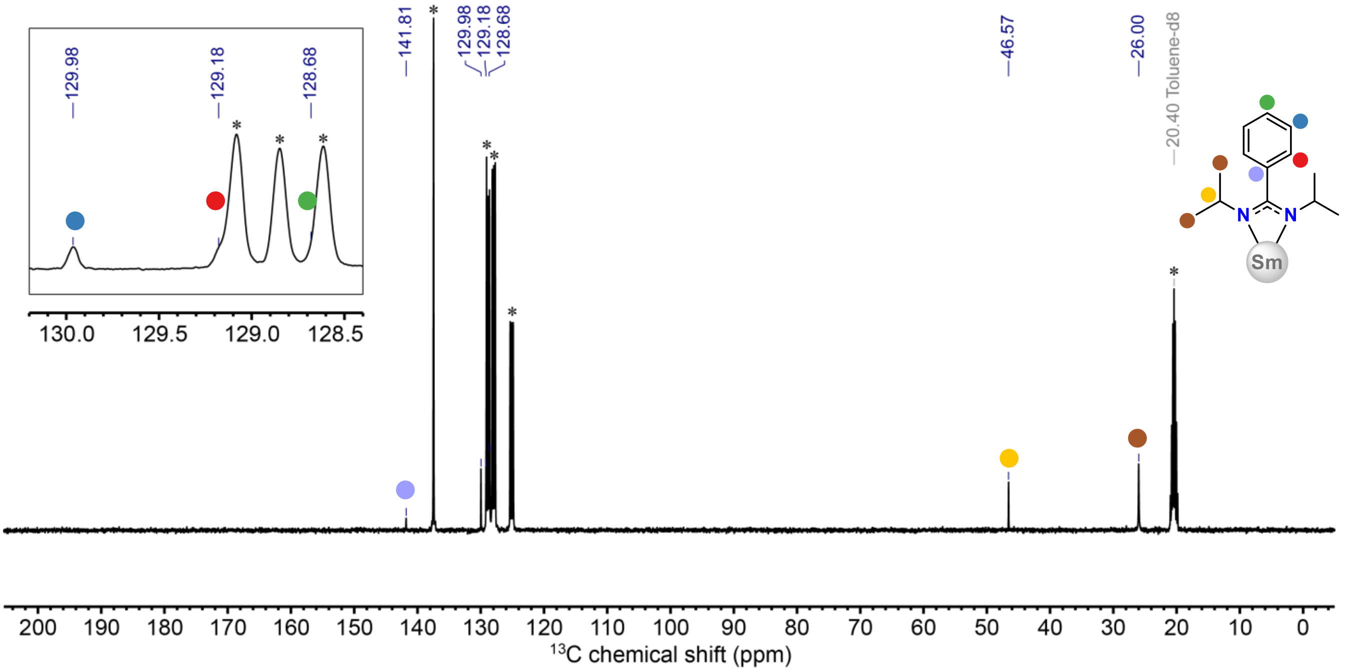

Figure S68. ^{13}C-NMR spectrum of [Sm(*i*Pr$_2$BA)$_3$] (**3**) in toluene-*d*$_8$ (indicated by asterisks) at 303 K. A scaled-up version of the compound peaks is shown as an insert above the main spectrum for clarity.

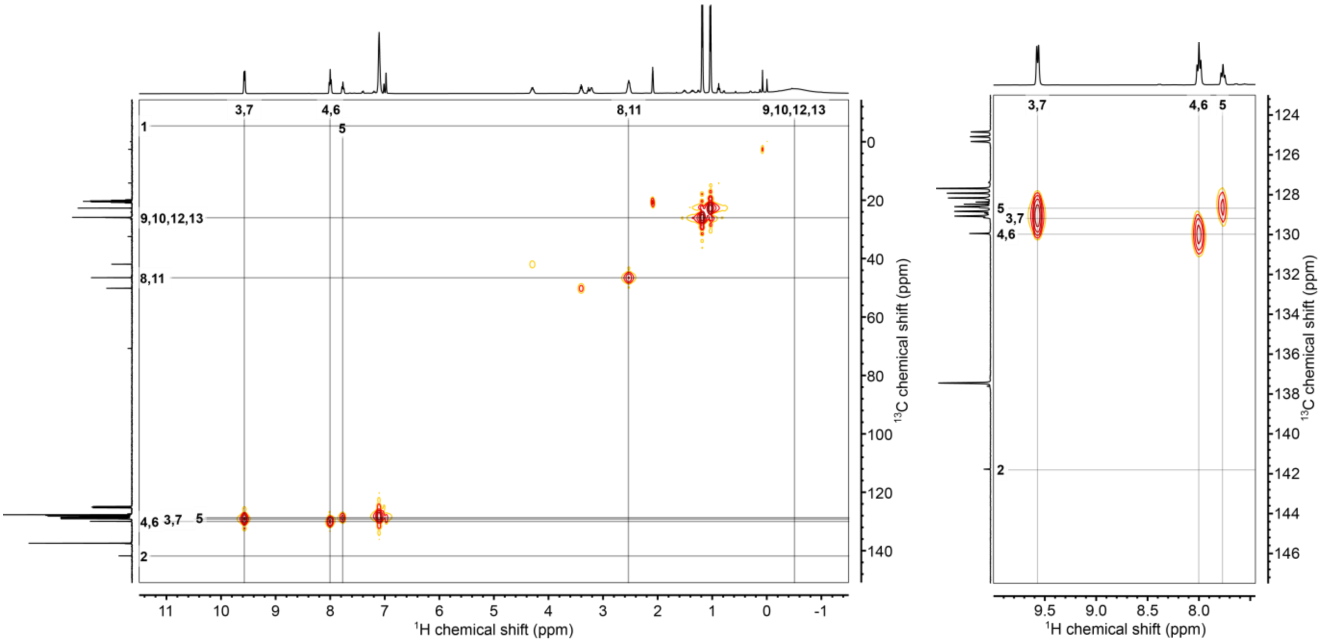

Figure S69. ^{1}H-^{13}C HSQC spectra of [Sm(*i*Pr$_2$BA)$_3$] (**3**) in toluene-d_8 at 303 K.

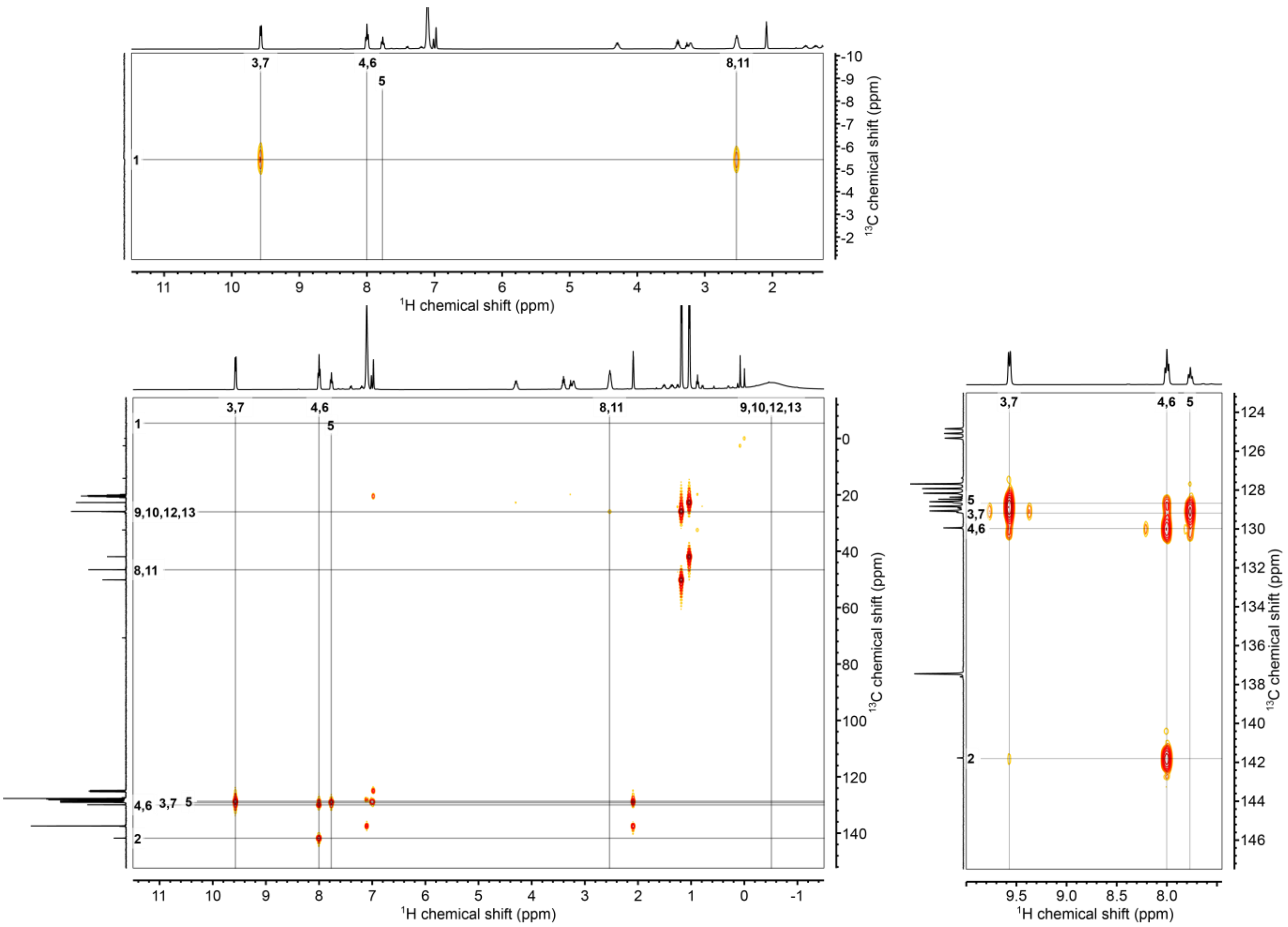

Figure S70. ^{1}H-^{13}C HMBC spectra of [Sm(*i*Pr$_2$BA)$_3$] (**3**) in toluene-d_8 at 303 K.

[Eu(*i*Pr$_2$BA)$_3$] (**4**)

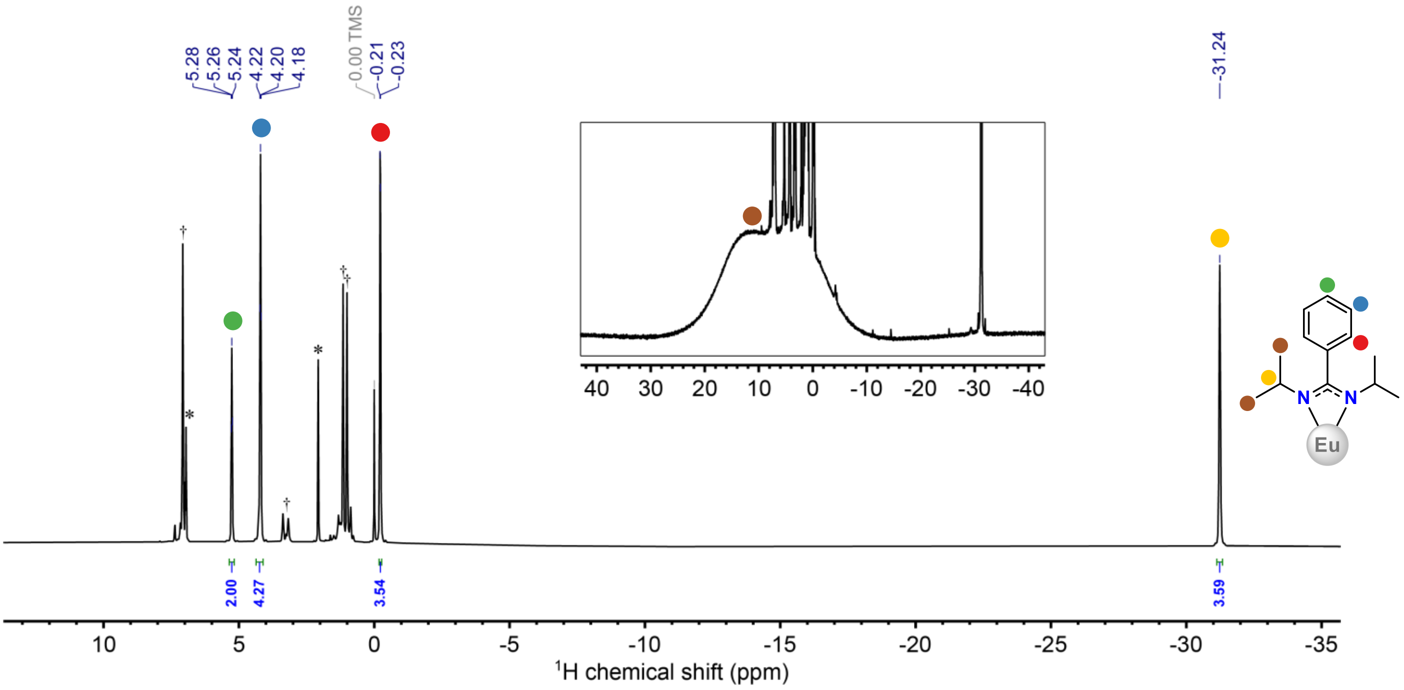

Figure S71. ^{1}H-NMR spectrum of [Eu(*i*Pr$_2$BA)$_3$] (**4**) in toluene-d_8 (indicated by asterisks) at 303 K. Dagger (†) indicates signals from inseparable free ligand. A scaled-up version of the broad isopropyl methyl peak is shown as an insert above the main spectrum for clarity.

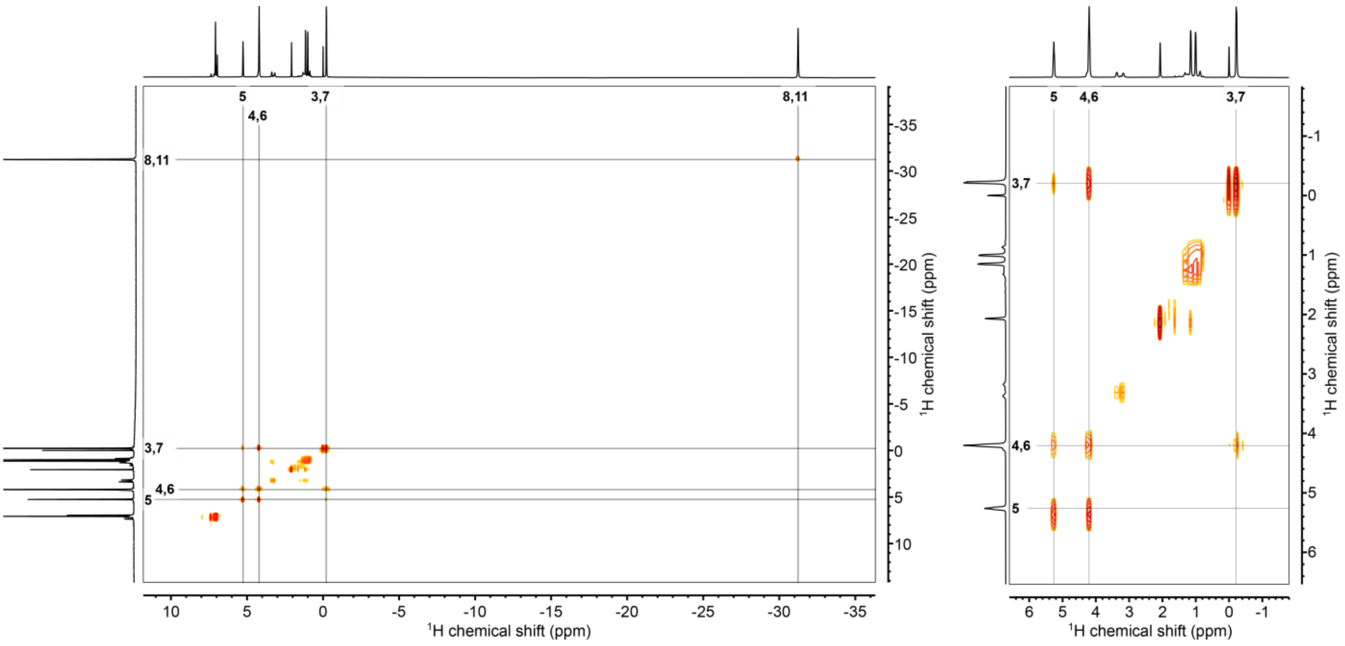

Figure S72. ^{1}H-^{1}H COSY spectra of [Eu(*i*Pr$_2$BA)$_3$] (**4**) in toluene-d_8 at 303 K.

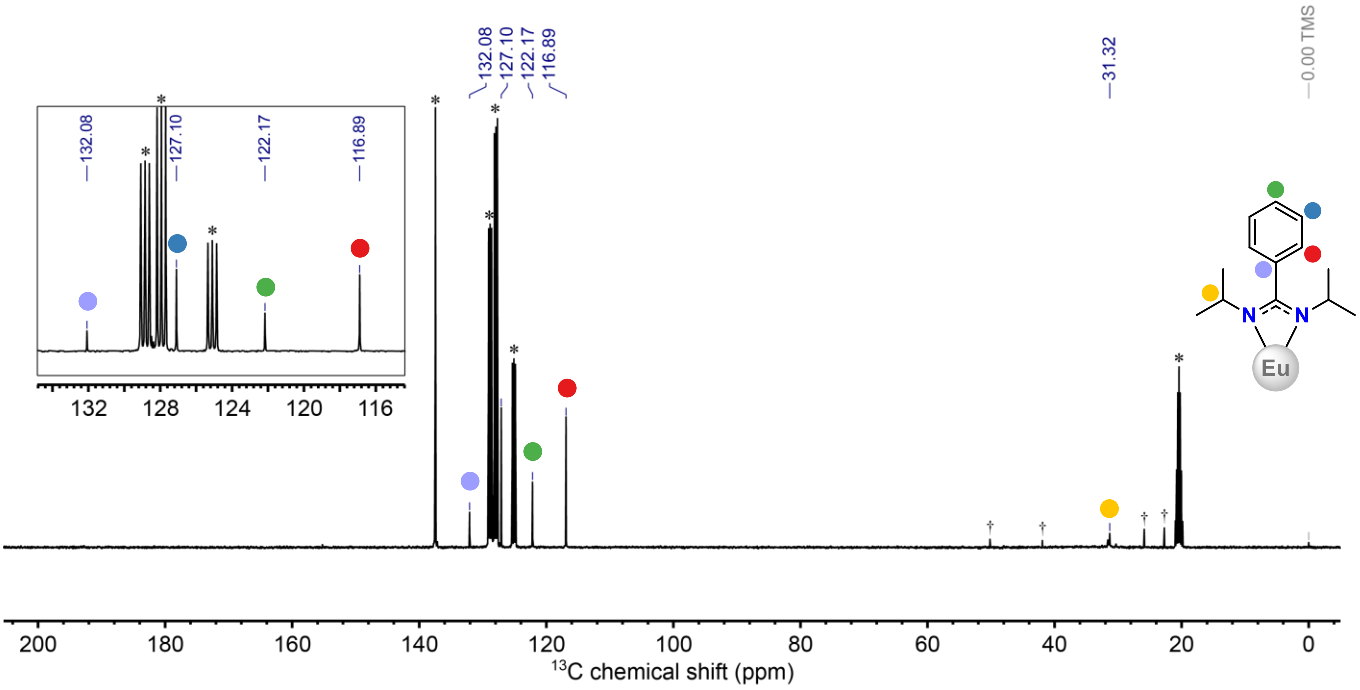

Figure S73. ^{13}C-NMR spectrum of [Eu(*i*Pr$_2$BA)$_3$] (**4**) in toluene-d_8 (indicated by asterisks) at 303 K. Dagger (†) indicates signals from inseparable free ligand. A scaled-up version of the compound peaks is shown as an insert above the main spectrum for clarity. Signals for the isopropyl methyl group carbons could not be observed at 303 K.

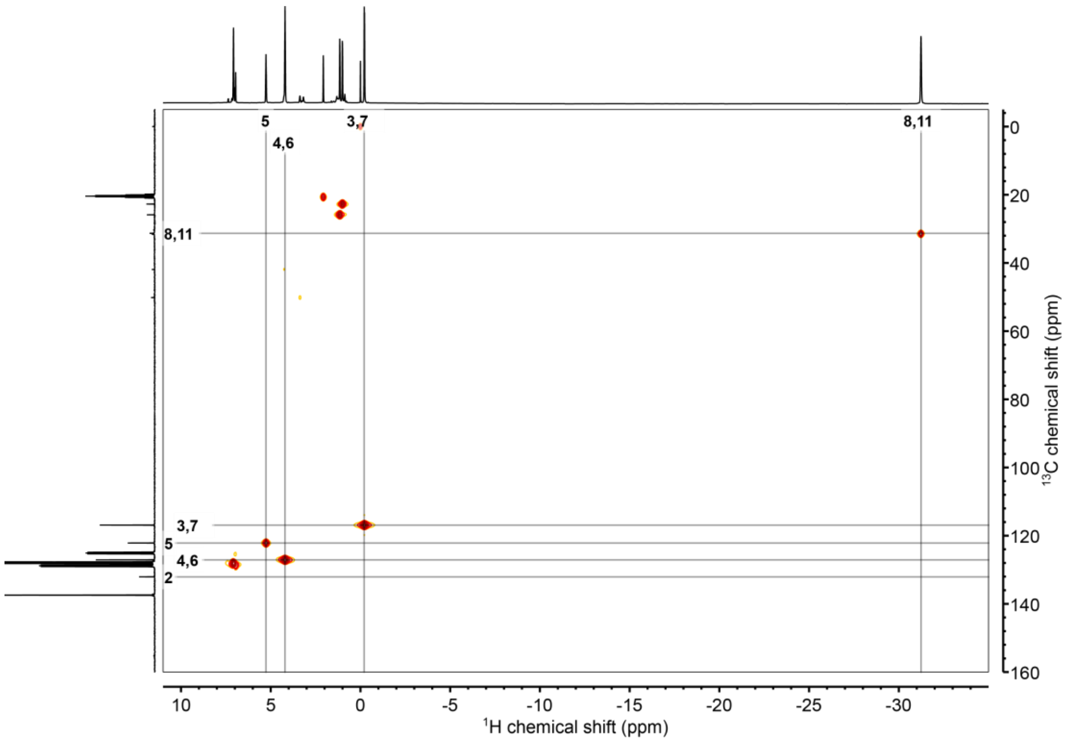

Figure S74. ^{1}H-^{13}C HSQC spectrum of [Eu(*i*Pr$_2$BA)$_3$] (**4**) in toluene-d_8 at 303 K.

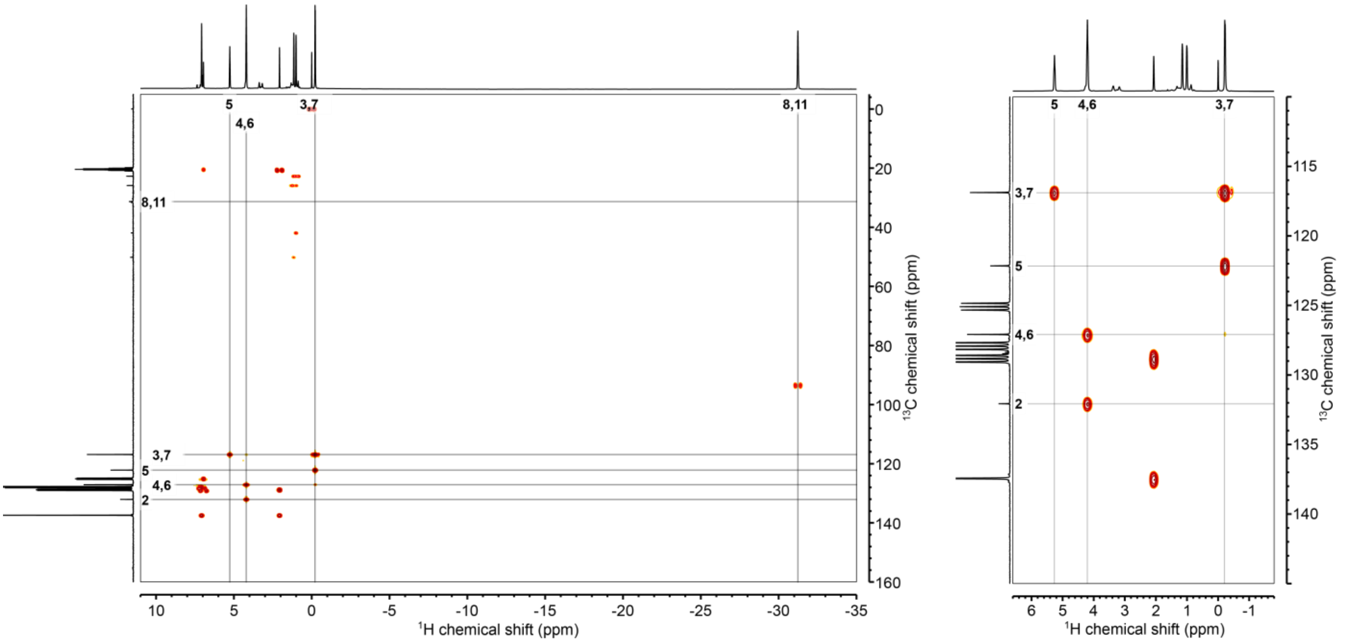

Figure S75. ^{1}H-^{13}C HMBC spectra of [Eu(*i*Pr$_2$BA)$_3$] (**4**) in toluene-*d*$_8$ at 303 K.

[Yb(*i*Pr$_2$BA)$_3$] (**5**)

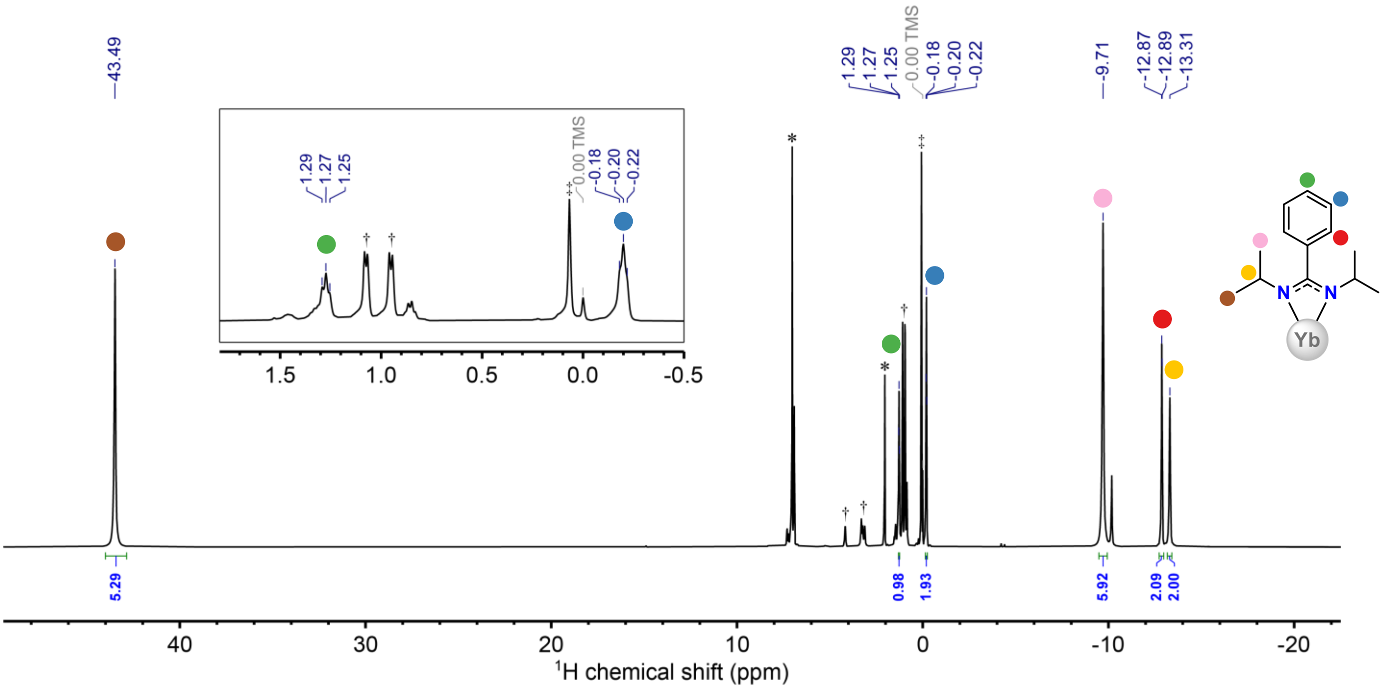

Figure S76. ^{1}H-NMR spectrum of [Yb(*i*Pr$_2$BA)$_3$] (**5**) in toluene-d_8 (indicated by asterisks) at 303 K. Dagger (†) indicates signals from inseparable free ligand. A scaled-up version of the compound peaks is shown as an insert above the main spectrum for clarity.

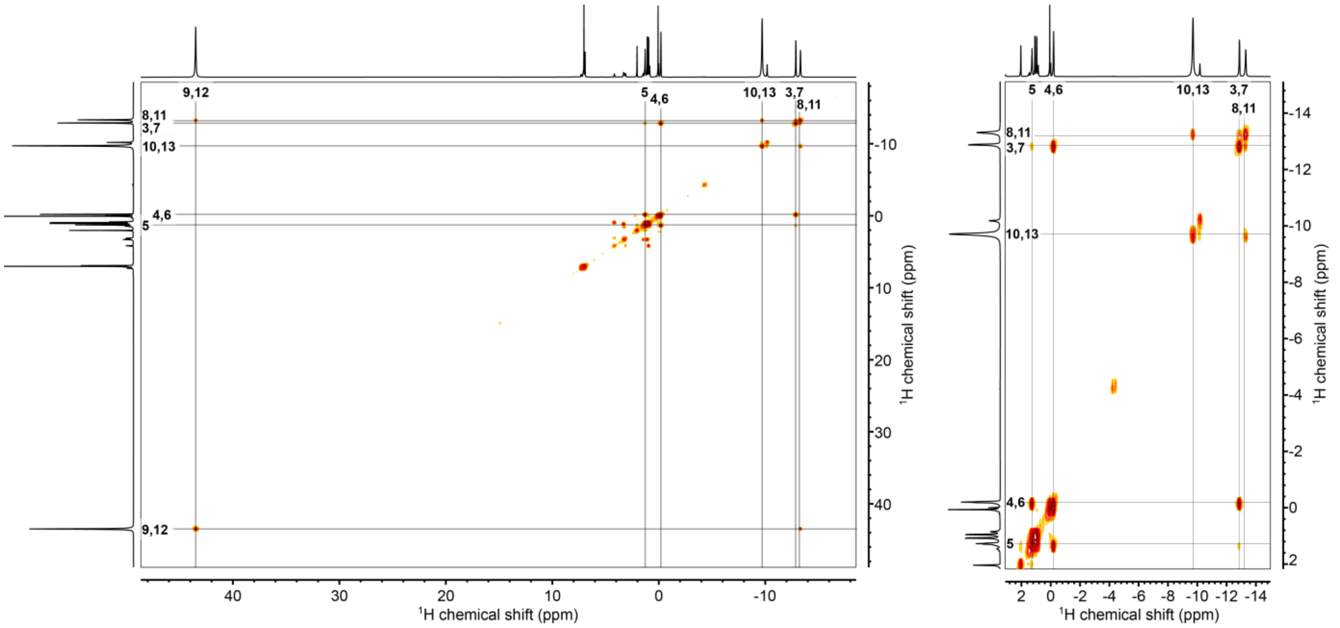

Figure S77. ^{1}H-^{1}H COSY spectra of [Yb(*i*Pr$_2$BA)$_3$] (**5**) in toluene-d_8 at 303 K.

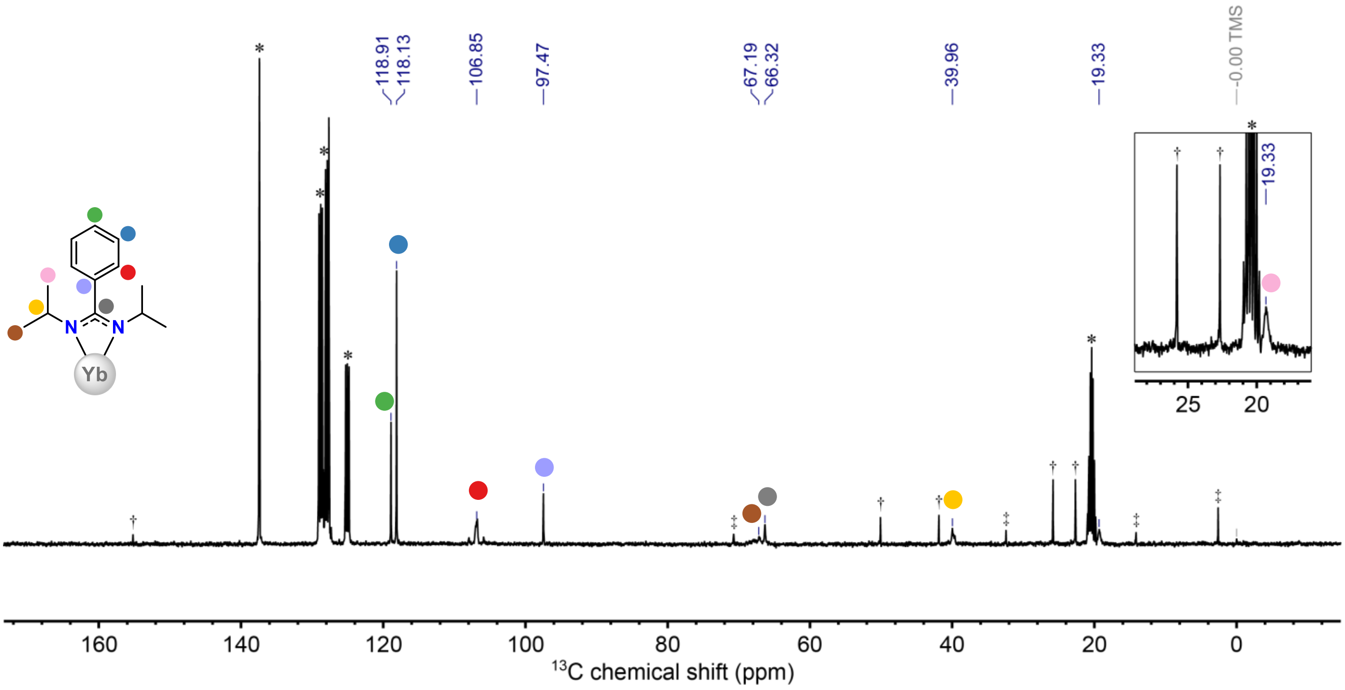

Figure S78. ^{13}C-NMR spectrum of [Yb(*i*Pr$_2$BA)$_3$] (**5**) in toluene-d_8 (indicated by asterisks) at 303 K. Dagger (†) indicates signals from inseparable free ligand. Double dagger (‡) indicates signals from HMDS impurities from base. A scaled-up version of the compound peaks is shown as an insert above the main spectrum for clarity.

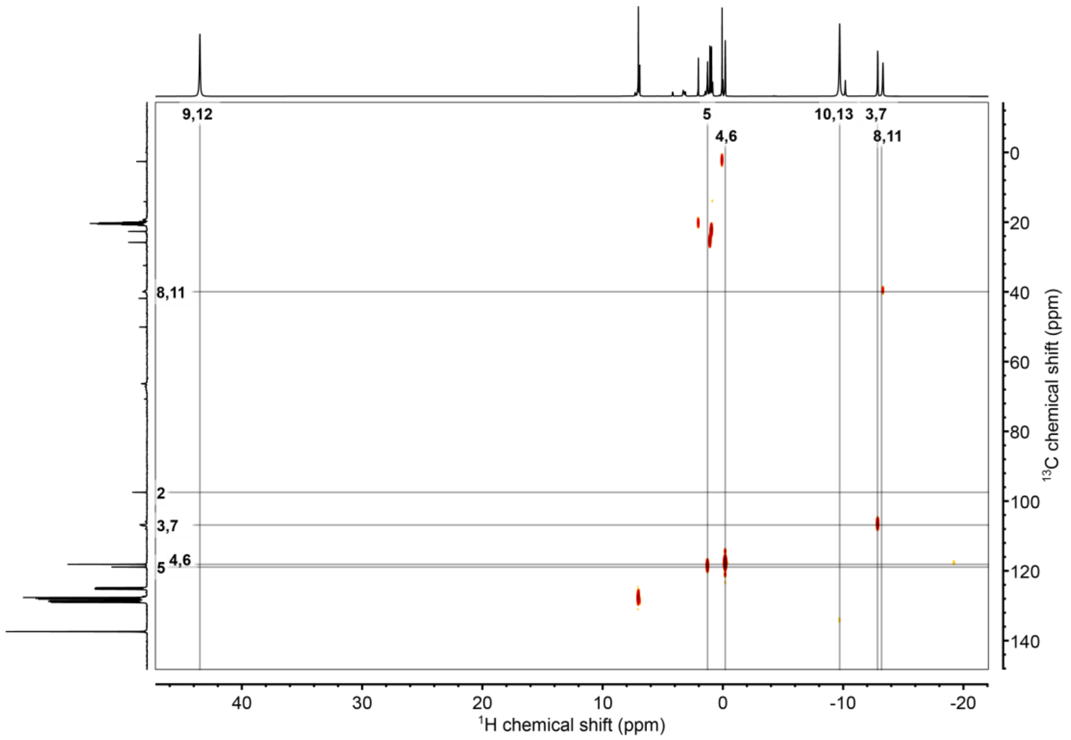

Figure S79. ^{1}H-^{13}C HSQC spectrum of [Yb(*i*Pr$_2$BA)$_3$] (**5**) in toluene-d_8 at 303 K.

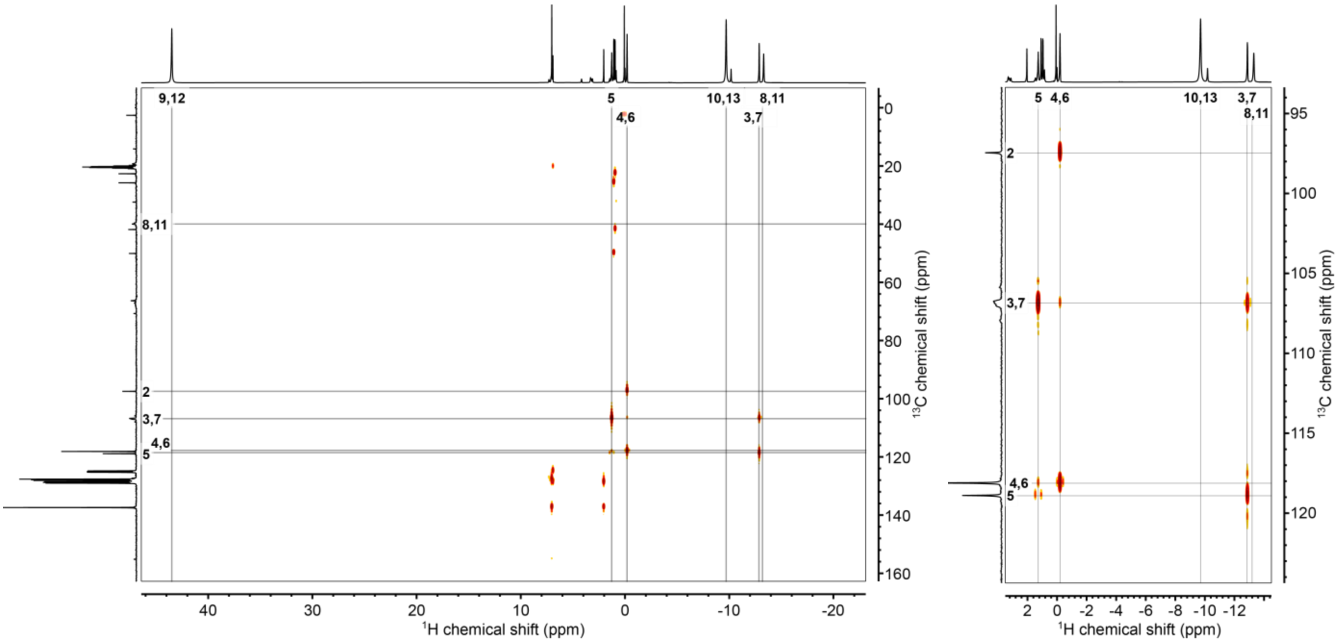

Figure S80. ^{1}H-^{13}C HMBC spectra of [Yb(iPr$_2$BA)$_3$] (**5**) in toluene-d_8 at 303 K.

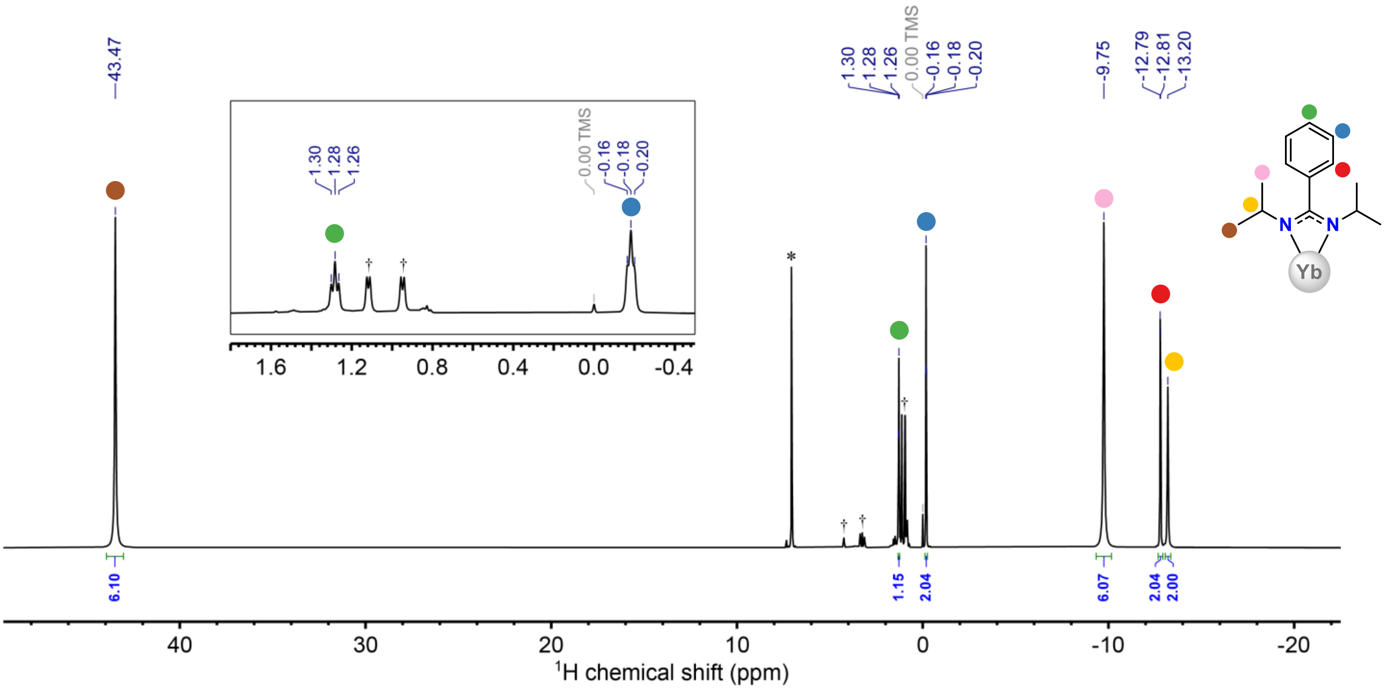

Figure S81. ^{1}H-NMR spectrum of [Yb(*i*Pr$_2$BA)$_3$] (**5**) in C_6D_6 (indicated by asterisks) at 303 K. Dagger (†) indicates signals from inseparable free ligand. A scaled-up version of the compound peaks is shown as an insert above the main spectrum for clarity.

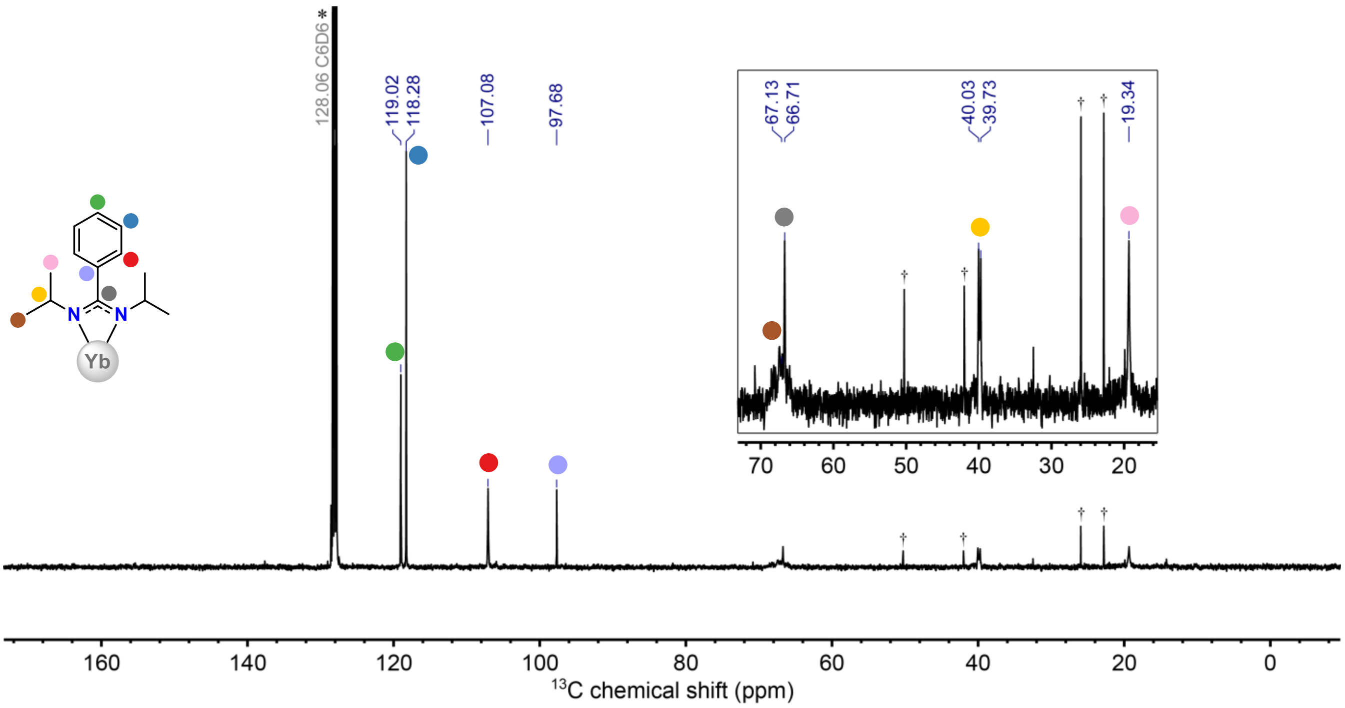

Figure S82. ^{13}C-NMR spectrum of [Yb(*i*Pr$_2$BA)$_3$] (**5**) in C_6D_6 (indicated by asterisks) at 303 K. Dagger (†) indicates signals from inseparable free ligand. A scaled-up version of the compound peaks is shown as an insert above the main spectrum for clarity.

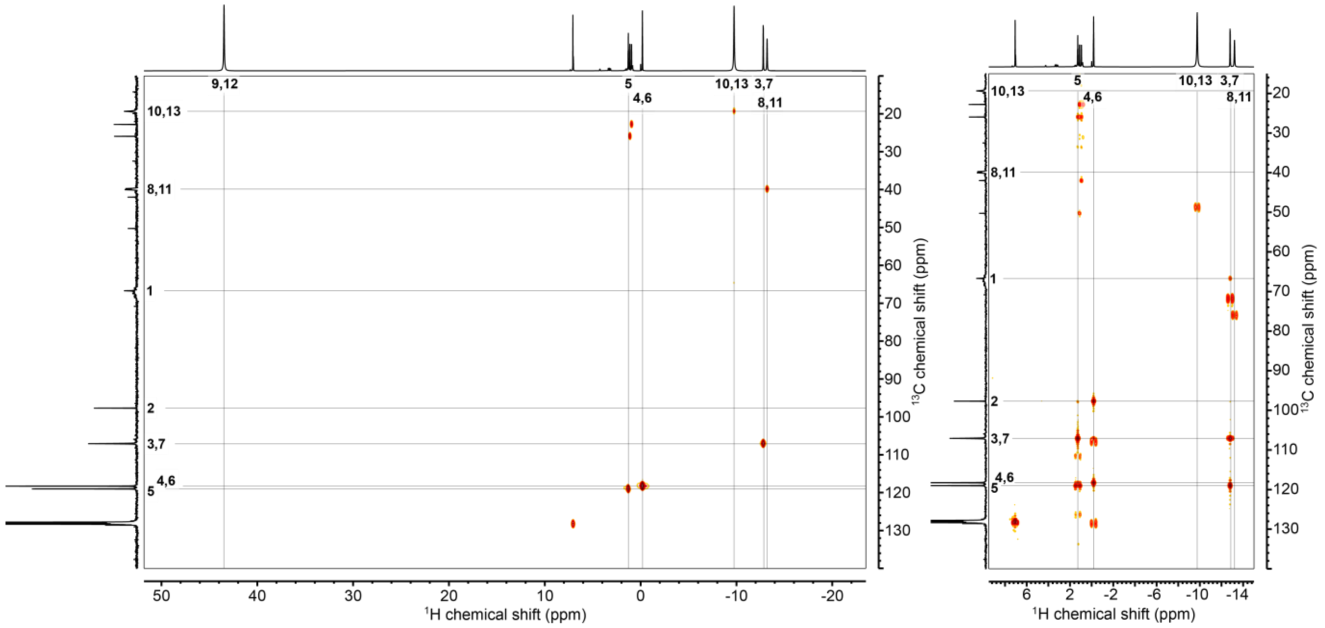

Figure S83. ^{1}H-^{13}C HSQC (left) and ^{1}H-^{13}C HMBC (right) spectra of [Yb(*i*Pr$_2$BA)$_3$] (**5**) in C_6D_6 at 303 K.

[Lu(*i*Pr$_2$BA)$_3$] (**6**)

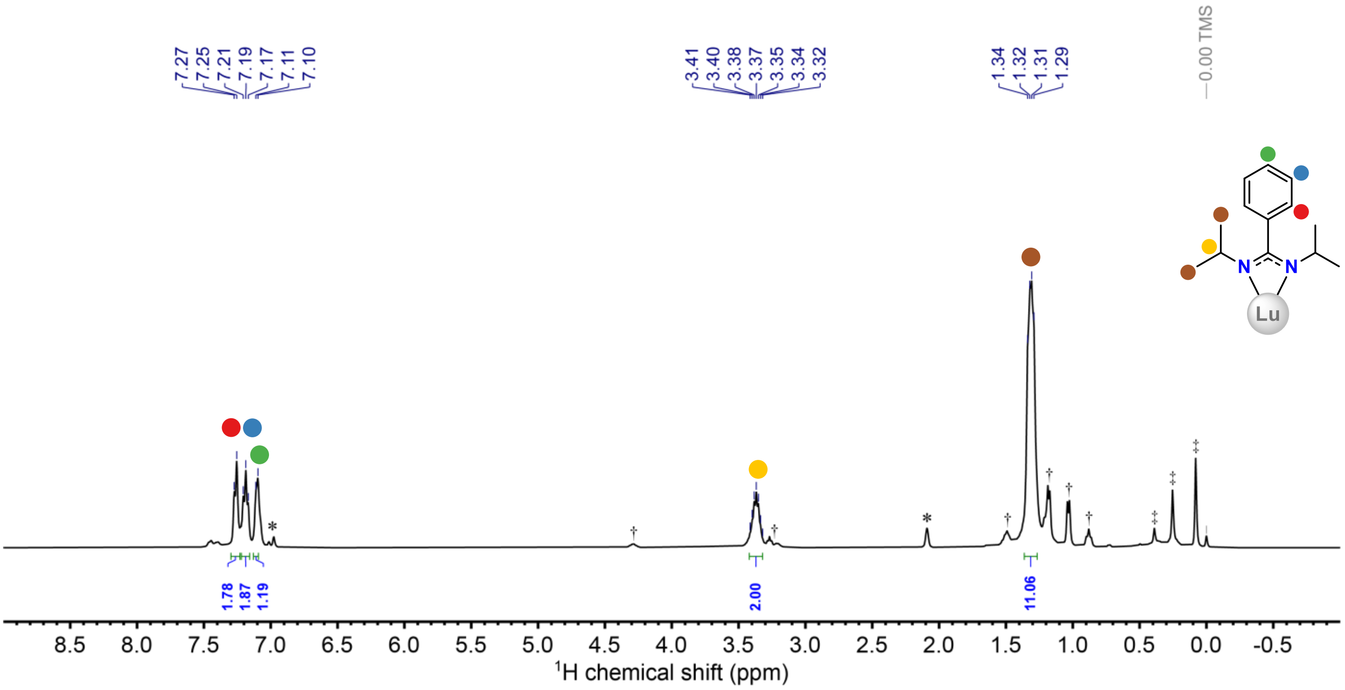

Figure S84. ^{1}H-NMR spectrum of [Lu(*i*Pr$_2$BA)$_3$] (**6**) in toluene-d_8 (indicated by asterisks) at 303 K. Dagger (†) indicates signals from inseparable free ligand. Double dagger (‡) indicates signals from HMDS impurities from base.

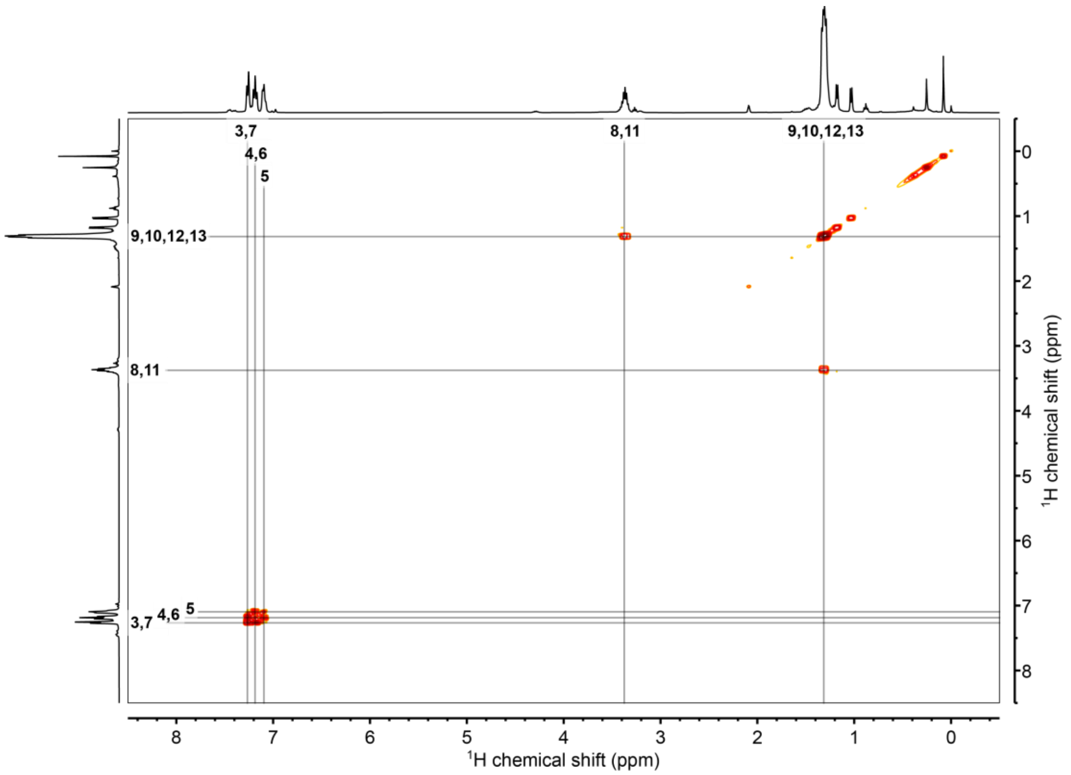

Figure S85. ^{1}H-^{1}H COSY spectrum of [Lu(*i*Pr$_2$BA)$_3$] (**6**) in toluene-d_8 at 303 K.

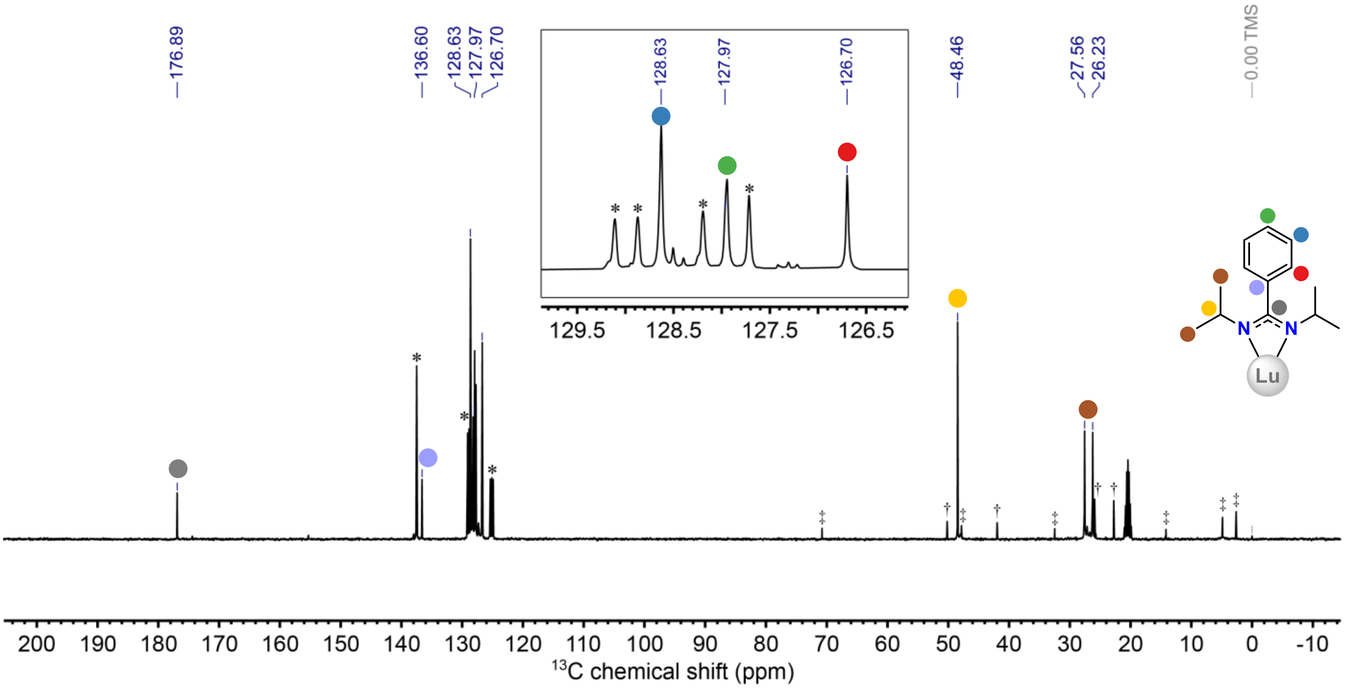

Figure S86. ^{13}C-NMR spectrum of [Lu(iPr$_2$BA)$_3$] (**6**) in toluene-d_8 (indicated by asterisks) at 303 K. Dagger (†) indicates signals from inseparable free ligand. Double dagger (‡) indicates signals from HMDS impurities from base. A scaled-up version of the compound peaks is shown as an insert above the main spectrum for clarity.

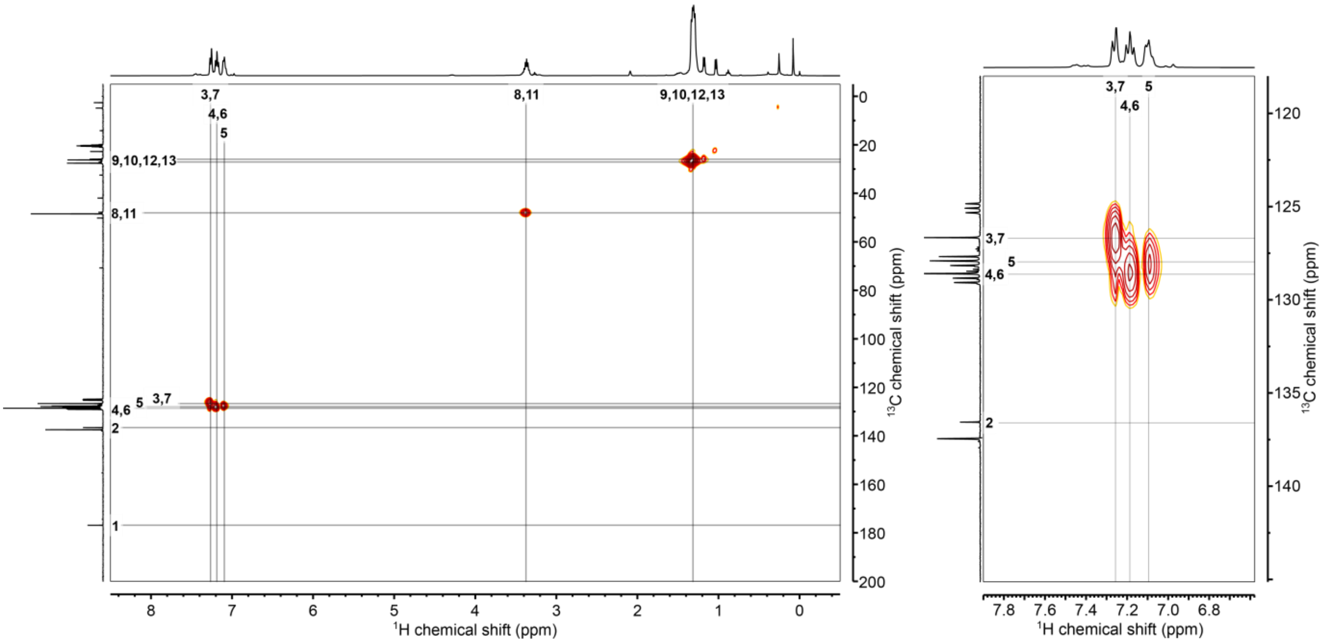

Figure S87. ^{1}H-^{13}C HSQC spectra of [Lu(iPr$_2$BA)$_3$] (**6**) in toluene-d_8 at 303 K.

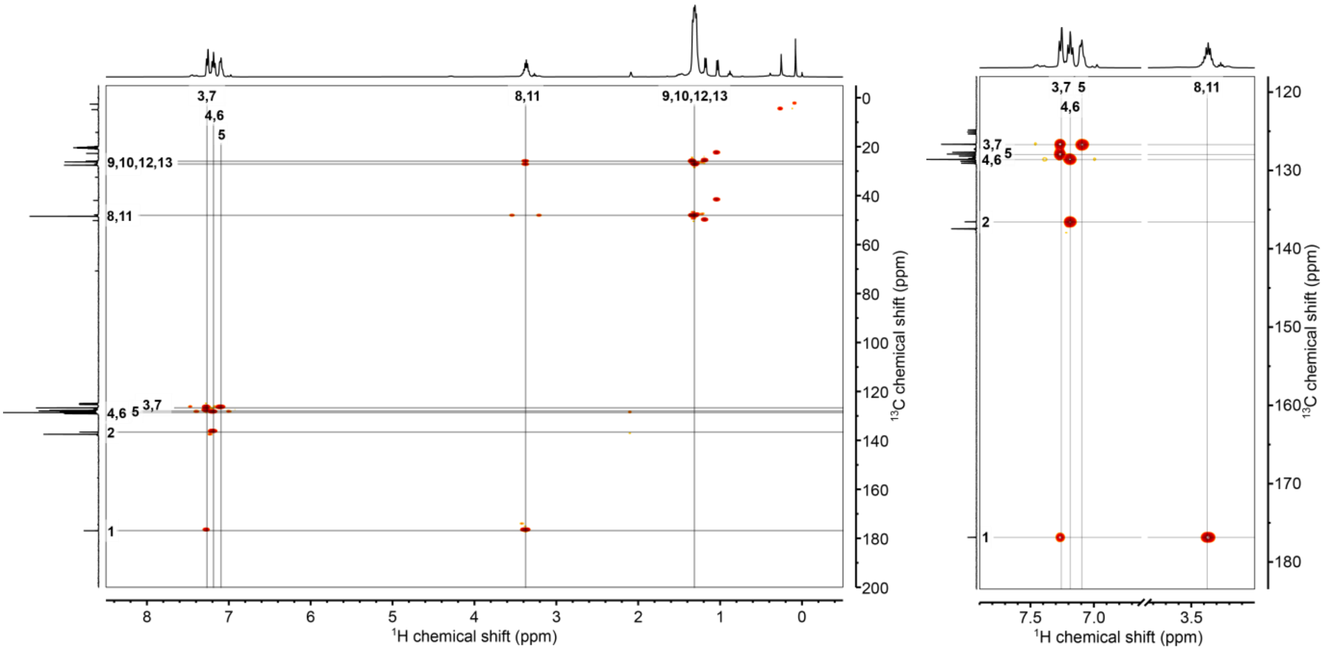

Figure S88. ^{1}H-^{13}C HMBC spectra of [Lu(*i*Pr$_2$BA)$_3$] (**6**) in toluene-d_8 at 303 K.

[U(*i*Pr$_2$BA)$_3$] (**7**)

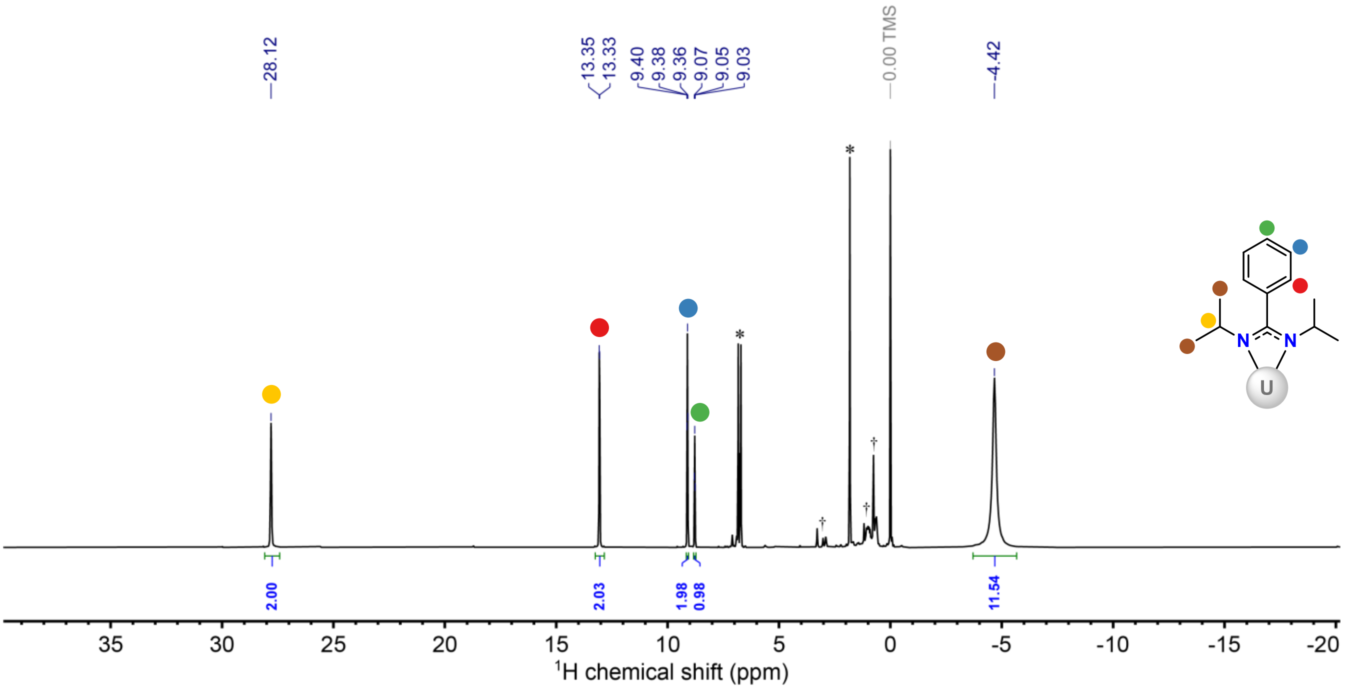

Figure S89. ^{1}H-NMR spectrum of [U(*i*Pr$_2$BA)$_3$] (**7**) in toluene-d_8 (indicated by asterisks) at 303 K. Dagger (†) indicates signals from inseparable free ligand.

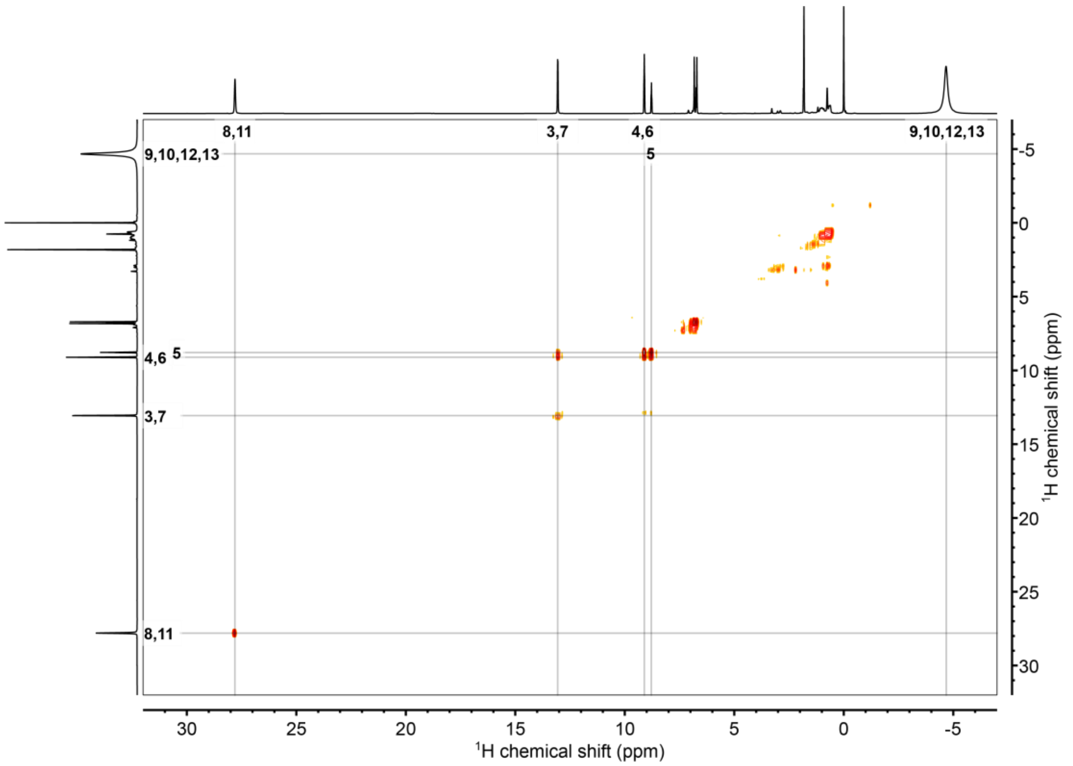

Figure S90. ^{1}H-^{1}H COSY spectrum of [U(*i*Pr$_2$BA)$_3$] (**7**) in toluene-d_8 at 303 K.

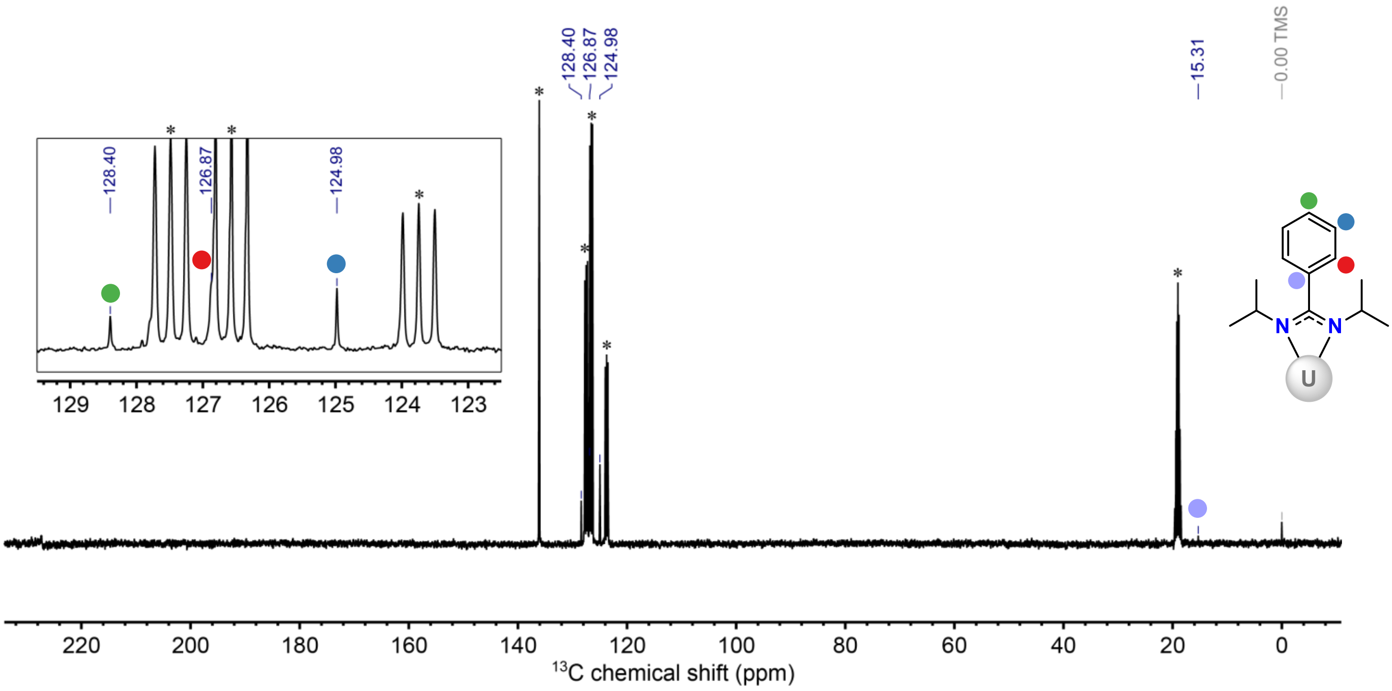

Figure S91. ^{13}C-NMR spectrum of [U(*i*Pr$_2$BA)$_3$] (**7**) in toluene-*d*$_8$ (indicated by asterisks) at 303 K. A scaled-up version of the compound peaks is shown as an insert above the main spectrum for clarity. Signals for the N***C***N, (***C***H$_3$)$_2$CHN, (CH$_3$)$_2$***C***HN carbons could not be observed.

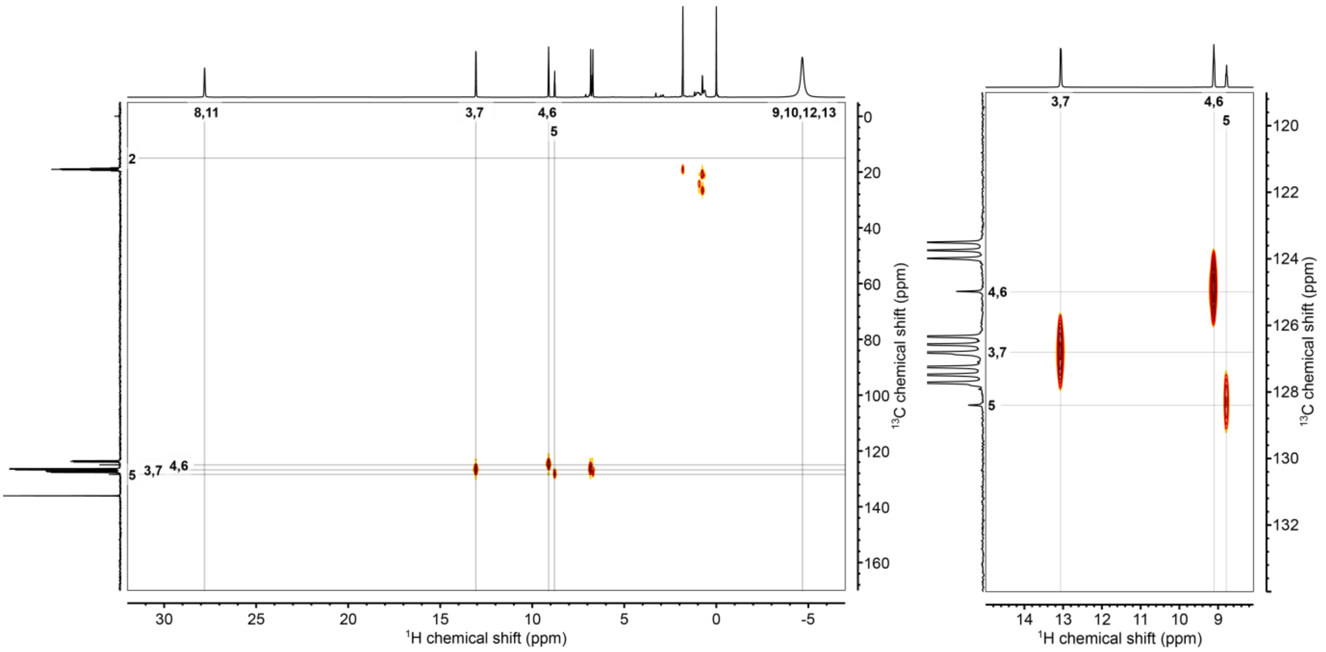

Figure S92. ^{1}H-^{13}C HSQC spectra of [U(*i*Pr$_2$BA)$_3$] (**7**) in toluene-*d*$_8$ at 303 K.

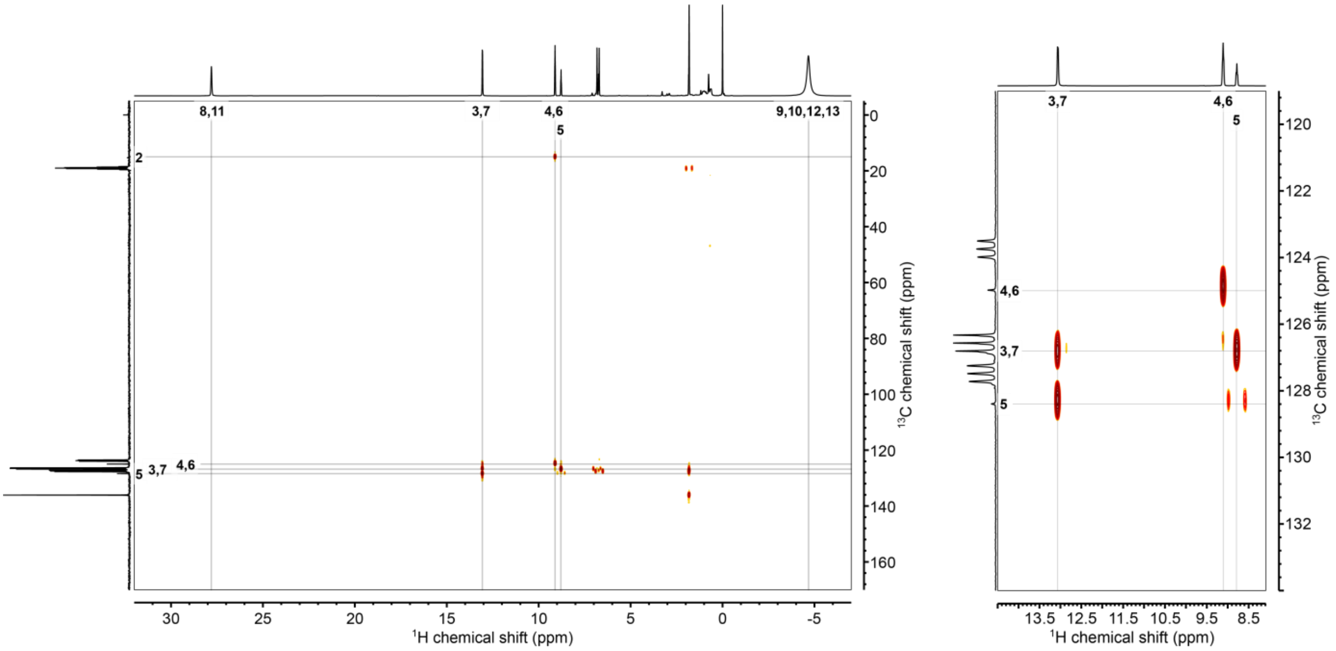

Figure S93. ^{1}H-^{13}C HMBC spectra of [U(*i*Pr_2BA)$_3$] (**7**) in toluene-d_8 at 303 K.

[Np(*i*Pr$_2$BA)$_3$] (**8**)

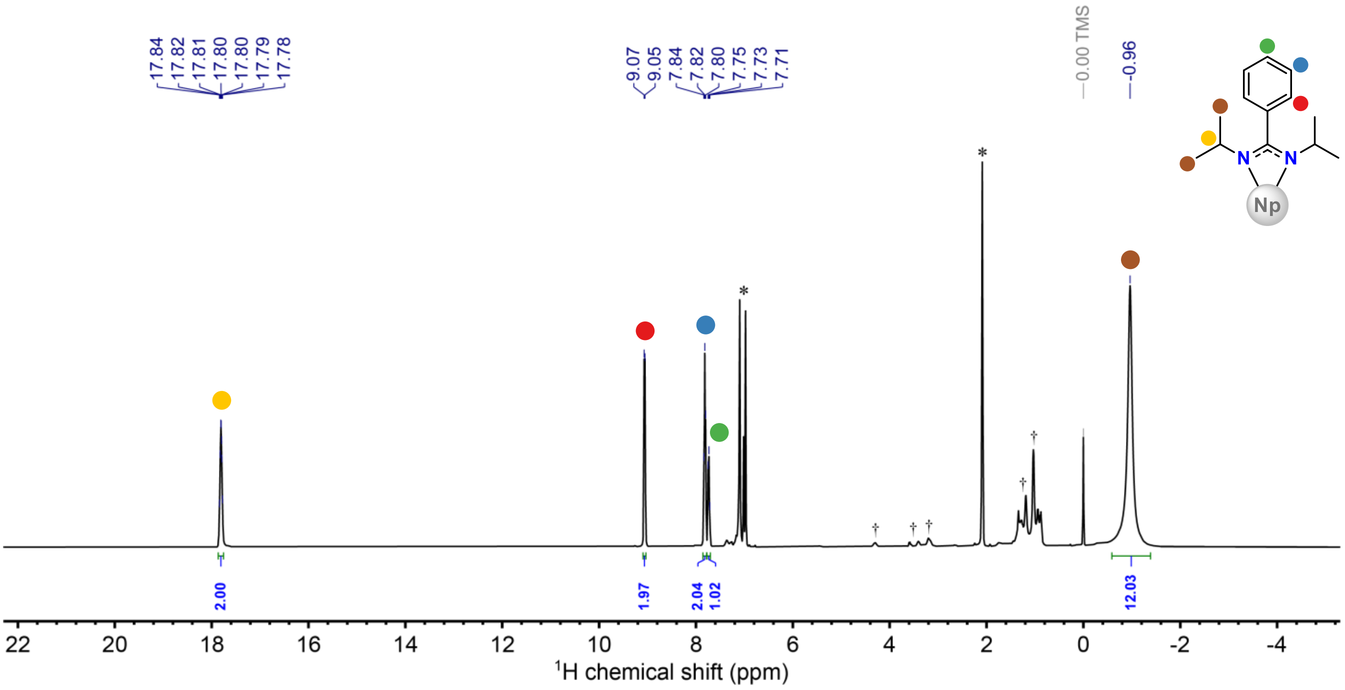

Figure S94. ^{1}H-NMR spectrum of [Np(*i*Pr$_2$BA)$_3$] (**8**) in toluene-d_8 (indicated by asterisks) at 303 K. Dagger (†) indicates signals from inseparable free ligand.

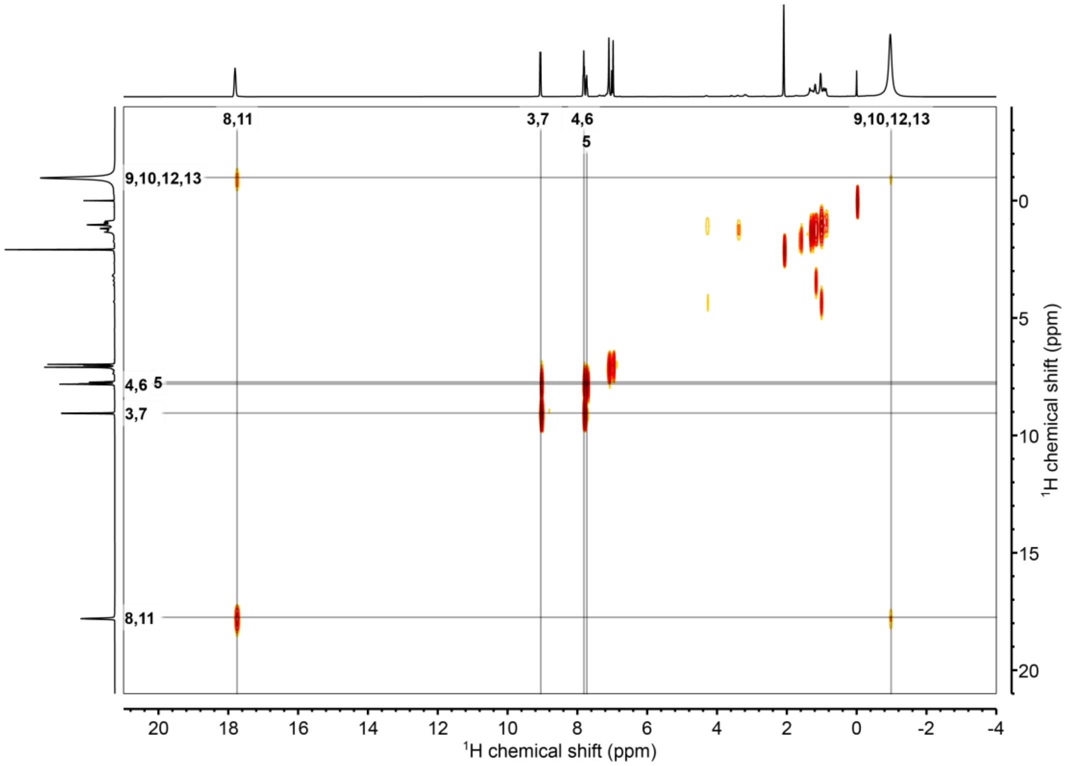

Figure S95. ^{1}H-^{1}H COSY spectrum of [Np(*i*Pr$_2$BA)$_3$] (**8**) in toluene-d_8 at 303 K.

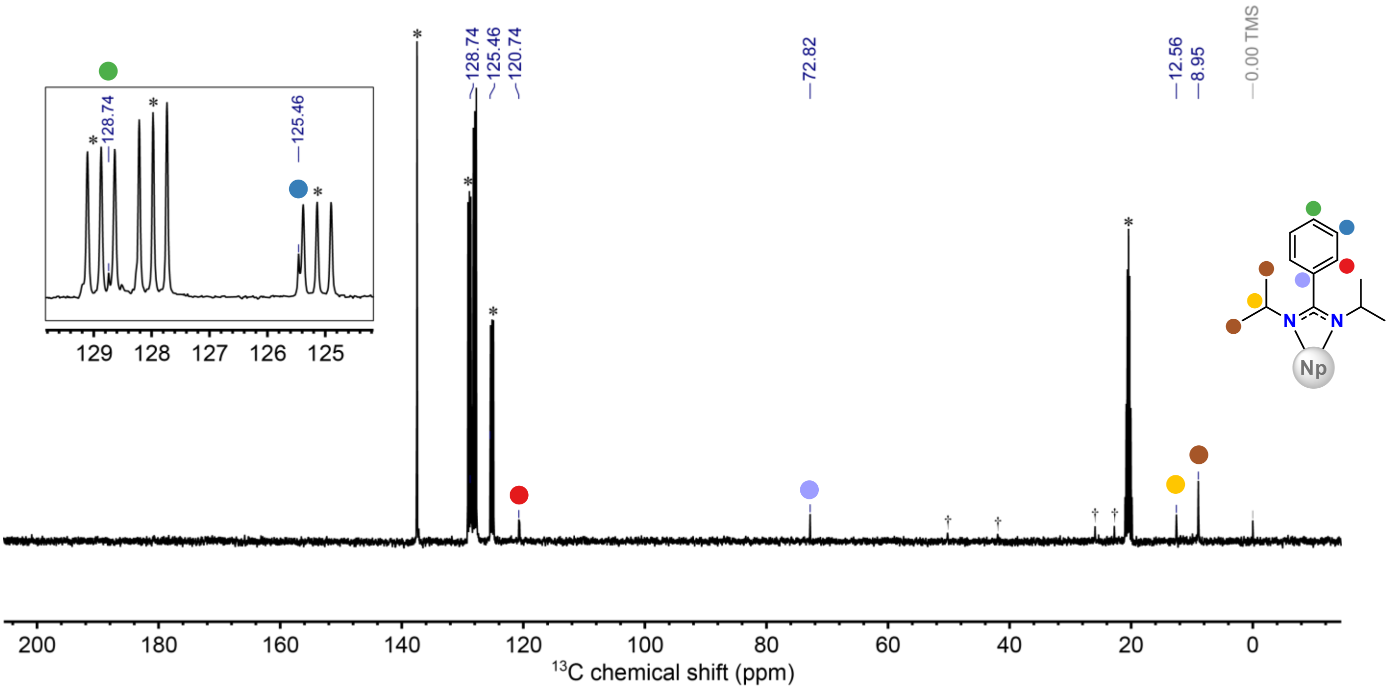

Figure S96. ^{13}C-NMR spectrum of [Np(*i*Pr$_2$BA)$_3$] (**8**) in toluene-*d*$_8$ (indicated by asterisks) at 303 K. Dagger (†) indicates signals from inseparable free ligand. A scaled-up version of the compound peaks is shown as an insert above the main spectrum for clarity. Signal for the N*C*N carbon could not be observed.

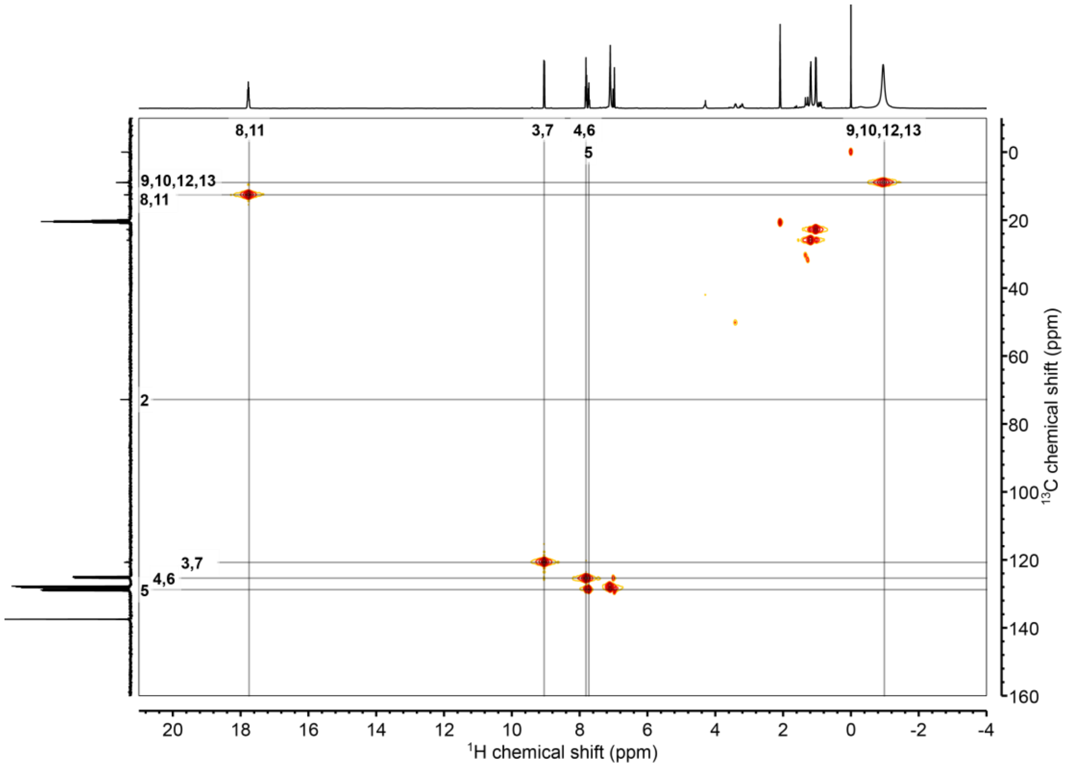

Figure S97. ^{1}H-^{13}C HSQC spectrum of [Np(*i*Pr$_2$BA)$_3$] (**8**) in toluene-*d*$_8$ at 303 K.

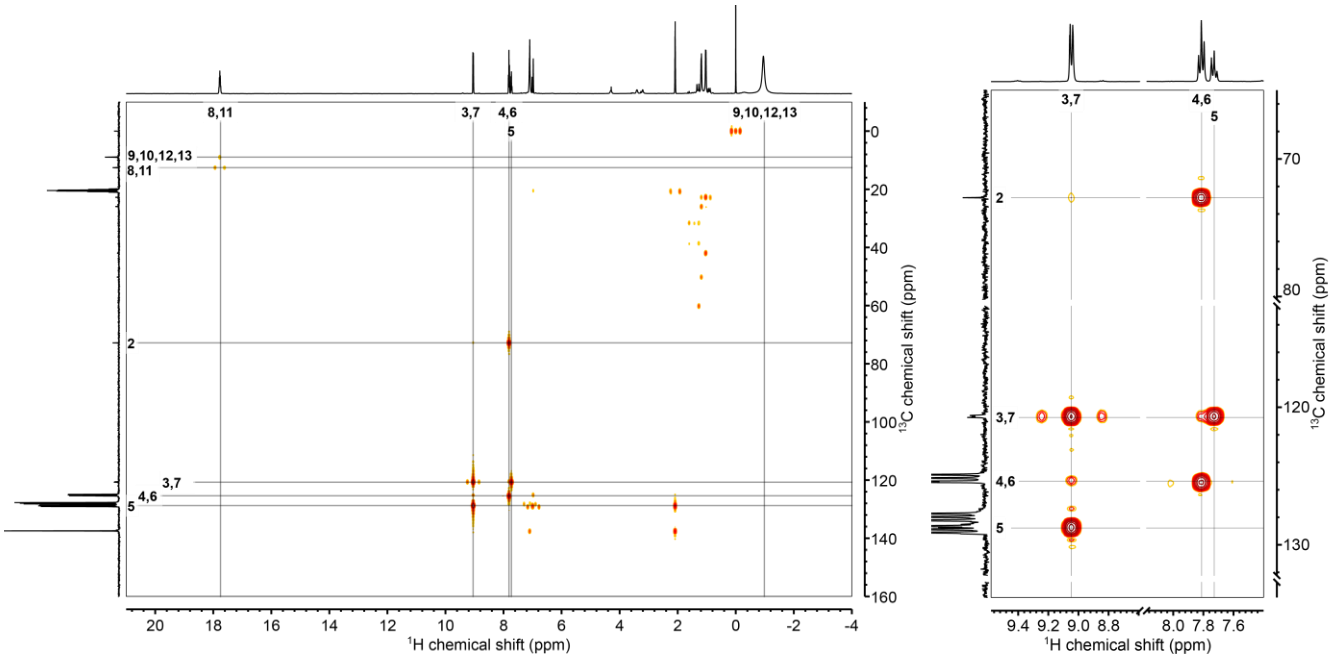

Figure S98. ^{1}H-^{13}C HMBC spectra of [Np(*i*Pr$_2$BA)$_3$] (**8**) in toluene-d_8 at 303 K.

[UCl(*i*Pr$_2$BA)$_3$] (**7-Cl**)

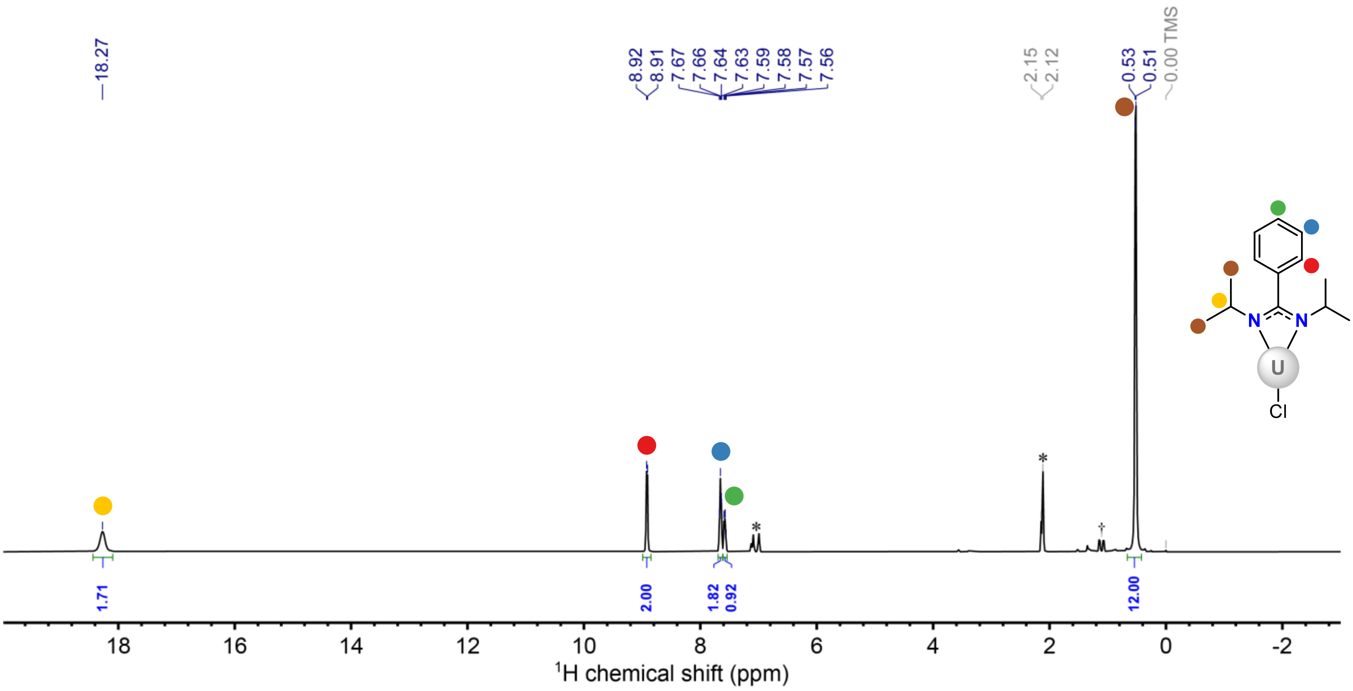

Figure S99. ^{1}H-NMR spectrum of [UCl(*i*Pr$_2$BA)$_3$] (**7-Cl**) in toluene-d_8 (indicated by asterisks) at 368 K. Dagger (†) indicates signals from inseparable free ligand.

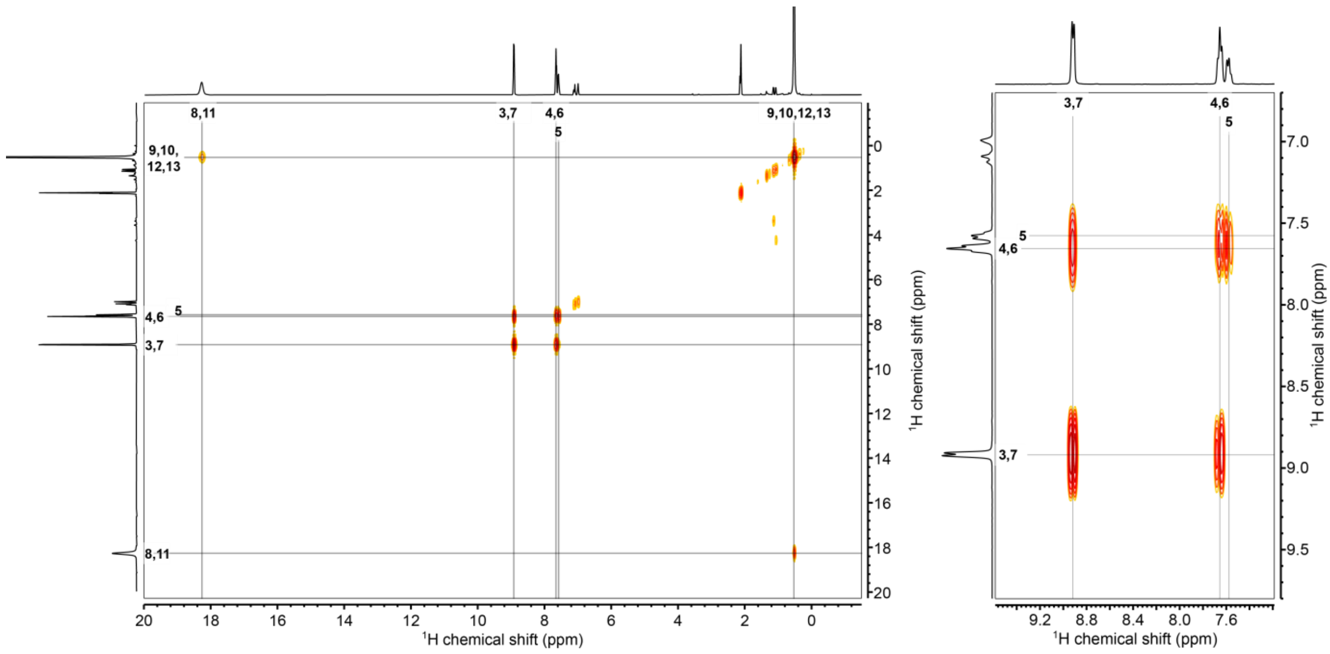

Figure S100. ^{1}H-^{1}H COSY spectra of [UCl(*i*Pr$_2$BA)$_3$] (**7-Cl**) in toluene-d_8 at 368 K.

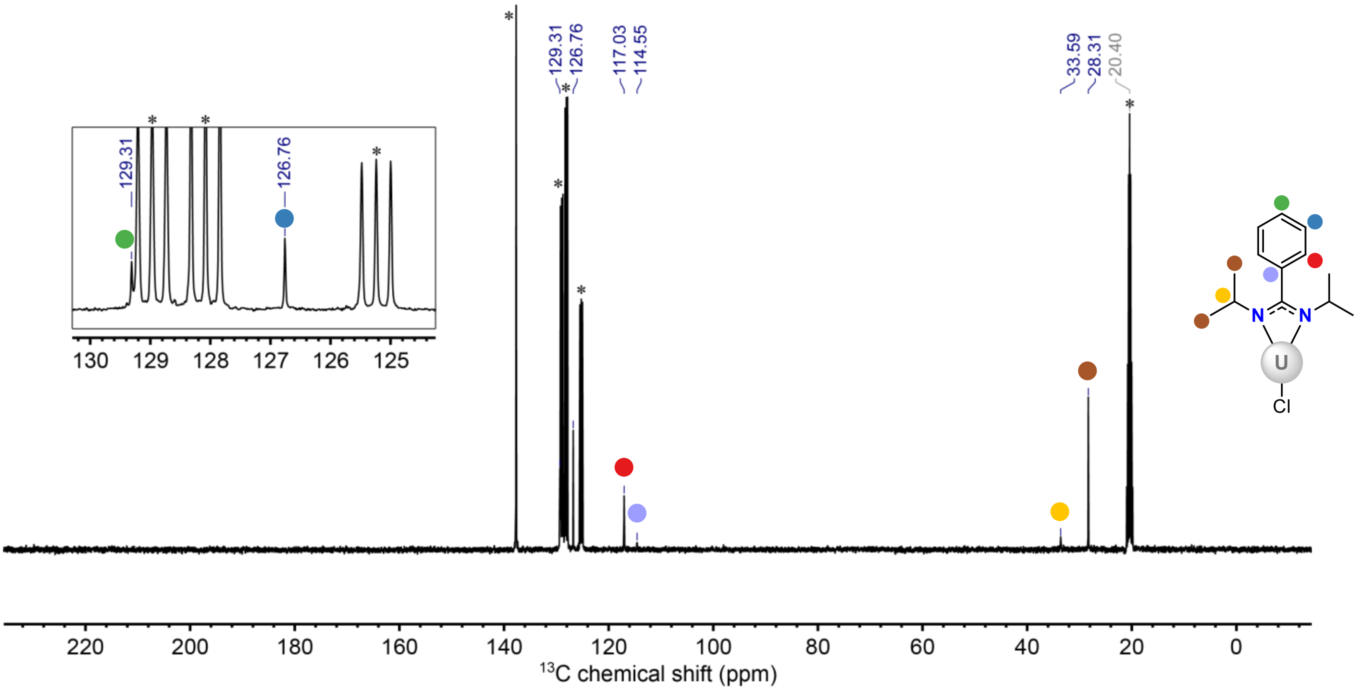

Figure S101. ^{13}C-NMR spectrum of [UCl(iPr$_2$BA)$_3$] (**7-Cl**) in toluene-d_8 (indicated by asterisks) at 368 K. A scaled-up version of the compound peaks is shown as an insert above the main spectrum for clarity. Signal for the N***C***N carbon could not be observed.

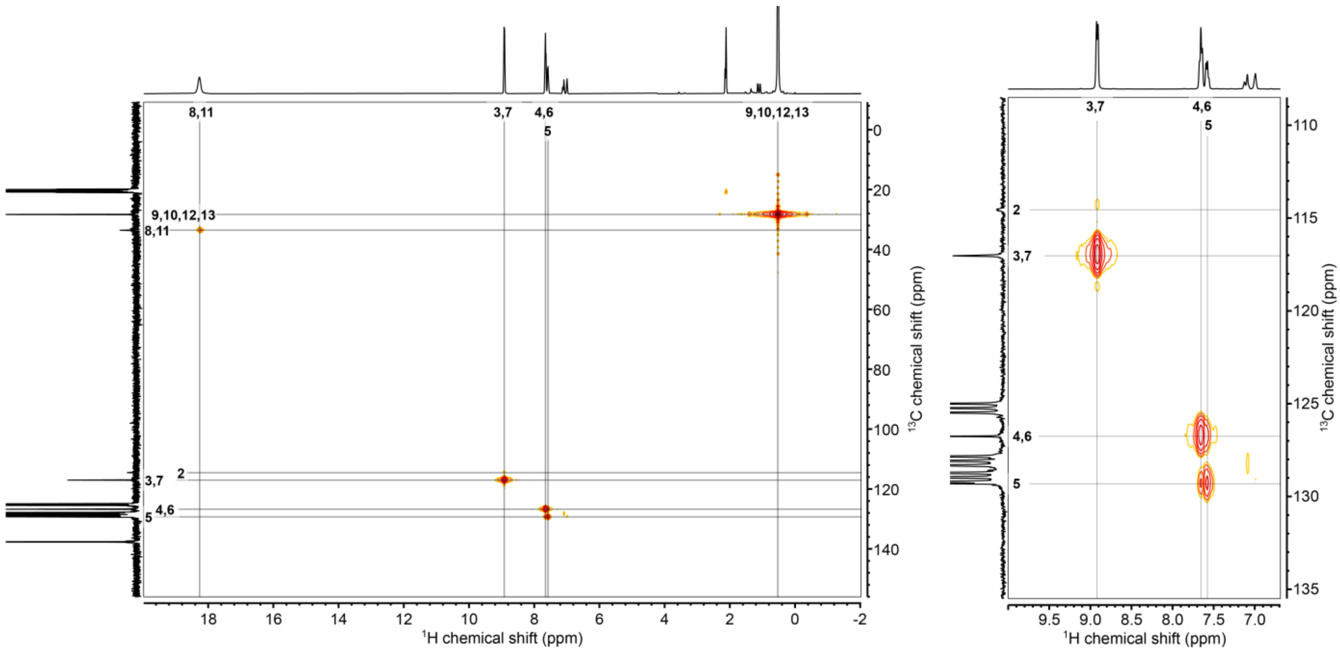

Figure S102. ^{1}H-^{13}C HSQC spectra of [UCl(iPr$_2$BA)$_3$] (**7-Cl**) in toluene-d_8 at 368 K.

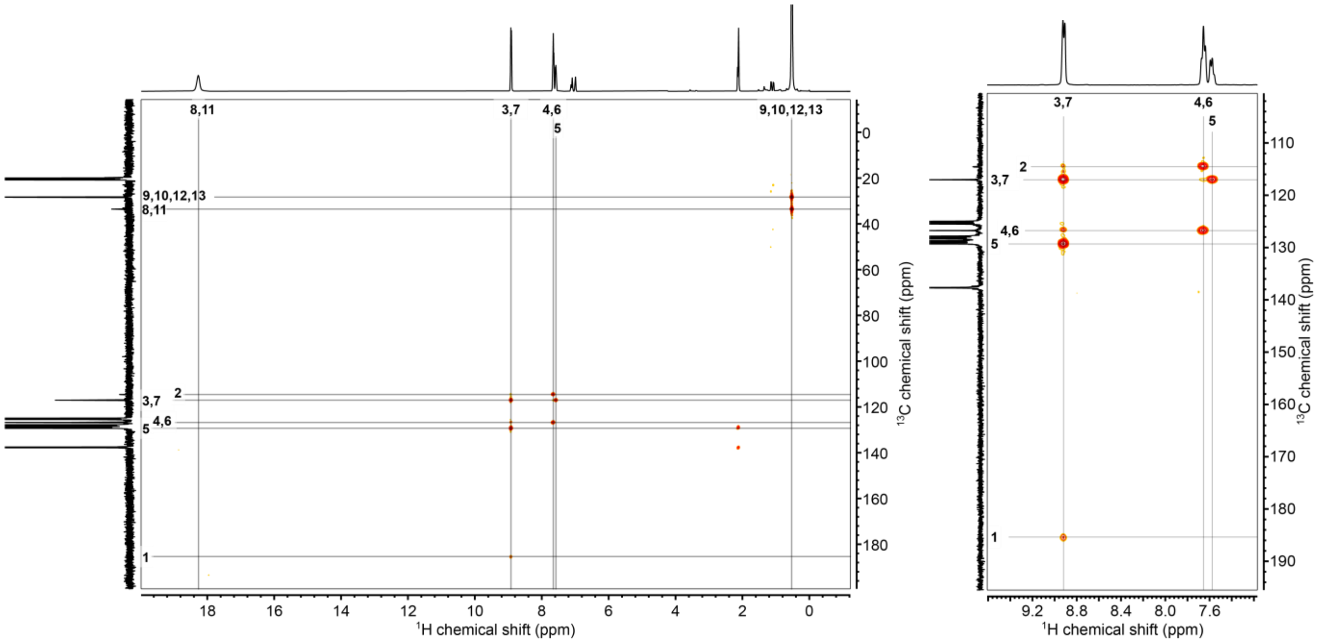

Figure S103. ^{1}H-^{13}C HMBC spectra of [UCl(*i*Pr$_2$BA)$_3$] (**7-Cl**) in toluene-d_8 at 368 K.

[NpCl(*i*Pr$_2$BA)$_3$] (**8-Cl**)

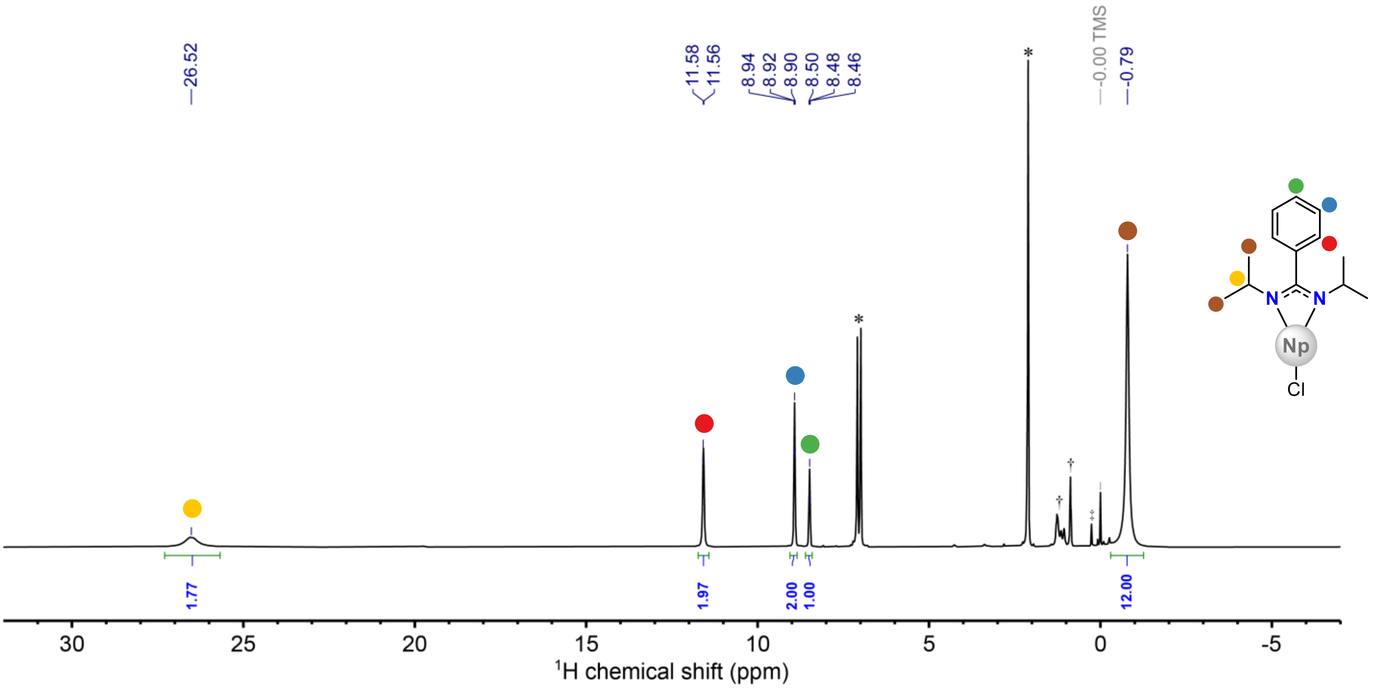

Figure S104. ^{1}H-NMR spectrum of [NpCl(*i*Pr$_2$BA)$_3$] (**8-Cl**) in toluene-d_8 (indicated by asterisks) at 363 K. Dagger (†) indicates signals from inseparable free ligand. Double dagger (‡) indicates signals from HMDS impurities from base.

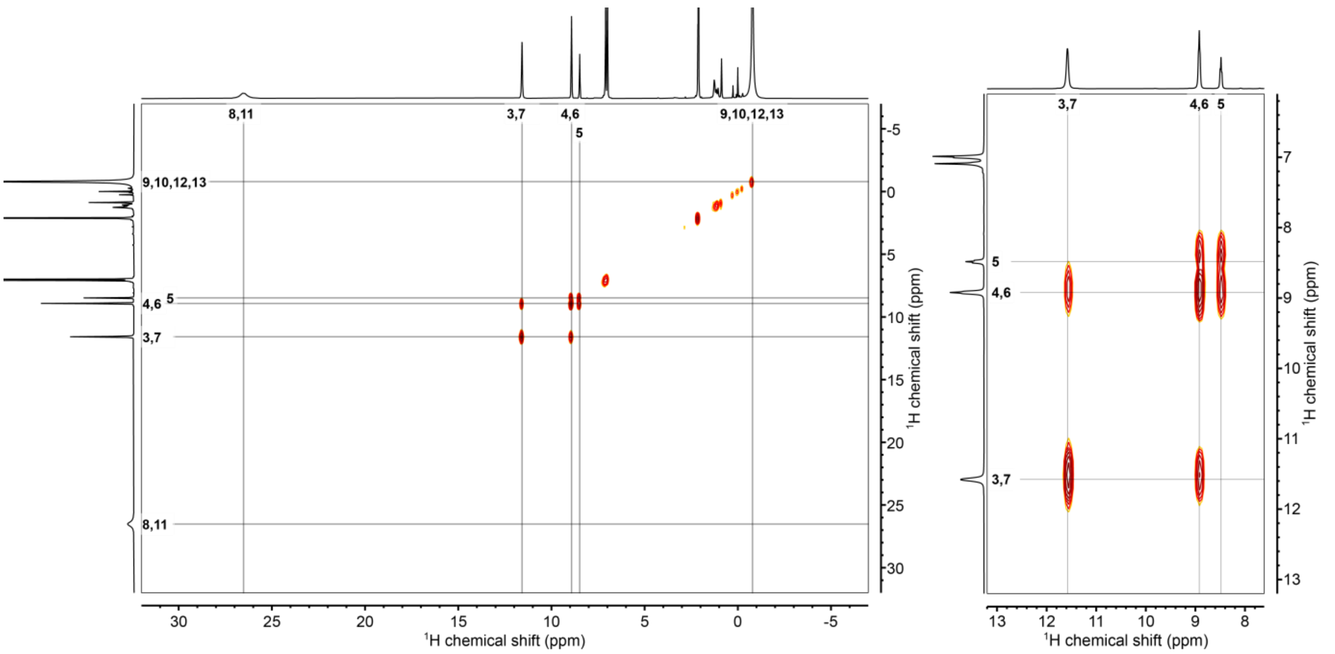

Figure S105. ^{1}H-^{1}H COSY spectra of [NpCl(*i*Pr$_2$BA)$_3$] (**8-Cl**) in toluene-d_8 at 363 K.

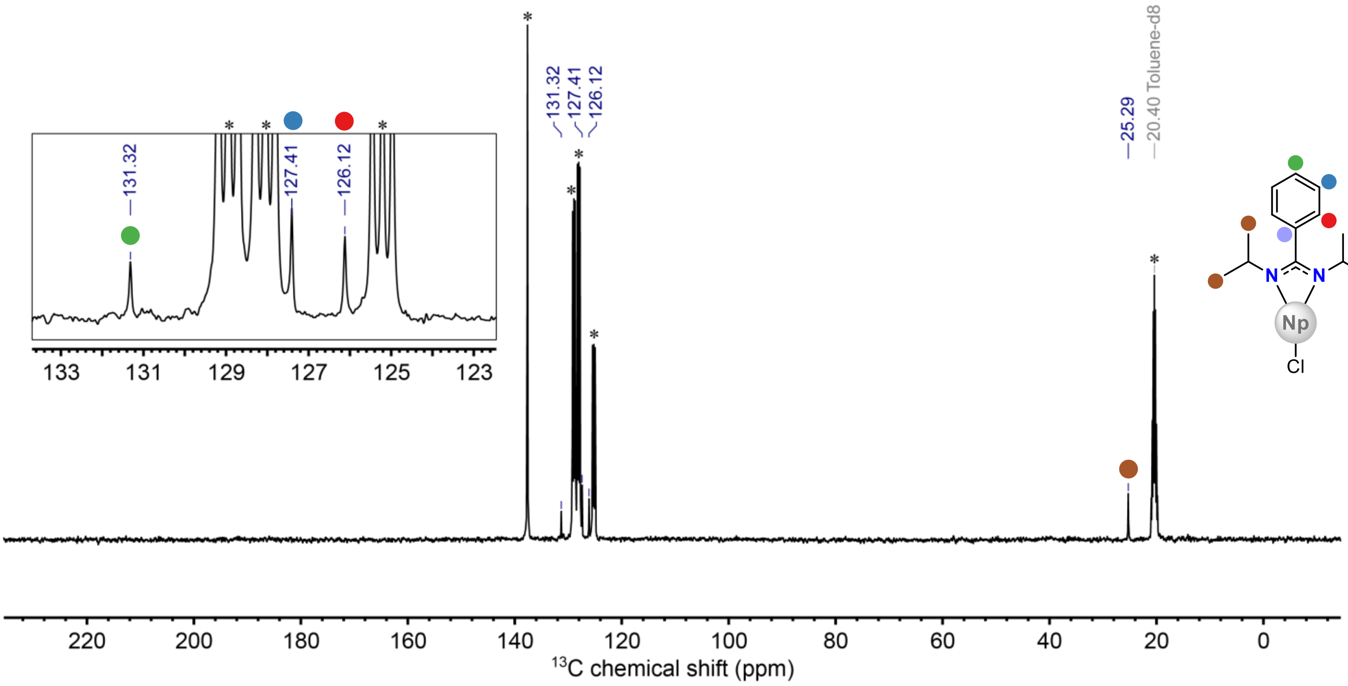

Figure S106. ^{13}C-NMR spectrum of [NpCl(iPr$_2$BA)$_3$] (**8-Cl**) in toluene-d_8 (indicated by asterisks) at 363 K. A scaled-up version of the compound peaks is shown as an insert above the main spectrum for clarity. Signals for the N***C***N, (CH$_3$)$_2$***C***HN carbons could not be observed. In the case of the ***i***-Ph carbon, it exhibited a strong correlation signal at 78.1 ppm in the ^{1}H-^{13}C HMBC spectrum, but no peak was found in the ^{13}C spectrum.

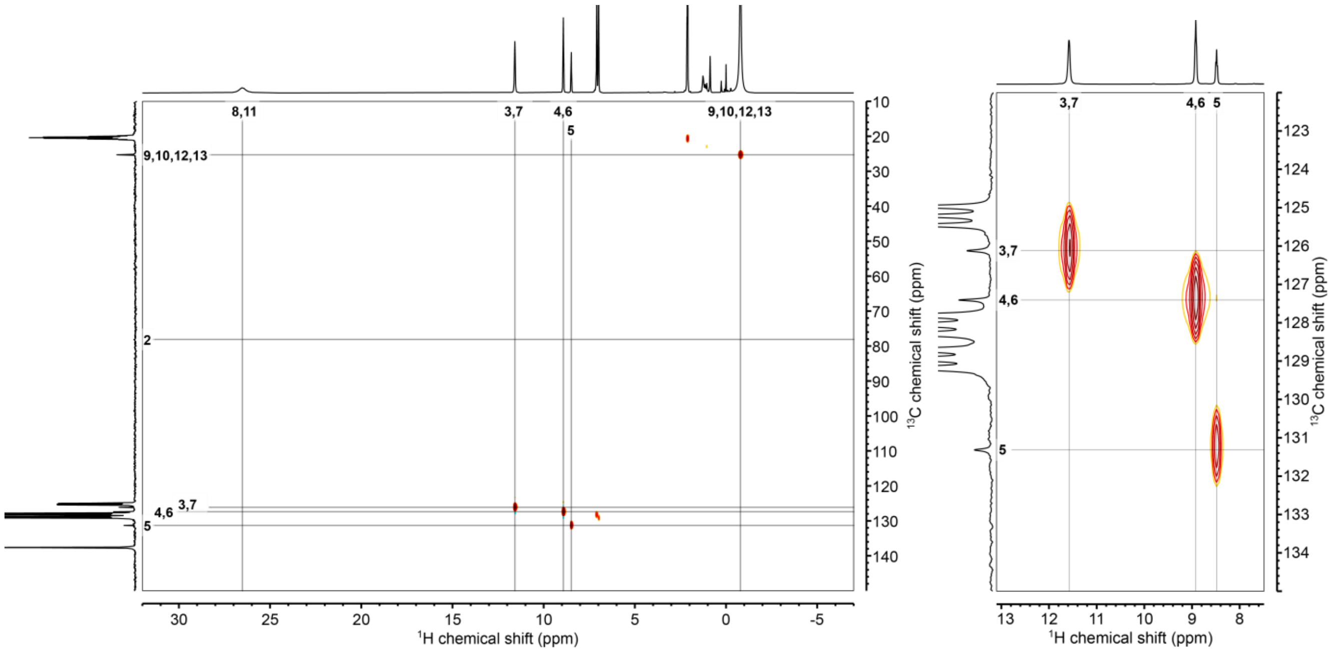

Figure S107. ^{1}H-^{13}C HSQC spectra of [NpCl(iPr$_2$BA)$_3$] (**8-Cl**) in toluene-d_8 at 363 K.

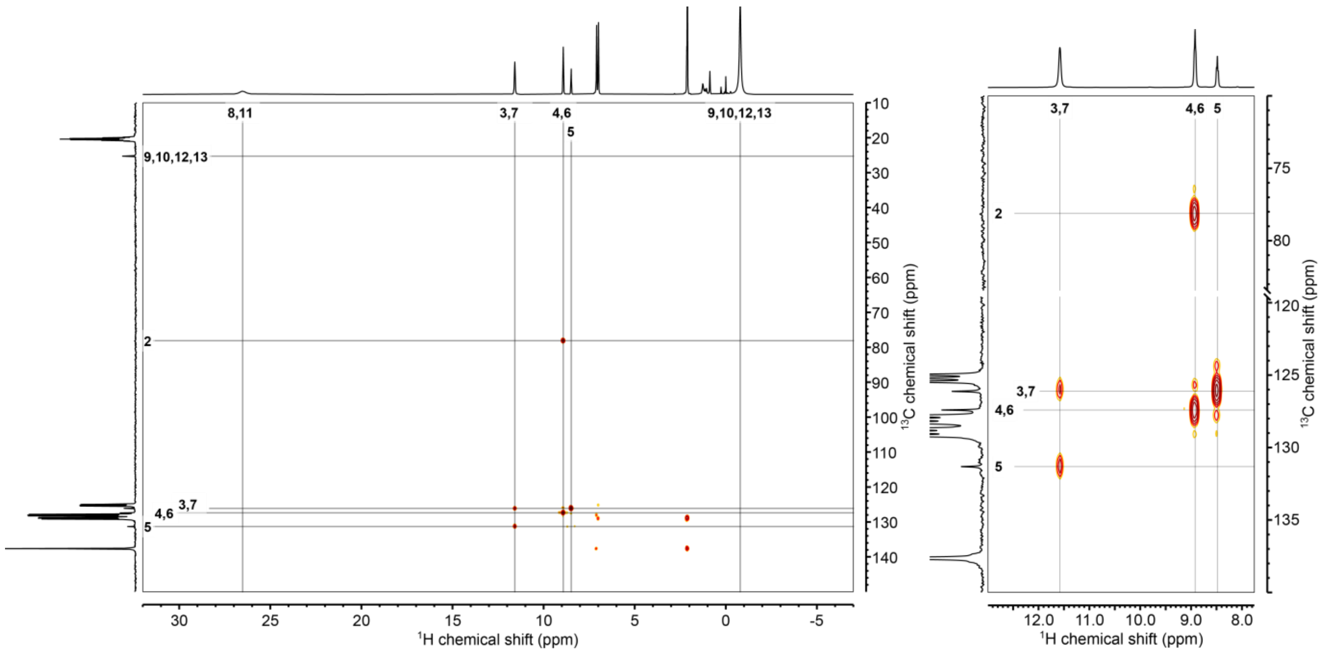

Figure S108. ^{1}H-^{13}C HMBC spectra of [NpCl(*i*Pr$_2$BA)$_3$] (**8-Cl**) in toluene-d_8 at 363 K.

8. Literature

(1) SMART Software Ver. 5.625 for the CCD Detector System; Bruker AXS Inc.: Madison, WI, 2001.

(2) SAINTPLUS Software Ver. 6.22 for the CCD Detector System; Bruker AXS Inc.: Madison, WI, 2001.

(3) Blessing, R. H. An Empirical Correction for Absorption Anisotropy. *Acta Cryst. A* **1995**, *51* (1), 33–38, DOI: 10.1107/S0108767394005726

(4) Sheldrick, G. M. SHELXTL, Ver. 2013/4. Universität Göttingen: Göttingen, Germany, 2013.

(5) Sheldrick, G. M. A Short History of SHELX. *Acta Cryst. A* **2008**, *64* (1), 112–122, DOI: 10.1107/S0108767307043930

(6) Farrugia, L. J. WinGX and ORTEP for Windows: An Update. *J. Appl. Cryst.* **2012**, *45* (4), 849–854, DOI: 10.1107/S0021889812029111

(7) Spek, A. PLATON, An Integrated Tool for the Analysis of the Results of a Single Crystal Structure Determination. *Acta Cryst. A* **1990**, *46* (s1), c34, DOI: 10.1107/S0108767390099780

(8) Parkin, S.; Moezzi, B.; Hope, H. XABS2: An Empirical Absorption Correction Program. *J. Appl. Cryst.* **1995**, *28* (1), 53–56, DOI: 10.1107/S0021889894009428

(9) Dröse, P.; Hrib, C. G.; Blaurock, S.; Edelmann, F. T. Tris(N,N′-Diisopropyl-benzamidinato)Cerium(III). *Acta Cryst. E* **2010**, *66* (11), m1474–m1474, DOI: 10.1107/S1600536810042704

(10) Klaus brandenburg. Diamond, Ver. 3.2f. Crystal Impact GbR: Bonn, Germany, 2010.

(11) Shannon, R. D. Revised Effective Ionic Radii and Systematic Studies of Interatomic Distances in Halides and Chalcogenides. *Acta Cryst. A* **1976**, *32* (5), 751–767, DOI: 10.1107/S0567739476001551

(12) Minasian, S. G.; Boland, K. S.; Feller, R. K.; Gaunt, A. J.; Kozimor, S. A.; May, I.; Reilly, S. D.; Scott, B. L.; Shuh, D. K. Synthesis and Structure of $(Ph_4P)_2MCl_6$ (M = Ti, Zr, Hf, Th, U, Np, Pu). *Inorg. Chem.* **2012**, *51* (10), 5728–5736, DOI: 10.1021/ic300179d

(13) Raymond, K. N.; Eigenbrot, C. W. Jr. Structural Criteria for the Mode of Bonding of Organoactinides and -Lanthanides and Related Compounds. *Acc. Chem. Res.* **1980**, *13* (8), 276–283, DOI: 10.1021/ar50152a005

(14) Bienfait, A. M.; Wolf, B. M.; Törnroos, K. W.; Anwander, R. Trivalent Rare-Earth-Metal Bis(Trimethylsilyl)Amide Halide Complexes by Targeted Oxidations. *Inorg. Chem.* **2018**, *57* (9), 5204–5212, DOI: 10.1021/acs.inorgchem.8b00240

(15) Herrmann, W. A.; Anwander, R.; Munck, F. C.; Scherer, W.; Dufaud, V.; Huber, N. W.; Artus, G. R. J. Lanthanoid complexes. Pt. 9. Reactivity control of lanthanoid amides through ligand effects: Synthesis and structures of sterically congested alkoxy complexes. Lanthanoiden-Komplexe. T. 9. Reaktivitaetsbestimmender Einfluss der Ligandenkonstitution bei Seltenerdamiden: Herstellung und Struktur sterisch ueberladener Alkoxid-Komplexe. *Z. Naturforsch., B: Chem. Sci.* **1994**, *49:12*.

(16) Lüert, D.; Herbst-Irmer, R.; Stalke, D. Structural and Magnetic Studies on Lanthanide Bis(Benzoxazol-2-Yl)Methanides. *Eur. J. Inorg. Chem.* **2021**, *2021* (48), 5085–5090, DOI: 10.1002/ejic.202100822

(17) B. Hitchcock, P.; G. Hulkes, A.; F. Lappert, M.; Li, Z. Cerium(III) Dialkyl Dithiocarbamates from $[Ce\{N(SiMe_3)_2\}_3]$ and Tetraalkylthiuram Disulfides, and $[Ce(\kappa^2\text{-}S_2CNEt_2)_4]$ from the Ce^{III} Precursor; Tb^{III} and Nd^{III} Analogues. *Dalton Trans.* **2004**, *0* (1), 129–136, DOI: 10.1039/B311397C

(18) Brady, E. D.; Clark, D. L.; Gordon, J. C.; Hay, P. J.; Keogh, D. W.; Poli, R.; Scott, B. L.; Watkin, J. G. Tris(Bis(Trimethylsilyl)Amido)Samarium: X-Ray Structure and DFT Study. *Inorg. Chem.* **2003**, *42* (21), 6682–6690, DOI: 10.1021/ic0341690

(19) Andersen, R. A.; Templeton, D. H.; Zalkin, A. Structure of Tris(Bis(Trimethylsilyl)Amido)Neodymium(III), $Nd[N(Si(CH_3)_3)_2]_3$. *Inorg. Chem.* **1978**, *17* (8), 2317–2319, DOI: 10.1021/ic50186a062

(20) William S. Rees, J.; Just, O.; Derveer, D. S. V. Molecular Design of Dopant Precursors for Atomic Layer Epitaxy of SrS:Ce. *J. Mater. Chem.* **1999**, *9* (1), 249–252, DOI: 10.1039/A805757E

(21) Wursthorn, L.; Beckett, K.; Rothbaum, J. O.; Cywar, R. M.; Lincoln, C.; Kratish, Y.; Marks, T. J. Selective Lanthanide-Organic Catalyzed Depolymerization of Nylon-6 to ϵ-Caprolactam. *Angew. Chem. Int. Ed.* **2023**, *62* (4), e202212543, DOI: 10.1002/anie.202212543

(22) Gaunt, A. J.; Enriquez, A. E.; Reilly, S. D.; Scott, B. L.; Neu, M. P. Structural Characterization of $Pu[N(SiMe_3)_2]_3$, a Synthetically Useful Nonaqueous Plutonium(III) Precursor. *Inorg. Chem.* **2008**, *47* (1), 26–28, DOI: 10.1021/ic701673e

(23) Goodwin, C. A. P.; Janicke, M. T.; Scott, B. L.; Gaunt, A. J. $[AnI_3(THF)_4]$ (An = Np, Pu) Preparation Bypassing An^0 Metal Precursors: Access to Np^{3+}/Pu^{3+} Nonaqueous and Organometallic Complexes. *J. Am. Chem. Soc.* **2021**, *143* (49), 20680–20696, DOI: 10.1021/jacs.1c07967

(24) Stewart, J. L.; Andersen, R. A. Trivalent Uranium Chemistry: Molecular Structure of $[(Me_3Si)_2N]_3U$. *Polyhedron* **1998**, *17* (5), 953–958, DOI: 10.1016/S0277-5387(97)00244-1

(25) B. Jones, M.; J. Gaunt, A.; C. Gordon, J.; Kaltsoyannis, N.; P. Neu, M.; L. Scott, B. Uncovering F-Element Bonding Differences and Electronic Structure in a Series of 1:3 and 1:4 Complexes with a Diselenophosphinate Ligand. *Chem. Sci.* **2013**, *4* (3), 1189–1203, DOI: 10.1039/C2SC21806B

(26) Gaunt, A. J.; Reilly, S. D.; Enriquez, A. E.; Scott, B. L.; Ibers, J. A.; Sekar, P.; Ingram, K. I. M.; Kaltsoyannis, N.; Neu, M. P. Experimental and Theoretical Comparison of Actinide and Lanthanide Bonding in $M[N(EPR_2)_2]_3$ Complexes (M = U, Pu, La, Ce; E = S, Se, Te; R = Ph, *i*Pr, H). *Inorg. Chem.* **2008**, *47* (1), 29–41, DOI: 10.1021/ic701618a

(27) Coles, M. P.; Hitchcock, P. B.; Lappert, M. F.; Protchenko, A. V. Syntheses and Structures of the Crystalline, Highly Crowded 1,3-Bis(Trimethylsilyl)Cyclopentadienyls $[MCp''_3]$ (M = Y, Er, Yb), $[PbCp''_2]$, $[YCp''_2(\mu\text{-}OH)_2]$, $[(ScCp''_2)_2(\mu\text{-}\eta^2\text{:}\eta^2\text{-}C_2H_4)]$, $[YbCp''_2Cl(\mu\text{-}Cl)K(18\text{-}Crown\text{-}6)]$, and $[KCp''_\infty]$. *Organometallics* **2012**, *31* (7), 2682–2690, DOI: 10.1021/om2009364

(28) Xie, Z.; Chui, K.; Liu, Z.; Xue, F.; Zhang, Z.; Mak, T. C. W.; Sun, J. Systematic Studies on the Reactions of Lanthanide Trichlorides with Na[1,3-Bis(Trimethylsilyl) Cyclopentadienyl]. Crystal Structures of $[1,3\text{-}(Me_3Si)_2C_5H_3]_3Ln$ (Ln = La, Nd, Gd, Dy). *J. Organomet. Chem.* **1997**, *549* (1), 239–244, DOI: 10.1016/S0022-328X(97)00489-0

(29) Evans, W. J.; Keyer, R. A.; Ziller, J. W. Synthesis and Reactivity of Bis(Trimethylsilyl)Cyclopentadienyl Samarium Complexes Including the X-Ray Crystal Structure of $[(Me_3Si)_2C_5H_3]_3Sm$. *J. Organomet. Chem.* **1990**, *394* (1), 87–97, DOI: 10.1016/0022-328X(90)87226-4

(30) Stults, S. D.; Andersen, R. A.; Zalkin, A. Structural Studies on Cyclopentadienyl Compounds of Trivalent Cerium: Tetrameric $(MeC_5H_4)_3Ce$ and Monomeric $(Me_3SiC_5H_4)_3Ce$ and $[(Me_3Si)_2C_5H_3]_3Ce$ and Their Coordination Chemistry. *Organometallics* **1990**, *9* (1), 115–122, DOI: 10.1021/om00115a018

(31) Windorff, C. J.; Chen, G. P.; Cross, J. N.; Evans, W. J.; Furche, F.; Gaunt, A. J.; Janicke, M. T.; Kozimor, S. A.; Scott, B. L. Identification of the Formal +2 Oxidation State of Plutonium: Synthesis and Characterization of $\{Pu^{II}[C_5H_3(SiMe_3)_2]_3\}^-$. *J. Am. Chem. Soc.* **2017**, *139* (11), 3970–3973, DOI: 10.1021/jacs.7b00706

(32) del Mar Conejo, M.; Parry, J. S.; Carmona, E.; Schultz, M.; Brennann, J. G.; Beshouri, S. M.; Andersen, R. A.; Rogers, R. D.; Coles, S.; Hursthouse, M. B. Carbon Monoxide and Isocyanide Complexes of Trivalent Uranium Metallocenes. *Chem. - Eur. J.* **1999**, *5* (10), 3000–3009, DOI: 10.1002/(SICI)1521-3765(19991001)5:10<3000::AID-CHEM3000>3.0.CO;2-Q

(33) Langeslay, R. R.; Fieser, M. E.; Ziller, J. W.; Furche, F.; Evans, W. J. Synthesis, Structure, and Reactivity of Crystalline Molecular Complexes of the $\{[C_5H_3(SiMe_3)_2]_3Th\}^{1-}$ Anion Containing Thorium in the Formal +2 Oxidation State. *Chem. Sci.* **2014**, *6* (1), 517–521, DOI: 10.1039/C4SC03033H

(34) Balasubramani, S. G.; Chen, G. P.; Coriani, S.; Diedenhofen, M.; Frank, M. S.; Franzke, Y. J.; Furche, F.; Grotjahn, R.; Harding, M. E.; Hättig, C.; Hellweg, A.; Helmich-Paris, B.; Holzer, C.; Huniar, U.; Kaupp, M.; Marefat Khah, A.; Karbalaei Khani, S.; Müller, T.; Mack, F.; Nguyen, B. D.; Parker, S. M.; Perlt, E.; Rappoport, D.; Reiter, K.; Roy, S.; Rückert, M.; Schmitz, G.; Sierka, M.; Tapavicza, E.; Tew, D. P.; van Wüllen, C.; Voora, V. K.; Weigend, F.; Wodyński, A.; Yu, J. M. TURBOMOLE: Modular Program Suite for Ab Initio Quantum-Chemical and Condensed-Matter Simulations. *J. Chem. Phys.* **2020**, *152* (18), 184107, DOI: 10.1063/5.0004635

(35) Klamt, A.; Schüürmann, G. COSMO: A New Approach to Dielectric Screening in Solvents with Explicit Expressions for the Screening Energy and Its Gradient. *J. Chem. Soc., Perkin Trans. 2* **1993**, No. 5, 799–805, DOI: 10.1039/P29930000799

(36) Bader, R. F. W. *Atoms in Molecules: A Quantum Theory*; Clarendon Press, 1990.

(37) Douglas, M.; Kroll, N. M. Quantum Electrodynamical Corrections to the Fine Structure of Helium. *Ann. Phys.* **1974**, *82* (1), 89–155, DOI: 10.1016/0003-4916(74)90333-9

(38) Jansen, G.; Hess, B. A. Revision of the Douglas-Kroll Transformation. *Phys. Rev. A* **1989**, *39* (11), 6016–6017, DOI: 10.1103/PhysRevA.39.6016

(39) Neese, F. Software Update: The ORCA Program System—Version 5.0. *Wiley Interdiscip. Rev. Comput. Mol. Sci.* **2022**, *12* (5), e1606, DOI: 10.1002/wcms.1606

(40) Lu, T.; Chen, F. Multiwfn: A Multifunctional Wavefunction Analyzer. *J. Comput. Chem.* **2012**, *33* (5), 580–592, DOI: 10.1002/jcc.22885

(41) Humphrey, W.; Dalke, A.; Schulten, K. VMD: Visual Molecular Dynamics. *J. Mol. Graph.* **1996**, *14* (1), 33–38, DOI: 10.1016/0263-7855(96)00018-5

(42) Autillo, M.; Guerin, L.; Dumas, T.; Grigoriev, M. S.; Fedoseev, A. M.; Cammelli, S.; Solari, P. L.; Guillaumont, D.; Guilbaud, P.; Moisy, P.; Bolvin, H.; Berthon, C. Insight of the Metal–Ligand Interaction in f-Element Complexes by Paramagnetic NMR Spectroscopy. *Chem. - Eur. J.* **2019**, *25* (17), 4435–4451, DOI: 10.1002/chem.201805858

(43) Piguet, C.; Geraldes, C. F. G. C. Paramagnetic NMR Lanthanide Induced Shifts for Extracting Solution Structures. In *Handbook on the Physics and Chemistry of Rare Earths*; Gschneidner, K. A., Bünzli, J.-C. G., Pecharsky, V. K., Eds.; Handbook on the Physics and Chemistry of Rare Earths; Elsevier, 2003; Vol. 33, pp 353–463, DOI: 10.1016/S0168-1273(02)33005-8

(44) Suturina, E. A.; Mason, K.; Geraldes, C. F. G. C.; Kuprov, I.; Parker, D. Beyond Bleaney's Theory: Experimental and Theoretical Analysis of Periodic Trends in Lanthanide-Induced Chemical Shift. *Angew. Chem. Int. Ed.* **2017**, *56* (40), 12215–12218, DOI: 10.1002/anie.201706931

(45) Autillo, M.; Islam, Md. A.; Héron, J.; Guérin, L.; Acher, E.; Tamain, C.; Illy, M.-C.; Moisy, P.; Colineau, E.; Griveau, J.-C.; Berthon, C.; Bolvin, H. Temperature Dependence of 1H Paramagnetic Chemical Shifts in Actinide Complexes, Beyond Bleaney's Theory: The $An^{VI}O_2^{2+}$–Dipicolinic Acid Complexes (An=Np, Pu) as an Example. *Chem. - Eur. J.* **2021**, *27* (24), 7138–7153, DOI: 10.1002/chem.202005147

(46) Di Pietro, S.; Piano, S. L.; Di Bari, L. Pseudocontact Shifts in Lanthanide Complexes with Variable Crystal Field Parameters. *Coord. Chem. Rev.* **2011**, *255* (23), 2810–2820, DOI: 10.1016/j.ccr.2011.05.010

(47) Pinkerton, A. A.; Rossier, M.; Spiliadis, S. Lanthanide-Induced Contact Shifts. the Average Electron Spin Polarization, Theory and Experiment. *J. Magn. Reson. (1969)* **1985**, *64* (3), 420–425, DOI:

10.1016/0022-2364(85)90104-0

(48) Castro, G.; Regueiro-Figueroa, M.; Esteban-Gómez, D.; Pérez-Lourido, P.; Platas-Iglesias, C.; Valencia, L. Magnetic Anisotropies in Rhombic Lanthanide(III) Complexes Do Not Conform to Bleaney's Theory. *Inorg. Chem.* **2016**, *55* (7), 3490–3497, DOI: 10.1021/acs.inorgchem.5b02918

(49) Golding, R. M.; Pyykkö, P. On the Theory of Pseudocontact N.M.R. Shifts Due to Lanthanide Complexes. *Mol. Phys.* **1973**, *26* (6), 1389–1396, DOI: 10.1080/00268977300102561

(50) Bleaney, B.; Dobson, C. M.; Levine, B. A.; Martin, R. B.; Williams, R. J. P.; Xavier, A. V. Origin of Lanthanide Nuclear Magnetic Resonance Shifts and Their Uses. *J. Chem. Soc., Chem. Commun.* **1972**, No. 13, 791b–793, DOI: 10.1039/C3972000791B

(51) Platas, C.; Avecilla, F.; de Blas, A.; Geraldes, C. F. G. C.; Rodríguez-Blas, T.; Adams, H.; Mahía, J. 1H NMR in Solution and Solid State Structural Study of Lanthanide(III) Cryptates. *Inorg. Chem.* **1999**, *38* (13), 3190–3199, DOI: 10.1021/ic981314e
